# Supplementary material for: Barcode-free hit discovery from massive libraries enabled by automated small molecule structure annotation
Source: Nat Commun. 2025 Oct 27;16:9479. doi: 10.1038/s41467-025-65282-1 (PMC12559350; doi:10.1038/s41467-025-65282-1)
Supplement: Supplementary file 1 — Supplementary Information [file 41467_2025_65282_MOESM1_ESM.pdf]

Supplementary Materials for

Barcode free hit discovery from massive libraries enabled by automated small molecule structure annotation

Edith van der Nol[1,2]+, Nils Alexander Haupt[3]+, Qing Qing Gao[1], Benthe A.M. Smit[1], Martin Andre Hoffmann[4], Martin Engler-Lukajewski[4], Marcus Ludwig[4], Sean McKenna[1,2], J. Miguel Mata[1,2], Olivier J. M. Béquignon[1], Gerard van Westen[1], Tiemen J. Wendel[2,5], Sylvie M. Noordermeer [2,5], Sebastian Böcker[3]*, Sebastian Pomplun[1,2]*

[1] LACDR, Leiden University; Leiden, 2333 CC, The Netherlands

[2] Oncode Institute; Utrecht, 3521 AL, The Netherlands

[3] Chair for Bioinformatics, Institute for Computer Science, Friedrich Schiller University Jena, Jena, 07737, Germany

[4] Bright Giant GmbH, Hans-Knöll-Straße 6, 07745 Jena, Germany

[5] Department of Human Genetics, Leiden University Medical Center; Leiden, 2333 ZA, The Netherlands,

*Corresponding authors.
Email: sebastian.boecker@uni-jena.de, s.j.pomplun@lacdr.leidenuniv.nl

+These authors contributed equally to this work

Table of Contents

1. Supplementary figures 4
   1.1. General procedure nucleophilic aromatic substitution 4
   1.2. General procedure heterocyclization 6
   1.3. General procedure Suzuki-Miyaura coupling 8
2. Abbreviations 23
3. Material and methods 24
   3.1. Reagents and supplies 24
   3.2. Instrumentation 24
   3.3. General procedures 25
   3.4. Software 27
4. COMET 27
   4.1. Feature Filtering 30
   4.2. Fragmentation pattern analysis of Mini-SELs 1-3 30
   4.3. Annotation Procedure 31
   4.4. Fragmentation Analysis 31
   4.5. Ranking of candidate structures with EPIMETHEUS 33
5. Building block scoring & selection 35
6. Library synthesis and characterization 36
   6.1. SEL 1: Carboxylic acid library 36
   6.2. SEL 2: Benzimidazole library 37
   6.3. SEL 3: Suzuki-Miyaura library 38
   6.4. SEL 4: Focused 4.000 membered library 39
7. Building block synthesis 40
   7.1. CA131 40
   7.2. CA132 42
8. Affinity selection against CAIX 43
   8.1. Procedure 43
   8.2. Screening of SEL 1 against CAIX 43
      8.2.1. List of hit structures 43
      8.2.2. Enrichment plots 46
      8.2.3. Hit-Identification (LC-MS/MS) 47
      8.2.4. Hit re-synthesis 48
      8.2.5. Hit validation: Biolayer interferometry 49

8.3. Screening of SEL 2 against CAIX ... 50
8.3.1. List of hit structures ... 50
8.3.2. Enrichment plots ... 52
8.3.3. Hit-Identification (LC-MS/MS) ... 52
8.3.4. Synthesis of hit structures ... 53
8.3.5. Hit validation: Biolayer interferometry ... 55
8.4. Screening of SEL 3 against CAIX ... 55
8.4.1. List of hit structures ... 55
8.4.2. Enrichment plots ... 58
8.4.3. Hit-Identification (LC-MS/MS) ... 58
8.4.4. Synthesis of hit structures ... 58
8.4.5. Hit validation: Biolayer interferometry ... 59
9. Affinity selection against FEN1 ... 60
9.1. Procedure ... 60
9.2. Screening of SEL 1 against FEN1 ... 61
9.2.1. Hit-Identification (LC-MS/MS) ... 61
9.2.2. Synthesis of hit structures ... 62
10. Appendix ... 66
10.1. List of building blocks ... 66
11. Supplementary NMR data ... 82
12. Supplementary LC-MS data ... 89
12.1. LC-MS data from the scope of primary amines ... 90
12.2. LC-MS data from the scope of aldehydes ... 120
12.3. LC-MS data from the scope of aryl bromides ... 152
12.4. LC-MS data from the scope of boronic acids ... 158
12.5. LC-MS data from the synthesis of CA131 and CA132 ... 187

1. Supplementary figures

A

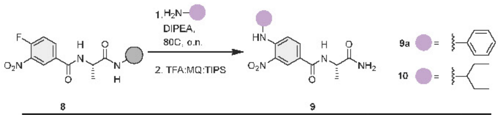

Entry	Temp (ºC)	Time	9a (%)[b]	10 (%)[b]
1	60	2h	11%	96%
2	60	o.n.	54%	62%
3	80	o.n.	85%	92%
4	100	o.n.	59%	86%

B

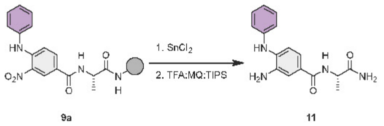

Entry	Temp (ºC)	Time	11 (%)[b]
1	r.t.	2h	41
2	r.t.	1d	93
3	r.t.	2d	79
4	35 °C	2h	76
5	35 °C	1d	64
6	35 °C	2d	64

C

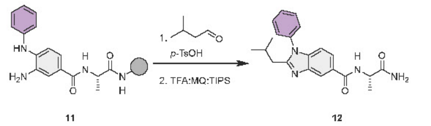

Entry	Acid/Base	Temp (ºC)	Solvent	12 (%)[b]
1	-	r.t.	DMF	26
2	Pyridine	r.t.	DMF	43
3	-	r.t.	Pyridine	55
4	p-TsOH	r.t.	DMF	57
5	p-TsOH	37 °C	DMF	58

General reaction conditions:

A: Compound **8** (5 µmol, 1.0 eq.), amine (50 µmol, 10 eq.), DIPEA (50 µmol, 10 eq.) in DMF (100 µL)

B: Compound **9a** (5 µmol, 1.0 eq.), $SnCl_2$ (312.5 µmol, 62.5 eq.) in DMF (312 µL).

C: Compound **11** (5 µmol, 1.0 eq.), isovaleraldehyde (25 µmol, 5 eq.), acid/base (25 µmol, 5 eq.) in DMF (100 µL), r.t., o.n.

[b] Conversion was determined by LC-MS analysis.

Supplementary Fig 1. Optimization of trisubstituted benzimidazole synthesis on solid support. A) Optimization of the nucleophilic aromatic substitution reaction between 4-fluoro-3-nitrobenzoic acid and the primary amines aniline and pentan-3-amine B) Optimization of the reduction of nitro group. C) Optimization of the cyclization between compound 5 and isovaleraldehyde.

1.1. General procedure nucleophilic aromatic substitution

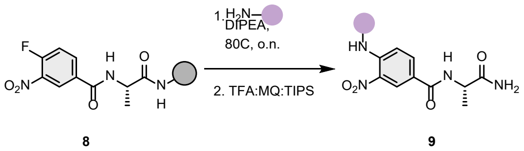

Starting material **8** was synthesized according to general procedure: Manual solid-phase synthesis (SPS) as described in section 3.3. TentaGel S NH_2 resin (90 µm, 0.24 mmol/g loading, 19 mg, 5 µmol, 1.0 eq.) was transferred to an eppendorf tube. Amine **9a-9cm** (50 µmol, 10 eq.) and DIPEA (8.71 µL, 50 µmol, 10 eq.) in DMF (100 µL, 0.5 M) were added to the resin. The mixture was shaken at 1 x g overnight at 80 °C. The resin was washed with DMF (5 x 2 mL) and DCM (5 x 2 mL) before cleaving with a solution of TFA:H_2O:TIPS (92.5:5:2.5) for 1h and washed once with a solution of TFA:H_2O:TIPS (92.5:5:2.5). LC-MS samples were prepared by adding 10 µL of the TFA mixture with 90 µL of MeCN:MQ:*t*BuOH (1:1:1) The reaction mixture was characterized by LC-MS.

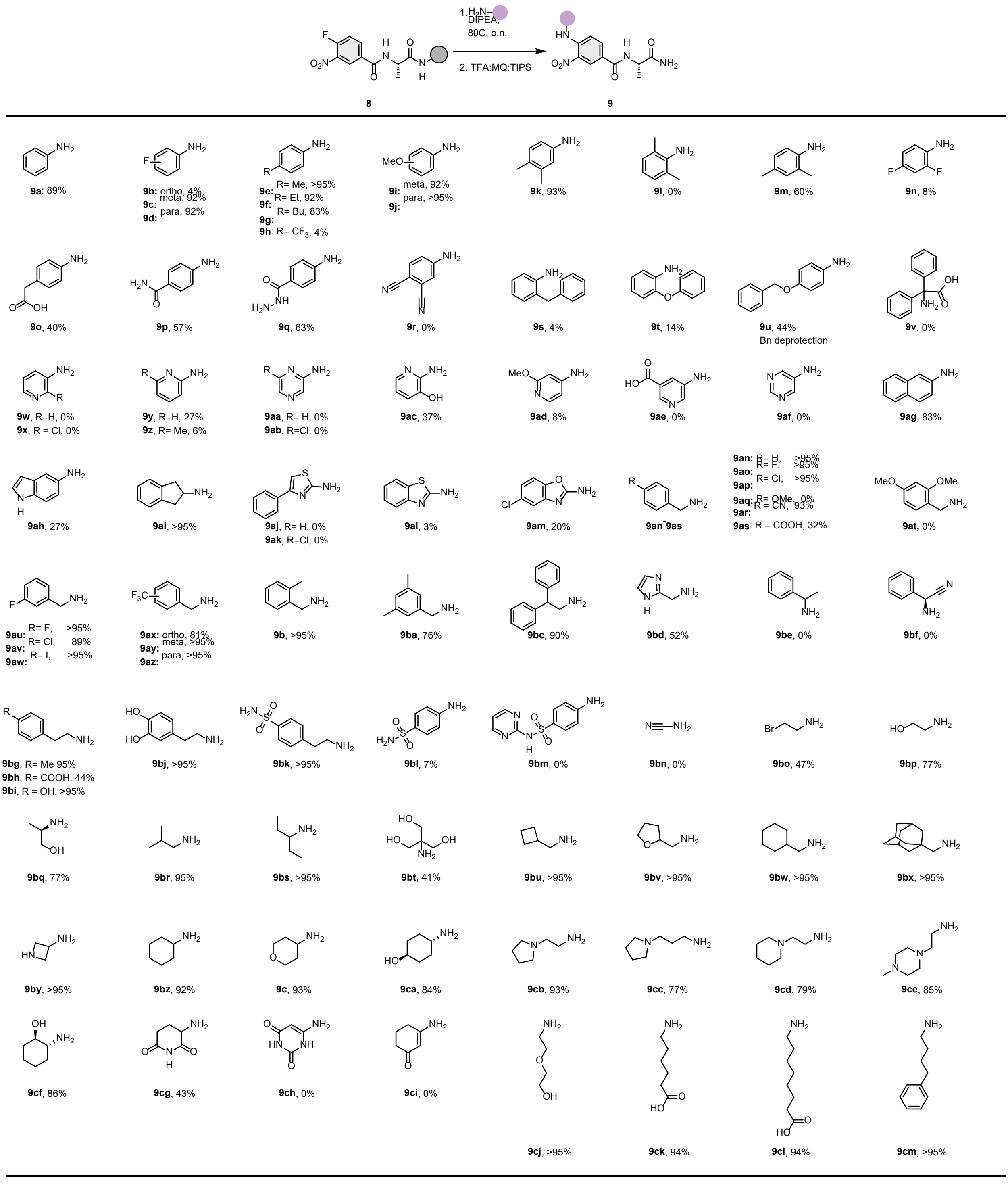

Supplementary Fig 2. Reaction scope for nucleophilic aromatic substitutions in the benzimidazole scaffold (primary amines)

a) Reaction conditions: Compound **8** (5 µmol, 1.0 eq.), amine **9a-9cm** (50 µmol, 10 eq.), DIPEA (50 µmol, 10 eq.) in DMF (100 µL), 80 ºC, overnight.
b) Conversion was determined by LC-MS analysis.

1.2. General procedure heterocyclization

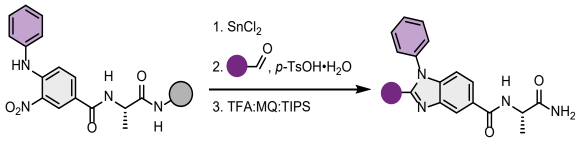

Starting material **9a** was synthesized as described in "1.1. General procedure nucleophilic aromatic substitution". A solution of 1.0 M $SnCl_2$ (62.5 eq.) in DMF was added to the resin (1.0 eq.). The mixture was incubated at r.t. overnight, whereafter the resin was washed 50% MQ (in DMF, 5x), with DMF (5x,) and DCM (5x). TentaGel S NH_2 resin (90 µm, 0.24 mmol/g loading, 19 mg, 5 µmol, 1.0 eq.) was transferred to an eppendorf tube. A solution the appropriate aldehyde **13a-13co** (0.25 M, 25 µmol, 5 eq.) and *p*-TsOH•H_2O (4.76 mg, 25 µmol, 5 eq.) in DMF (103 µL) was added to the resin. The mixture was incubated at r.t., overnight on 1 x g. After incubation, the resin was washed DMF (5x) and DCM (5x). The resin was incubated for 1h with a solution of TFA:H_2O:TIPS (92.5:5:2.5) and washed once with a solution of TFA:H_2O:TIPS (92.5:5:2.5). LC-MS samples were prepared by adding 10 µL of the TFA mixture with 90 µL of MeCN:MQ:*t*BuOH (1:1:1) The reaction mixture was characterized by LC-MS.

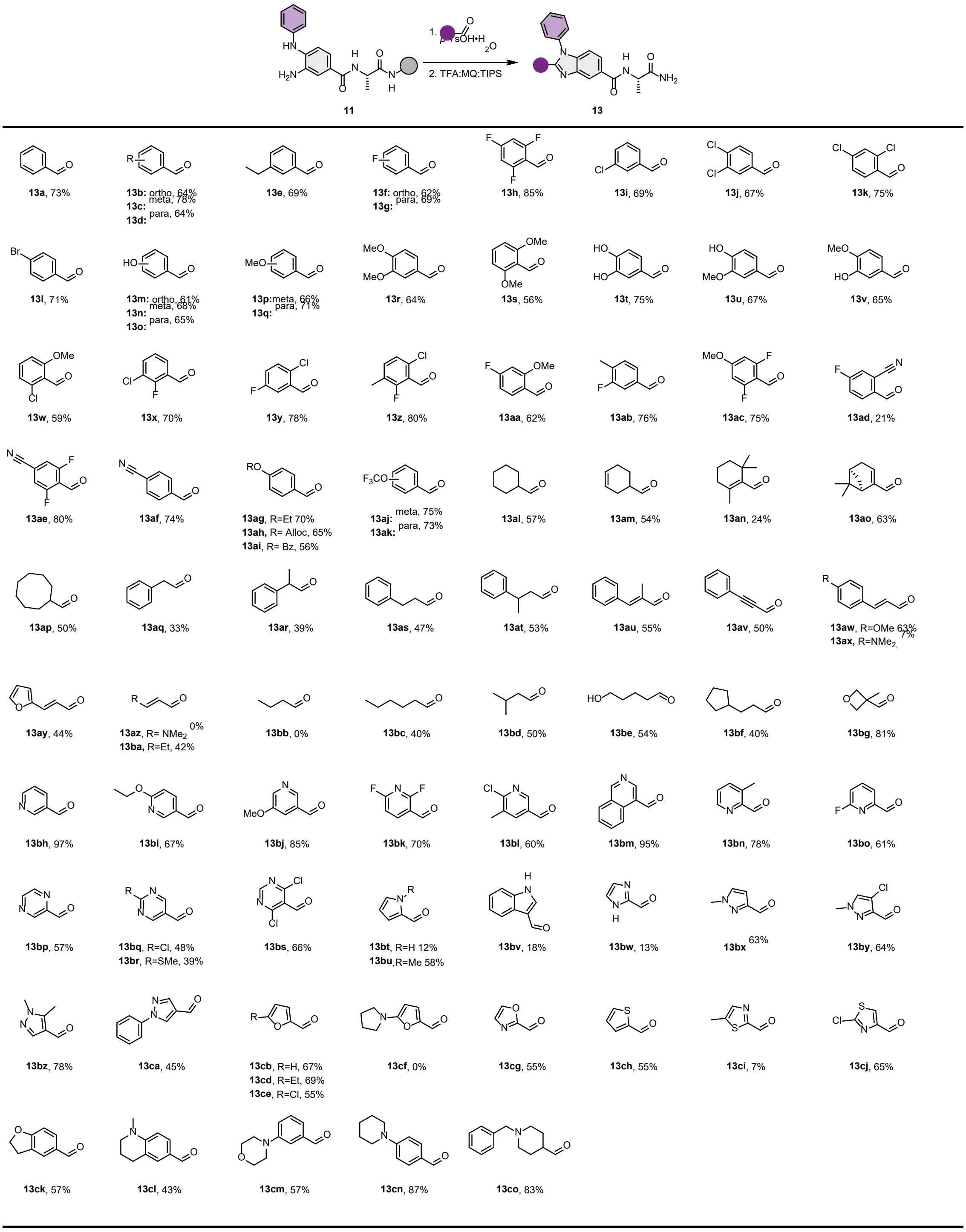

Supplementary Fig 3. Reaction scope for heterocyclization to the benzimidazole scaffold (aldehydes)

Reaction conditions: Compound **11** (5 µmol, 1.0 eq.), aldehyde (25 µmol, 5 eq.), *p*-TsOH•H_2O (25 µmol, 5 eq.) in DMF (100 µL), r.t., o.n. b) Conversion was determined by LC-MS analysis.

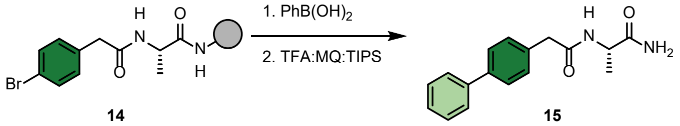

Entry	Catalyst	Ligand	Time (h)	15 (%)[b]
1	10 mol% $PdCl_2$	-	17	50
2	10 mol% $PdCl_2$	20 mol% Johnphos	17	59
3	10 mol% $PdCl_2$	20 mol% CyJohnphos	17	92
4	10 mol% $PdCl_2$	20 mol% Davephos	17	92
5	10 mol% $PdCl_2$	20 mol% Sphos	17	89
6	10 mol% $PdCl_2$	20 mol% Xphos	17	95
7	10 mol% $PdCl_2$	20 mol% Xphos	8	90
8	10 mol% $PdCl_2$	20 mol% Xphos	6	92
9	10 mol% $PdCl_2$	20 mol% Xphos	4	92
10	10 mol% $PdCl_2$	20 mol% Xphos	2	93
11	10 mol% $PdCl_2$	20 mol% Xphos	0.5	92

Supplementary Fig 4. Optimization of the Suzuki-Miyaura reaction on solid support
a) Reaction conditions: compound **14** (5 µmol, 1.0 eq), phenylboronic acid (10 µmol, 2.0 eq), $PdCl_2$ (0.5 µmol, 10 mol%), XPhos (0.1 µmol, 20 mol%), K_2CO_3 (10 µmol, 2 eq), solvent 9:1 DMF/H_2O (100 µL), 80 °C
b) Conversion was determined by LC-MS analysis.

1.3. General procedure Suzuki-Miyaura coupling

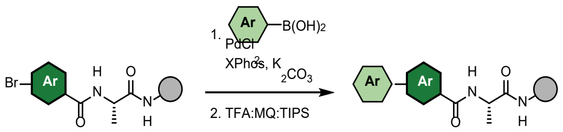

Starting material **16** was synthesized according to general procedure: Manual solid-phase synthesis (SPS) as described in section 3.3. TentaGel S NH_2 resin (90 µm, 0.24 mmol/g loading, 4.8 mg, 5 µmol, 1.0 eq.) was transferred to an eppendorf tube. Boronic acid (10 µmol, 2 eq.), $PdCl_2$ (0.09 mg, 0.5 µmol, 0.1 eq.), XPhos (0.48 mg, 1 µmol, 0.2 eq.) and K_2CO_3 (1.4 mg, 10 µmol, 2.0 eq.) were dissolved in DMF:H_2O (9:1, 100 µL) and added to the resin. The reaction was stirred at 1 x g on 80°C for 2 hours. The resin was washed DMF (5x) and DCM (5x). The resin was incubated for 1 hour with a solution of TFA:H_2O:TIPS (92.5:5:2.5) and washed once with a solution of TFA:H_2O:TIPS (92.5:5:2.5). LC-MS samples were prepared by adding 10 µL of the TFA mixture with 90 µL of MeCN:MQ:*t*BuOH (1:1:1). The reaction mixture was characterized by LC-MS.

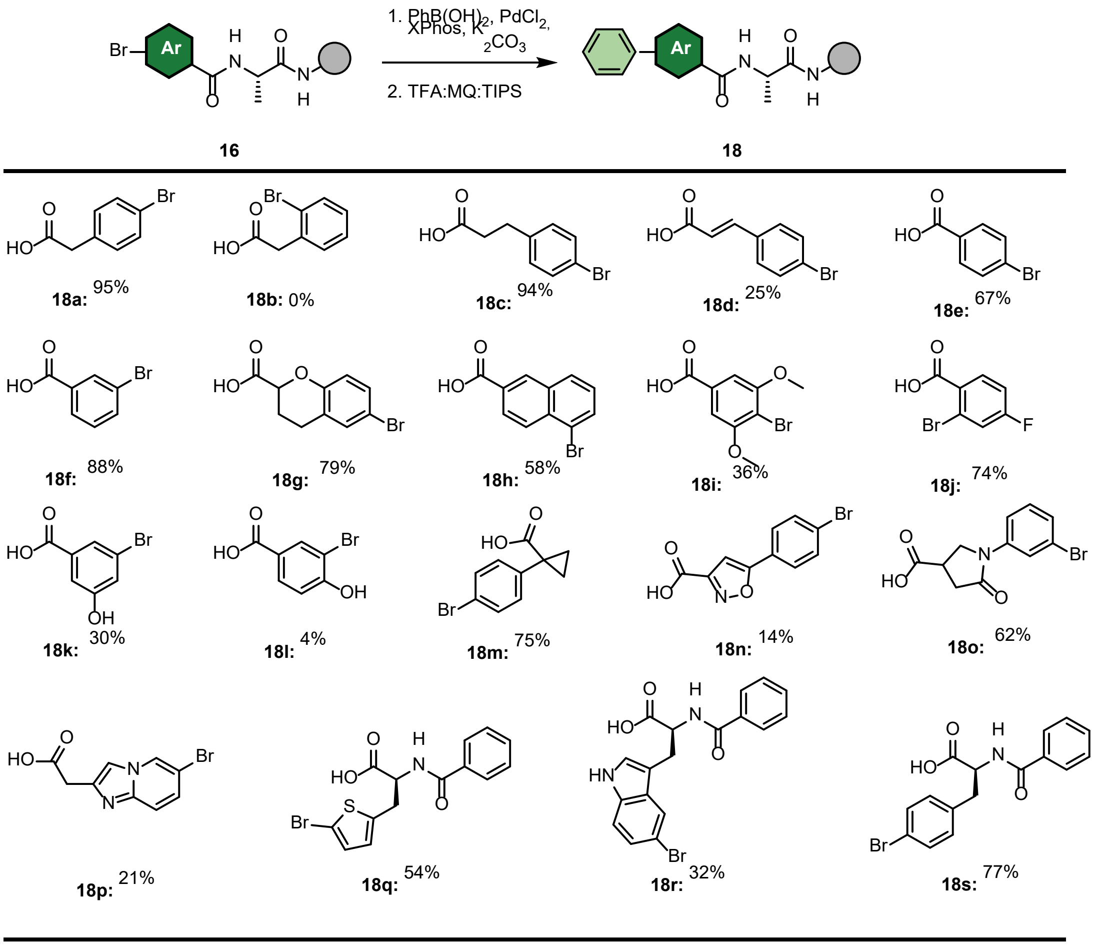

Supplementary Fig 5. Reaction scope of different aryl bromides in the Suzuki-Miyaura reaction

a) Reaction conditions: Compound **16** (5 µmol, 1.0 eq.), phenylboronic acid (10 µmol, 2 eq.), $PdCl_2$ (10 mol%), XPhos (20 mol%), K_2CO_3 (10 µmol, 2 eq.), 9:1 DMF/H_2O (100 µL), 80 °C o.n. b) Conversion was determined by LC-MS analysis.

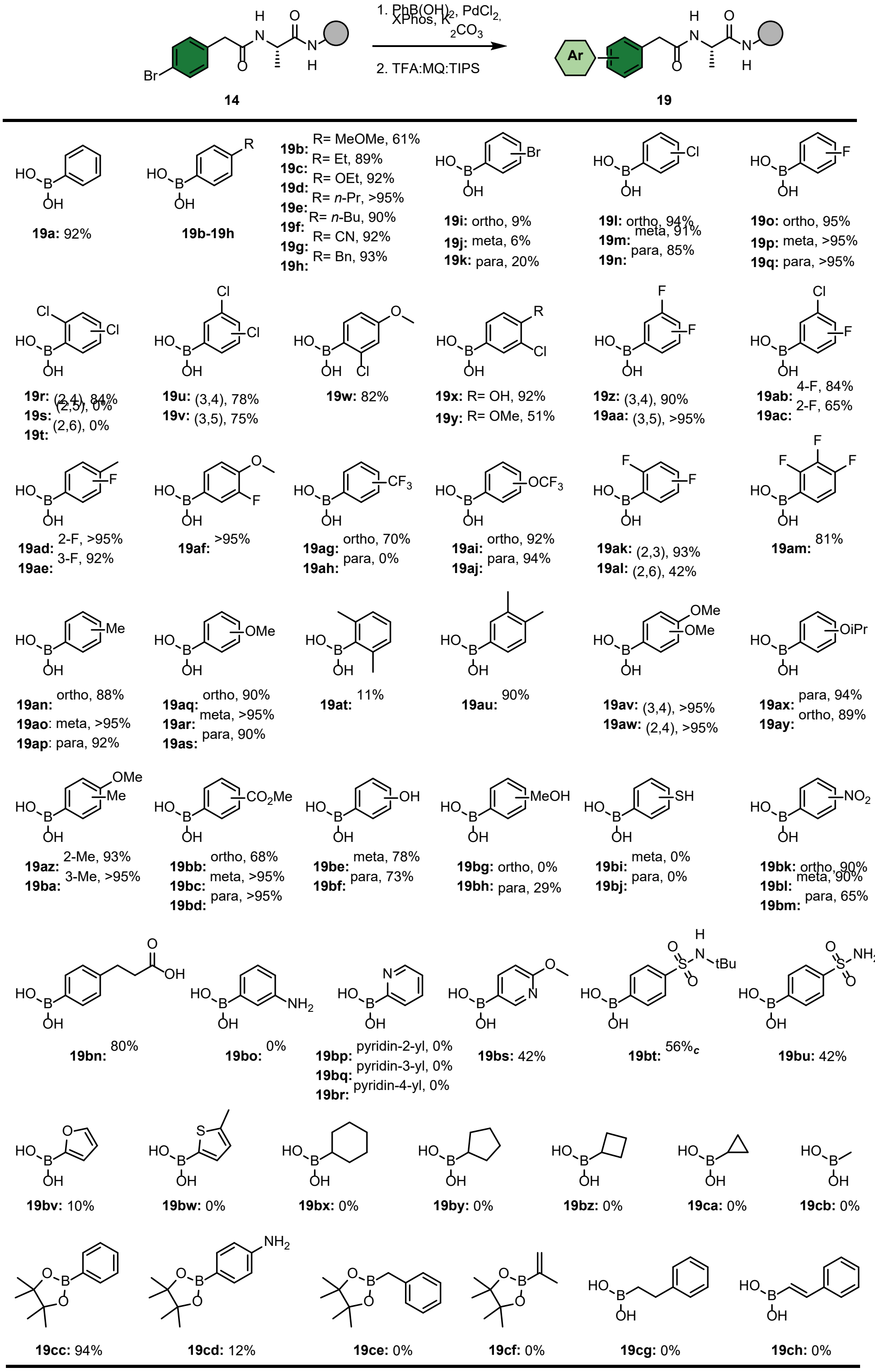

Supplementary Fig 6. Reaction scope of different boronic acids/esters in the Suzuki-Miyaura reaction

a) Reaction conditions: Compound **14** (5 µmol, 1.0 eq.), phenylboronic acid (10 µmol, 2 eq.), $PdCl_2$ (10 mol%), XPhos (20 mol%), K_2CO_3 (10 µmol, 2 eq.), 9:1 DMF/H_2O (100 µL), 80 °C o.n. b) Conversion was determined by LC-MS analysis [c] *t*Bu removed with TFA.

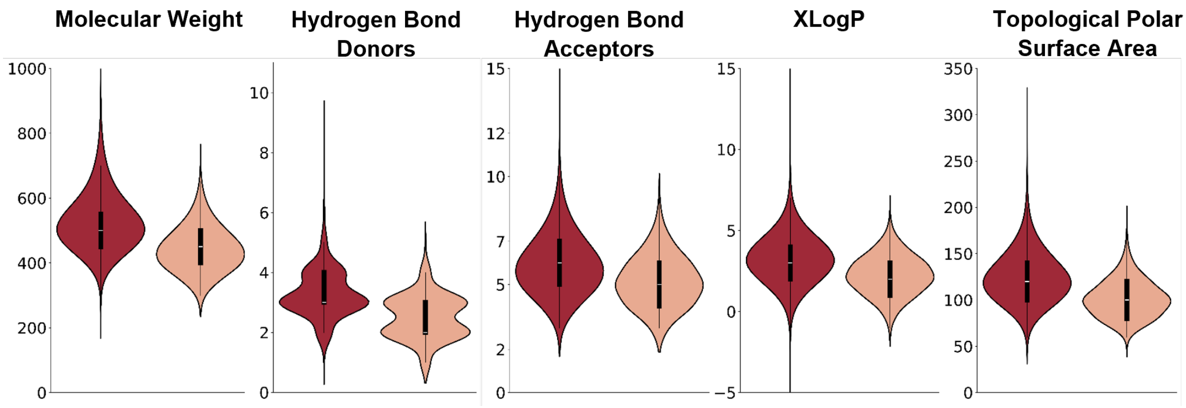

Supplementary Fig 7. Libraries with selected building blocks have optimized drug like properties compared to libraries with randomly chosen building blocks
Left: before selection (1000*1000*1000=1 billion membered library). Right = After selection (54*54*44 =128.304 membered library)

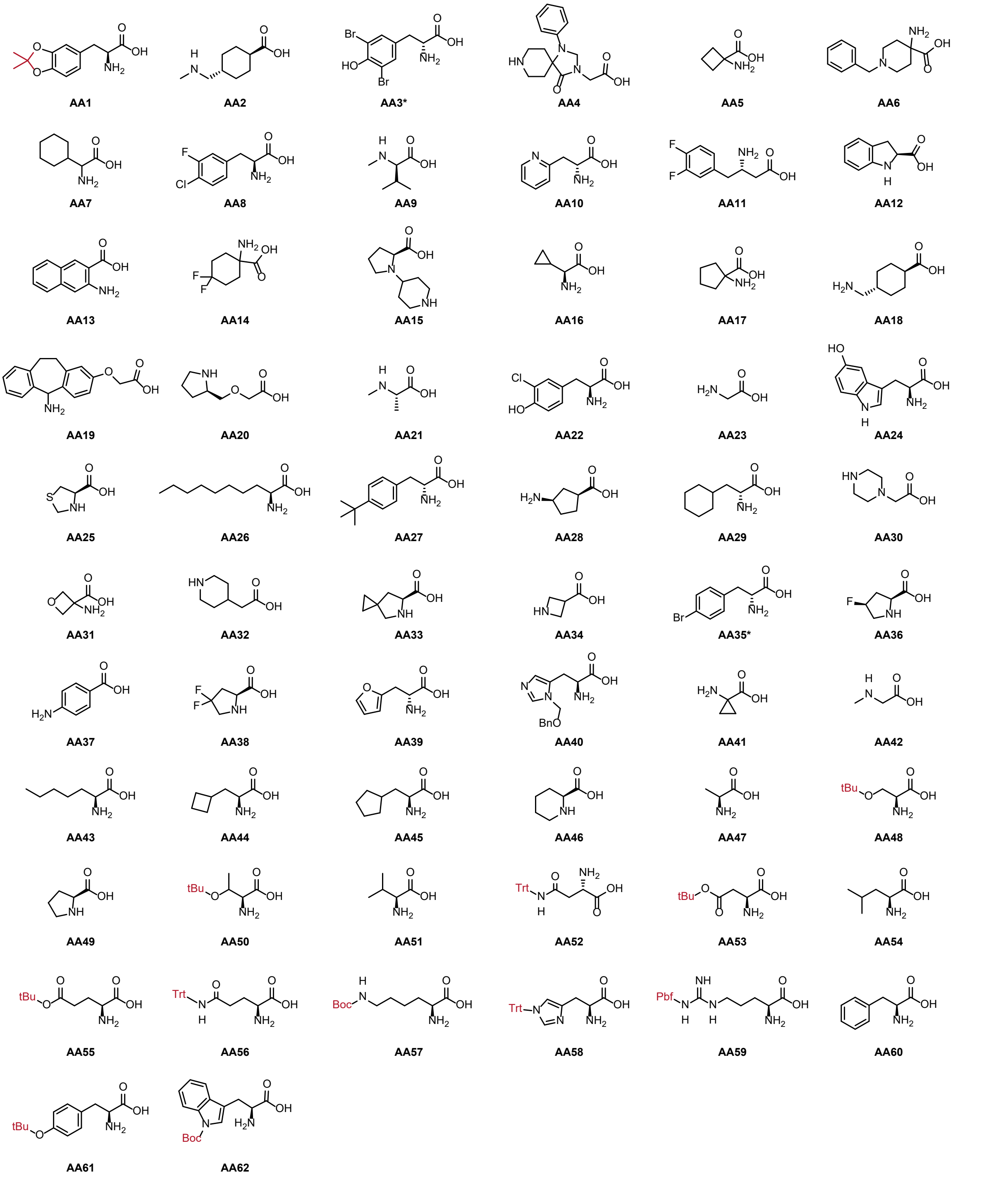

Supplementary Fig 8. Amino acid building blocks used to synthesize SEL 1, 2 and 3

Fmoc protected variants of the amino acids were used in the library synthesis. Building blocks marked with a * are not compatible with SEL 3 and were excluded for that design. Protecting groups indicated in red are removed during cleavage.

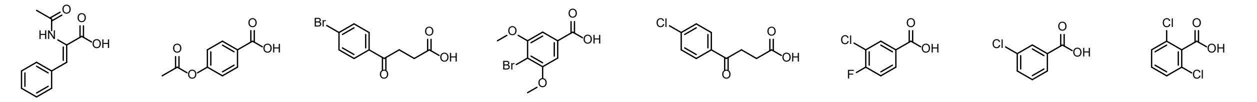

CA1 CA2 CA3 CA4 CA5 CA6 CA7 CA8

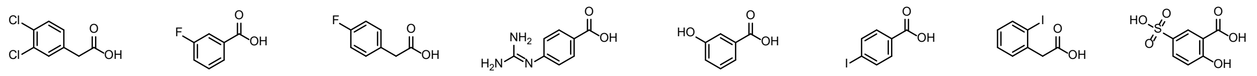

CA9 CA10 CA11 CA12 CA13 CA14 CA15 CA16

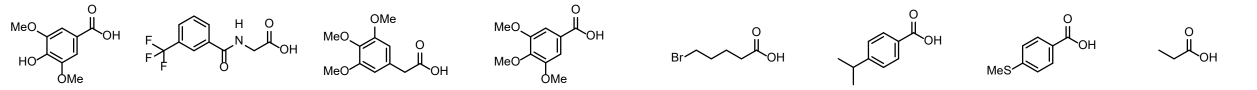

CA17 CA18 CA19 CA20 CA21 CA22 CA23 CA24

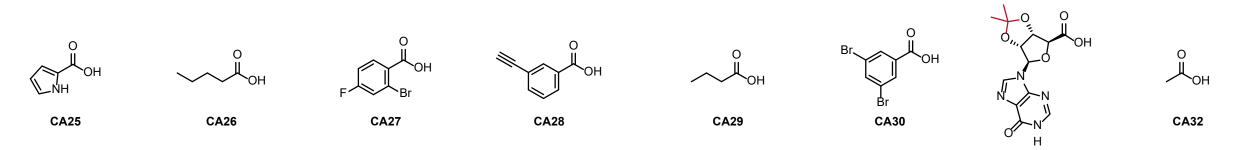

CA25 CA26 CA27 CA28 CA29 CA30 CA31 CA32

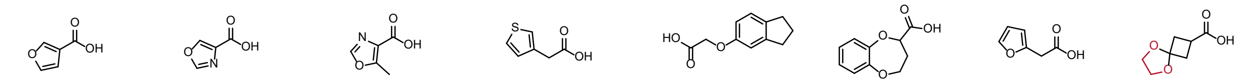

CA33 CA34 CA35 CA36 CA37 CA38 CA39 CA40

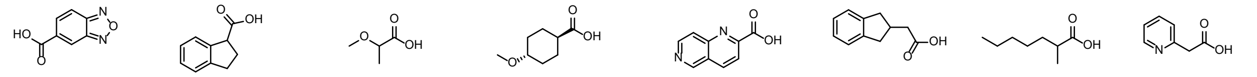

CA41 CA42 CA43 CA44 CA45 CA46 CA47 CA48

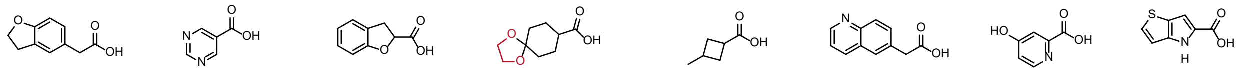

CA49 CA50 CA51 CA52 CA53 CA54 CA55 CA56

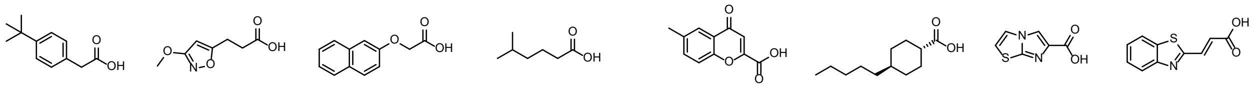

CA57 CA58 CA59 CA60 CA61 CA62 CA63 CA64

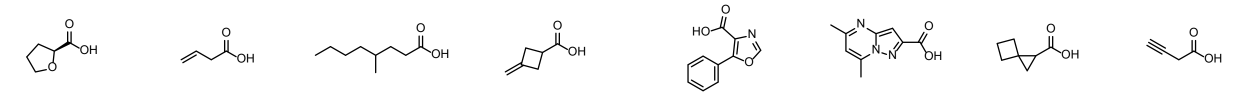

CA65 CA66 CA67 CA68 CA69 CA70 CA71 CA72

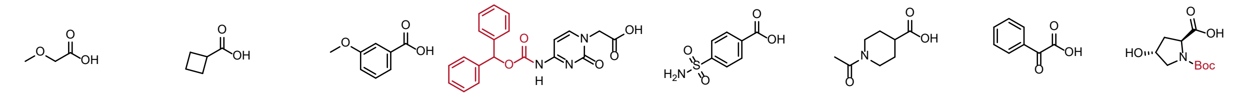

CA73 CA74 CA75 CA76 CA77 CA78 CA79 CA80

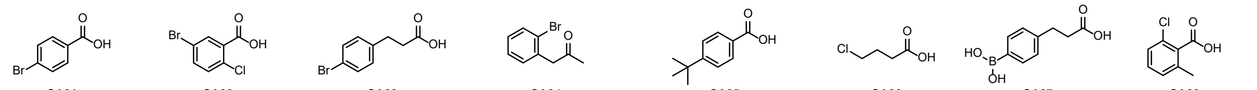

CA81 CA82 CA83 CA84 CA85 CA86 CA87 CA88

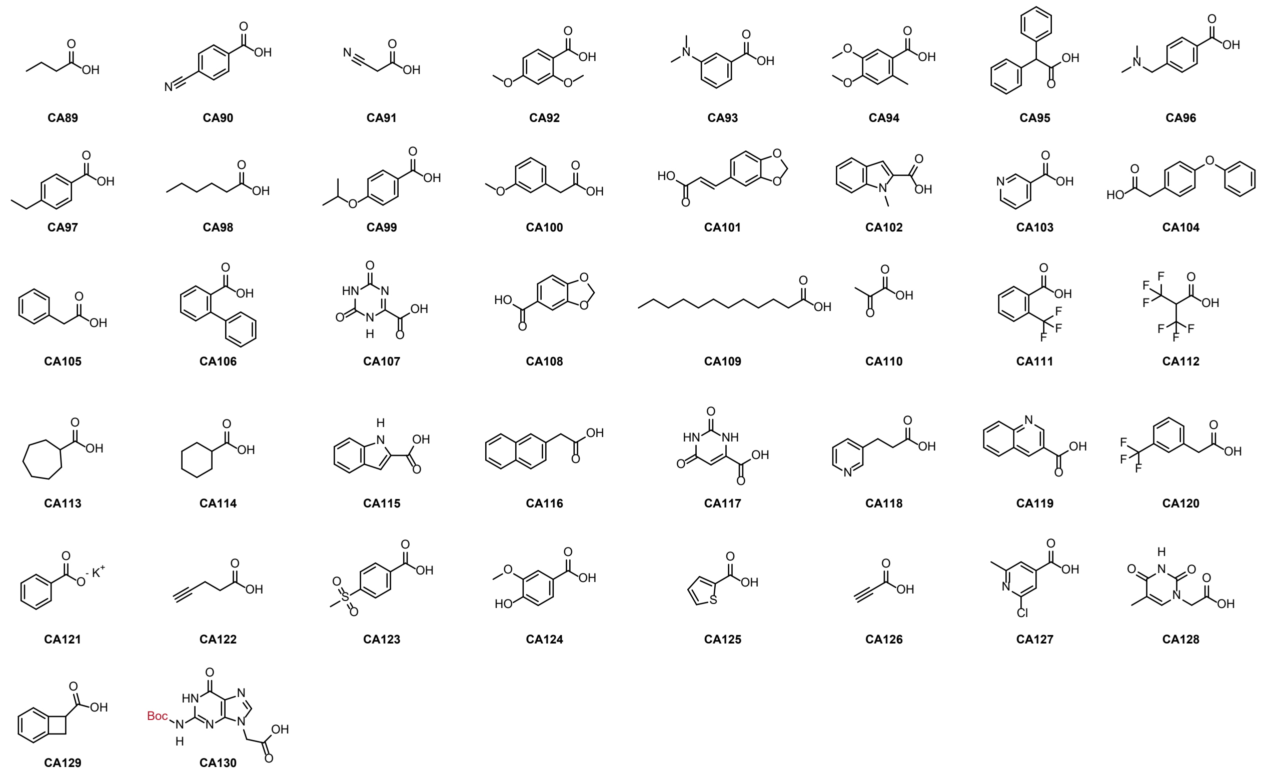

Supplementary Fig 9. Carboxylic acid building blocks used to synthesize SEL 1

Protecting groups indicated in red are removed during cleavage.

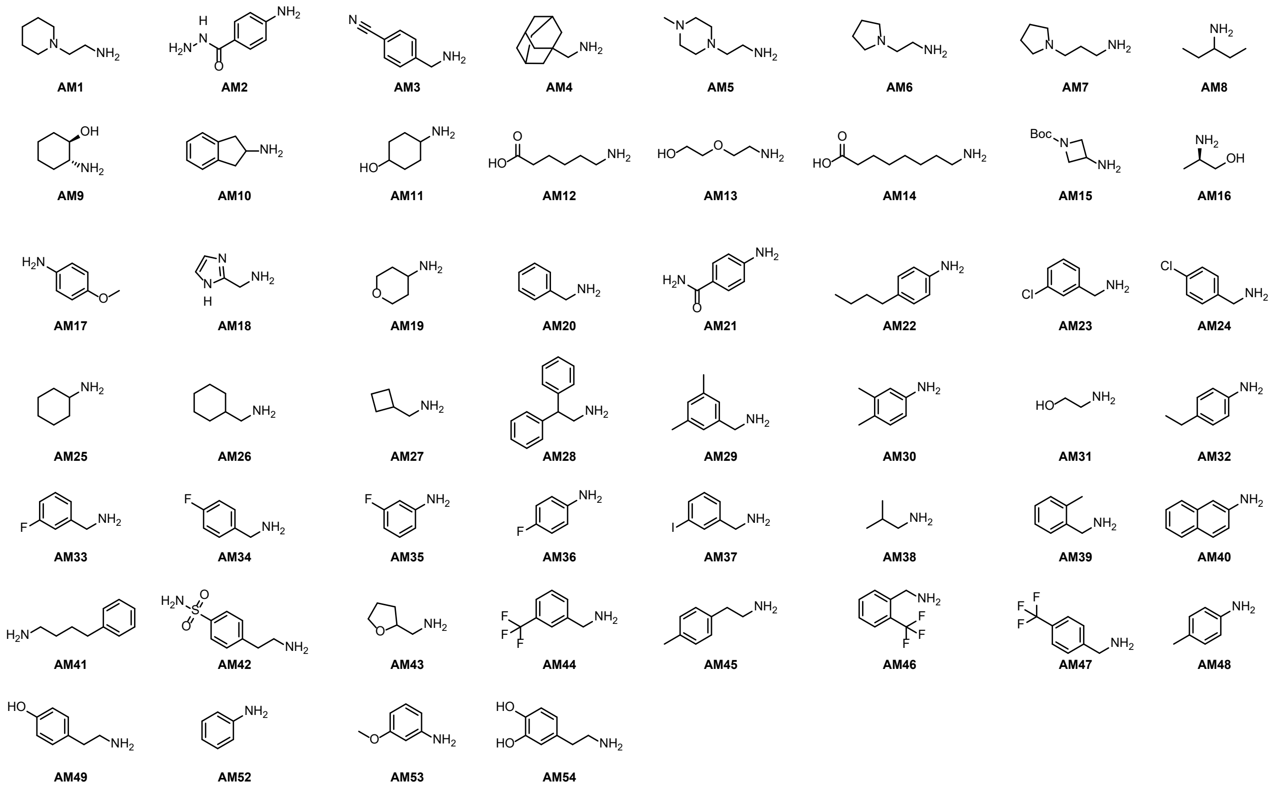

Supplementary Fig 10. Primary amine building blocks used to synthesize SEL 2

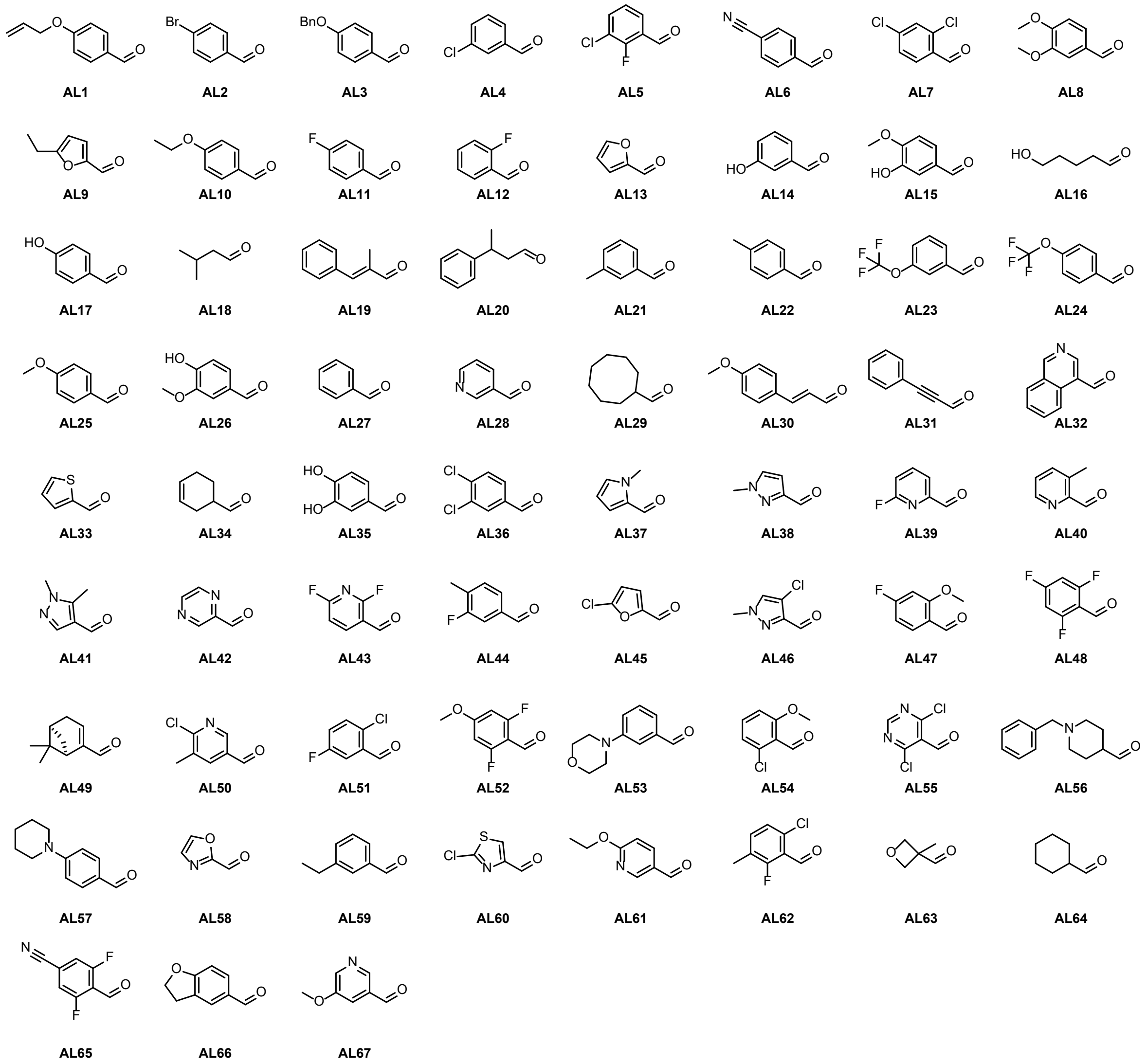

Supplementary Fig 11. Aldehyde building blocks used to synthesize SEL 2

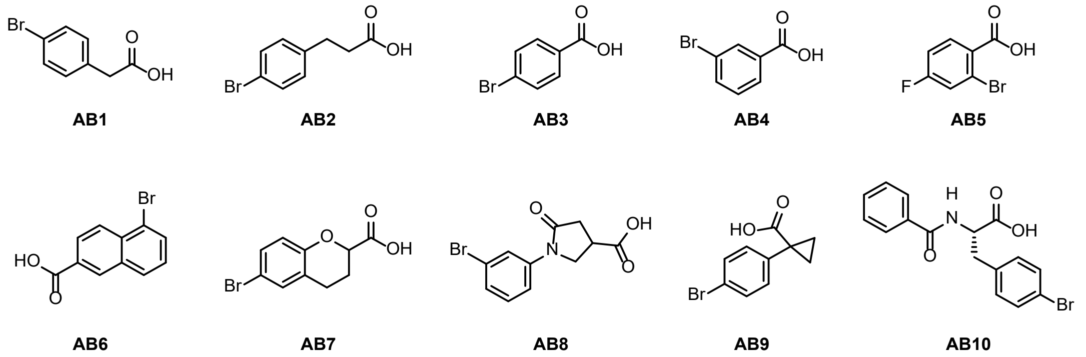

Supplementary Fig 12. Aryl bromide building blocks used to synthesize SEL 3

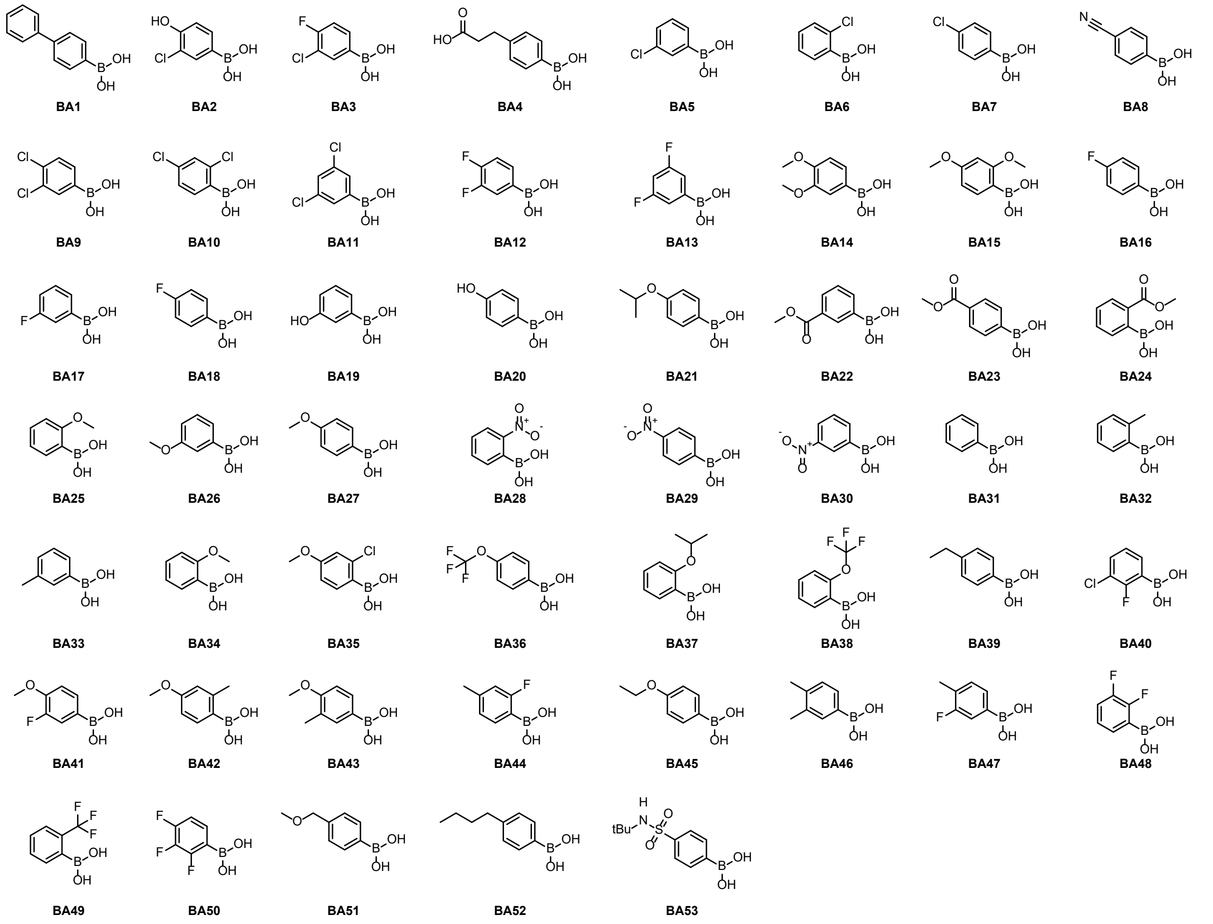

Supplementary Fig 13. Boronic acid building blocks used to synthesize SEL 3

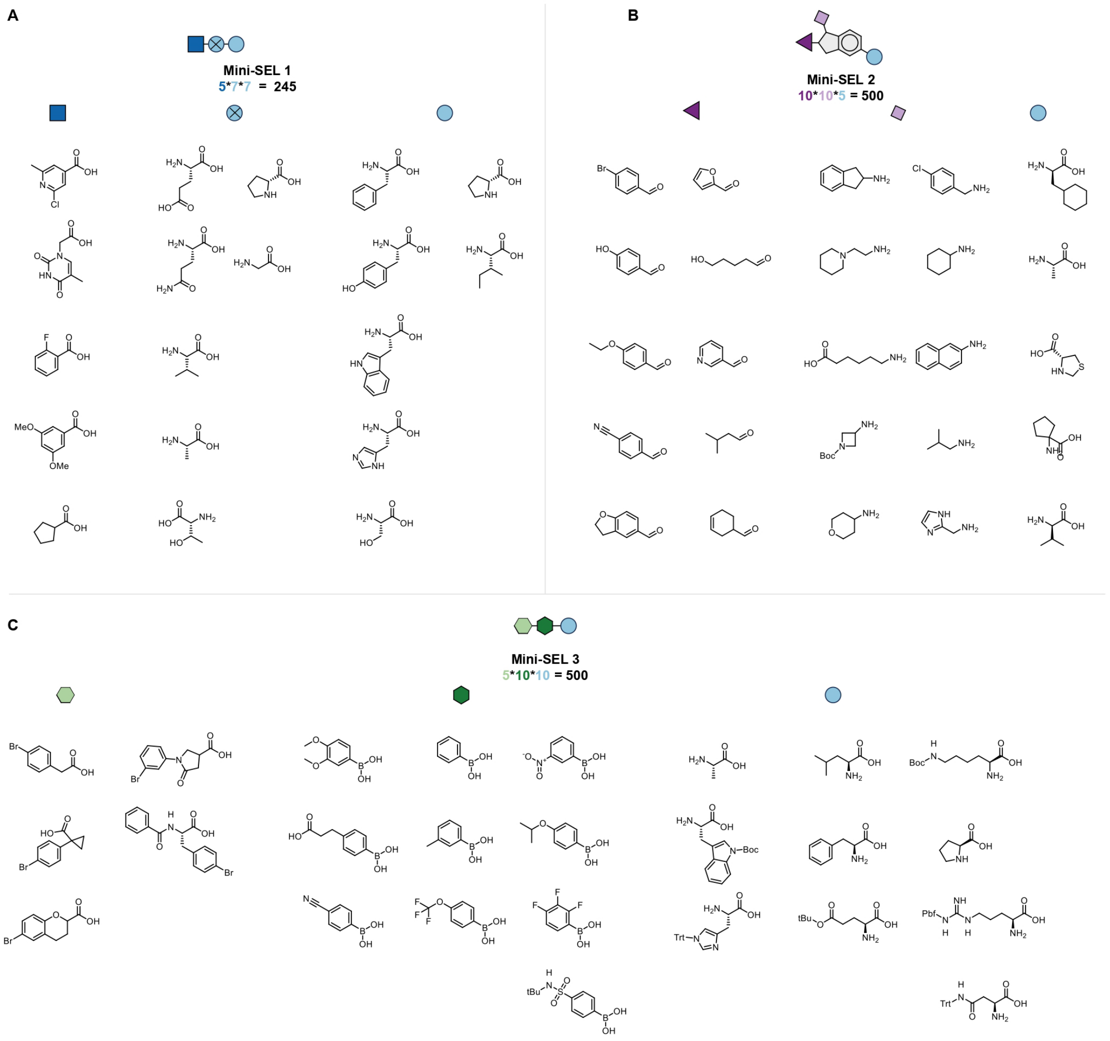

Supplementary Fig 14. Building blocks used for Mini-SEL 1-3

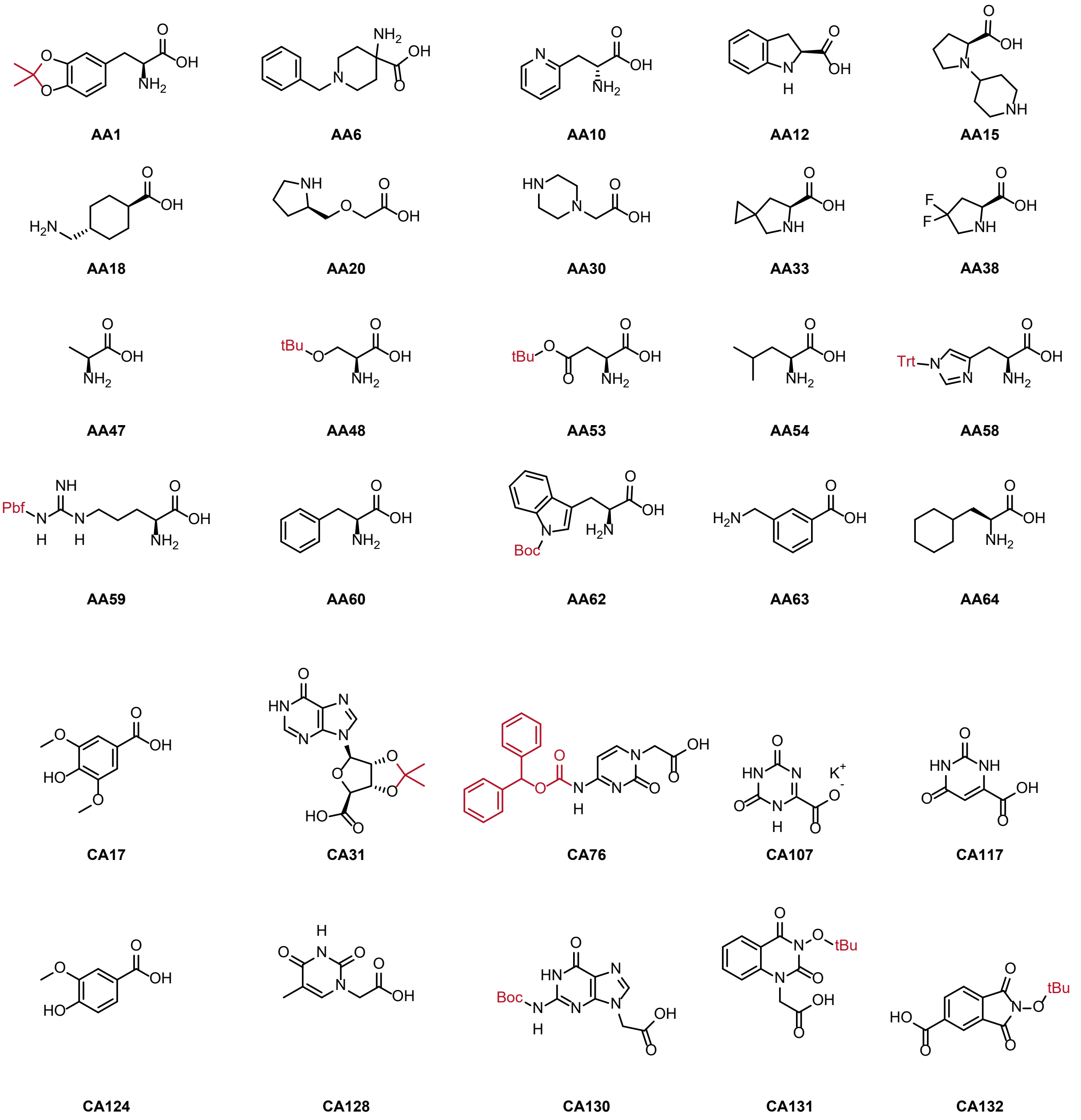

Supplementary Fig 15. Amino acid and carboxylic acid building blocks used to synthesize SEL 4

Supplementary Table 1. The impact of the number of matching fragments on the AS-MS selection against CAIX.

	Matching 1 fragment [hits/total annotated]	Matching 2 fragments [hits/total annotated]	Matching 3 fragments [hits/total annotated]
SEL 1	75/228	72/124	19/29
SEL 2	30/75	1/4	0/0
SEL 3	47/51	14/14	0/0

Supplementary Table 2. Selection against CAIX with a decreasing library concentration.

[Library member]	Total compounds identified	Compound containing CA77	True positive ratio
1 pmol	407	41	10%
100 fmol	188	31	16%
10 fmol	114	0	0%

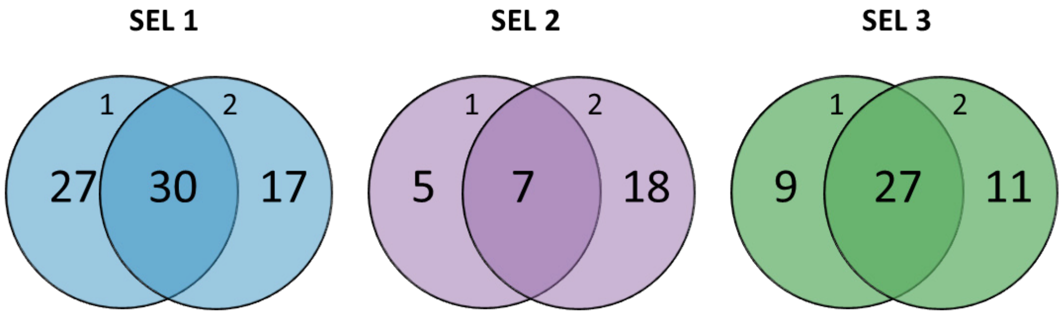

Supplementary Fig 16. Venn diagrams illustrating the overlap of annotated hits identified in the two duplicate affinity selections targeting CAIX for SEL 1-3.

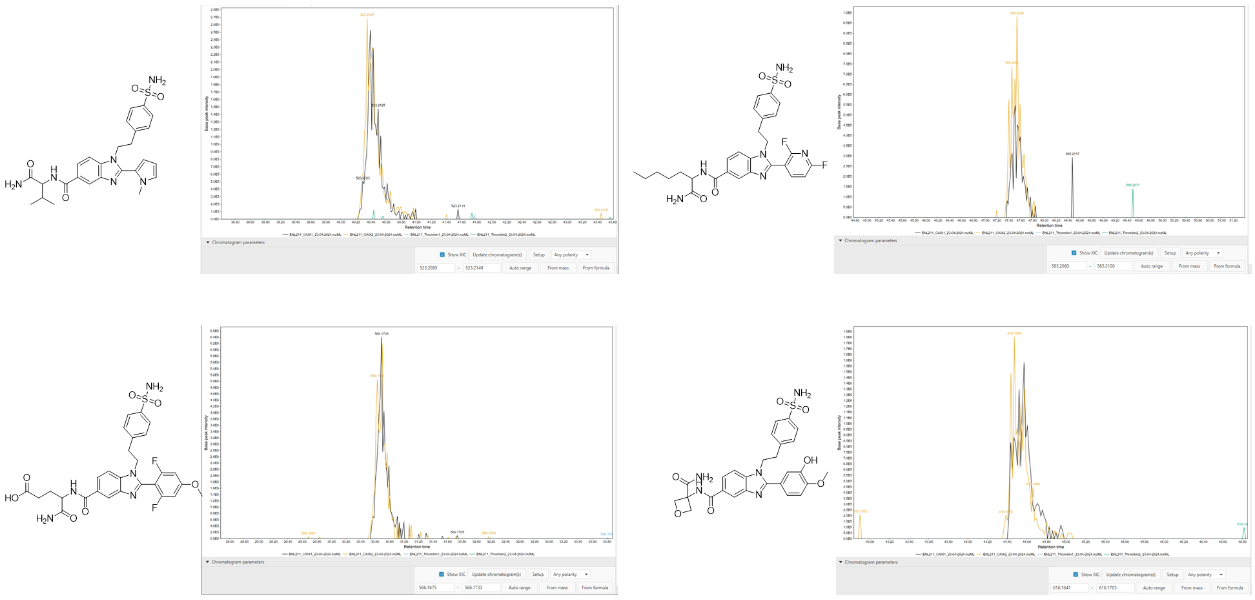

Supplementary Fig 17. Examples of SEL 2 hits targeting CAIX that display an enrichment in MS1 intensity but were only annotated in 1 replicate

a)

**SEL1**	**# sulfonamide**	**# non sulfonamide**	**Total**
After AS-MS	74	154	228
Before AS-MS	3770	495722	499492
Total	3844	495876	499720
p = 8.362941e-97			

**SEL2**	**# sulfonamide**	**# non sulfonamide**	**Total**
After AS-MS	30	45	75
Before AS-MS	4124	211809	215933
Total	4154	211854	216008
p = 1.00912e-31			

**SEL3**	**# sulfonamide**	**# non sulfonamide**	**Total**
After AS-MS	47	4	51
Before AS-MS	553	31196	31749
Total	600	31200	31800
p = 3.45304e-77			

b)

```
####### SEL1 #######
library_size <- 62 * 62 * 130
strcts_w_substrct <- 62 * 62
compounds_after_selection <- 228
found_binders <- 74


structs_wo_substrct <- library_size – strcts_w_substrct
nonHits_w_substrct <- strcts_w_substrct - found_binders
hits_wo_substrct <- compounds_after_selection - found_binders
nonHits_wo_subsrct <- library_size - compounds_after_selection - nonHits_w_substrct


data <- data.frame(
"sulfonamide containing structures" = c(found_binders, nonHits_w_substrct),
"structures without sulfonamide" = c(hits_wo_substrct, nonHits_wo_subsrct),
row.names=c("After AS-MS", " Before AS-MS")
)
fisher.test(data)$p.value
```

Supplementary Fig 18. Statistical significance calculation using Fisher's exact test a) Contingency tables showing the numerical data used for Fisher's exact test. **b)** Representative calculation of the p-value for SEL1 using Fisher's exact test in RStudio.

2. Abbreviations

BB	Building block
BLI	Biolayer interferometry
Boc	*tert*-butoxycarbonyl
CAIX	Carbonic anhydrase IX
CDI	1,1′-carbonyldiimidazole
DAG	Directed acyclic graph
DCM	Dichloromethane
DEL	DNA-encoded library
DIPEA	Di-isopropylethylamine
DMF	Dimethylformamide
DMSO	Dimethylsulfoxide
Et	Ethyl
Eq	Equivalent
FA	Formic acid
FBS	Fetal bovine serum
FEN1	Flap endonuclease 1
Fmoc	Fluorenylmethoxycarbonyl
HATU	Hexafluorophosphate Azabenzotriazole Tetramethyl Uronium
HBA	Hydrogen bond acceptor
HBD	Hydrogen bond donor
HEPES	(4-(2-hydroxyethyl)-1-piperazineethanesulfonic acid)
iPr	Isopropyl
Me	Methyl
MeCN	Acetonitrile
MeOH	Methanol
MW	Molecular weight
MQ	MilliQ
Pbf	2,2,4,6,7-Pentamethyl-2,3-dihydrobenzofuran-5-sulfonyl
PBS	Phosphate-buffered saline
PEL	Peptide-encoded library
Ppm	Parts per million
r.t.	Room temperature
SA	Streptavidin
SEL	Self-encoded library
t-Bu	*tert*-Butyl
TFA	Trifluoroacetic acid
TIPS	Triisopropylsilane
TPSA	Topological polar surface area
Trt	Trityl
Tween-20	Polysorbate 20

3. Material and methods

3.1. Reagents and supplies

Chemicals: Reagents and solvents were purchased from Sigma-Aldrich (Merck), Fisher Scientific, BLDpharm, Fluorochem or VWR and were used without further purification unless stated otherwise. Carboxylic acids, aldehydes and Fmoc-protected amino acids building blocks were partially purchased from Chemspace. Tentagel® S NH2 90 µm (S30902) and TentaGel® M NH_2 30 µm (M30352) were purchased from Rapp-Polymere.

Materials for affinity selection: Dynabeads MyOne Streptavidin T1 and Dynabeads His-Tag Isolation and Pulldown were purchased from ThermoFisher Scientific. A KingFisher™ Duo Prime Purification System was used to perform our affinity selection experiments. The protocols were developed with BindIt 4.1 Software.

Proteins: Biotinylated Human Carbonic Anhydrase IX (38-414), His,Avitag (CA9-H82E3) and Human Carbonic Anhydrase IX (38-414), His,Avitag (CA9-H5226) were purchased from ACROBiosystems. FEN1 was obtained from Sylvie Noordermeer (Department of Human Genetics, Leiden University Medical Center).

3.2. Instrumentation

LC-MS analysis: Compound purity was determined by LC–MS, using the LCMS-2020 system (Shimadzu) with a Gemini 3 µm C18 110 Å column (50 × 3 mm) using the following parameters: flow rate = 0.55 mL/min, scan range = 160-800 m/z, column temperature (°C) = 40. The following gradient of 10−90% $MeCN/H_2O$ (0.1% formic acid) over 15 min and measuring UV absorbance at 254 nm was used, unless stated otherwise. Compounds were dissolved in H_2O:MeCN:*t*-BuOH (1:1:1) before injection.

LC-MS/MS analysis: Analysis was performed on an Vanquish™ Neo UHPLC system (Thermo Scientific) connected to an Orbitrap Exploris 240 mass spectrometer (Thermo Fisher Scientific). Samples were run on a Double nanoViper PepMap Neo column (2 µm particle size, 15 cm x 75 µm, Thermo Fisher Scientific, DNV75150PN) following a PepMap Neo Trap Cartridge (5 µm C18 300 µm X 5 mm). The standard nano-LC method was run without temperature control and a flow rate of 300 nL/min with the following gradient: 0% solvent B ramping linearly to 60% B in A over 78 min, with solvent A = water (0.1% FA), and solvent B = 80% acetonitrile, 20% water (0.1% FA). Positive spray ionization was set at 1900 V. The following parameters were used for MS1 collection: resolution = 120.000, scan range = 275-650 m/z for SEL 1 and SEL 4, 300-800 m/z for SEL 2 and 275-700 for SEL 3, maximum injection times = 300 ms, RF Lens = 80%, microscans = 1, AGC target = standard, Ion Transfer Tube Temp (°C) = 280, Charge State = +1. For the data dependent MS/MS event the following settings were used: resolution = 15000, number of Dependent Scans= 20, isolation window (m/z) = 1.5, intensity threshold = 1E5, MIPS mode = small molecule, absolute collision energy = 15, 25 eV, AGC Target (%) = 50, microscans = 1, RF Lens(%) = 70, dynamic exclusion = on (exclude after n times = 3, Exclusion duration (s) = 15, excluding isotopes, 10 ppm mass tolerance).

NMR: ^{1}H and ^{13}C NMR spectra were recorded on a Bruker AV-400 (400 MHz) spectrometer. Chemical shift values are reported in parts per million (ppm) and designated by δ. Tetramethylsilane or solvent resonance was used as internal standard. Coupling constants (J) are reported in Hertz (Hz) and multiplicities are indicated by s (singlet), bs (broad singlet), d (doublet), t (triplet), td (triplet of doublets), p (pentuplet), h (hexuplet) or m (multiplet).

Purification: The automatic flash chromatography was performed on a Biotage Selekt System with pre-packed flash cartridges (Biotage® Sfär Bio C18 - Duo 300 Å 20 µm). MQ 0.1% TFA (buffer A) and MeCN 0.1% TFA (buffer B) were used as mobile phase

3.3. General procedures

Manual solid-phase synthesis (SPS): TentaGel S NH_2 resin (90 µm, 0.26 mmol/g, 1.0 eq.) was functionalized with a Rink linker using the following protocol before attaching other building blocks.

TentaGel S NH_2 resin (90 µm, 0.26 mmol/g, 1.0 eq.) was loaded onto a fritted syringe and swelled for 5 min in DMF. A solution of Fmoc-protected amino-acid (3.0 eq), HATU (0.4 M, 2.98 eq) and DIPEA (9.0 eq) in DMF was added to the resin. After 20 min the resin was washed with DMF (5x) and Fmoc deprotection was performed by washing with piperidine (20% in DMF, 1x) before incubating with piperidine (20% in DMF) for 10 min. The resin was washed with DMF (5x) before adding the next Fmoc-protected amino-acid. The protocol was repeated until completion of the synthesis.

The compounds were cleaved of the resin by incubating at room temperature for 1 hour with a solution of TFA:H_2O:TIPS (92.5:5:2.5) and washed with TFA:H_2O:TIPS (92.5:5:2.5). The volume was reduced by evaporating the TFA solution with a N_2 stream. The reaction mixture was dissolved in H_2O:MeCN:*t*-BuOH (1:1:1) before injection into a LC-MS.

Protein biotinylation: EZ-Link™ Sulfo-NHS-LC-Biotin was used in accordance with the user guide from ThermoFisher Scientific.

Affinity selection: A KingFisher™ Duo Prime Purification System was used to perform our affinity selection experiments. The protocols were developed with BindIt 4.1 Software. Affinity selection experiments against CAIX were performed in duplicates with protein (150 pmol) immobilized on Dynabeads MyOne Streptavidin T1 (1 mg) and library (100 fmol/member) with the King Fisher protocol as described in Supplementary Fig.19 unless stated otherwise. Affinity selection experiments against FEN1 were performed in duplicates with protein (150 pmol) immobilized on Dynabeads™ His-Tag Isolation and Pulldown (1 mg) and library (1 pmol/member) with the King Fisher protocol as described in Supplementary Fig.19.

Biolayer interferometry (BLI): Purified biotinylated compounds were dissolved to 1 µM in 1x PBS, 0.02% Tween-20, 1 mg/ml BSA (0.1% (w/v)) (kinetic buffer) used for immobilization onto streptavidin Octet SA Biosensors (SATORIUS). Biolayer interferometry (BLI) assays were performed in 96 well plates (GreinerBio-One, polypropylene, flat-bottom, chimney well) using an Octet R4 system (SATORIUS). Wells were filled with 200 µL with kinetic buffer, compound solution or CAIX solution.

Biotinylated compound was immobilized onto the streptavidin biosensor for 60 s. Sensors were then dipped into kinetic buffer for 60 s, CAIX solution (500 nM, 250 nM, 125 nM, 62.5 nM) for 600 s and into kinetic buffer for 600s. Measurements were carried out at 30 °C.

A

	General		Beginning of step		Mixing/Heating		End of step		
	Buffer	Volume (µL)	Release time (s)	Release speed	Mixing time (min)	Temp	Mixing speed	Collect count	Collect time (s)
Bead uptake		100	-	-	5	r.t.	Bottom mix	5	10
Bead washing (3x)	A	1000	30	Medium	3	r.t.	Medium	5	10
Protein incubation	B	100	30	Medium	60	10 °C	Medium	5	10
Biotin blocking (2x)	C	1000	30	Medium	10	r.t.	Medium	5	10
Bead washing	A	1000	30	Medium	3	r.t.	Medium	5	10
Library Incubation	E	1000	30	Medium	60	10 °C	Medium	5	10
Bead washing (5x)	F	1000	30	Medium	0.5	r.t.	Medium	5	10
Elution (2x)	G	100	30	Medium	3	r.t.	Medium	5	10

B

Buffer	CAIX	FEN1
A	10% FBS, 1x PBS, 0.02% Tween-20	50 mM HEPES, 100 mM KCl, 5 mM $MgCl_2$, 1 mM DTT, 10% FBS, 0.02% Tween-20, pH = 7.5
B	CAIX (1.5 µM) in **A**	FEN1 (1.5 µM) in **A**
C	10% FBS, 1x PBS, 0.02% Tween-20, 400 µM d-biotin	50 mM HEPES, 100 mM KCl, 5 mM $MgCl_2$, 1 mM DTT, 10% FBS, 0.02% Tween-20, 20 mM imidazole pH = 7.5
D	10% FBS, 1x PBS	50 mM HEPES, 100 mM KCl, 5 mM $MgCl_2$, 1 mM DTT, 10% FBS, pH = 7.5
E	100 fmol/member library in buffer **D**	100 fmol/member library in buffer **D** + 20 mM imidazole
F	1x PBS	50 mM HEPES, 100 mM KCl, 5 mM $MgCl_2$, 1 mM DTT, pH = 7.5
G	MeCN:MQ (1:1) 0.1%FA	MeCN:MQ (1:1) 0.1%FA

Supplementary Fig 19. Overview of the affinity selection procedure.
A) The KingFisher program used for affinity selection. B) Buffer conditions for CAIX and FEN1

Sample preparation after AS: Samples from the affinity selection procedure were lyophilized and resuspended in 50 µL MQ 0.1% FA. The StageTips were prepared as described by Rappsilber et al. using C18 material from Empore SPE 47 mm discs (66883-U, Merck)[1]. The StageTips were pre-conditioned with 200 µL MeOH, 200 µL of 0.1% (v/v) FA in MeCN and 200 µL of 0.1% (v/v) FA in MQ, respectively by centrifuging for 3 min at 300 x g. The samples were then loaded on the StageTips and washed with 200 µL of 0.1% (v/v) FA in MQ. Compounds were eluted by adding 200 µL of 0.1% (v/v) FA in MeCN:MQ (7:3) and subsequently lyophilized before resuspending in 10 µL 0.1% (v/v) FA in UPLC-MS grade water. The samples were centrifuged for 5 min at 21,000 x g. Afterwards, 9 µL was transferred to a LC-MS vial and 8 µL was injected into the LC-MS/MS system.

3.4. Software

KNIME 5.1.2 software was used for library enumeration, molecular property calculations and the generation of the enriched building block plots in Fig. 4A. The following extensions were installed RDkit Nodes version 4.7., CDK version 1.5.6 and ChemAxon version 4.7.0. The KNIME workflow is available through Zenodo (https://doi.org/10.5281/zenodo.14070388).

Proteowizard (MSconvert)[2]: Raw files (.raw) from the Orbitrap Exploris 240 mass spectrometer were converted to .mzML files using MSConvert (version: 3.0.25106-8b1114c) selecting Peak Picking>MS levels 1-2. Resulting .mzML files were analyzed by using COMET.

4. COMET

COMET software version 1.0.0-SNAPSHOT is available through GitHub (https://github.com/sirius-ms/comet). Enumerated libraries generated by the above KNIME workflow were imported into COMET as custom structure databases using the GUI. Spectra files were imported as .mzML files and background subtraction was performed using the "Tags" panel in the general filter dialog window of the GUI using the following settings; MS1 m/z accuracy = 5 ppm, RT accuracy = 10 sec and max intensity ratio = 3 (see Supplementary Fig 20). Features from actual samples that also appeared in the control runs within a ten second retention time tolerance, a five ppm mass deviation tolerance, and a fold change of less than three were removed. The COMET filter can be accessed through the same filter dialog window, where a separate section for COMET is provided (Fig. S17). For its application, we used the following settings: scaffold formula: C8H3N2O for SEL 2 and left blank for SEL 1, 3 and 4, MS1 mass accuracy (ppm) = 5 ppm, considered fragment types = SEL 1: S[0;1],S[1;2],0,2, SEL 2: S[0;2],S[1;2],0,1, SEL 3: S[1:2],0, SEL 4: S[0;1],S[1;2],0,2 minimum number of matching peaks = 1, number of considered peaks = 5, number of allowed hydrogen shifts = 1, MS2 mass accuracy (ppm) = 5. The specification of such fragment types is illustrated in Supplementary Fig 22. For each library, a file containing information about all the library's building blocks has to be provided in COMET. These .csv files were generated using the python script "COMET_Building blocks_input.ipynb" which is available through Zenodo (https://doi.org/10.5281/zenodo.14070388). See section 4.1 for method details on the COMET filters. Molecular formula generation in COMET was performed using formula database search in the imported custom library, all other settings were left to default. After fingerprint prediction (score threshold enabled), the imported custom library was used for structure database search. Structure candidates were ranked according to EPIMETHEUS, see section 4.5. for method details.

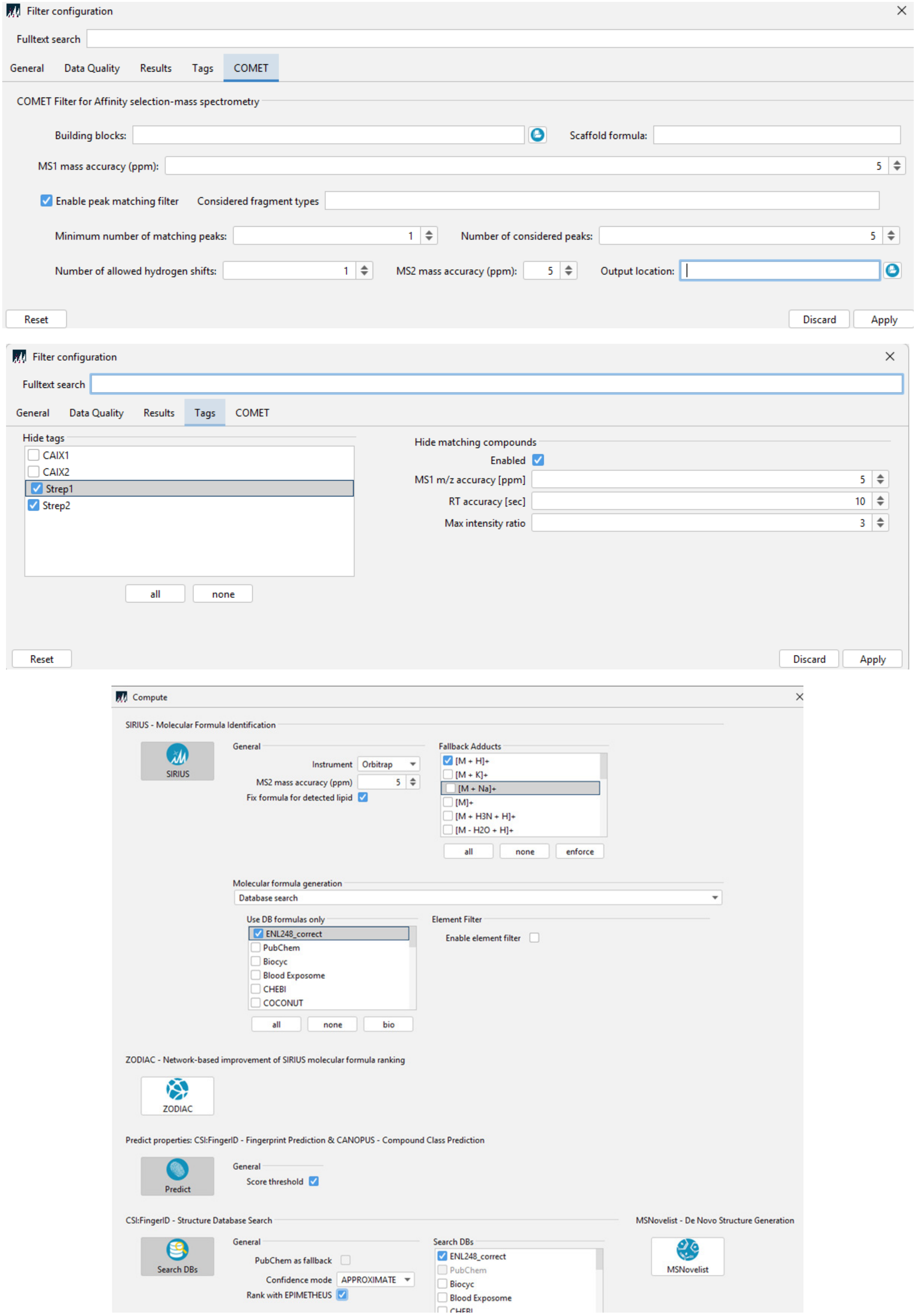

Supplementary Fig 20. Overview of parameters used for COMET and CSI:FingerID annotation

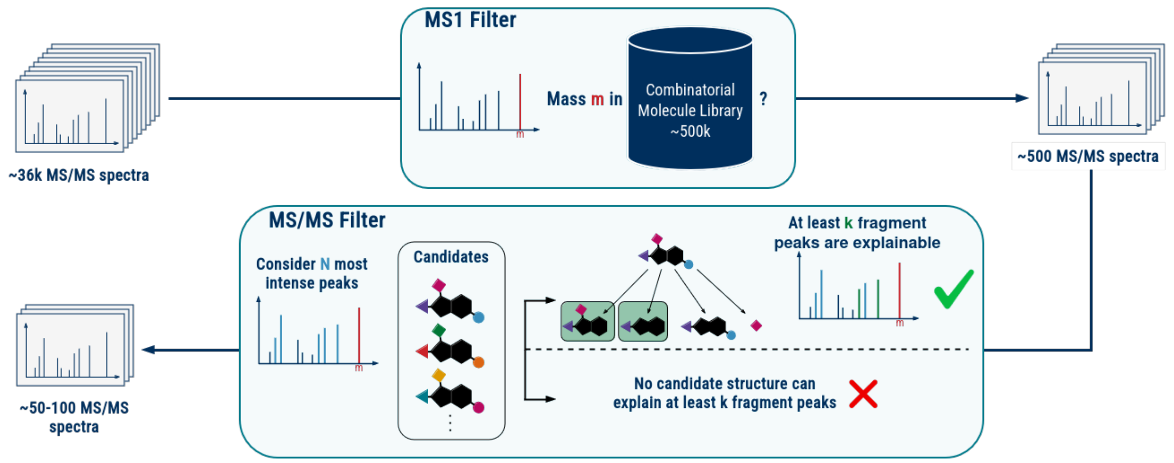

Supplementary Fig 21. Overview of the COMET workflow.
COMET consists of two filtering steps for removing MS/MS spectra unrelated to the library compounds. The first step selects only those MS/MS spectra for which at least one candidate structure in the library exists. The second step determines for each of the remaining MS/MS spectra whether there is a candidate structure whose fragments can explain at least k of the N most intense fragment peaks. Here, only those fragments are considered that either result from the cleavage of building blocks or represent individual building blocks. If such a candidate structure doesn't exist, the corresponding spectrum is excluded.

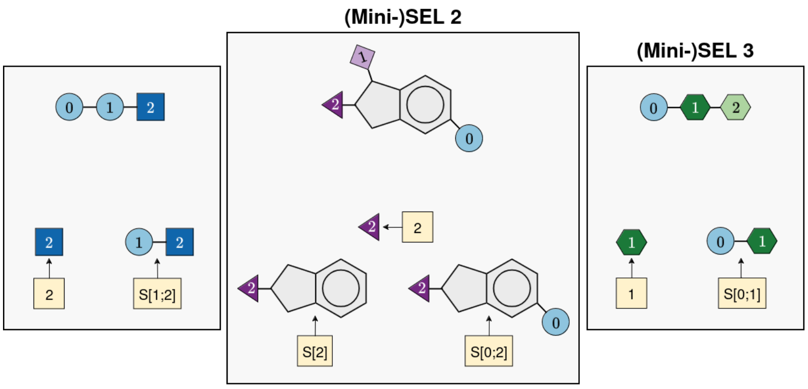

Supplementary Fig 22. Examples of fragment types together with their specification in COMET.

4.1. Feature Filtering

An affinity selection experiment results in a final sample containing the library molecules with high affinity to the target receptor. A crucial step is the identification of these isolated hit compounds. This is accomplished by analyzing this sample via LC-MS/MS and annotating the obtained MS/MS spectra with potential hits of the library. Because most of the obtained spectra correspond to background noise, we developed COMET (COmbinatorial Mass Encoding decoding Tool). COMET excludes those MS/MS spectra which are unrelated to a library molecule, either due to a non-matching precursor mass or a non-matching fragmentation pattern. Therefore, COMET aims to improve the structure annotation by decreasing the proportion of false positives in the final set of proposed compounds.

COMET consists of two consecutive filtering steps for removing MS/MS spectra that do not match to a library compound (Supplementary Fig 21). The first filter considers only the precursor peak of each MS/MS spectrum and discards all those spectra whose precursor mass doesn't match any compound in the library (i.e. all spectra for which no library candidate exists). The second filtering step is based on our observation that some of the covalent bonds connecting the building blocks are fragmented more frequently during CID. This leads to fragment peaks in the MS/MS spectra which correspond to single building blocks or fragments where at least one building block was cleaved off (Fig. 3d). Therefore, this filter retains those of the remaining MS/MS spectra that contain a minimum number of these fragment peaks which should be explainable by one of the candidate structures. Here, only the N most intense fragment peaks (excluding the precursor peak) of each spectrum are considered, of which at least k peaks should be explainable.

To check if a spectrum has at least k of these fragment peaks, each candidate structure is first fragmented into a predefined set of fragments by cleaving only those bonds that are incident to a building block. For each fragment peak in the spectrum, it is then assessed if there is a fragment of the current candidate structure whose mass matches the neutral mass of the peak regarding a specified mass deviation. If such fragment exists, the fragment peak is seen as "explained" and the assessment continues with the next fragment peak, if present. Because of the possibility that hydrogen rearrangements occur during the CID, a maximum of Δ hydrogen shifts of the fragment's mass are allowed. If none of the spectrum's candidate structures can explain at least k peaks, the spectrum will be removed.

For each split-and-pool library, fragmentation rules defining this kind of fragmentation can be derived and specified in COMET. If such information isn't known beforehand and thus not provided, all those fragments of a candidate structure will be generated by combinatorially breaking only the bonds connecting the building blocks.

4.2. Fragmentation pattern analysis of Mini-SELs 1-3

We synthesized defined subsets of 245 to 500 compounds for each self-encoded library and measured the resulting Mini-SELs 1-3 via nanoLC-MS/MS (Fig. 3b). Based on the obtained LC-MS/MS data, we assessed the capability of SIRIUS 6 for detecting the actual measured compounds when searching in the corresponding larger library (Fig. 3d). Furthermore, we used the LC-MS/MS data to analyze how these molecules fragment during the collision-induced dissociation (CID) and to derive certain fragmentation rules for each library design (Fig. 3d). To do this, we first annotated the measured MS/MS spectra with the corresponding library compounds, resulting in three datasets of annotated MS/MS spectra.

4.3. Annotation Procedure

The measured LC-MS/MS data of each test library was imported into SIRIUS 6. Here, the wavelet filter with a coefficient of 32 was used for smoothing, and SIRIUS detected for Mini-SEL 1 3248, for Mini-SEL 2 3682, and for Mini-SEL 3 1629 features having at least one MS/MS spectrum and being classified as "decent" or "good" by its quality filter. Of these features, precisely those that do not match a library compound in terms of their precursor mass and a mass deviation of 10 ppm were removed. This resulted for Mini-SEL 1 in 301, for Mini-SEL 2 in 725, and for Mini-SEL 3 in 400 features. For each of these remaining features, molecular formula candidates were retrieved from the corresponding custom database and fragmentation trees were computed. A filtering of those features where an associated fragmentation tree explains at least three peaks in the MS/MS spectrum was applied resulting in the final set of 254 features for Mini-SEL 1, 690 for Mini-SEL 2, and 368 for Mini-SEL 3. These features and mainly their MS/MS spectra were considered for the structure annotation.

For Mini-SEL 2, 23 features were manually annotated with compounds of the corresponding library. The other 667 features were automatically annotated by ranking their candidate structures according to EPIMETHEUS (see section 4.5.) and using the top ranked structure for annotation. This automatic annotation workflow was also applied for all remaining features belonging to Mini-SEL 1 and Mini-SEL 3. Because this procedure may create conflicts by annotating several features with the same compound or with an already manually assigned compound, these features were removed. The result are three datasets consisting of 119 annotated features for Mini-SEL 1, 232 for Mini-SEL 2, and 194 for Mini-SEL 3. The MS and MS/MS spectra of these features together with their structure annotations were extracted and stored as .ms files. The .ms files are available through Zenodo. (https://doi.org/10.5281/zenodo.14070388).

4.4. Fragmentation Analysis

By manually inspecting the collected LC-MS/MS data, we found that some MS/MS spectra contain fragment peaks corresponding to either single building blocks or fragments resulting from their cleavage. To investigate this further, we analyzed how often these fragment peaks can be observed in the MS/MS spectra by utilizing the annotated datasets described above.

Since the molecules of such combinatorially synthesized libraries share a consistent structure - specifically, the scaffold and connectivity of the building blocks -, those fragments created by the cleavage of building blocks can be grouped into several fragment types. For instance, the molecules of the SEL 1 library are characterized by the common abstract structure A-B-C, where A and B are amino acids, and C is a carboxylic acid. Here, A-B represents such a fragment type and defines for each molecule in the library the structure obtained by cleaving off the carboxylic acid (C).

For each test library, we iterated through the annotated MS/MS spectra and counted for each fragment type the number of spectra containing a corresponding fragment peak. Specifically, for each spectrum-molecule pair, we generated the corresponding fragment based on the molecule's structure and determined if the spectrum contains a matching fragment peak, allowing for a mass deviation of five ppm. Instead of considering only the primary fragment mass mF, we also accounted for possible hydrogen additions or losses during CID by checking if peaks were present at mF ± mH, where mH refers to the mass of a hydrogen atom. The results of this analysis are shown in Supplementary Table 3 and Fig. 3d.

Supplementary Table 3. Results of the fragmentation pattern analysis.

Based on the annotated MS/MS spectra of each test library, the fragmentation patterns of the molecules were analyzed. For each fragment type of a test library, the number of spectra containing a corresponding fragment peak was determined.

Library	Fragment types	Spectra with a matching fragment peak	Total number of annotated spectra
**Mini-SEL 1**	S[0;1]	10	119
	S[1;2]	100	
	0	92	
	1	8	
	2	89	
**Mini-SEL 2**	S[0]	0	232
	S[1]	0	
	S[0;1]	4	
	S[2]	170	
	S[0;2]	116	
	S[1;2]	203	
	0	30	
	1	79	
	2	0	
**Mini-SEL 3**	S[0;1]	1	194
	S[1;2]	105	
	0	81	
	1	2	
	2	7	

4.5. Ranking of candidate structures with EPIMETHEUS

Throughout this study, we used EPIMETHEUS to rank the candidate structures for a measured MS/MS spectrum. EPIMETHEUS is a combinatorial fragmenter, like FiD[3], MetFrag[4], EPIC[5] and MAGMa[6]. EPIMETHEUS explains the fragment peaks in the MS/MS spectrum with the most likely substructures of the candidate molecule. By doing that, EPIMETHEUS solves an optimization problem which means that an objective function is being optimized. Its value serves as a score which reflects how well the candidate structure can explain the peaks in the spectrum and with which the candidate structures can be ranked.

Similar to MetFrag[4],CFM-ID[7], and DEREPLICATOR+[8] each candidate structure undergoes an *in silico* fragmentation where all non-ring bonds (referred to as *bridges* in graph theory) and all pairs of bonds within each ring (known as *2-cuts* in graph theory) are cleaved combinatorially. This is an iterative process in which these fragmentation operations are repeatedly applied to each generated substructure. The result is a *directed acyclic graph* (DAG) with the intact molecule as its root and where the nodes represent the generated fragment search space. The directed edges in this DAG reflect the fragmentation process, with each edge describing the formation of a fragment from another fragment by breaking either a single non-ring bond or a pair of bonds within the same ring system. Each edge is weighted with the estimated log-probability that the associated fragmentation event occurs.

Because each fragment peak in the MS/MS spectrum should be assigned with the most likely substructure, the task is to find an optimal mapping between the fragments in the generated DAG and the peaks in the spectrum. To this end, a fragmentation tree[9] is computed which labels the peaks in the spectrum with molecular formulas. With this information, only those fragments can be considered for the assignment whose molecular formula matches one molecular formula of the now labelled peaks. Specifically, each node of the fragmentation tree is inserted into the DAG as a *pseudo-fragment* and then connected to all fragments whose molecular formula differ only in the amount of hydrogen atoms, allowing hydrogen rearrangements to be taken into account. Unlike the edges of the DAG, these pseudo-fragments are weighted with positive scores proportional to the relative intensities of their corresponding fragment peaks. In order to calculate the optimal mapping between the fragments of the candidate molecule and the peaks of the spectrum, the Maximum Arborescence Problem is solved where the subtree with maximum weight, i.e. the sum of its edge and node weights, is computed in the DAG. This maximum arborescence defines the optimal mapping, by assigning each peak the fragment which is connected by an edge to the corresponding pseudo-fragment. Furthermore, the weight of this maximum arborescence represents the score of the corresponding candidate molecule.

Due to the NP-hardness of the Maximum Arborescence Problem[10] and the size of these DAGs, EPIMETHEUS uses a heuristic to compute the maximum subtree. This heuristic is similar to the CriticalPath[1] heuristic from [11].

Supplementary Table 4. The reaction SMARTS used for each library enumeration

SEL 1	Reaction SMARTS
Fmoc-deprotection	[#7:17]-[#6:16](=[O:18])-[#8:15]-[#6:14]-[#6:1]-1-[c:5]2[c:9][c:8][c:7][c:6][c:4]2-[c:3]2[c:10][c:11][c:12][c:13][c:2]-12>>[#7:17].[O:18]=[#6:16]-[#8:15]-[#6:14]-[#6:1]-1-[c:2]2[c:13][c:12][c:11][c:10][c:3]2-[c:4]2[c:6][c:7][c:8][c:9][c:5]-12
Amide bond formation	[#7;A;X3;H2,H1;!$(NC=O);!$(NC=CC=O);!$(NC=S);!$(NC=N)!$(N-S):3].[#8;A;X1H0-,X2H1][#6;A;X3:2]=[O:1]>>[#7;A;X3:3][#6;X3:2]=[O;X1:1]

SEL 2	Reaction SMARTS
Fmoc-deprotection	[#7:17]-[#6:16](=[O:18])-[#8:15]-[#6:14]-[#6:1]-1-[c:5]2[c:9][c:8][c:7][c:6][c:4]2-[c:3]2[c:10][c:11][c:12][c:13][c:2]-12>>[#7:17].[O:18]=[#6:16]-[#8:15]-[#6:14]-[#6:1]-1-[c:2]2[c:13][c:12][c:11][c:10][c:3]2-[c:4]2[c:6][c:7][c:8][c:9][c:5]-12
Amide bond formation	[#7;A;X3;H2,H1;!$(NC=O);!$(NC=CC=O);!$(NC=S);!$(NC=N)!$(N-S):3].[#8;A;X1H0-,X2H1][#6;A;X3:2]=[O:1]>>[#7;A;X3:3][#6;X3:2]=[O;X1:1]
NAS	[#7:1]-[#6:2](=[O:3])-[c:4]1[c:5][c:6][c:7](F)[c:8]([c:10]1)-[#7+](-[#8-])=O.[#7;A;H2X3;!$(NC=[!#6]);!$(N-S):15]>>[#7:1]-[#6:2](=[O:3])-[c:4]1[c:5][c:6][c:7](-[#7:15])[c:8]([c:10]1)-[#7+](-[#8-])=O
NO_2 reduction	[#7:9]-[#6:7](=[O:10])-[c:5]1[c:4][c:3][c:2][c:1]([c:6]1)-[#7+](-[#8-])=O>>[#7:9]-[#6:7](=[O:10])-[c:5]1[c:4][c:3][c:2][c:1](-[#7:8])[c:6]1
Cyclization	[#7:11]-[#6:9](=[O:10])-[c:6]1[c:5][c:4][c:2](-[#7:18])[c:3](-[#7:8])[c:7]1.[#6:13][#6;A;H1X3:12]=[O:15]>>[#6:13]-[c:12]1[n:18][c:2]2[c:4][c:5][c:6]([c:7][c:3]2[n:8]1)-[#6:9](-[#7:11])=[O:10]

SEL 3	Reaction SMARTS
Fmoc-deprotection	[#7:17]-[#6:16](=[O:18])-[#8:15]-[#6:14]-[#6:1]-1-[c:5]2[c:9][c:8][c:7][c:6][c:4]2-[c:3]2[c:10][c:11][c:12][c:13][c:2]-12>>[#7:17].[O:18]=[#6:16]-[#8:15]-[#6:14]-[#6:1]-1-[c:2]2[c:13][c:12][c:11][c:10][c:3]2-[c:4]2[c:6][c:7][c:8][c:9][c:5]-12
Amide bond formation	[#7;A;X3;H2,H1;!$(NC=O);!$(NC=CC=O);!$(NC=S);!$(NC=N)!$(N-S):3].[#8;A;X1H0-,X2H1][#6;A;X3:2]=[O:1]>>[#7;A;X3:3][#6;X3:2]=[O;X1:1]
Suzuki-Miyaura coupling	Br[*:1].[#8]-[#5](-[#8])-[*:2]>>[#6:2]-[*:1]

Protecting group	Deprotection SMARTS
Fmoc	[#7:17]-[#6:16](=[O:18])-[#8:15]-[#6:14]-[#6:1]-1-[c:5]2[c:9][c:8][c:7][c:6][c:4]2-[c:3]2[c:10][c:11][c:12][c:13][c:2]-12>>[#7:17].[O:18]=[#6:16]-[#8:15]-[#6:14]-[#6:1]-1-[c:2]2[c:13][c:12][c:11][c:10][c:3]2-[c:4]2[c:6][c:7][c:8][c:9][c:5]-12
Trt	[N:13][C:14]([c:12]1[c:1][c:2][c:3][c:4][c:5]1)([c:11]1[c:6][c:7][c:8][c:9][c:10]1)[c:15]1[c:16][c:17][c:18][c:19][c:20]1>>[N;A;X3,H2:13]
Trt	[n:13][C:14]([c:12]1[c:1][c:2][c:3][c:4][c:5]1)([c:11]1[c:6][c:7][c:8][c:9][c:10]1)[c:15]1[c:16][c:17][c:18][c:19][c:20]1>>[n;A;X3,H1:13]
Boc	[N:3]-[#6:4](=[O:10])-[#8:5][C:6]([#6:7])([#6:8])[#6:9]>>[N;A;X3:3]
Boc	[n:3]-[#6:4](=[O:10])-[#8:5][C:6]([#6:7])([#6:8])[#6:9]>>[n;A;X3;H1:3]
OtBu	[#6;H3:5][C:2]([#6;H3:4])([#6;H3:3])[#8:1]>>[#8:1]
NtBu	[#6;H3:5][C:2]([#6;H3:4])([#6;H3:3])[#7:1]>>[#7:1]
Pbf	[#6:11]-[#8:10]-[c:9]1[c:8][c:6](-[#6:7])[c:5]([c:14](-[#6:15])[c:12]1-[#6:13])[S:2]([#7:1])(=[O:3])=[O:4]>>[#7:1]
AA1	[#6:6][C:5]1([#6:7])[#8:4]-[c:3]2[c:2][c:1][c:11][c:10][c:9]2-[#8:8]1>>[#8:4]-[c:3]1[c:2][c:1][c:11][c:10][c:9]1-[#8:8]
CA31	[#6:1][C:2]1([#6:10])[#8:3]-[#6@@H:4]-2-[#6:9]-[#8:8]-[#6:7]-[#6@@H:5]-2-[#8:6]1>>[#8:6]-[#6@H:5]-1-[#6:7]-[#8:8]-[#6:9]-[#6@H:4]-1-[#8:3]
CA40	[#6:8]-1-[#6:5][C:6]2([#6:7]-1)[#8:1]-[#6:2]-[#6:3]-[#8:4]2>>O=[#6:6]-1-[#6:7]-[#6:8]-[#6:5]-1
CA52	[O:1]=[#6:2]-[#6:3]-1-[#6:4]-[#6:5][C:6]2([#6:7]-[#6:8]-1)[#8:9]-[#6:10]-[#6:11]-[#8:12]2>>[O:1]=[#6:2]-[#6:3]-1-[#6:8]-[#6:7]-[#6:6](=[O:12])-[#6:5]-[#6:4]-1
CA76	[#7:1]-[#6:2](=[O:17])-[#8:3]-[#6:4](-[c:5]1[c:6][c:7][c:8][c:9][c:10]1)-[c:11]1[c:12][c:13][c:14][c:15][c:16]1>>[#7:1]

5. Building block scoring & selection

Fmoc-amino acid and carboxylic acid BBs were selected based on their druglike properties (molecular weight (MW), logP, hydrogen bond donors (HBD), hydrogen bond acceptors (HBA), and total polar surface area (TPSA)), using the following python scripts "BB selection_main.py", "BB_selection_utils.py" and "BB_selection_reactions.py" which are available through Zenodo (https://doi.org/10.5281/zenodo.14070388).

Chemspace provided lists of their in-stock Fmoc-amino acids and carboxylic acids, containing 1.7K and 6.3K building blocks (BBs), respectively. These lists were then refined to 1,000 BBs each by applying filters for price, availability, and compatibility with our library design. The refined lists were then used as input for the "BB selection_main.py" script, where the virtual library was enumerated based on the provided reaction SMARTS.

For each library member the molecular properties MW, HBD, HBA, logP and TPSA were calculated. The scoring of each library member was determined by abiding the following thresholds: MW<500, HBA<10, HBD<5, logP<5 and TPSA<140. Per matching properties it would score one point, resulting in a maximum score of 5 per library member. The scores were grouped per BB and the ranked BBs were used to select the most drug-like BBs for our library. Selected BBs can be seen in Section 10.1. List of building blocks.

6. Library synthesis and characterization

6.1. SEL 1: Carboxylic acid library

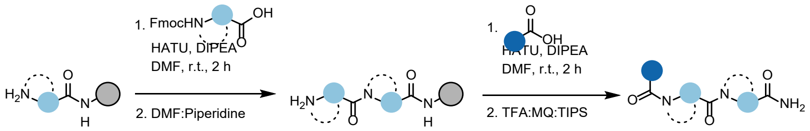

TentaGel S NH_2 resin (30 µm, 0.24 mmol/g loading, 2.625 g, 630 µmol, 1.0 eq.) functionalized with Fmoc-Rink Amide linker was over divided over 62 fritted syringes. A solution of each Fmoc-protected amino-acid **AA1-AA62** (30 µmol, 3.0 eq), HATU (29.8 µmol, 0.4 M, 2.98 eq) and DIPEA (90 µmol, 9.0 eq) in DMF was added to the resin and reacted for 2 hours. The resin was pooled and washed with DMF (5 x 2 mL) and 20% piperidine in DMF (1 x 2 mL) before incubating with 20% piperidine in DMF for 10 min. The resin was washed with DMF (5 x 2 mL) and split over 62 fritted syringes.

The second building block was incorporated using the same reaction conditions. A solution of each Fmoc-protected amino-acid **AA1-AA62** (30 µmol, 3.0 eq), HATU (29.8 eq. 0.4 M, 2.98 eq) and DIPEA (90 µmol, 9.0 eq) in DMF was added to the resin and reacted for 2 hours. The resin was pooled into a fritted syringe (20 mL) and washed with DMF (5 x 2 mL) and 20% piperidine in DMF (1x, 2 mL) before incubating with 20% piperidine in DMF for 10 min

For the incorporation of the 3rd building block, the resin (350 µmol, 1.0 eq) was divided over 130 eppendorf tubes. A solution of each carboxylic acid **CA1-CA130** (8.08 µmol, 3.0 eq), HATU (80.2 µL, 0.1 M, 8.02 µmol, 2.98 eq) and DIPEA (4.22 µL, 24.23 µmol, 9.0 eq) in DMF was added to the resin (2.69 µmol). The reactions were stirred overnight at r,t. The resin was pooled and washed with DMF (5 x 2 mL) and DCM (5 x 2 mL).

The resin was incubated for 1.5 hour with a solution of TFA:H_2O:TIPS (92.5:5:2.5) and washed once with a solution of TFA:H_2O:TIPS (92.5:5:2.5). TFA was evaporated under a stream of N_2 and the library was purified using reverse phase column chromatography with a stepwise gradient of 00-70-100% MeCN:H_2O (0.1% TFA).

6.2. SEL 2: Benzimidazole library

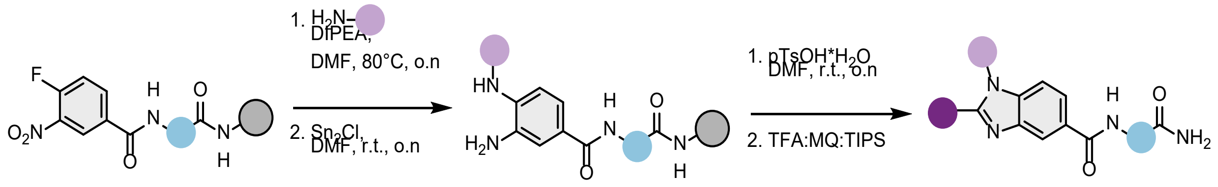

TentaGel S NH_2 resin (30 µm, 0.24 mmol/g loading, 2.625 g, 630 µmol, 1.0 eq.) functionalized with Fmoc-Rink Amide linker was over divided over 62 fritted syringes. A solution of each Fmoc-protected amino-acid **AA1-AA62** (30 µmol, 3.0 eq), HATU (29.8 µmol, 0.4 M, 2.98 eq) and DIPEA (90 µmol, 9.0 eq) in DMF was added to the resin and reacted for 2 hours. The resin was pooled and washed with DMF (5 x 2 mL) and 20% piperidine in DMF (1x, 2 mL) before incubating with 20% piperidine in DMF for 10 min. The resin was washed with DMF (5 x 2 mL) and a solution of 4-fluoro-3-nitrobenzoic acid (175 mg, 945 µmol, 3.0 eq), HATU (2.346 mL, 939 µmol, 2.98 eq) and DIPEA (494 µL, 2.835 mmol, 9.0 eq) in DMF was added to the resin. After 1h the reaction as washed with DMF (5 x 2 mL).

The resin was divided over 52, eppendorf tubes, to which amines **AM1-AM52** (59.6 µmol, 10 eq.) and DIPEA (10.55 µL, 59.6 µmol, 10 eq.) in DMF (150 µL, 0.4 M) were added. The mixture was shaken at 1 x g overnight at 80 °C. The resin was pooled and washed with DMF (5 x 2 mL) and DCM (5 x 2 mL). A solution of 1.0 M $SnCl_2$ (3.73 g, 19.7 mmol, 62.5 eq.) in DMF was added to the resin (315 µmol). The mixture was incubated overnight at r.t., whereafter the resin was washed with DMF:H_2O (1:1, 5 x 2 mL), with DMF (5x, 2 mL) and DCM (5x, 2 mL).

The resin (4.31 µmol, 1.0 eq.) was split over 67 eppendorf tubes to which the appropriate aldehyde **AL1-AL67** (0.25 M in DMF (103.3 µL), 25 µmol, 5 eq.) and p-TsOH•H_2O (4.76 mg, 25 µmol, 5 eq.) were added. The mixture was incubated overnight at 1 x g, overnight at r.t. After incubation, the resin was washed DMF (5 x 2 mL) and DCM (5 x 2 mL). The resin was incubated for 1.5 hour with a solution of TFA:H_2O:TIPS (92.5:5:2.5) and washed once with a solution of TFA:H2O:TIPS (92.5:5:2.5). TFA was evaporated under a stream of N_2 and the library was purified using reverse phase column chromatography with a stepwise gradient of 00-70-100% MeCN:H_2O (0.1% TFA).

6.3. SEL 3: Suzuki-Miyaura library

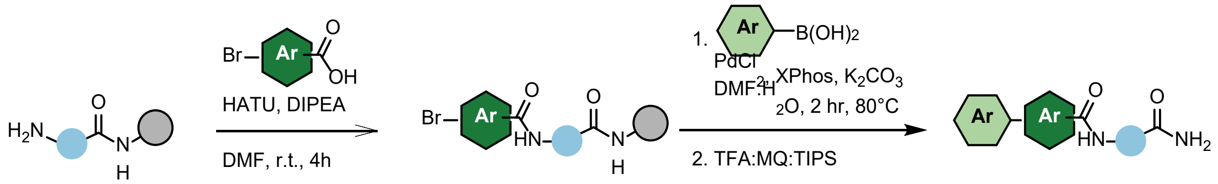

TentaGel S NH_2 resin (30 µm, 0.24 mmol/g loading, 1.1 g, 265 µmol, 1.0 eq.) functionalized with Fmoc-Rink Amide linker was over divided over 60 fritted syringes. A solution of each Fmoc-protected amino-acid (Supplementary Table 12) (12.8 µmol, 3.0 eq), HATU (12.7 µmol, 0.4 M, 2.98 eq) and DIPEA (38.5 µmol, 9.0 eq) in DMF was added to the resin and reacted for 4 hours. The resin was pooled into a fritted syringe (20 mL) and washed with DMF (5 x 2 mL) and 20% piperidine in DMF (1x, 2 mL) before incubating with 20% piperidine in DMF for 10 min.

The resin was divided over 10 eppendorf tubes. A solution of aryl bromide **AB1-AB10** (26.50 µmol, 3.0 eq), 0.4M HATU (78.97 µmol, 197 µL, 2.98 eq), DIPEA (41.50 µL, 238.5 µmol, 9.0 eq) and DMF (400 µL) were added to the resin (26.50 µmol). After 4h, the resin was washed with DMF (5 x 2 mL). Aryl bromide **AB10** was deprotected by washing with 20% piperidine in DMF (1x, 2 mL) before incubating with 20% piperidine in DMF for 10 min. A solution of benzoic acid (9.7 mg, 79.50 µmol, 3 eq.), DIPEA (41.50 µL, 238 µmol, 9.0 eq) and DMF (400 µL) were added to the resin containing **AB10** (26.50 µmol) and reacted for 1h. The resin was combined in a fritted syringe and was washed with DMF (5x).

The resin was divided over 53 eppendorf tubes. Boronic acid **BA1-BA53** (10 µmol, 2.0 eq.), K_2CO_3 (1.4 mg, 10 µmol, 2.0 eq.), $PdCl_2$ (89 µg, 0.5 µmol, 10 mol%), XPhos (0.48 mg, 1 µmol, 20 mol%) and DMF (100 µL) were added to the resin (5.0 µmol). The reaction was stirred at 1 x g at 80 °C for 21h. The resin was washed with DMF (5 x 2 mL) and DCM (5x) and incubated for 1:45 h with a solution of TFA:H_2O:TIPS (92.5:5:2.5). The resin was washed once with a solution of TFA:H_2O:TIPS (92.5:5:2.5) whereafter the TFA was evaporated. The library was purified with using reverse phase column chromatography with a stepwise gradient 00-70-100% MeCN (0.1% TFA).

6.4. SEL 4: Focused 4.000 membered library

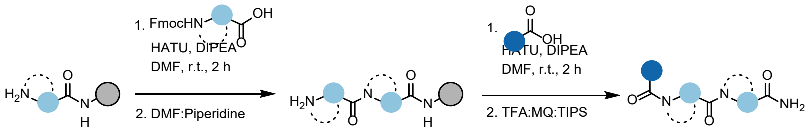

TentaGel S NH_2 resin (90 µm, 385 mg, 0.26 mmol/g, 100 µmol, 1.0 eq.) functionalized with Fmoc-Rink Amide linker was over divided over 20 fritted syringes. A solution of each Fmoc-protected amino-acid (Supplementary Table 15) (15 µmol, 3.0 eq), HATU (0.4 M, 2.98 eq) and DIPEA (9.0 eq) in DMF was added to the resin and reacted for 2.5 hours. The resin was pooled and washed with DMF (5 x 2 mL) and 20% piperidine in DMF (1 x 2 mL) before incubating with 20% piperidine in DMF for 10 min. The resin was washed with DMF (5 x 2 mL) and split over 20 fritted syringes. The second building block was incorporated using the same reaction conditions. A solution of each Fmoc-protected amino-acid (Supplementary Table 15) (15, µmol, 3.0 eq), HATU (0.4 M, 2.98 eq) and DIPEA (9.0 eq) in DMF was added to the resin and reacted for 2.5 hours. The resin was pooled into a fritted syringe (20 mL) and washed with DMF (5 x 2 mL) and 20% piperidine in DMF (1x, 2 mL) before incubating with 20% piperidine in DMF for 10 min

For the incorporation of the 3rd building block, the resin was divided over 10 syringes. A solution of each carboxylic acid (Supplementary Table 16) (30 µmol, 3.0 eq), HATU (149 µL, 0.2 M, 29.80 µmol, 2.98 eq) and DIPEA (15.7 µL, 90 µmol, 9.0 eq) in DMF was added to the resin (10 µmol) and reacted for 2.5 hours. The resin was pooled and washed with DMF (5 x 2 mL) and DCM (5 x 2 mL).

The resin was incubated for 1.5 hour with a solution of TFA:H_2O:TIPS (92.5:5:2.5) and washed once with a solution of TFA:H_2O:TIPS (92.5:5:2.5). TFA was evaporated under a stream of N_2 and the library was purified using reverse phase column chromatography with a stepwise gradient of 00-70-100% MeCN:H_2O (0.1% TFA).

7. Building block synthesis

7.1. CA131

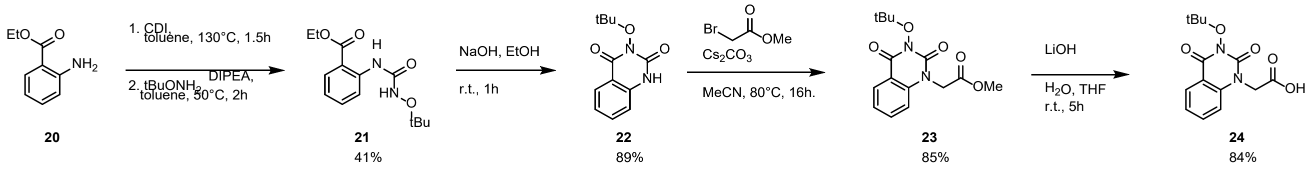

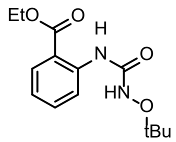

21

Ethyl 2-aminobenzoate (500 mg, 3.03 mmol, 1.0 eq.) and CDI (736 mg, 4.54 mmol, 1.5 eq.) were dissolved in dry toluene (15 mL) and heated under reflux for 1.5 h. The reaction was cooled to 50 °C and O-(tert-butyl)hydroxylamine hydrochloride (570 mg, 4.45 mmol, 1.5 eq.) and DIPEA (791 µL, 4.45 mmol, 1.5 eq.) were added to the reaction. The reaction was stirred overnight at 50°C. The reaction was quenched with water and the aqueous phase extracted with EtOAc (2x). The organic layer was washed with H_2O (2x) and brine, dried over $MgSO_4$ and concentrated in vacuo. The crude mixture was purified by column chromatography (PE:EtOAc 10-60%). The product was obtained as a white solid (350 mg, 1.25 mmol, 41%).

^{1}H -NMR: (400 MHz, DMSO) δ 11.09 (s, 1H), 9.45 (s, 1H), 8.61 (dd, J = 8.5, 1.2 Hz, 1H), 7.99 (dd, J = 8.0, 1.7 Hz, 1H), 7.59 (td, J = 7.2, 1.7 Hz, 1H), 4.35 (q, J = 7.1 Hz, 2H), 1.35 (t, J = 7.1 Hz, 3H), 1.27 (s, 9H). **^{13}C NMR** (101 MHz, DMSO) δ 161.12, 149.91, 138.63, 134.99, 127.48, 122.52, 115.26, 114.39, 86.18, 27.24. **MS (ESI):** m/z calculated for $C_{14}H_{20}N_2O_4$ [M+H] = 281.15, found = 281.00

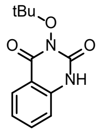

22

Intermediate **21** (290 mg, 1.04 mmol, 1.0 eq.) was taken up in 0.25M NaOH in EtOH (20 mL, 5.0 mmol, 4.8 eq.) and stirred for 1 h at room temperature. The reaction was concentrated in vacuo. Water (10 mL) and acetic acid (0.6 mL) were added. The precipitated product was filtered, washed with H_2O (3 x 10 mL) and dried in vacuo. 3-(tert-butoxy)quinazoline-2,4(1H,3H)-dione (216 mg, 0.93 mmol, 89% yield) was collected as a white solid.

^{1}H NMR: (400 MHz, DMSO) δ 11.51 (s, 1H), 7.93 (dd, *J* = 8.0, 1.5 Hz, 1H), 7.67 (ddd, *J* = 8.5, 7.3, 1.6 Hz, 1H), 7.23 (td, *J* = 8.2, 7.2, 1.0 Hz, 1H), 7.19 (d, *J* = 8.1 Hz, 1H), 1.33 (s, 9H). **^{13}C NMR** (101 MHz, DMSO) δ 161.12, 149.91, 138.63, 134.99, 127.48, 122.52, 115.26, 114.39, 86.18, 27.24. **MS (ESI):** m/z calculated for $C_{12}H_{14}N_2O_3$ [M-tBu+H] =179.00, found = 178.95

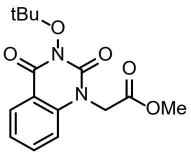

23

3-(tert-butoxy)quinazoline-2,4(1H,3H)-dione (1.5 g, 6.40 mmol, 1.0 eq.) was dissolved in MeCN (100 mL), to which was added methyl bromoacetate (0.89 mL 9.60 mmol, 1.5 eq.) and cesium carbonate (6.22 g, 19.2 mmol, 3.0 eq.). The reaction was heated to 80 °C for 16 h. The reaction was concentrated *in vacuo* and crude was redissolved in ethyl acetate (100 mL) and water (100 mL). The organic layers were washed with saturated brine solution (60 mL), dried over $MgSO_4$. And concentrated *in vacuo.* The crude was purified by column chromatography (5-55% EtOAc:PE). Fractions containing target product were combined and concentrated to yield methyl 2-(3-(tert-butoxy)-2,4-dioxo-3,4-dihydroquinazolin-1(2H)-yl)acetate (1.67 g, 5.45 mmol, 85%) as a colorless oil.

^{1}H NMR (400 MHz, DMSO) δ 8.09 (dd, *J* = 7.8, 1.6 Hz, 1H), 7.77 (ddd, *J* = 8.7, 7.2, 1.6 Hz, 1H), 7.45 (d, *J* = 8.5 Hz, 1H), 7.40 – 7.25 (m, 1H), 4.99 (d, *J* = 10.7 Hz, 2H), 3.72 (s, 3H), 1.33 (s, 9H). **^{13}C NMR** (101 MHz, DMSO) δ 168.53, 150.53, 138.94, 135.52, 128.18, 123.41, 115.00, 114.62, 86.70, 52.44, 45.03, 27.12. **MS (ESI):** m/z calculated for $C_{15}H_{18}N_2O_5$ [M-tBu+H] =251.07, found = 250.90

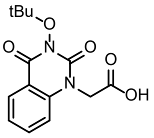

24

The intermediate **23** (135 mg, 0.44 mmol, 1.0 eq.) was diluted in THF (20 mL), to which a solution of 0.5M LiOH in H_2O (2.64 mmol, 1.32 mmol, 3.0 eq.) was added. The reaction was stirred at room temperature for 5 h. The reaction was concentrated in vacuo, diluted in ethyl acetate and washed with 1M citric acid solution. The organic layer was washed with brine, dried over $MgSO_4$ and concentrated *in vacuo*. The crude product was purified by reverse phase column chromatography (C-18, 2-100% MeCN in H_2O, 0.1% TFA) and afforded a white solid (109 mg, 0.37 mmol, 84% yield).

^{1}H NMR (400 MHz, DMSO) δ 13.22 (s, 1H), 8.08 (dd, *J* = 7.9, 1.6 Hz, 1H), 7.78 (ddd, *J* = 8.7, 7.2, 1.7 Hz, 1H), 7.43 (d, *J* = 8.4 Hz, 1H), 7.34 (ddd, *J* = 8.0, 7.3, 0.8 Hz, 1H), 4.88 (d, *J* = 13.3 Hz, 2H), 1.33 (s, 9H). **^{13}C NMR:** (101 MHz, DMSO) δ 169.84, 160.63, 151.05, 139.60, 135.98, 128.62, 123.76, 115.44, 115.15, 87.14, 45.56, 27.63. **MS (ESI):** m/z calculated for $C_{14}H_{16}N_2O_5$ [M-tBu+H] =237.05, found = 236.95

7.2. CA132

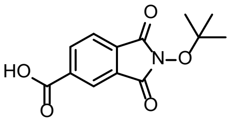

25

To a solution of O-(tert-butyl)hydroxylamine hydrochloride (1.60 g, 10 mmol, 1.0 equiv.) in pyridine (20 mL) was added trimellitic anhydride (1.92 g, 10 mmol, 1.0 equiv.) at room temperature. The reaction mixture was heated to reflux for 16 h. After cooling to room temperature, the reaction was acidified with a 4 M acetic acid solution. The organic substances were extracted with EtOAc (3x 100 mL), washing with saturated brine solution, and dried over $MgSO_4$ and concentrated *in vacuo.* The crude product was purified by reverse phase column chromatography (C-18, 2-100% MeCN in H_2O, 0.1% TFA). The product was isolated as a white solid (1.315 g, 5.0 mmol, 50%).

^{1}H NMR: (400 MHz, DMSO) δ 13.77 (s, 1H), 8.37 (dd, *J* = 7.8, 1.5 Hz, 1H), 8.21 (s, 1H), 7.99 (dd, *J* = 7.8, 0.7 Hz, 1H), 1.35 (s, 9H). **^{13}C NMR:** (101 MHz, DMSO) δ 166.26, 165.07, 165.05, 135.96, 132.88, 129.99, 124.13, 123.63, 86.88, 27.45.

8. Affinity selection against CAIX

8.1. Procedure

MyOne Streptavidin T1 Dynabeads (100 µL of 10 mg/mL stock per well, measured in duplicates) were washed with 3x 1 mL 10% FBS, 1x PBS, 0.02% Tween-20. The beads were incubated with biotinylated CAIX (100 µL, 1.5 µM per well) in 10% FBS, 1x PBS, 0.02% Tween-20 for 1h at 4°C. The beads were washed 2x 1 mL 10% FBS, 1x PBS, 0.02% Tween-20, 400 µM d-biotin and 1x 1 mL 10% FBS, 1x PBS, 0.02% Tween-20 before incubating with the library (100 µL, 100 fmol/member per well) in 10% FBS, 1x PBS for 1h at 4°C. The beads were washed with 5x 1 mL of 1x PBS and subsequently eluted with 2x MeCN:MQ (1:1) 0.1%FA (100 µL per well).

Sample preparation after AS: Samples from the affinity selection procedure were lyophilized and resuspended in 50 µL MQ 0.1%FA. The StageTips were prepared as described by Rappsilber et al. using C18 material from Empore SPE 47 mm discs (66883-U, Merck) (*46*). The StageTips were pre-conditioned with 200 µL MeOH, 200 µL of 0.1% (v/v) FA in MeCN and 200 µL of 0.1% (v/v) FA in MQ, respectively by centrifuging for 3 min at 300 x g. The samples were then loaded on the StageTips and washed with 200 µL of 0.1% (v/v) FA in MQ. Compounds were eluted by adding 200 µL of 0.1% (v/v) FA in MeCN:MQ (7:3). The samples were lyophilized before resuspending in 10 µL 0.1% (v/v) FA in UPLC-MS grade water. The samples were centrifuged for 5 min at 21,000 x g. Afterwards, 9 µL was transferred to a LC-MS vial and 8 µL was injected into the LC-MS/MS system.

8.2. Screening of SEL 1 against CAIX

8.2.1. List of hit structures

Supplementary Table 5. **Identified bindings for CAIX after AS-MS with SEL 1**

SMILES
O=C(N)C(NC(=O)C(N(C(=O)C1=CC=C(C=C1)S(=O)(=O)N)C)C(C)C)CC2=CC=C(Cl)C(F)=C2
O=C(N)C(NC(=O)C(N(C(=O)C1=CC=C(C=C1)S(=O)(=O)N)C)C(C)C)CCCCN
O=C(N)C(NC(=O)C(NC(=O)C1=CC=C(C=C1)S(=O)(=O)N)C(C)C)CC2=CC=C(C=C2)C(C)(C)C
O=C(N)C(NC(=O)C(NC(=O)C1=CC=C(C=C1)S(=O)(=O)N)C(O)C)CC2=CC=C(Cl)C(F)=C2
O=C(N)C(NC(=O)C(NC(=O)C1=CC=C(C=C1)S(=O)(=O)N)C)CC2=CC=C(Cl)C(F)=C2
O=C(N)C(NC(=O)C(NC(=O)C1=CC=C(C=C1)S(=O)(=O)N)C2CC2)CC3=CC=C(C=C3)C(C)(C)C
O=C(N)C(NC(=O)C(NC(=O)C1=CC=C(C=C1)S(=O)(=O)N)C2CC2)CC3=CC=C(Cl)C(F)=C3
O=C(N)C(NC(=O)C(NC(=O)C1=CC=C(C=C1)S(=O)(=O)N)C2CC2)CC3=CNC=4C=CC=CC43
O=C(N)C(NC(=O)C(NC(=O)C1=CC=C(C=C1)S(=O)(=O)N)C2CC2)CCCCCCCC
O=C(N)C(NC(=O)C(NC(=O)C1=CC=C(C=C1)S(=O)(=O)N)CC2=CC=C(Br)C=C2)CC=3N=CNC3
O=C(N)C(NC(=O)C(NC(=O)C1=CC=C(C=C1)S(=O)(=O)N)CC2=CC=C(Cl)C(F)=C2)CC3=NC=CC=C3
O=C(N)C(NC(=O)C(NC(=O)C1=CC=C(C=C1)S(=O)(=O)N)CC2=CC=C(O)C=C2)CC3CCCC3
O=C(N)C(NC(=O)C(NC(=O)C1=CC=C(C=C1)S(=O)(=O)N)CC2=CC=C(O)C=C2)CC3CCCCC3
O=C(N)C(NC(=O)C(NC(=O)C1=CC=C(C=C1)S(=O)(=O)N)CC2=CC=C(O)C=C2)CC=3N=CNC3
O=C(N)C(NC(=O)C(NC(=O)C1=CC=C(C=C1)S(=O)(=O)N)CC2=CC=C(O)C=C2)CCCCCCCC
O=C(N)C(NC(=O)C(NC(=O)C1=CC=C(C=C1)S(=O)(=O)N)CC2=CNC=3C=CC=CC32)CC=4C=CC=CC4
O=C(N)C(NC(=O)C(NC(=O)C1=CC=C(C=C1)S(=O)(=O)N)CC2=CNC=3C=CC=CC32)CCCCN
O=C(N)C(NC(=O)C(NC(=O)C1=CC=C(C=C1)S(=O)(=O)N)CC2CCC2)CC3=CC=C(Cl)C(F)=C3
O=C(N)C(NC(=O)C(NC(=O)C1=CC=C(C=C1)S(=O)(=O)N)CC=2C=CC=CC2)CC=3C=CC=CC3
O=C(N)C(NC(=O)C(NC(=O)C1=CC=C(C=C1)S(=O)(=O)N)CC=2N=CNC2)CCCCCCCC
O=C(N)C(NC(=O)C(NC(=O)C1=CC=C(C=C1)S(=O)(=O)N)CC=2OC=CC2)CC3=CC=C(Br)C=C3
O=C(N)C(NC(=O)C(NC(=O)C1=CC=C(C=C1)S(=O)(=O)N)CC=2OC=CC2)CC3=CC=C(C=C3)C(C)(C)C
O=C(N)C(NC(=O)C(NC(=O)C1=CC=C(C=C1)S(=O)(=O)N)CC=2OC=CC2)CC=3N=CNC3
O=C(N)C(NC(=O)C(NC(=O)C1=CC=C(C=C1)S(=O)(=O)N)CCCCC)CC2=CC=C(O)C(O)=C2
O=C(N)C(NC(=O)C(NC(=O)C1=CC=C(C=C1)S(=O)(=O)N)CCCNC(=N)N)CC2=CC=C(Cl)C(F)=C2
O=C(N)C(NC(=O)C(NC(=O)C1=CC=C(C=C1)S(=O)(=O)N)CCCNC(=N)N)CC=2C=CC=CC2

O=C(N)C(NC(=O)C1CCC(NC(=O)C2=CC=C(C=C2)S(=O)(=O)N)C1)CC3=CC=C(C=C3)C(C)(C)C
O=C(N)C(NC(=O)C1CN(C(=O)C2=CC=C(C=C2)S(=O)(=O)N)C1)CC3=CC=C(Cl)C(F)=C3
O=C(N)C(NC(=O)C1CN(C(=O)C2=CC=C(C=C2)S(=O)(=O)N)C1)CCCCCCCC
O=C(N)C(NC(=O)C1N(C(=O)C2=CC=C(C=C2)S(=O)(=O)N)C=3C=CC=CC3C1)CCCCC
O=C(N)C(NC(=O)C1N(C(=O)C2=CC=C(C=C2)S(=O)(=O)N)CC3(CC3)C1)CC4=CC=C(Cl)C(F)=C4
O=C(N)C(NC(=O)C1N(C(=O)C2=CC=C(C=C2)S(=O)(=O)N)CCCC1)CC3=NC=CC=C3
O=C(N)C(NC(=O)C1N(C(=O)C2=CC=C(C=C2)S(=O)(=O)N)CCCC1)CCCCC
O=C(N)C(NC(=O)CC(NC(=O)C1=CC=C(C=C1)S(=O)(=O)N)CC2=CC=C(F)C(F)=C2)CC3=CNC=4C=CC
=CC43
O=C(N)C1(NC(=O)C(NC(=O)C2=CC=C(C=C2)S(=O)(=O)N)CC3=CC=C(Br)C=C3)CC1
O=C(N)C1CCC(CNC(=O)C(NC(=O)C2=CC=C(C=C2)S(=O)(=O)N)CC3=CC=C(O)C=C3)CC1
O=C(N)C1CN(C(=O)C(NC(=O)C2=CC=C(C=C2)S(=O)(=O)N)CC3=CC=C(Br)C=C3)C1
O=C(N)C1N(C(=O)C(NC(=O)C2=CC=C(C=C2)S(=O)(=O)N)CC3=CC=C(Br)C=C3)CCCC1
O=C(N)C1N(C(=O)C(NC(=O)C2=CC=C(C=C2)S(=O)(=O)N)CC3=CC=C(Cl)C(F)=C3)CC(F)(F)C1
O=C(N)C1N(C(=O)C(NC(=O)C2=CC=C(C=C2)S(=O)(=O)N)CC3=CC=C(O)C=C3)CCCC1
O=C(N)C1N(C(=O)C(NC(=O)C2=CC=C(C=C2)S(=O)(=O)N)CC3=CN=CN3COCC=4C=CC=CC4)CCCC1
O=C(N)C1N(C(=O)C(NC(=O)C2=CC=C(C=C2)S(=O)(=O)N)CCCCC)C=3C=CC=CC3C1
O=C(N)C1N(C(=O)CC(NC(=O)C2=CC=C(C=C2)S(=O)(=O)N)CC3=CC=C(F)C(F)=C3)CC(F)C1
O=C(N)C1N(C(=O)CC(NC(=O)C2=CC=C(C=C2)S(=O)(=O)N)CC3=CC=C(F)C(F)=C3)CSC1
O=C(N)CC(NC(=O)C(NC(=O)C1=CC=C(C=C1)S(=O)(=O)N)C(C)C)CC2=CC=C(F)C(F)=C2
O=C(N)CC(NC(=O)C(NC(=O)C1=CC=C(C=C1)S(=O)(=O)N)C2CC2)CC3=CC=C(F)C(F)=C3
O=C(N)CC(NC(=O)C(NC(=O)C1=CC=C(C=C1)S(=O)(=O)N)CC2=CC=C(Cl)C(F)=C2)CC3=CC=C(F)C(F)
=C3
O=C(N)CC(NC(=O)C(NC(=O)C1=CC=C(C=C1)S(=O)(=O)N)CC2=CC=C(O)C=C2)CC3=CC=C(F)C(F)=C
3
O=C(N)CC(NC(=O)C(NC(=O)C1=CC=C(C=C1)S(=O)(=O)N)CC=2N=CNC2)CC3=CC=C(F)C(F)=C3
O=C(N)CC(NC(=O)C(NC(=O)C1=CC=C(C=C1)S(=O)(=O)N)CC=2OC=CC2)CC3=CC=C(F)C(F)=C3
O=C(N)CC(NC(=O)C(NC(=O)C1=CC=C(C=C1)S(=O)(=O)N)CCCCC)CC2=CC=C(F)C(F)=C2
O=C(N)CC(NC(=O)C(NC(=O)C1=CC=C(C=C1)S(=O)(=O)N)CO)CC2=CC=C(F)C(F)=C2
O=C(N)CC(NC(=O)C1(NC(=O)C2=CC=C(C=C2)S(=O)(=O)N)CC1)CC3=CC=C(F)C(F)=C3
O=C(N)CC(NC(=O)C1(NC(=O)C2=CC=C(C=C2)S(=O)(=O)N)CCC(F)(F)CC1)CC3=CC=C(F)C(F)=C3
O=C(N)CC(NC(=O)C1(NC(=O)C2=CC=C(C=C2)S(=O)(=O)N)CCN(CC=3C=CC=CC3)CC1)CC4=CC=C(F
)C(F)=C4
O=C(N)CC(NC(=O)C1=CC=C(C=C1)S(=O)(=O)N)C(=O)NC(C(=O)N)C2CCCCC2
O=C(N)CC(NC(=O)C1=CC=C(C=C1)S(=O)(=O)N)C(=O)NC(C(=O)N)CC2=CC=C(C=C2)C(C)(C)C
O=C(N)CC(NC(=O)C1=CC=C(C=C1)S(=O)(=O)N)C(=O)NC(CC(=O)N)CC2=CC=C(F)C(F)=C2
O=C(N)CC(NC(=O)C1N(C(=O)C2=CC=C(C=C2)S(=O)(=O)N)CC(F)(F)C1)CC3=CC=C(F)C(F)=C3
O=C(N)CC(NC(=O)CC(NC(=O)C1=CC=C(C=C1)S(=O)(=O)N)CC2=CC=C(F)C(F)=C2)CC3=CC=C(F)C(F
)=C3
O=C(N)CC(NC(=O)CNC(=O)C1=CC=C(C=C1)S(=O)(=O)N)CC2=CC=C(F)C(F)=C2
O=C(N)CC1CCN(C(=O)C(NC(=O)C2=CC=C(C=C2)S(=O)(=O)N)CC3=CNC=4C=CC=CC43)CC1
O=C(N)CC1CCN(C(=O)C(NC(=O)C2=CC=C(C=C2)S(=O)(=O)N)CC=3C=CC=CC3)CC1
O=C(N)CCC(NC(=O)C1=CC=C(C=C1)S(=O)(=O)N)C(=O)NC(C(=O)N)CC2=CC=C(Cl)C(F)=C2
O=C(N)CCC(NC(=O)C1=CC=C(C=C1)S(=O)(=O)N)C(=O)NC(C(=O)N)CC=2C=CC=CC2
O=C(N)CN1CCN(C(=O)C(NC(=O)C2=CC=C(C=C2)S(=O)(=O)N)CCCCCCCC)CC1
O=C(N)CNC(=O)C(NC(=O)C1=CC=C(C=C1)S(=O)(=O)N)CC=2C=CC=CC2
O=C(N)CNC(=O)C(NC(=O)C1=CC=C(C=C1)S(=O)(=O)N)CCCCCCCC
O=C(N)CNC(=O)CC(NC(=O)C1=CC=C(C=C1)S(=O)(=O)N)CC2=CC=C(F)C(F)=C2
O=C(NC(C(=O)N(C)CC1CCC(C(=O)N)CC1)C2CC2)C3=CC=C(C=C3)S(=O)(=O)N
O=C(NC(C(=O)NC(C(=O)N)CCCCCCCC)CC1CCCC1)C2=CC=C(C=C2)S(=O)(=O)N
O=C(O)CC(NC(=O)C1=CC=C(C=C1)S(=O)(=O)N)C(=O)NC(CC(=O)N)CC2=CC=C(F)C(F)=C2
O=C(O)CC(NC(=O)CC(NC(=O)C1=CC=C(C=C1)S(=O)(=O)N)CC2=CC=C(F)C(F)=C2)C(=O)N
O=C(O)CCC(NC(=O)C1=CC=C(C=C1)S(=O)(=O)N)C(=O)NC(CC(=O)N)CC2=CC=C(F)C(F)=C2

List of structures identified after performing AS-MS in duplicates on CAIX using SEL 1. Using COMET with a least matching 1 out of the 5 biggest peaks and 5 ppm, 74 unique structures containing building block **CA77** were identified.

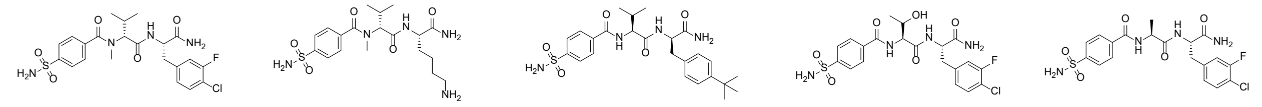

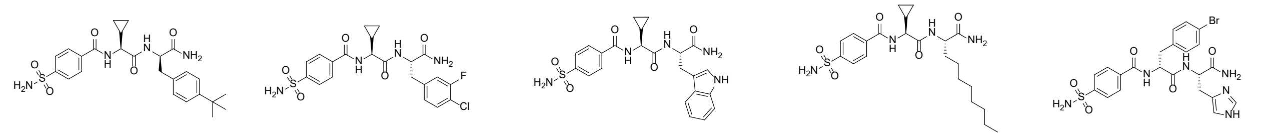

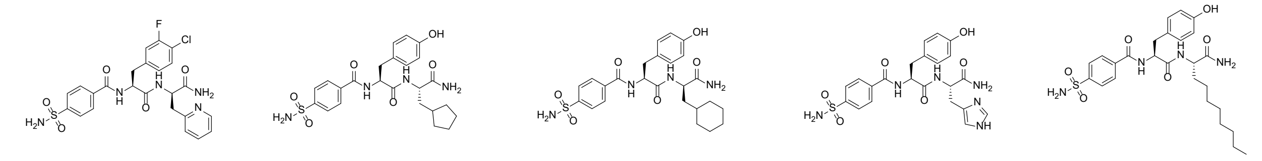

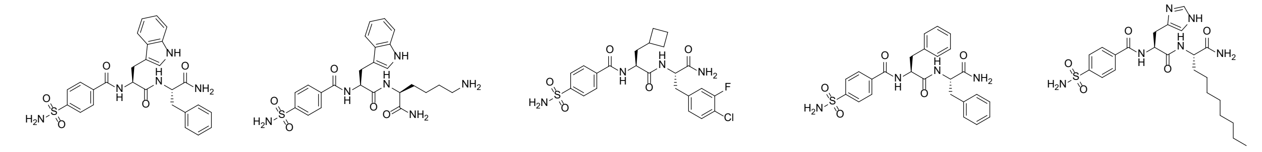

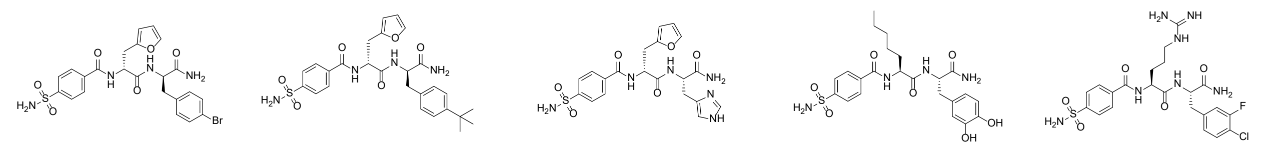

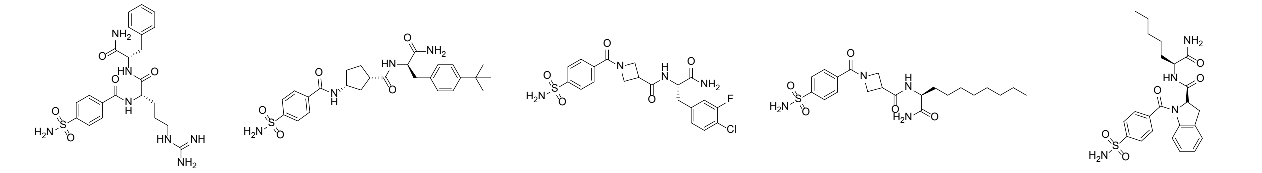

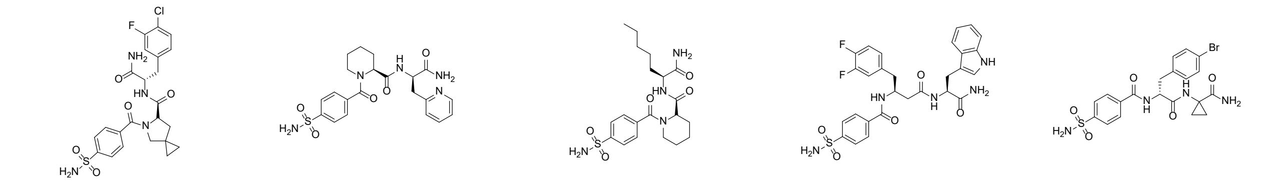

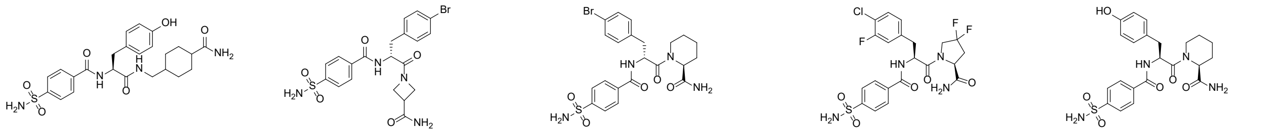

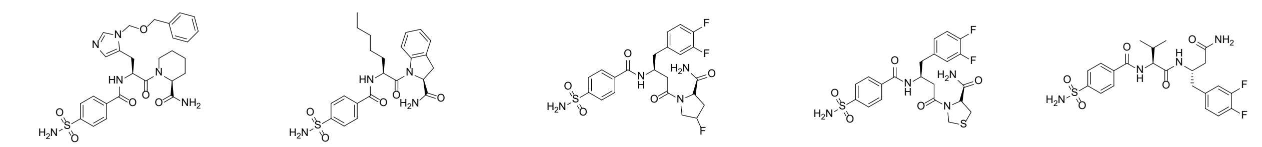

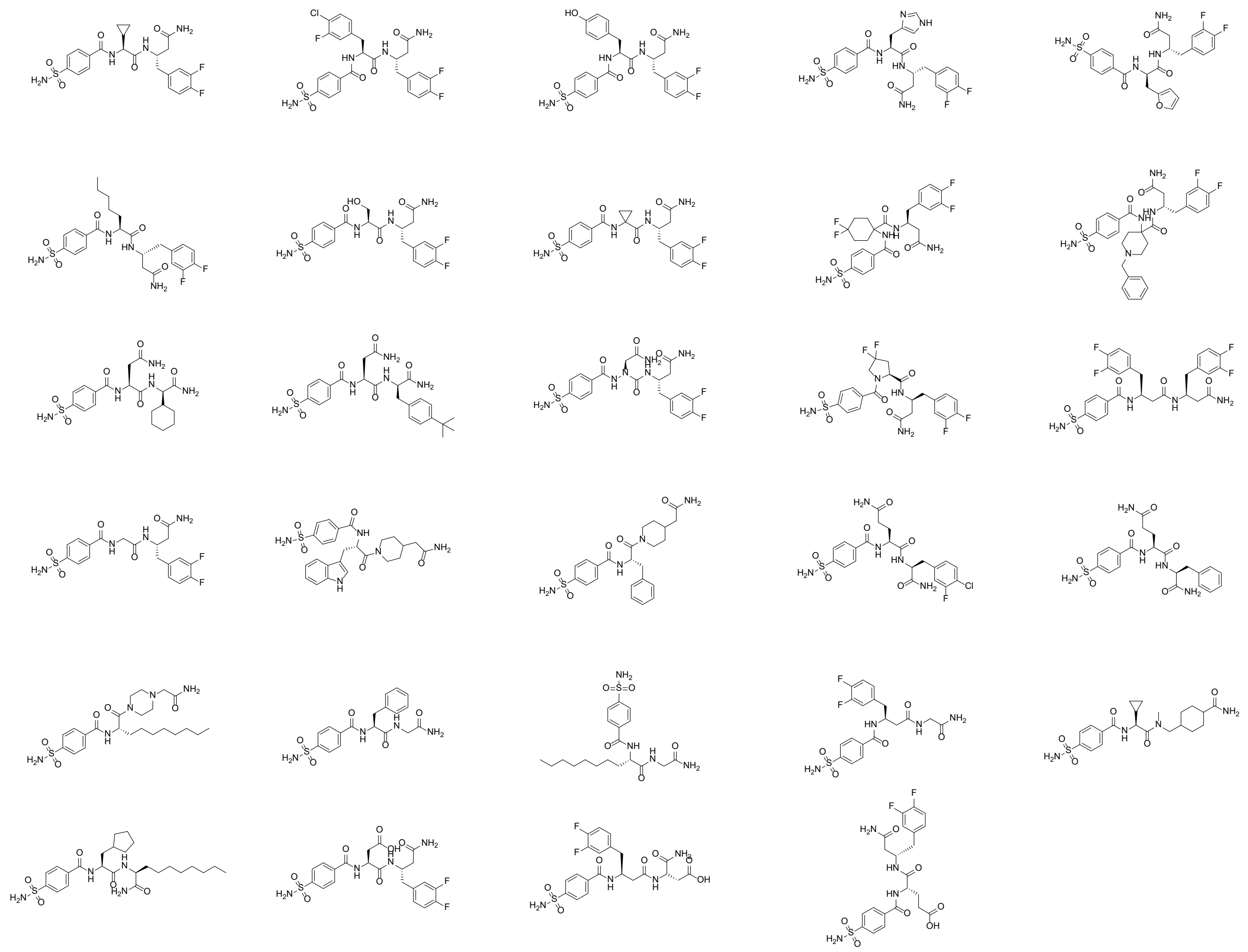

Supplementary Fig 23. Structures of the 74 hit compounds found after AS-MS against CAIX with SEL 1.

8.2.2. Enrichment plots

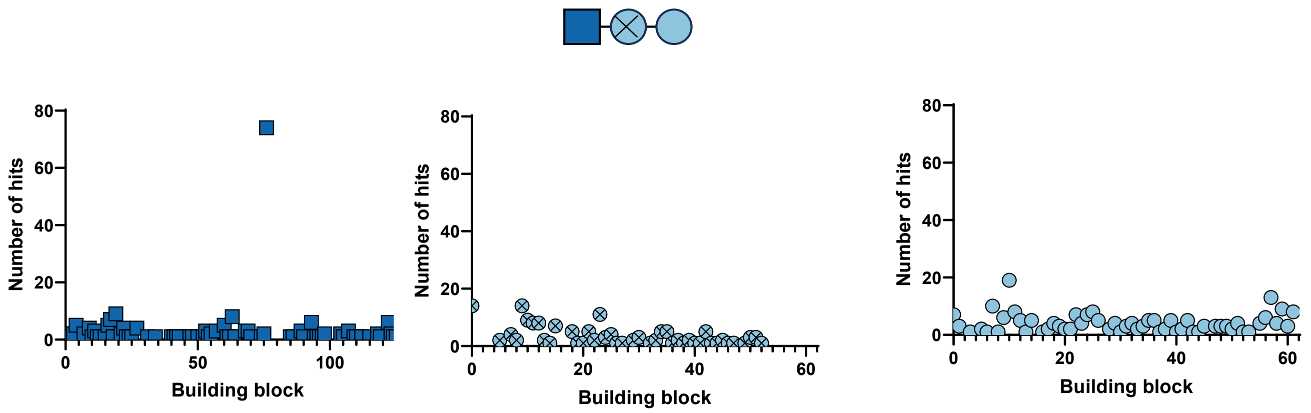

Supplementary Fig 24. Building block analysis after affinity selection with SEL 1 against CAIX.
The top ranked structures by EPIMETHEUS were taken and deconstructed into the respective building blocks.

8.2.3. Hit-Identification (LC-MS/MS)

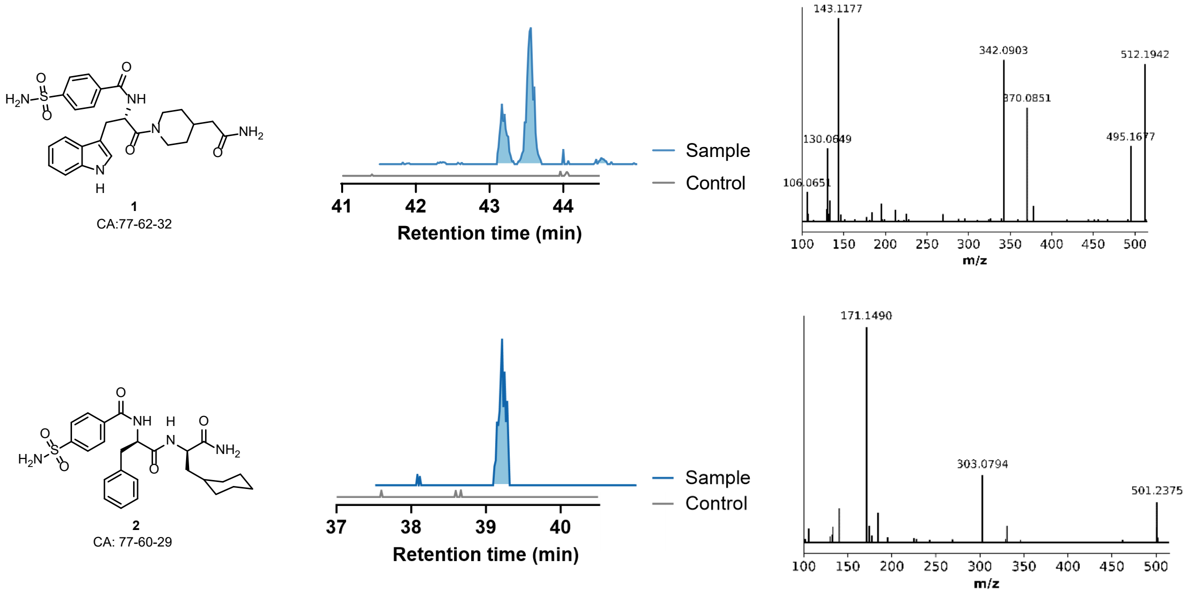

Supplementary Fig 25. Hit identified using COMET
Chemical structures of hit **1** and **2**, with the extracted ion chromatogram (EIC) and LC-MS/MS chromatogram from the affinity selection of SEL 1 against CAIX.

8.2.4. Hit re-synthesis

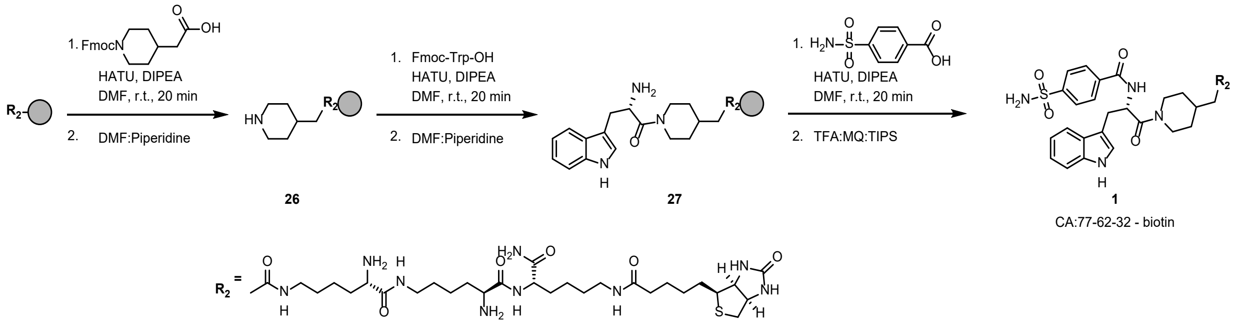

Product **1** was synthesized on a 50 µmol scale according to general procedure: Manual solid-phase synthesis (SPS) as described in section 3.3 by respectively coupling Fmoc-Lys(biotin)-OH and Boc-Lys(Fmoc)-OH (2x), Fmoc-Trp-OH, 1-Fmoc-4-piperidineacetic acid and 4-sulfomylbenzoic acid. The compound was cleaved of the resin by incubating at room temperature for 1 hour with a solution of TFA:H_2O:TIPS (92.5:5:2.5) and washed with TFA:H_2O:TIPS (92.5:5:2.5). The volume was reduced by evaporating the TFA solution with a N_2 stream. The crude mixture was purified by reverse phase column chromatography (C18, 00-45% MQ:MeCN) and product **1** was isolated in 3% yield (1.54 mg, 1.37 µmol, >95% purity). MS (ESI): m/z calculated for $C_{53}H_{79}N_{13}O_{10}S_2$ [M+H] = 1122,5587, found = 1122.60.

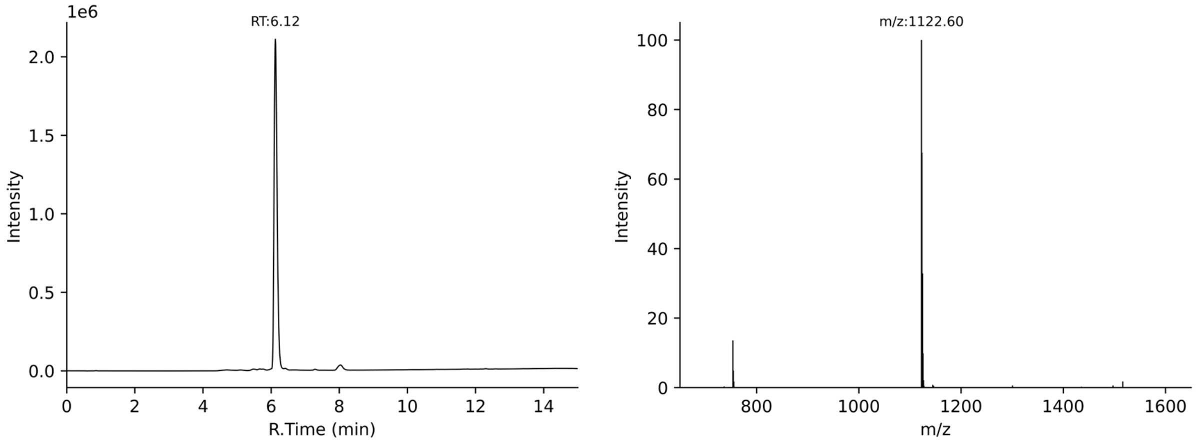

Supplementary Fig 26. LC-MS chromatogram from purified compound 1.

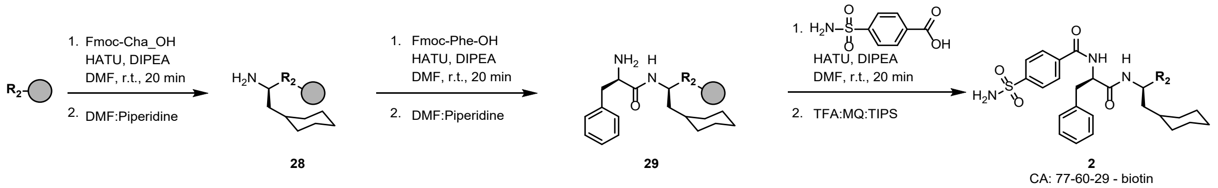

Product **2** was synthesized on a 50 µmol scale according to general procedure: Manual solid-phase synthesis (SPS) as described in section 3.3 by respectively coupling Fmoc-Lys(biotin)-OH and Boc-Lys(Fmoc)-OH (2x), Fmoc-Cha-OH, Fmoc-Phe-OH and 4-sulfomylbenzoic acid. The compound was cleaved of the resin by incubating at room temperature for 1 hour with a solution of TFA:H_2O:TIPS (92.5:5:2.5) and washed with TFA:H_2O:TIPS (92.5:5:2.5). The volume was reduced by evaporating the TFA solution with a N_2 stream. The crude mixture was purified by reverse phase column chromatography (C18, 00-45% MQ:MeCN) and product **2** was isolated in 1% yield (0.37 mg, 0.33 µmol, >95% purity). MS (ESI): m/z calculated for $C_{53}H_{82}N_{12}O_{10}S_2$ [M+H] = 1111,5791, found = 1111,65.

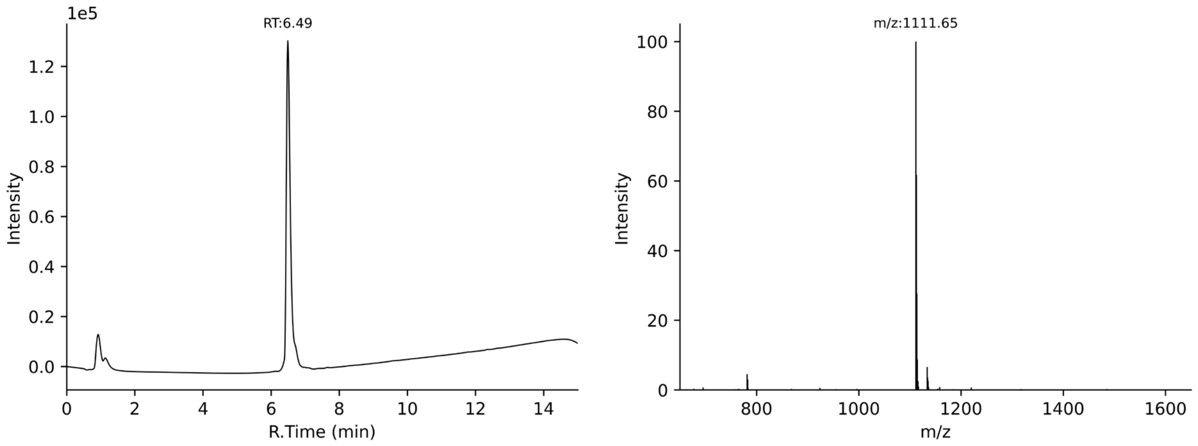

Supplementary Fig 27. LC-MS chromatogram from purified compound 2.

8.2.5. Hit validation: Biolayer interferometry

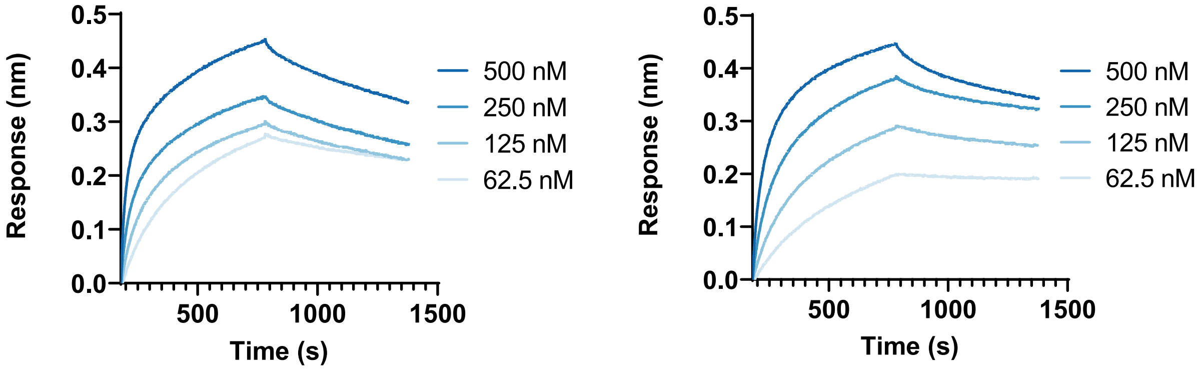

Supplementary Fig 28. Biolayer interferometry association and dissociation curves for compound 1 and 2.

The biolayer interferometry experiment was performed according to the general procedure describe in section 3.3

8.3. Screening of SEL 2 against CAIX

8.3.1. List of hit structures

Supplementary Table 6. Identified bindings for CAIX after AS-MS with SEL 2

SMILES
CC(C)C(NC(=O)c1ccc2c(c1)nc(-c1cccn1C)n2CCc1ccc(S(N)(=O)=O)cc1)C(N)=O
CC(C)C(NC(=O)c1ccc2c(c1)nc(-c1cccnc1)n2CCc1ccc(S(N)(=O)=O)cc1)C(N)=O
CC(C)C(NC(=O)c1ccc2c(c1)nc(-c1cncc3ccccc13)n2CCc1ccc(S(N)(=O)=O)cc1)C(N)=O
CC(C)CC(NC(=O)c1ccc2c(c1)nc(-c1cccc(F)n1)n2CCc1ccc(S(N)(=O)=O)cc1)C(N)=O
CC(C)CC(NC(=O)c1ccc2c(c1)nc(-c1cccn1C)n2CCc1ccc(S(N)(=O)=O)cc1)C(N)=O
CC(C)CC(NC(=O)c1ccc2c(c1)nc(-c1cccnc1)n2CCc1ccc(S(N)(=O)=O)cc1)C(N)=O
CCCCCC(NC(=O)c1ccc2c(c1)nc(-c1ccc(F)nc1F)n2CCc1ccc(S(N)(=O)=O)cc1)C(N)=O
CCOc1ccc(-c2nc3cc(C(=O)NC(Cc4ccco4)C(N)=O)ccc3n2CCc2ccc(S(N)(=O)=O)cc2)cn1
COc1cc(F)c(-c2nc3cc(C(=O)NC(CC(=O)O)C(N)=O)ccc3n2CCc2ccc(S(N)(=O)=O)cc2)c(F)c1
COc1cc(F)c(-c2nc3cc(C(=O)NC(CCC(=O)O)C(N)=O)ccc3n2CCc2ccc(S(N)(=O)=O)cc2)c(F)c1
COc1ccc(-c2nc3cc(C(=O)NC(CC(=O)O)C(N)=O)ccc3n2CCc2ccc(S(N)(=O)=O)cc2)cc1O
COc1ccc(-c2nc3cc(C(=O)NC4(C(N)=O)CCCC4)ccc3n2CCc2ccc(S(N)(=O)=O)cc2)cc1
COc1ccc(-c2nc3cc(C(=O)NC4(C(N)=O)COC4)ccc3n2CCc2ccc(S(N)(=O)=O)cc2)cc1O
Cc1ccc(Cl)c(-c2nc3cc(C(=O)NC(CC(=O)O)C(N)=O)ccc3n2CCc2ccc(S(N)(=O)=O)cc2)c1F
Cc1ccc(Cl)c(-c2nc3cc(C(=O)NC(CCC(=O)O)C(N)=O)ccc3n2CCc2ccc(S(N)(=O)=O)cc2)c1F
Cn1cccc1-c1nc2cc(C(=O)NC(C(N)=O)C3CCCCC3)ccc2n1CCc1ccc(S(N)(=O)=O)cc1
Cn1cccc1-c1nc2cc(C(=O)NC(CC(N)=O)Cc3ccc(F)c(F)c3)ccc2n1CCc1ccc(S(N)(=O)=O)cc1
Cn1cccc1-c1nc2cc(C(=O)NC(Cc3c[nH]c4ccccc34)C(N)=O)ccc2n1CCc1ccc(S(N)(=O)=O)cc1
Cn1cccc1-c1nc2cc(C(=O)NC(Cc3ccc(Br)cc3)C(N)=O)ccc2n1CCc1ccc(S(N)(=O)=O)cc1
Cn1cccc1-c1nc2cc(C(=O)NC(Cc3ccc(O)cc3)C(N)=O)ccc2n1CCc1ccc(S(N)(=O)=O)cc1
Cn1cccc1-c1nc2cc(C(=O)NC(Cc3ccccc3)C(N)=O)ccc2n1CCc1ccc(S(N)(=O)=O)cc1
Cn1cccc1-c1nc2cc(C(=O)NCC(N)=O)ccc2n1CCc1ccc(S(N)(=O)=O)cc1
Cn1cccc1-c1nc2cc(C(=O)Nc3ccc(C(N)=O)cc3)ccc2n1CCc1ccc(S(N)(=O)=O)cc1
N#Cc1cc(F)c(-c2nc3cc(C(=O)NCC4CCC(C(N)=O)CC4)ccc3n2CCc2ccc(S(N)(=O)=O)cc2)c(F)c1
NC(=O)C(CC(=O)O)NC(=O)c1ccc2c(c1)nc(-c1ccc(Br)cc1)n2CCc1ccc(S(N)(=O)=O)cc1
NC(=O)C(CC1CCCC1)NC(=O)c1ccc2c(c1)nc(-c1cccc(F)n1)n2CCc1ccc(S(N)(=O)=O)cc1
NC(=O)C(CCC(=O)O)NC(=O)c1ccc2c(c1)nc(-c1cccc(Cl)c1F)n2CCc1ccc(S(N)(=O)=O)cc1
NC(=O)C(CCC(=O)O)NC(=O)c1ccc2c(c1)nc(C#Cc1ccccc1)n2CCc1ccc(S(N)(=O)=O)cc1
NC(=O)C1CC(F)CN1C(=O)c1ccc2c(c1)nc(-c1ncco1)n2CCc1ccc(S(N)(=O)=O)cc1
NC(=O)CC(NC(=O)c1ccc2c(c1)nc(-c1cccc(F)n1)n2CCc1ccc(S(N)(=O)=O)cc1)C(N)=O

List of structures identified after performing AS-MS in duplicates on CAIX using SEL 3. Using COMET filter with a least matching 1 out of the 5 biggest peaks and 5 ppm accuracy, 30 unique structures containing building block **AM42** were identified.

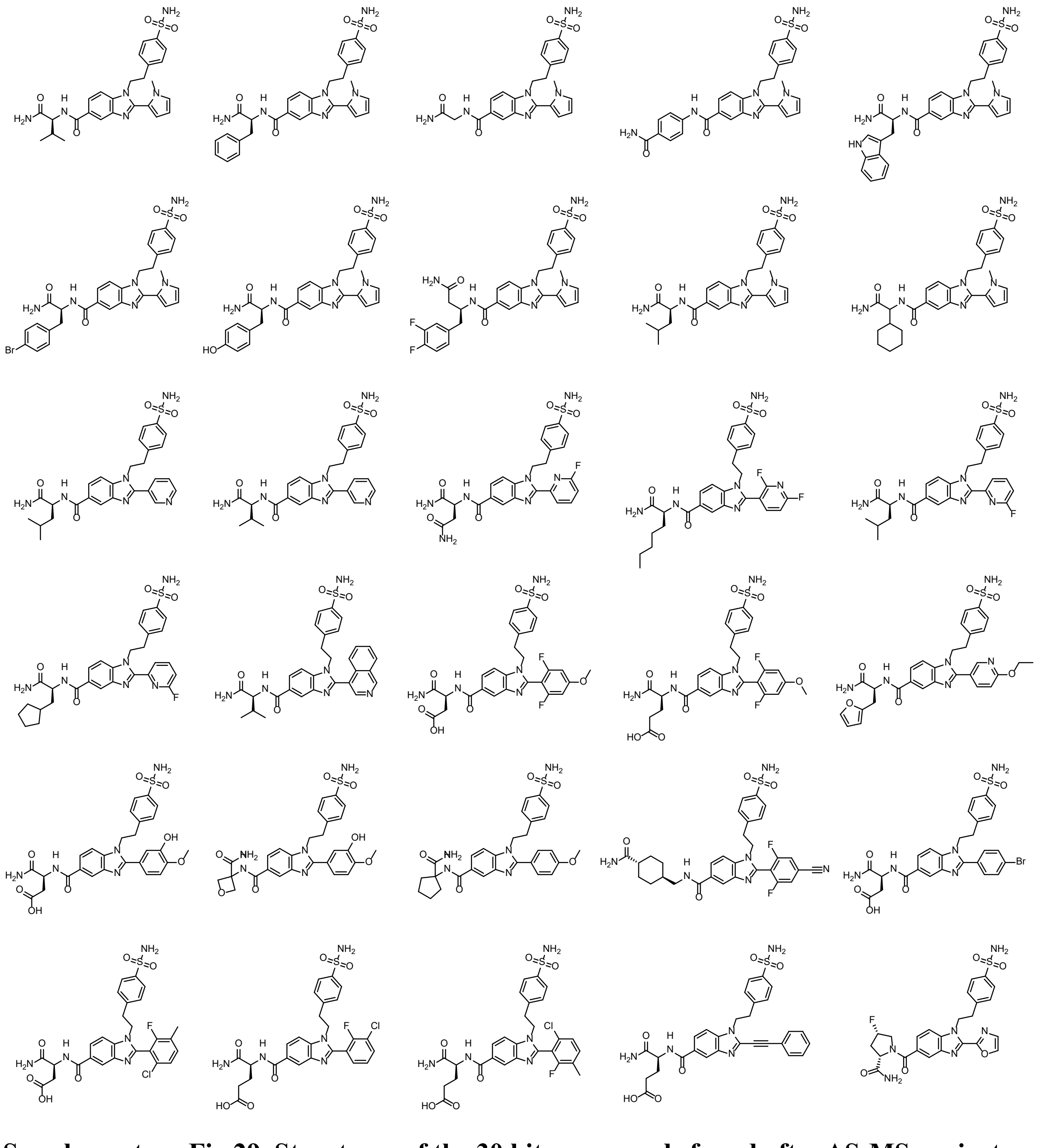

Supplementary Fig 29. Structures of the 30 hit compounds found after AS-MS against CAIX with SEL 2.

8.3.2. Enrichment plots

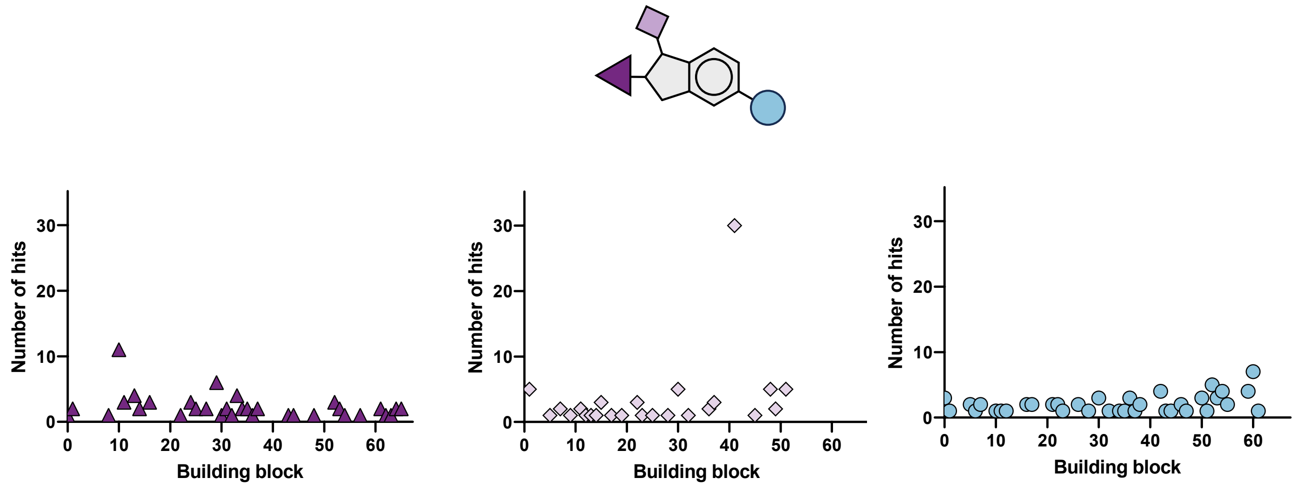

Supplementary Fig 30. Building block analysis after affinity selection with SEL 2 against CAIX.
The top ranked structures by EPIMETHEUS were taken and deconstructed into the respective building blocks.

8.3.3. Hit-Identification (LC-MS/MS)

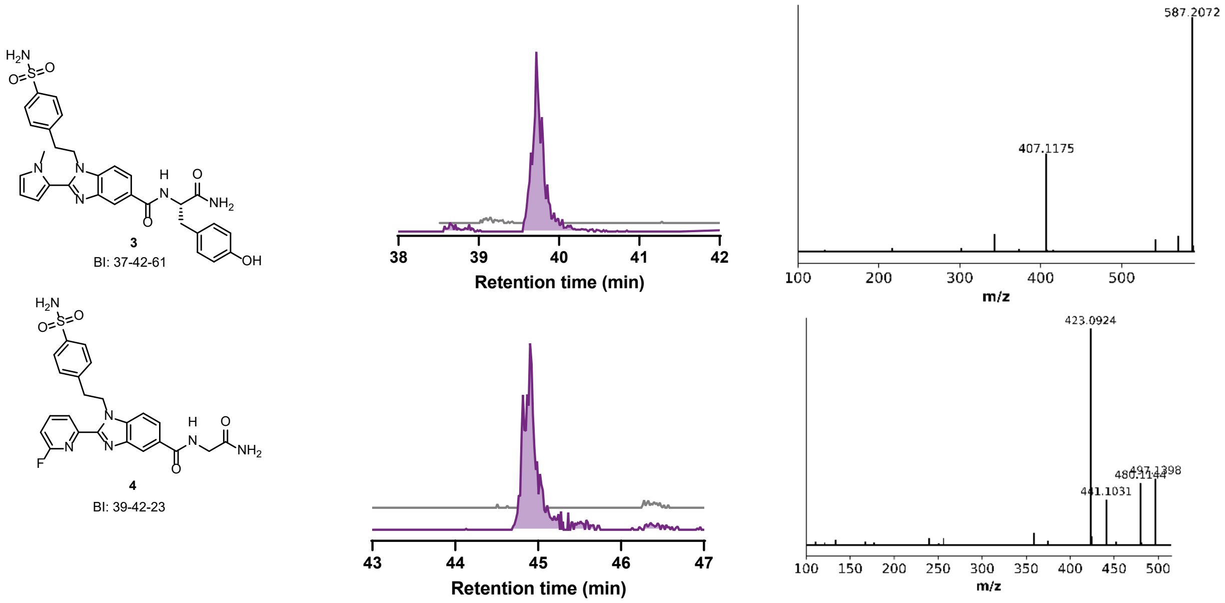

Supplementary Fig 31. Hit identified using COMET
Chemical structures of hit **3** and **4**, with the extracted ion chromatogram (EIC) and LC-MS/MS chromatogram from the affinity selection of SEL 2 against CAIX.

8.3.4. Synthesis of hit structures

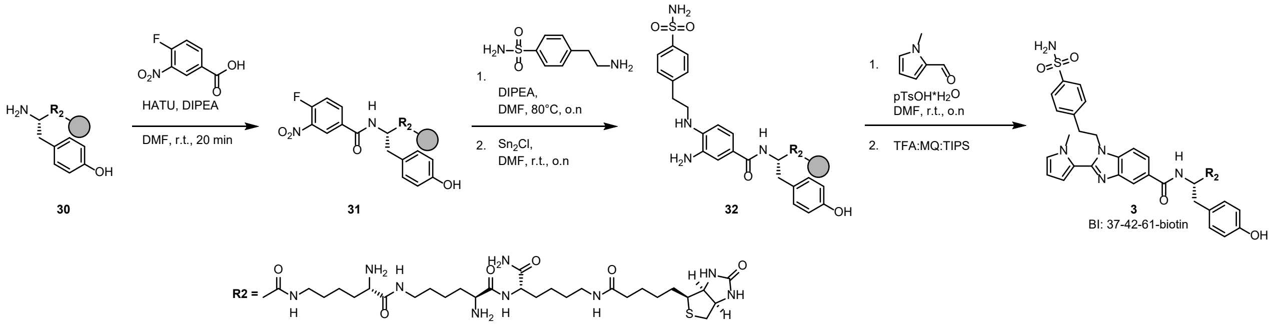

Starting material **30** was synthesized on a 50 µmol scale according to general procedure: Manual solid-phase synthesis (SPS) as described in section 3.3 by respectively coupling Fmoc-Lys(biotin)-OH and Boc-Lys(Fmoc)-OH (2x), Fmoc-Tyr-OH. A solution of 4-fluoro-3-nitrobenzoic acid (27.8 mg, 450 µmol, 3.0 eq), 0.4M HATU (372.5 µL, 149 mmol, 2.98 eq) and DIPEA (78.4 µL, 450 mmol, 9.0 eq) in DMF was added to the resin and reacted for 20 min. The resin was washed with DMF (5 x 2 mL). The resin was transferred to an eppendorf tube. 4-(2-aminoethyl)benzenesulfonamide (100 mg, 500 µmol, 10 eq.) and DIPEA (87 µL, 500 µmol, 10 eq.) in DMF (150µL, 0.4 M). The mixture was shaken at 1 x g overnight at 80 °C. The resin was washed with DMF (5 x 2 mL) and DCM (5 x 2 mL). A solution of 1.0 M SnCl2 (592 mg, 3.125 mmol, 62.5 eq.) in DMF was added to the resin (50 µmol). The mixture was incubated on r.t. overnight, whereafter the resin was washed with DMF in 50% MQ (5x, 2 mL), with DMF (5x, 2 mL) and DCM (5x, 2 mL).The resin was suspended in a solution of *N*-Methyl-2-pyrrolecarboxaldehyde (0.25 M in DMF (105 µL), 250 µmol, 5 eq.) and *p*-TsOH•H_2O (47.6 mg, 250 µmol, 5 eq.). The mixture was incubated on r.t. overnight on 1 x g. After incubation, the resin was washed DMF (5x, 2 mL) and DCM (5x, 2 mL). The compound was cleaved of the resin by incubating at room temperature for 1 hour with a solution of TFA:H_2O:TIPS (92.5:5:2.5) and washed with TFA:H_2O:TIPS (92.5:5:2.5). The volume was reduced by evaporating the TFA solution with a N_2 stream. The crude mixture was purified by reverse phase column chromatography (C18, 00-45% MQ:MeCN) and product **3** was isolated in 6% yield (3.7 mg, 3.1 µmol, 93% purity). MS (ESI): m/z calculated for $C_{58}H_{80}N_{14}O_{10}S_2$ [M+H] = 1197.6353, found = 1197.70.

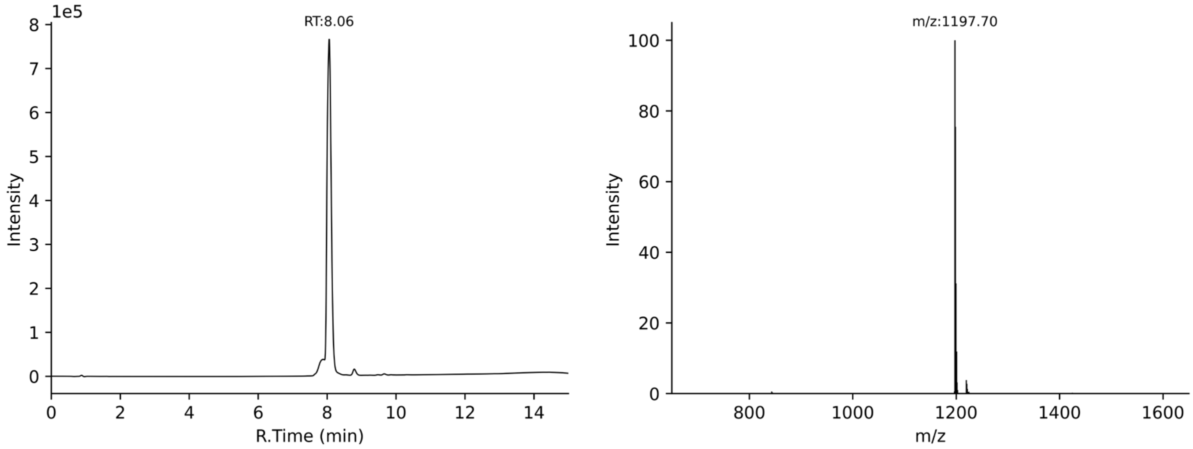

Supplementary Fig 32. LC-MS chromatogram from purified compound 3.

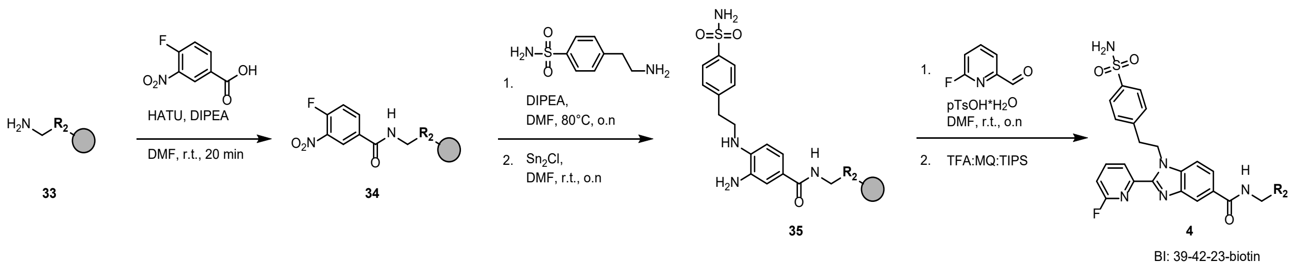

Starting material **33** was synthesized on a 50 µmol scale according to general procedure: Manual solid-phase synthesis (SPS) as described in section 3.3 by respectively coupling Fmoc-Lys(biotin)-OH and Boc-Lys(Fmoc)-OH (2x), Fmoc-Gly-OH. A solution of 4-fluoro-3-nitrobenzoic acid (27.8 mg, 450 µmol, 3.0 eq), 0.4M HATU (372.5 µL, 149 mmol, 2.98 eq) and DIPEA (78.4 µL, 450 mmol, 9.0 eq) in DMF was added to the resin and reacted for 20 min. The resin was washed with DMF (5 x 2 mL). The resin was transferred to an eppendorf tube. 4-(2-aminoethyl)benzenesulfonamide (100 mg, 500 µmol, 10 eq.) and DIPEA (87 µL, 500 µmol, 10 eq.) in DMF (150µL, 0.4 M). The mixture was shaken at 1 x g overnight at 80 °C. The resin was washed with DMF (5 x 2 mL) and DCM (5 x 2 mL). A solution of 1.0 M SnCl2 (592 mg, 3.125 mmol, 62.5 eq.) in DMF was added to the resin (50 µmol). The mixture was incubated on r.t. overnight, whereafter the resin was washed with DMF in 50% MQ (5x, 2 mL), with DMF (5x, 2 mL) and DCM (5x, 2 mL). The resin was suspended in a solution of *6*-Fluoropicolinaldehyde (0.25 M in DMF (105 µL), 250 µmol, 5 eq.) and *p*-TsOH•H_2O (47.6 mg, 250 µmol, 5 eq.). The mixture was incubated on r.t. overnight on 1 x g. After incubation, the resin was washed DMF (5x, 2 mL) and DCM (5x, 2 mL). The compound was cleaved of the resin by incubating at room temperature for 1 hour with a solution of TFA:H_2O:TIPS (92.5:5:2.5) and washed with TFA:H_2O:TIPS (92.5:5:2.5). The volume was reduced by evaporating the TFA solution with a N_2 stream. The crude mixture was purified by reverse phase column chromatography (C18, 00-45% MQ:MeCN) and product **4** was isolated in 1% yield (0.70 mg, 0.63 µmol, 89% purity). MS (ESI): m/z calculated for $C_{51}H_{71}FN_{14}O_9S_2$ [M+H] = 1107.5684, found = 1107.65.

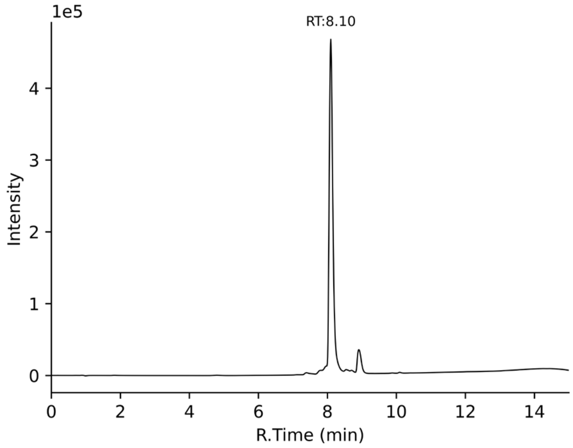

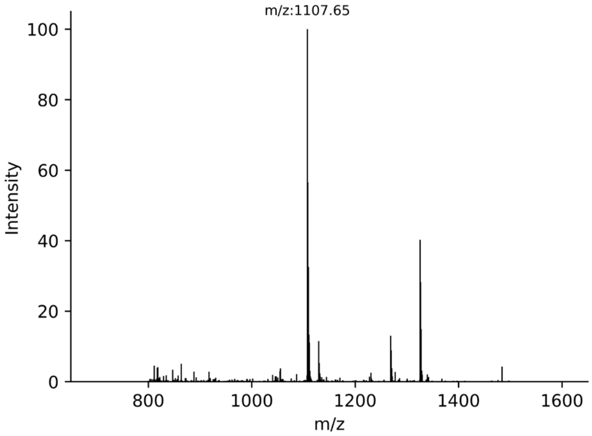

Supplementary Fig 33. LC-MS chromatogram from purified compound 4.

8.3.5. Hit validation: Biolayer interferometry

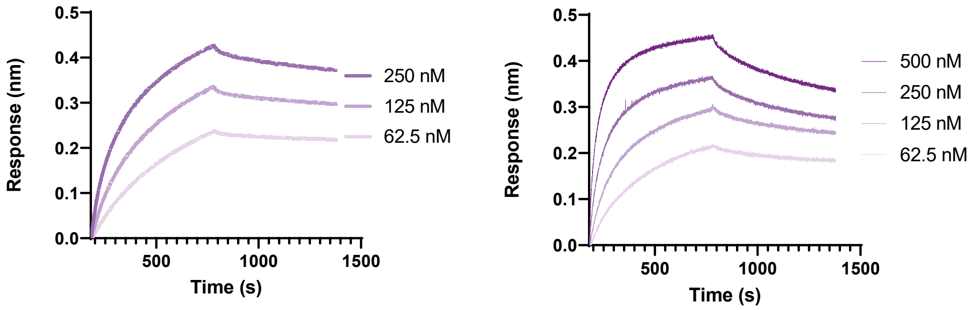

Supplementary Fig 34. Biolayer interferometry association and dissociation curves for compound 3 and 4.
The biolayer interferometry experiment was performed according to the general procedure describe in section 3.3

8.4. Screening of SEL 3 against CAIX

8.4.1. List of hit structures

Supplementary Table 7. Identified bindings for CAIX after AS-MS with SEL 3

SMILES
O=C(N)C(N(C(=O)C1=CC=C(F)C=C1C=2C=CC(=CC2)S(=O)(=O)N)C)C(C)C
O=C(N)C(NC(=O)C(NC(=O)C=1C=CC=CC1)CC2=CC=C(C=C2)C=3C=CC(=CC3)S(=O)(=O)N)C4CC4
O=C(N)C(NC(=O)C(NC(=O)C=1C=CC=CC1)CC2=CC=C(C=C2)C=3C=CC(=CC3)S(=O)(=O)N)CC4=NC =CC=C4
O=C(N)C(NC(=O)C(NC(=O)C=1C=CC=CC1)CC2=CC=C(C=C2)C=3C=CC(=CC3)S(=O)(=O)N)CCCCN
O=C(N)C(NC(=O)C(NC(=O)C=1C=CC=CC1)CC2=CC=C(C=C2)C=3C=CC(=CC3)S(=O)(=O)N)CCCNC(=N)N
O=C(N)C(NC(=O)C1(C2=CC=C(C=C2)C=3C=CC(=CC3)S(=O)(=O)N)CC1)C(C)C
O=C(N)C(NC(=O)C1(C2=CC=C(C=C2)C=3C=CC(=CC3)S(=O)(=O)N)CC1)CC4CCC4
O=C(N)C(NC(=O)C1(C2=CC=C(C=C2)C=3C=CC(=CC3)S(=O)(=O)N)CC1)CO
O=C(N)C(NC(=O)C1=CC=C(F)C=C1C=2C=CC(=CC2)S(=O)(=O)N)C3CCCCC3
O=C(N)C(NC(=O)C1=CC=C(F)C=C1C=2C=CC(=CC2)S(=O)(=O)N)CCCCC
O=C(N)C(NC(=O)C1=CC=C2C(C=CC=C2C3=CC=C(C=C3)S(=O)(=O)N)=C1)CC4=NC=CC=C4
O=C(N)C(NC(=O)C1=CC=C2C(C=CC=C2C3=CC=C(C=C3)S(=O)(=O)N)=C1)CC4CCC4
O=C(N)C(NC(=O)C1=CC=C2C(C=CC=C2C3=CC=C(C=C3)S(=O)(=O)N)=C1)CC=4N=CNC4
O=C(N)C(NC(=O)C1=CC=C2C(C=CC=C2C3=CC=C(C=C3)S(=O)(=O)N)=C1)CCCCN
O=C(N)C(NC(=O)C1=CC=C2C(C=CC=C2C3=CC=C(C=C3)S(=O)(=O)N)=C1)CCCNC(=N)N
O=C(N)C(NC(=O)C1=CC=C2C(C=CC=C2C3=CC=C(C=C3)S(=O)(=O)N)=C1)CO
O=C(N)C(NC(=O)C=1C=CC(=CC1)C2=CC=C(C=C2)S(=O)(=O)N)C(O)C
O=C(N)C(NC(=O)C=1C=CC=C(C1)C=2C=CC(=CC2)S(=O)(=O)N)CC=3N=CNC3
O=C(N)C(NC(=O)C=1C=CC=C(C1)C=2C=CC(=CC2)S(=O)(=O)N)CCCCN
O=C(N)C(NC(=O)C=1C=CC=C(C1)C=2C=CC(=CC2)S(=O)(=O)N)CCCNC(=N)N
O=C(N)C(NC(=O)CCC1=CC=C(C=C1)C=2C=CC(=CC2)S(=O)(=O)N)CC3CCC3
O=C(N)C(NC(=O)CCC1=CC=C(C=C1)C=2C=CC(=CC2)S(=O)(=O)N)CC=3N=CNC3
O=C(N)C1(NC(=O)C(NC(=O)C=2C=CC=CC2)CC3=CC=C(C=C3)C=4C=CC(=CC4)S(=O)(=O)N)CCN(C C=5C=CC=CC5)CC1

O=C(N)C1(NC(=O)C2(C3=CC=C(C=C3)C=4C=CC(=CC4)S(=O)(=O)N)CC2)CCCC1

O=C(N)C1(NC(=O)C2=CC=C3C(C=CC=C3C4=CC=C(C=C4)S(=O)(=O)N)=C2)CCN(CC=5C=CC=CC5)CC1

O=C(N)C1CCC(CNC(=O)C2(C3=CC=C(C=C3)C=4C=CC(=CC4)S(=O)(=O)N)CC2)CC1

O=C(N)C1CCC(CNC(=O)C2=CC=C(F)C=C2C=3C=CC(=CC3)S(=O)(=O)N)CC1

O=C(N)C1CCC(NC(=O)CCC2=CC=C(C=C2)C=3C=CC(=CC3)S(=O)(=O)N)C1

O=C(N)C1N(C(=O)C(NC(=O)C=2C=CC=CC2)CC3=CC=C(C=C3)C=4C=CC(=CC4)S(=O)(=O)N)CC5(CC5)C1

O=C(N)C1N(C(=O)C2(C3=CC=C(C=C3)C=4C=CC(=CC4)S(=O)(=O)N)CC2)CC5(CC5)C1

O=C(N)C1N(CCC1)C2CCN(C(=O)C(NC(=O)C=3C=CC=CC3)CC4=CC=C(C=C4)C=5C=CC(=CC5)S(=O)(=O)N)CC2

O=C(N)CC(NC(=O)C(NC(=O)C=1C=CC=CC1)CC2=CC=C(C=C2)C=3C=CC(=CC3)S(=O)(=O)N)C(=O)N

O=C(N)CC(NC(=O)C1(C2=CC=C(C=C2)C=3C=CC(=CC3)S(=O)(=O)N)CC1)C(=O)N

O=C(N)CC(NC(=O)C1(C2=CC=C(C=C2)C=3C=CC(=CC3)S(=O)(=O)N)CC1)CC4=CC=C(F)C(F)=C4

O=C(N)CC(NC(=O)C=1C=CC(=CC1)C2=CC=C(C=C2)S(=O)(=O)N)CC3=CC=C(F)C(F)=C3

O=C(N)CCC(NC(=O)C(NC(=O)C=1C=CC=CC1)CC2=CC=C(C=C2)C=3C=CC(=CC3)S(=O)(=O)N)C(=O)N

O=C(N)CCC(NC(=O)C1=CC=C2C(C=CC=C2C3=CC=C(C=C3)S(=O)(=O)N)=C1)C(=O)N

O=C(N)CN(C(=O)C=1C=CC=C(C1)C=2C=CC(=CC2)S(=O)(=O)N)C

O=C(N)CN1CCN(C(=O)C(NC(=O)C=2C=CC=CC2)CC3=CC=C(C=C3)C=4C=CC(=CC4)S(=O)(=O)N)CC1

O=C(N)COCC1N(C(=O)C(NC(=O)C=2C=CC=CC2)CC3=CC=C(C=C3)C=4C=CC(=CC4)S(=O)(=O)N)CCC1

O=C(N)COCC1N(C(=O)C2=CC=C3C(C=CC=C3C4=CC=C(C=C4)S(=O)(=O)N)=C2)CCC1

O=C(N)COCC1N(C(=O)CCC2=CC=C(C=C2)C=3C=CC(=CC3)S(=O)(=O)N)CCC1

O=C(O)CC(NC(=O)C(NC(=O)C=1C=CC=CC1)CC2=CC=C(C=C2)C=3C=CC(=CC3)S(=O)(=O)N)C(=O)N

O=C(O)CC(NC(=O)C1(C2=CC=C(C=C2)C=3C=CC(=CC3)S(=O)(=O)N)CC1)C(=O)N

O=C(O)CC(NC(=O)C1=CC=C2C(C=CC=C2C3=CC=C(C=C3)S(=O)(=O)N)=C1)C(=O)N

O=C(O)CCC(NC(=O)C1(C2=CC=C(C=C2)C=3C=CC(=CC3)S(=O)(=O)N)CC1)C(=O)N

O=C(O)CCC(NC(=O)C1=CC=C2C(C=CC=C2C3=CC=C(C=C3)S(=O)(=O)N)=C1)C(=O)N

List of hit structures identified after performing AS-MS in duplicates on CAIX using SEL 3. Using COMET filter with a least matching 1 out of the 5 biggest peaks and 5 ppm accuracy, 47 unique structures containing building block **BA53** were identified.

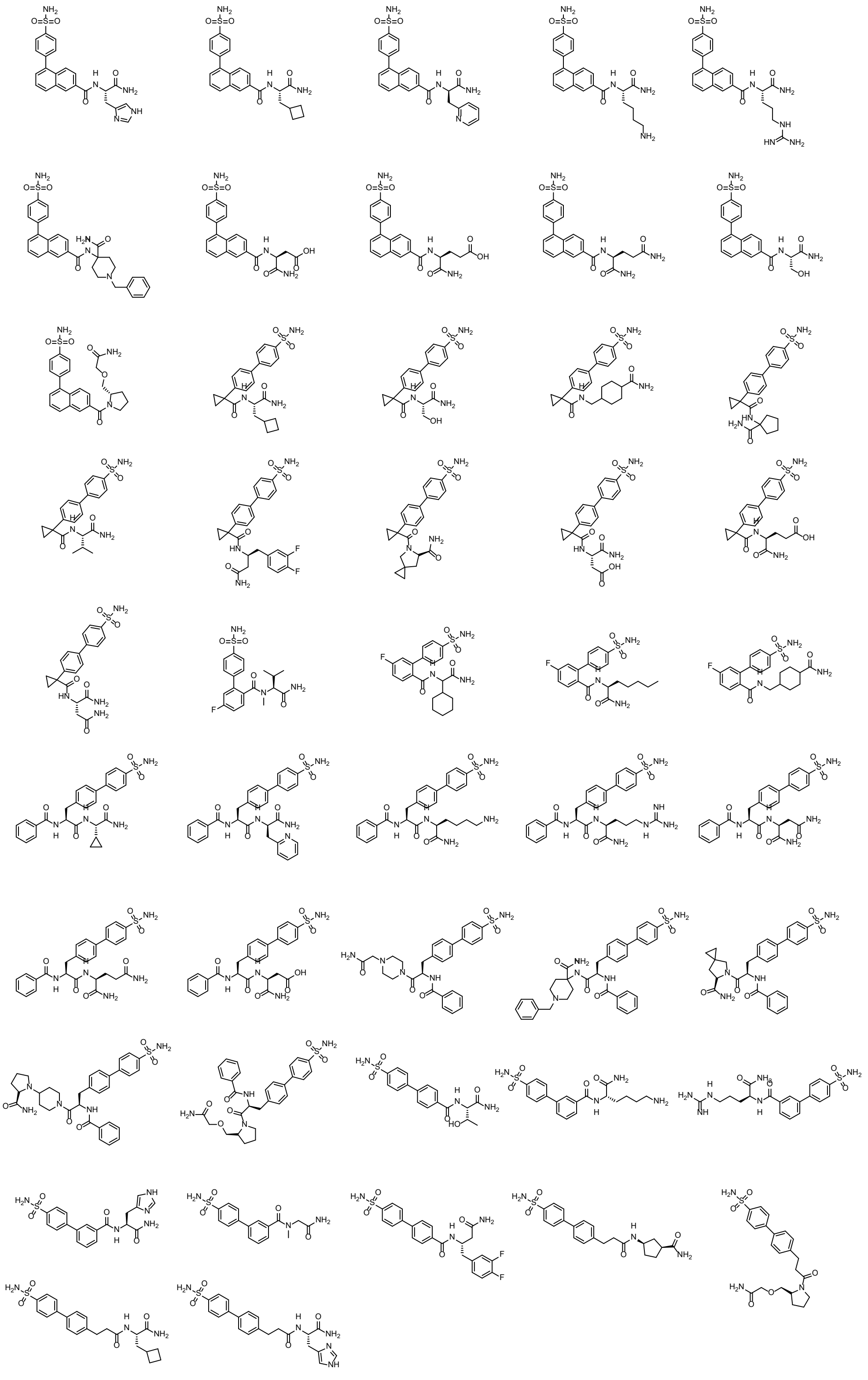

Supplementary Fig 35. Structures of the 47 hit compounds found after AS-MS against CAIX with SEL 3.

8.4.2. Enrichment plots

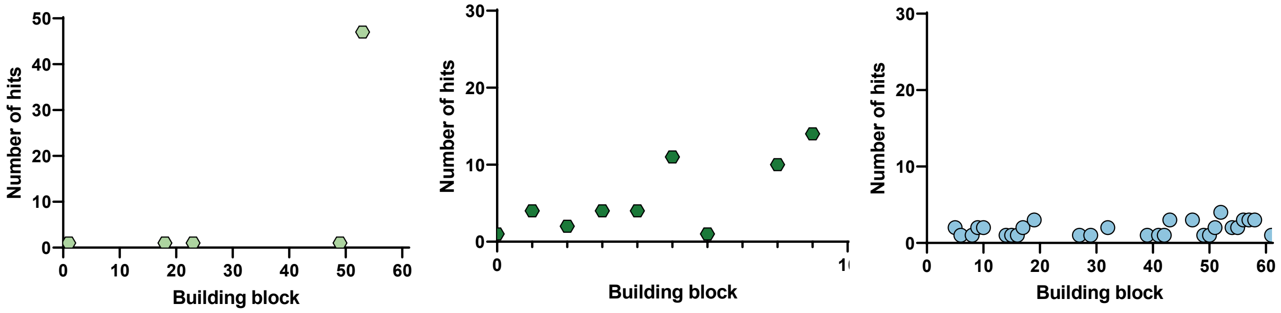

Supplementary Fig 36. Building block analysis after affinity selection with SEL 3 against CAIX.
The top ranked structures by EPIMETHEUS were taken and deconstructed into the respective building blocks.

8.4.3. Hit-Identification (LC-MS/MS)

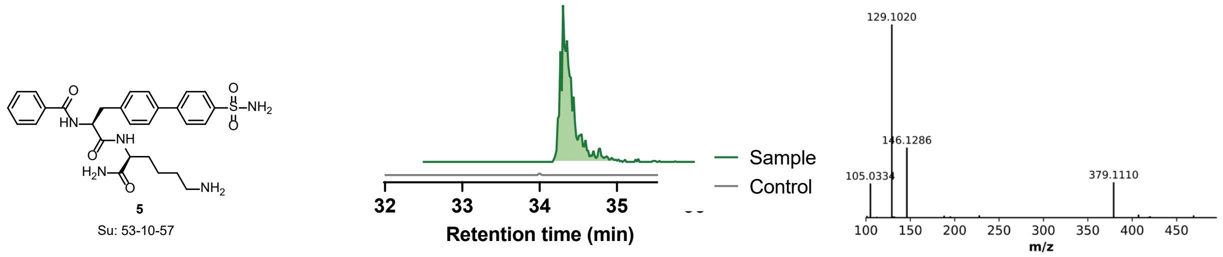

Supplementary Fig 37. Hit identified using COMET
Chemical structures of hit **5**, with the extracted ion chromatogram (EIC) and LC-MS/MS chromatogram from the affinity selection of SEL 3 against CAIX.

8.4.4. Synthesis of hit structures

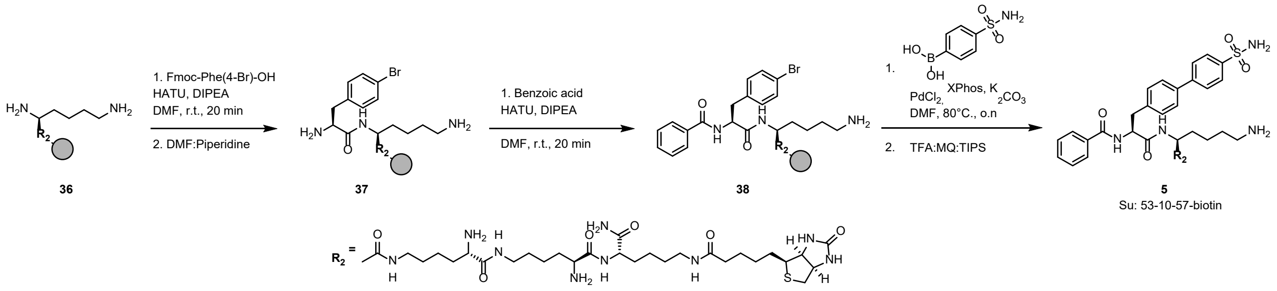

Starting material **36** was synthesized on a 50 µmol scale according to general procedure: Manual solid-phase synthesis (SPS) as described in section 3.3 by respectively coupling Fmoc-Lys(biotin)-OH and Boc-Lys(Fmoc)-OH (2x), Fmoc-Lys-OH and benzoic acid. 4-(tert-Butylaminosulphonyl)benzeneboronic acid (25.7 mg, 100 µmol, 2.0 eq.), K_2CO_3 (13.8 mg, 100 µmol, 2.0 eq.), $PdCl_2$ (0.89 mg, 5 µmol, 10 mol%), XPhos (4.77 mg, 10 µmol, 20 mol%) and DMF (1 mL) were added to the resin (50.0 µmol). The reaction was stirred at 1 x g at 80 °C for 17h. The resin was washed with DMF (5 x 2 mL) and DCM (5 x 2 mL). The compound was cleaved of the resin by incubating at room temperature for 1 hour with a solution of TFA:H_2O:TIPS (92.5:5:2.5) and washed with TFA:H_2O:TIPS (92.5:5:2.5). The volume was

reduced by evaporating the TFA solution with a N_2 stream. The crude mixture was purified by reverse phase column chromatography (C18, 00-45% MQ:MeCN) and product **5** was isolated in 1% yield (0.8 mg, 0.69 µmol, 95% purity). MS (ESI): m/z calculated for $C_{56}H_{83}N_{13}O_{10}S_2$ [M+H] =1162.5900, found =1162.60.

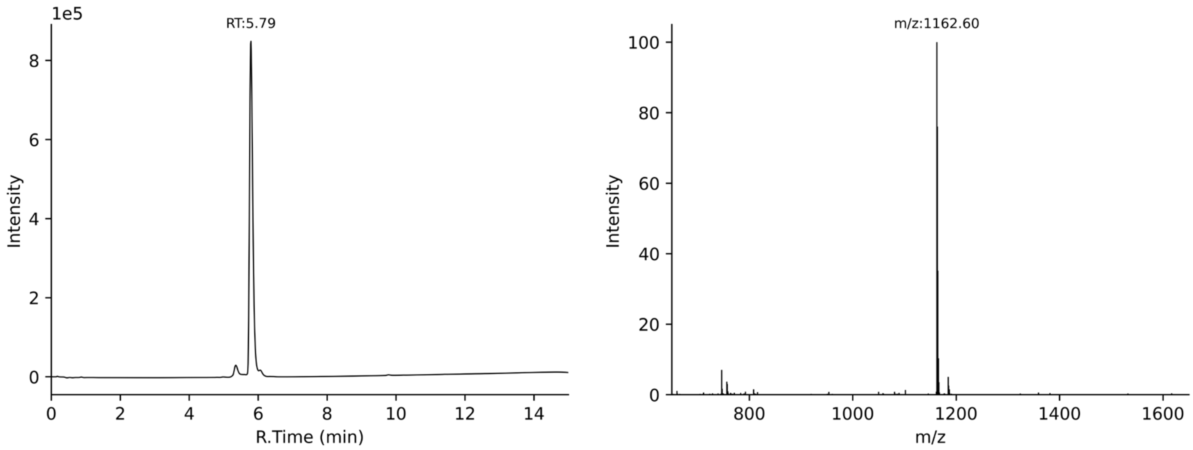

Supplementary Fig 38. LC-MS chromatogram from purified compound 5.

8.4.5. Hit validation: Biolayer interferometry

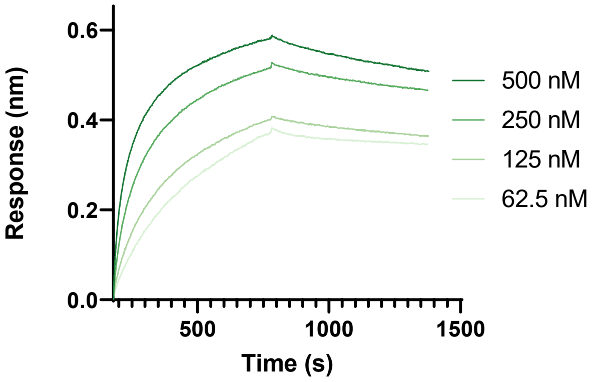

Supplementary Fig 39. Biolayer interferometry association and dissociation curves for compound 5

The biolayer interferometry experiment was performed according to the general procedure describe in section 3.3

9. Affinity selection against FEN1

9.1. Procedure

Dynabeads™ His-Tag Isolation and Pulldown (25 µL of 40 mg/mL stock per well, measured in duplicates) were washed with 3x 1 mL 50 mM HEPES, 100 mM KCl, 5 mM $MgCl_2$, 1 mM DTT, 10% FBS, 0.02% Tween-20, pH = 7.5. The beads were incubated with FEN1 (100 µL, 1.5 µM per well) in 50 mM HEPES, 100 mM KCl, 5 mM $MgCl_2$, 1 mM DTT, 10% FBS, 0.02% Tween-20, pH = 7.5 for 1h at 4°C. The beads were washed 2x 1 mL 50 mM HEPES, 100 mM KCl, 5 mM $MgCl_2$, 1 mM DTT, 10% FBS, 0.02% Tween-20, 20 mM imidazole, pH = 7.5 and 1x 1 mL 50 mM HEPES, 100 mM KCl, 5 mM $MgCl_2$, 1 mM DTT, 10% FBS, 0.02% Tween-20, pH = 7.5 before incubating with the library (100 µL, 100 fmol/member per well) in 50 mM HEPES, 100 mM KCl, 5 mM $MgCl_2$ 1 mM DTT, 10% FBS, 20 mM imidazole, pH = 7.5 for 1h at 4°C. The beads were washed with 5x 1 mL of 50 mM HEPES, 100 mM KCl, 5 mM $MgCl_2$, 1 mM DTT, pH = 7.5 and subsequently eluted with 2x MeCN:MQ (1:1) 0.1%FA (100 µL per well).

Sample preparation after AS: Samples from the affinity selection procedure were lyophilized and resuspended in 50 µL MQ 0.1%FA. The StageTips were prepared as described by Rappsilber et al. using C18 material from Empore SPE 47 mm discs (66883-U, Merck)[1]. The StageTips were pre-conditioned with 200 µL MeOH, 200 µL of 0.1% (v/v) FA in MeCN and 200 µL of 0.1% (v/v) FA in MQ, respectively by centrifuging for 3 min at 300 rcf. The samples were then loaded on the StageTips and washed with 200 µL of 0.1% (v/v) FA in MQ. Compounds were eluted by adding 200 µL of 0.1% (v/v) FA in MeCN:MQ (7:3). The samples were lyophilized before resuspending in 10 µL 0.1% (v/v) FA in UPLC-MS grade water. The samples were centrifuged for 5 min at 21,000 x g. Afterwards, 9 µL was transferred to a LC-MS vial and 8 µL was injected into the LC-MS/MS system.

9.2. Screening of SEL 1 against FEN1

9.2.1. Hit-Identification (LC-MS/MS)

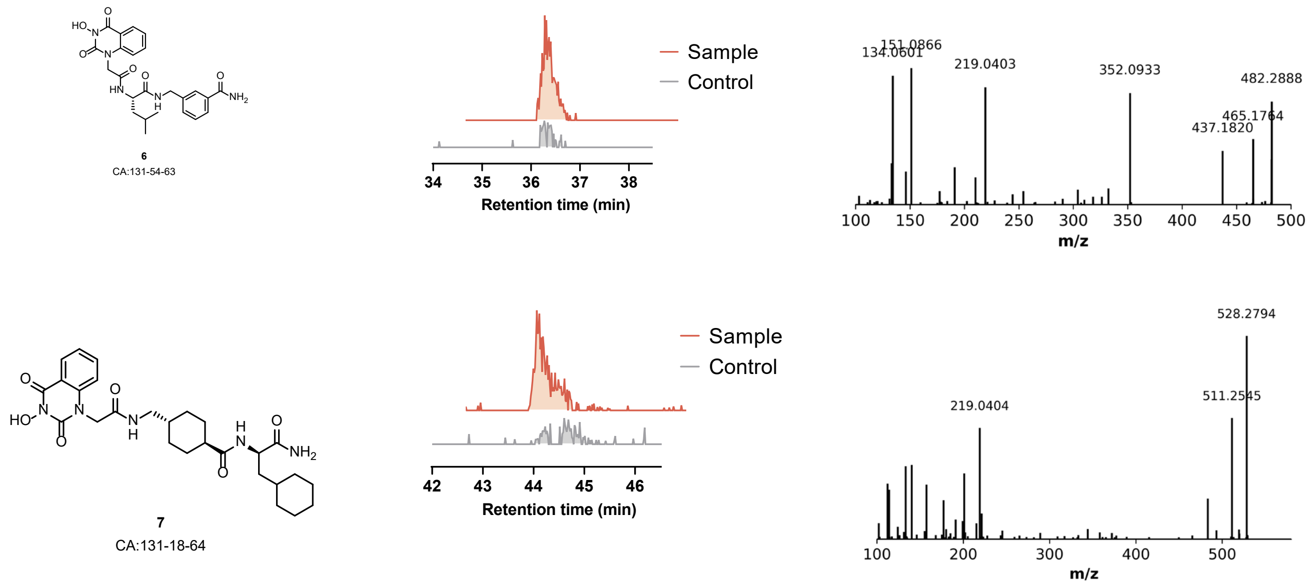

Supplementary Fig 40. Hit identified using COMET

Chemical structures of compounds **6** and **7**, with the extracted ion chromatogram (EIC) and LC-MS/MS chromatogram from the affinity selection of SEL 4 against FEN1.

9.2.2. Synthesis of hit structures

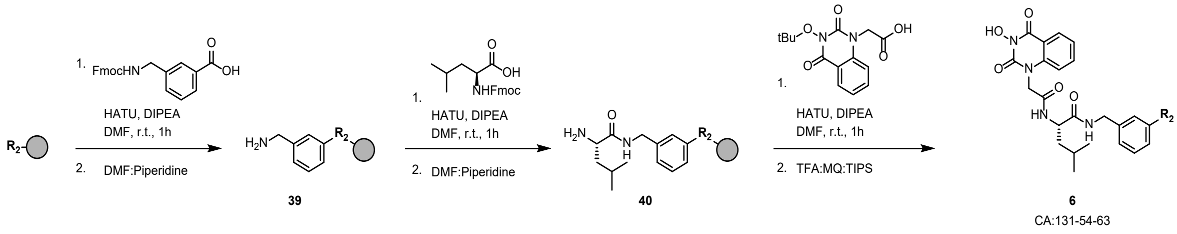

Product **6** was synthesized on a 50 µmol scale according to general procedure: Manual solid-phase synthesis (SPS) as described in section 3.3 by respectively coupling Fmoc-3-(aminomethyl)benzoic acid, Fmoc-Leu-OH and CA131. The compound was cleaved of the resin by incubating at room temperature for 1 hour with a solution of TFA:H_2O:TIPS (92.5:5:2.5) and washed with TFA:H_2O:TIPS (92.5:5:2.5). The volume was reduced by evaporating the TFA solution with a N_2 stream. The crude mixture was purified by reverse phase column chromatography (C18, 00-45% MQ:MeCN, 0.1% TFA) and product **6** was isolated in 26% yield (6.3 mg, 13 µmol, >95% purity). **^{1}H NMR** (400 MHz, DMSO) δ 10.87 (s, 1H), 8.72 – 8.47 (m, 2H), 8.08 (dd, *J* = 7.9, 1.6 Hz, 1H), 7.93 (s, 1H), 7.80 – 7.70 (m, 2H), 7.66 (ddd, *J* = 8.7, 7.3, 1.7 Hz, 1H), 7.41 – 7.27 (m, 4H), 7.20 (d, *J* = 8.5 Hz, 1H), 4.87 (s, 2H), 4.41 – 4.25 (m, 3H), 1.67 – 1.46 (m, 3H), 1.01 – 0.87 (m, 3H), 0.82 (d, *J* = 6.4 Hz, 3H). **MS (ESI):** m/z calculated for $C_{24}H_{27}N_5O_6$ [M+H] =482.20, found = 482.15

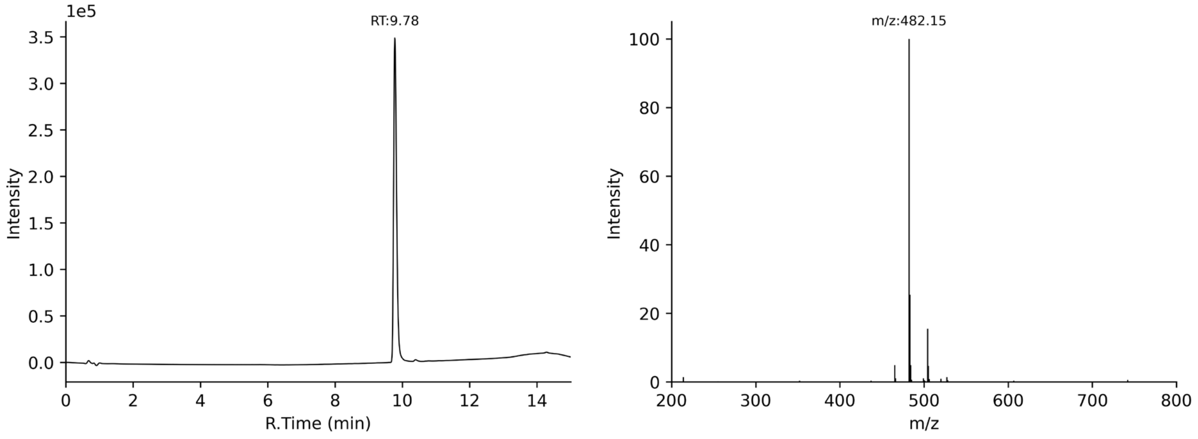

Supplementary Fig 41. LC-MS chromatogram from purified compound 6.

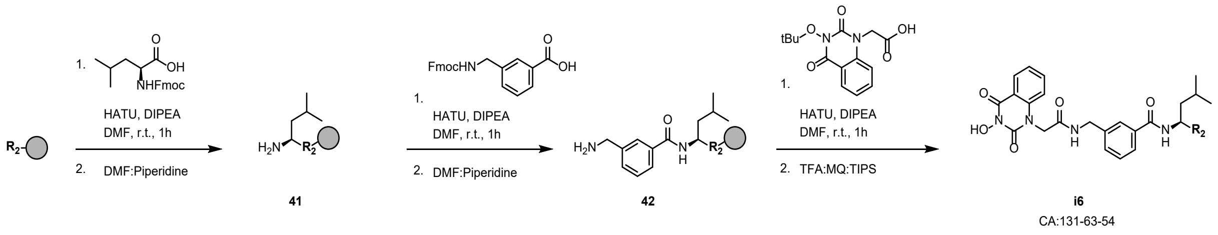

Product **i6** was synthesized on a 50 µmol scale according to general procedure: Manual solid-phase synthesis (SPS) as described in section 3.3 by respectively coupling Fmoc-Leu-OH, Fmoc-3-(aminomethyl)benzoic acid, and CA131. The compound was cleaved of the resin by incubating at room temperature for 1 hour with a solution of TFA:H_2O:TIPS (92.5:5:2.5) and washed with TFA:H_2O:TIPS (92.5:5:2.5). The volume was reduced by evaporating the TFA solution with a N_2 stream. The crude mixture was purified by reverse phase column chromatography (C18, 00-45% MQ:MeCN, 0.1% TFA) and product **i6** was isolated in 26% yield (6.3 mg, 13 µmol, >95% purity). **^{1}H NMR** (400 MHz, DMSO) δ 10.87 (s, 1H), 8.86 (t, *J* = 5.9 Hz, 1H), 8.35 (d, *J* = 8.3 Hz, 1H), 8.08 (dd, *J* = 7.8, 1.6 Hz, 1H), 7.81 – 7.77 (m, 2H), 7.75 (td, *J* = 8.7, 7.2, 1.7 Hz, 1H), 7.46 – 7.36 (m, 3H), 7.35 – 7.16 (m, 2H), 7.02 (d, *J* = 2.1 Hz, 1H), 4.86 (s, 2H), 4.53 – 4.43 (m, 1H), 4.36 (d, *J* = 5.9 Hz, 2H), 1.77 – 1.60 (m, 2H), 1.59 – 1.48 (m, 1H), 0.91 (dd, *J* = 12.7, 6.4 Hz, 6H). **MS (ESI):** m/z calculated for $C_{24}H_{27}N_5O_6$ [M+H] =482.20, found = 482.15

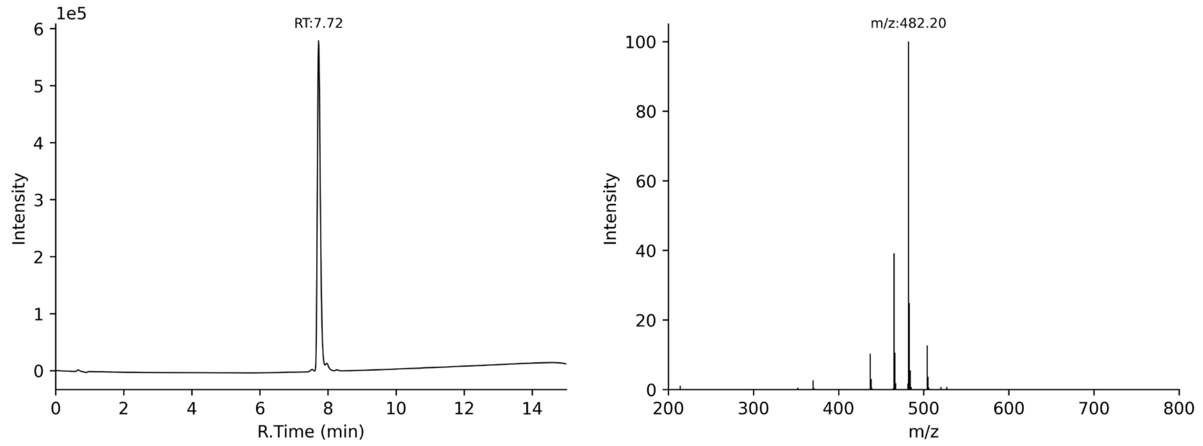

Supplementary Fig 42. LC-MS chromatogram from purified compound i6.

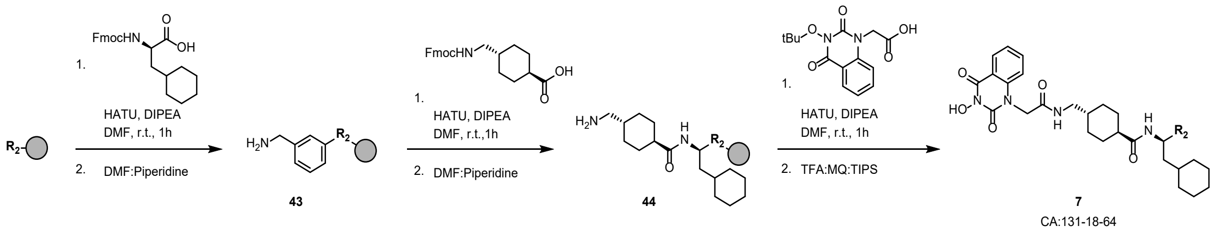

Product **7** was synthesized on a 50 µmol scale according to general procedure: Manual solid-phase synthesis (SPS) as described in section 3.3 by respectively coupling Fmoc-Cha-OH, Fmoc-tranexamic acid and CA131. The compound was cleaved of the resin by incubating at room temperature for 1 hour with a solution of TFA:H_2O:TIPS (92.5:5:2.5) and washed with TFA:H_2O:TIPS (92.5:5:2.5). The volume was reduced by evaporating the TFA solution with a N_2 stream. The crude mixture was purified by reverse phase column chromatography (C18, 00-45% MQ:MeCN) and product **7** was isolated in 28% yield (7.3 mg, 14 µmol, >95% purity). **^{1}H NMR** (400 MHz, DMSO) δ 10.88 (s, 1H), 8.50 (d, *J* = 8.3 Hz, 1H), 8.08 (dd, *J* = 7.9, 1.6 Hz, 1H), 7.98 (t, *J* = 5.8 Hz, 1H), 7.76 – 7.67 (m, 1H), 7.33 (t, *J* = 7.5 Hz, 1H), 7.19 (d, *J* = 8.4 Hz, 2H), 6.67 (s, 1H), 5.12 – 4.70 (m, 2H), 4.34 (td, *J* = 8.8, 5.7 Hz, 1H), 3.03 – 2.92 (m, 1H), 2.89 – 2.78 (m, 1H), 1.77 – 1.58 (m, 9H), 1.55 – 1.39 (m, 2H), 1.37 – 1.17 (m, 5H), 1.17 – 1.03 (m, 2H), 0.94 – 0.77 (m, 4H). **MS (ESI):** m/z calculated for $C_{27}H_{37}N_5O_6$ [M+H] =528.28, found = 528.30

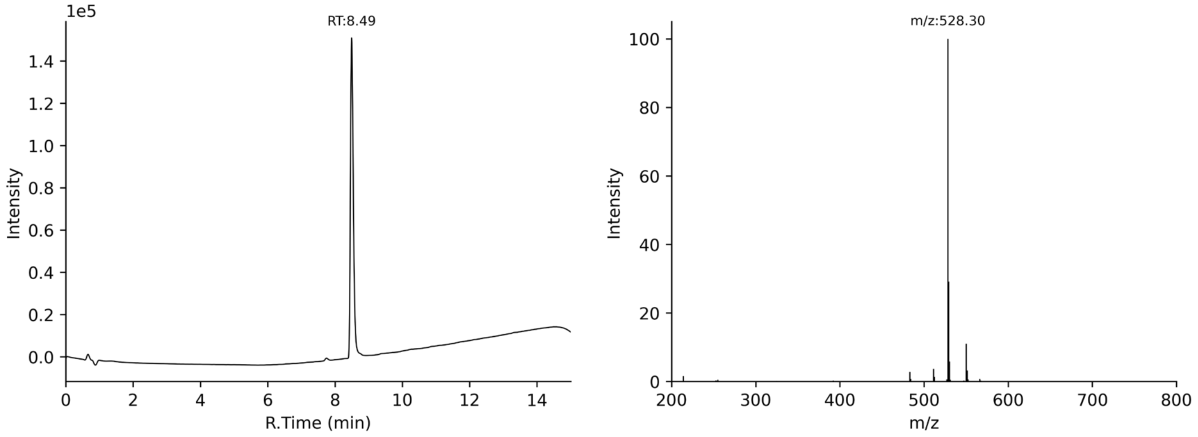

Supplementary Fig 43. LC-MS chromatogram from purified compound 7.

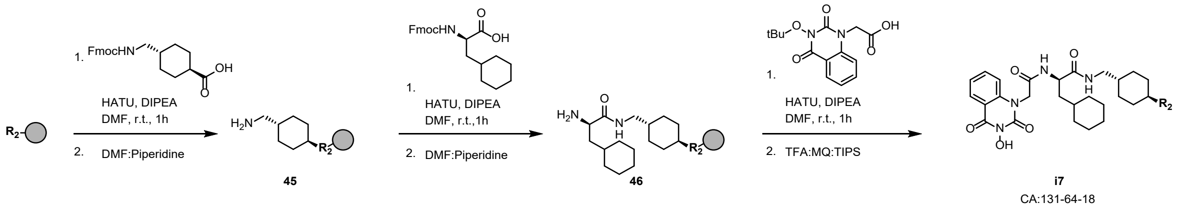

Product **i7** was synthesized on a 50 µmol scale according to general procedure: Manual solid-phase synthesis (SPS) as described in section 3.3 by respectively coupling, Fmoc-tranexamic acid, Fmoc-Cha-OH and CA131. The compound was cleaved of the resin by incubating at room temperature for 1 hour with a solution of TFA:H_2O:TIPS (92.5:5:2.5) and washed with TFA:H_2O:TIPS (92.5:5:2.5). The volume was reduced by evaporating the TFA solution with a N_2 stream. The crude mixture was purified by reverse phase column chromatography (C18, 00-45% MQ:MeCN) and product **i7** was isolated in 28% yield (7.3 mg, 14 µmol, >95% purity). **^{1}H NMR** (400 MHz, DMSO) δ 10.86 (s, 1H), 8.26 (s, 1H), 8.09 (d, *J* = 8.1 Hz, 1H), 7.73 (d, *J* = 9.3 Hz, 2H), 7.44 – 7.10 (m, 3H), 6.92 (s, 1H), 4.77 (s, 2H), 4.24 (s, 1H), 3.52 (s, 1H), 2.94 (s, 3H), 2.12 (s, 2H), 1.67 (d, *J* = 17.2 Hz, 10H), 1.50 – 0.98 (m, 13H), 0.85 (s, 4H). **MS (ESI):** m/z calculated for $C_{27}H_{37}N_5O_6$ [M+H] =528.28, found = 528.30

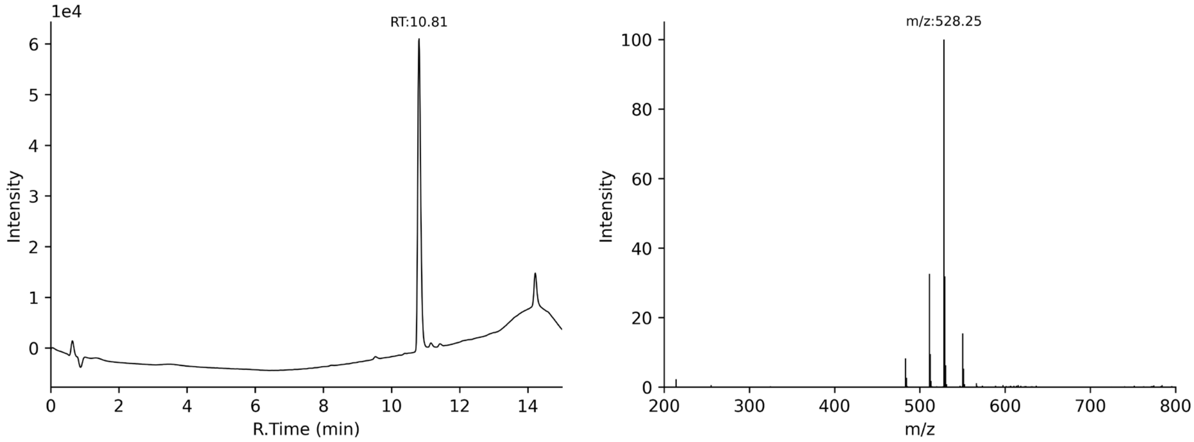

Supplementary Fig 44. LC-MS chromatogram from purified compound i7.

10. Appendix

10.1. List of building blocks

Supplementary Table 8. Overview of amino acids used in SEL 1 and 2

Amino acid (AA)	Cas no	SMILES
AA1	852288-18-7	CC1(C)OC2=CC=C(C[C@H](NC(=O)OCC3C4=CC=CC=C4C4=CC=CC=C34)C(O)=O)C=C2O1
AA2	173690-50-1	CN(C[C@H]1CC[C@@H](CC1)C(O)=O)C(=O)OCC1C2=CC=CC=C2C2=CC=CC=C12
AA3	204693-22-1	OC(=O)[C@@H](CC1=CC(Br)=C(O)C(Br)=C1)NC(=O)OCC1C2=CC=CC=C2C2=CC=CC=C12
AA4	215190-27-5	OC(=O)CN1CN(C2=CC=CC=C2)C2(CCN(CC2)C(=O)OCC2C3=CC=CC=C3C3=CC=CC=C23)C1=O
AA5	885951-77-9	OC(=O)C1(CCC1)NC(=O)OCC1C2=CC=CC=C2C2=CC=CC=C12
AA6	368866-30-2	OC(=O)C1(CCN(CC2=CC=CC=C2)CC1)NC(=O)OCC1C2=CC=CC=C2C2=CC=CC=C12
AA7	186320-22-9	OC(=O)C(NC(=O)OCC1C2=CC=CC=C2C2=CC=CC=C12)C1CCCCC1
AA8	1629658-27-0	OC(=O)[C@H](CC1=CC=C(Cl)C(F)=C1)NC(=O)OCC1C2=CC=CC=C2C2=CC=CC=C12
AA9	103478-58-6	CC(C)[C@@H](N(C)C(=O)OCC1C2=CC=CC=C2C2=CC=CC=C12)C(O)=O
AA10	185379-39-9	OC(=O)[C@@H](CC1=CC=CC=N1)NC(=O)OCC1C2=CC=CC=C2C2=CC=CC=C12
AA11	270063-55-3	OC(=O)C[C@H](CC1=CC=C(F)C(F)=C1)NC(=O)OCC1C2=CC=CC=C2C2=CC=CC=C12
AA12	198560-38-2	OC(=O)[C@@H]1CC2=CC=CC=C2N1C(=O)OCC1C2=CC=CC=C2C2=CC=CC=C12
AA13	372159-75-6	OC(=O)C1=CC2=CC=CC=C2C=C1NC(=O)OCC1C2=CC=CC=C2C2=CC=CC=C12
AA14	1986905-26-3	OC(=O)C1(CCC(F)(F)CC1)NC(=O)OCC1C2=CC=CC=C2C2=CC=CC=C12
AA15	204318-02-5	OC(=O)[C@@H]1CCCN1C1CCN(CC1)C(=O)OCC1C2=CC=CC=C2C2=CC=CC=C12
AA16	1212257-18-5	OC(=O)[C@@H](NC(=O)OCC1C2=CC=CC=C2C2=CC=CC=C12)C1CC1
AA17	117322-30-2	OC(=O)C1(CCCC1)NC(=O)OCC1C2=CC=CC=C2C2=CC=CC=C12
AA18	167690-53-1	OC(=O)[C@H]1CC[C@H](CNC(=O)OCC2C3=CC=CC=C3C3=CC=CC=C23)CC1
AA19	212783-75-0	OC(=O)COC1=CC=C2C(NC(=O)OCC3C4=CC=CC=C4C4=CC=CC=C34)C3=CC=CC=C3CCC2=C1
AA20	1335206-44-4	OC(=O)COC[C@H]1CCCN1C(=O)OCC1C2=CC=CC=C2C2=CC=CC=C12
AA21	84000-07-7	C[C@H](N(C)C(=O)OCC1C2=CC=CC=C2C2=CC=CC=C12)C(O)=O
AA22	478183-58-3	OC(=O)[C@H](CC1=CC=C(O)C(Cl)=C1)NC(=O)OCC1C2=CC=CC=C2C2=CC=CC=C12
AA23	166881-42-1	O=C(O)CNC(OCC1C2=CC=CC=C2C3=CC=CC=C13)=O
AA24	178119-94-3	OC(=O)[C@H](CC1=CNC2=C1C=C(O)C=C2)NC(=O)OCC1C2=C(C=CC=C2)C2=C1C=CC=C2
AA25	133054-21-4	OC(=O)[C@@H]1CSCN1C(=O)OCC1C2=CC=CC=C2C2=CC=CC=C12
AA26	193885-59-5	CCCCCCCC[C@H](NC(=O)OCC1C2=CC=CC=C2C2=CC=CC=C12)C(O)=O

AA27	252049-14-2	CC(C)(C)C1=CC=C(C[C@@H](NC(=O)OCC2C3=CC=CC=C3C3=CC=CC=C23)C(O)=O)C=C1
AA28	220497-66-5	OC(=O)[C@H]1CC[C@H](C1)NC(=O)OCC1C2=CC=CC=C2C2=CC=CC=C12
AA29	144701-25-7	OC(=O)[C@@H](CC1CCCCC1)NC(=O)OCC1C2=CC=CC=C2C2=CC=CC=C12
AA30	180576-05-0	OC(=O)CN1CCN(CC1)C(=O)OCC1C2=CC=CC=C2C2=CC=CC=C12
AA31	1380327-56-9	OC(=O)C1(COC1)NC(=O)OCC1C2=CC=CC=C2C2=CC=CC=C12
AA32	180181-05-9	OC(=O)CC1CCN(CC1)C(=O)OCC1C2=CC=CC=C2C2=CC=CC=C12
AA33	2170726-27-7	OC(=O)[C@@H]1CC2(CC2)CN1C(=O)OCC1C2=CC=CC=C2C2=CC=CC=C12
AA34	193693-64-0	OC(=O)C1CN(C1)C(=O)OCC1C2=CC=CC=C2C2=CC=CC=C12
AA35	198545-76-5	OC(=O)[C@@H](CC1=CC=C(Br)C=C1)NC(=O)OCC1C2=CC=CC=C2C2=CC=CC=C12
AA36	203866-19-7	OC(=O)[C@@H]1C[C@H](F)CN1C(=O)OCC1C2=CC=CC=C2C2=CC=CC=C12
AA37	185116-43-2	OC(=O)C1=CC=C(NC(=O)OCC2C3=CC=CC=C3C3=CC=CC=C23)C=C1
AA38	203866-21-1	OC(=O)[C@@H]1CC(F)(F)CN1C(=O)OCC1C2=CC=CC=C2C2=CC=CC=C12
AA39	220497-85-8	OC(=O)[C@@H](CC1=CC=CO1)NC(=O)OCC1C2=CC=CC=C2C2=CC=CC=C12
AA40	84891-19-0	OC(=O)[C@H](CC1=CN=CN1COCC1=CC=CC=C1)NC(=O)OCC1C2=CC=CC=C2C2=CC=CC=C12
AA41	126705-22-4	OC(=O)C1(CC1)NC(=O)OCC1C2=CC=CC=C2C2=CC=CC=C12
AA42	77128-70-2	CN(CC(=O)O)C(=O)OCC1C2=CC=CC=C2C3=CC=CC=C13
AA43	1197020-22-6	CCCCC[C@@H](C(=O)O)NC(=O)OCC1C2=CC=CC=C2C3=CC=CC=C13
AA44	478183-62-9	OC(C(CC1CCC1)NC(OCC2C(C=CC=C3)=C3C4=C2C=CC=C4)=O)=O
AA45	371770-32-0	C1CCC(C1)C[C@@H](C(=O)O)NC(=O)OCC2C3=CC=CC=C3C4=CC=CC=C24
AA46	86069-86-5	C1CCN([C@@H](C1)C(=O)O)C(=O)OCC2C3=CC=CC=C3C4=CC=CC=C24
AA47	35661-39-3	O=C(O)[C@H](C)NC(OCC1C2=CC=CC=C2C3=CC=CC=C13)=O
AA48	71989-33-8	O=C(O)[C@@H](NC(OCC1C(C=CC=C2)=C2C3=C1C=CC=C3)=O)COC(C)(C)C
AA49	71989-31-6	O=C(O)[C@H]1N(C(OCC2C3=CC=CC=C3C4=CC=CC=C24)=O)CCC1
AA50	71989-35-0	O=C(O)[C@@H](NC(OCC1C(C=CC=C2)=C2C3=C1C=CC=C3)=O)C(C)OC(C)(C)C
AA51	68858-20-8	O=C(O)[C@H](C(C)C)NC(OCC1C2=CC=CC=C2C3=CC=CC=C13)=O
AA52	132388-59-1	O=C(O)[C@H](CC(NC(C1=CC=CC=C1)(C2=CC=CC=C2)C3=CC=CC=C3)=O)NC(OCC4C(C=CC=C5)=C5C6=C4C=CC=C6)=O
AA53	71989-14-5	O=C(O)[C@@H](NC(OCC1C(C=CC=C2)=C2C3=C1C=CC=C3)=O)CC(OC(C)(C)C)=O
AA54	35661-60-0	O=C(O)[C@H](CC(C)C)NC(OCC1C2=CC=CC=C2C3=CC=CC=C13)=O
AA55	71989-18-9	O=C(O)[C@H](CCC(OC(C)(C)C)=O)NC(OCC1C2=CC=CC=C2C3=CC=CC=C13)=O
AA56	132327-80-1	O=C(O)[C@H](CCC(NC(C1=CC=CC=C1)(C2=CC=CC=C2)C3=CC=CC=C3)=O)NC(OCC4C(C=CC=C5)=C5C6=C4C=CC=C6)=O
AA57	71989-26-9	O=C(O)[C@@H](NC(OCC1C(C=CC=C2)=C2C3=C1C=CC=C3)=O)CCCCNC(OC(C)(C)C)=O

AA58	109425-51-6	O=C(O)[C@@H](NC(OCC1C(C=CC=C2)=C2C3=C1C=CC=C3)=O)CC4=CN(C(C5=CC=CC=C5)(C6=CC=CC=C6)C7=CC=CC=C7)C=N4
AA59	154445-77-9	O=C(O)[C@@H](NC(OCC1C(C=CC=C2)=C2C3=C1C=CC=C3)=O)CCCNC(NS(C4=C(C)C(CC(C)(C)O5)=C5C(C)=C4C)(=O)=O)=N
AA60	35661-40-6	O=C(O)[C@H](CC1=CC=CC=C1)NC(OCC2C3=CC=CC=C3C4=CC=CC=C24)=O
AA61	71989-38-3	O=C(O)[C@@H](NC(OCC1C(C=CC=C2)=C2C3=C1C=CC=C3)=O)CC4=CC=C(OC(C)(C)C)C=C4
AA62	143824-78-6	O=C(O)[C@@H](NC(OCC1C(C=CC=C2)=C2C3=C1C=CC=C3)=O)CC4=CN(C(OC(C)(C)C)=O)C5=C4C=CC=C5

Supplementary Table 9. Overview of carboxylic acids used in SEL 1

Carboxylic acid (CA)	Cas no	SMILES
CA1	5469-45-4	CC(=O)N/C(=C\C1=CC=CC=C1)/C(=O)O
CA2	2345-34-8	CC(=O)OC1=CC=C(C=C1)C(=O)O
CA3	6340-79-0	C1=CC(=CC=C1C(=O)CCC(=O)O)Br
CA4	56518-42-4	COC1=CC(=CC(=C1Br)OC)C(=O)O
CA5	3984-34-7	C1=CC(=CC=C1C(=O)CCC(=O)O)Cl
CA6	403-16-7	C1=CC(=C(C=C1C(=O)O)Cl)F
CA7	535-80-8	C1=CC(=CC(=C1)Cl)C(=O)O
CA8	50-30-6	C1=CC(=C(C(=C1)Cl)C(=O)O)Cl
CA9	5807-30-7	C1=CC(=C(C=C1CC(=O)O)Cl)Cl
CA10	455-38-9	C1=CC(=CC(=C1)F)C(=O)O
CA11	405-50-5	C1=CC(=CC=C1CC(=O)O)F
CA12	42823-46-1	C1=CC(=CC=C1C(=O)O)N=C(N)N.Cl
CA13	99-06-9	C1=CC(=CC(=C1)O)C(=O)O
CA14	619-58-9	C1=CC(=CC=C1C(=O)O)I
CA15	18698-96-9	C1=CC=C(C(=C1)CC(=O)O)I
CA16	5965-83-3	C1=CC(=C(C=C1S(=O)(=O)O)C(=O)O)O.O.O
CA17	530-57-4	COC1=CC(=CC(=C1O)OC)C(=O)O
CA18	17794-48-8	C1=CC(=CC(=C1)C(F)(F)F)C(=O)NCC(=O)O
CA19	951-82-6	COC1=CC(=CC(=C1OC)OC)CC(=O)O
CA20	118-41-2	COC1=CC(=CC(=C1OC)OC)C(=O)O
CA21	2067-33-6	C(CCBr)CC(=O)O
CA22	536-66-3	CC(C)C1=CC=C(C=C1)C(=O)O
CA23	13205-48-6	CSC1=CC=C(C=C1)C(=O)O
CA24	79-09-4	CCC(=O)O
CA25	634-97-9	C1=CNC(=C1)C(=O)O
CA26	109-52-4	CCCCC(=O)O
CA27	1006-41-3	C1=CC(=C(C=C1F)Br)C(=O)O
CA28	10601-99-7	C#CC1=CC(=CC=C1)C(=O)O
CA29	156-54-7	CCCC(=O)[O-].[Na+]
CA30	618-58-6	c1c(cc(cc1Br)Br)C(=O)O
CA31	28440-13-3	CC1(O[C@H]2[C@@H](O1)[C@@H](O[C@@H]2C(=O)O)n3cnc4c3nc[nH]c4=O)C
CA32	64-19-7	CC(=O)O
CA33	488-93-7	OC(=O)C1=COC=C1
CA34	23012-13-7	Cl.OC(=O)C1=COC=N1
CA35	103879-58-9	CC1=C(N=CO1)C(O)=O
CA36	6964-21-2	OC(=O)CC1=CSC=C1
CA37	1878-58-6	OC(=O)COC1=CC=C2CCCC2=C1
CA38	33632-74-5	OC(=O)C1CCOC2=CC=CC=C2O1
CA39	2745-26-8	OC(=O)CC1=CC=CO1
CA40	1001907-64-7	OC(=O)C1CC2(C1)OCCO2
CA41	19155-88-5	OC(=O)C1=CC2=NON=C2C=C1
CA42	14381-42-1	OC(=O)C1CCC2=CC=CC=C12
CA43	4324-37-2	COC(C)C(O)=O

CA44	73873-61-7	CO[C@H]1CC[C@@H](CC1)C(O)=O
CA45	197507-59-8	OC(=O)C1=NC2=CC=NC=C2C=C1
CA46	37868-26-1	OC(=O)CC1CC2=CC=CC=C2C1
CA47	1188-02-9	CCCCCC(C)C(O)=O
CA48	16179-97-8	Cl.OC(=O)CC1=CC=CC=N1
CA49	69999-16-2	OC(=O)CC1=CC=C2OCCC2=C1
CA50	4595-61-3	OC(=O)C1=CN=CN=C1
CA51	1914-60-9	OC(=O)C1CC2=CC=CC=C2O1
CA52	66500-55-8	OC(=O)C1CCC2(CC1)OCCO2
CA53	57252-83-2	CC1CC(C1)C(O)=O
CA54	5622-34-4	OC(=O)CC1=CC2=CC=CN=C2C=C1
CA55	22468-26-4	OC(=O)C1=NC=CC(O)=C1
CA56	39793-31-2	OC(=O)C1=CC2=C(N1)C=CS2
CA57	32857-63-9	CC(C)(C)C1=CC=C(CC(O)=O)C=C1
CA58	52898-06-3	COC1=NOC(CCC(O)=O)=C1
CA59	120-23-0	OC(=O)COC1=CC2=C(C=CC=C2)C=C1
CA60	628-46-6	CC(C)CCCC(O)=O
CA61	5006-44-0	CC1=CC=C2OC(=CC(=O)C2=C1)C(O)=O
CA62	38289-29-1	CCCCC[C@H]1CC[C@@H](CC1)C(O)=O
CA63	53572-98-8	OC(=O)C1=CN2C=CSC2=N1
CA64	123530-67-6	OC(=O)\C=C\C1=NC2=CC=CC=C2S1
CA65	87392-07-2	OC(=O)[C@@H]1CCCO1
CA66	625-38-7	OC(=O)CC=C
CA67	54947-74-9	CCCCC(C)CCC(O)=O
CA68	15760-36-8	OC(=O)C1CC(=C)C1
CA69	99924-18-2	OC(=O)C1=C(OC=N1)C1=CC=CC=C1
CA70	300691-07-0	CC1=NC2=CC(=NN2C(C)=C1)C(O)=O
CA71	17202-56-1	OC(=O)C1CC11CCC1
CA72	2345-51-9	OC(=O)CC#C
CA73	625-45-6	COCC(=O)O
CA74	3721-95-7	C1CC(C1)C(=O)O
CA75	586-38-9	COc1cccc(c1)C(=O)O
CA76	186046-78-6	O=C1N(CC(O)=O)C=CC(NC(OC(C2=CC=CC=C2)C3=CC=CC=C3)=O)=N1
CA77	138-41-0	O=S(C(C=C1)=CC=C1C(O)=O)(N)=O
CA78	25503-90-6	CC(=O)N1CCC(CC1)C(=O)O
CA79	611-73-4	c1ccc(cc1)C(=O)C(=O)O
CA80	13726-69-7	CC(C)(C)OC(=O)N1C[C@@H](C[C@H]1C(=O)O)O
CA81	586-76-5	c1cc(ccc1C(=O)O)Br
CA82	21739-92-4	c1cc(c(cc1Br)C(=O)O)Cl
CA83	1643-30-7	c1cc(ccc1CCC(=O)O)Br
CA84	18698-97-0	c1ccc(c(c1)CC(=O)O)Br
CA85	98-73-7	CC(C)(C)c1ccc(cc1)C(=O)O
CA86	627-00-9	C(CC(=O)O)CCl
CA87	166316-48-9	B(c1ccc(cc1)CCC(=O)O)(O)O
CA88	21327-86-6	Cc1c(c(ccc1)Cl)C(=O)O
CA89	107-92-6	CCCC(=O)O

CA90	619-65-8	c1cc(ccc1C#N)C(=O)O
CA91	372-09-8	C(C#N)C(=O)O
CA92	91-52-1	COc1cc(c(cc1)C(=O)O)OC
CA93	99-64-9	CN(C)c1cccc(c1)C(=O)O
CA94	20736-28-1	Cc1cc(c(cc1C(=O)O)OC)OC
CA95	117-34-0	c1ccc(cc1)C(c2ccccc2)C(=O)O
CA96	17847-26-6	CN(C)Cc1ccc(cc1)C(=O)O.Cl
CA97	619-64-7	CCc1ccc(cc1)C(=O)O
CA98	142-62-1	CCCCCC(=O)O
CA99	13205-46-4	CC(C)Oc1ccc(cc1)C(=O)O
CA100	1798-09-0	COc1cccc(c1)CC(=O)O
CA101	2373-80-0	C1Oc2c(O1)cc(cc2)/C=C/C(=O)O
CA102	16136-58-6	Cn1c2ccccc2cc1C(=O)O
CA103	59-67-6	c1cc(cnc1)C(=O)O
CA104	6328-74-1	c1ccc(cc1)Oc2ccc(cc2)CC(=O)O
CA105	103-82-2	c1ccc(cc1)CC(=O)O
CA106	947-84-2	c1ccc(cc1)-c2ccccc2C(=O)O
CA107	2207-75-2	c1(nc(=O)[nH]c(=O)[nH]1)C(=O)[O-].[K+]
CA108	94-53-1	C1Oc2c(O1)cc(cc2)C(=O)O
CA109	10124-65-9	CCCCCCCCCCCC(=O)[O-].[K+]
CA110	127-17-3	CC(=O)C(=O)O
CA111	433-97-6	c1ccc(c(c1)C(=O)O)C(F)(F)F
CA112	564-10-3	C(C(=O)O)(C(F)(F)F)C(F)(F)F
CA113	1460-16-8	C1CCCC(CC1)C(=O)O
CA114	98-89-5	C1CCC(CC1)C(=O)O
CA115	1477-50-5	c1ccc2c(c1)cc([nH]2)C(=O)O
CA116	581-96-4	c1ccc2cc(ccc2c1)CC(=O)O
CA117	65-86-1	c1c([nH]c(=O)[nH]c1=O)C(=O)O
CA118	3724-19-4	c1cc(cnc1)CCC(=O)O
CA119	6480-68-8	c1ccc2c(c1)cc(cn2)C(=O)O
CA120	351-35-9	c1cc(cc(c1)C(F)(F)F)CC(=O)O
CA121	582-25-2	c1ccc(cc1)C(=O)[O-].[K+]
CA122	6089-09-4	C#CCCC(=O)O
CA123	4052-30-6	CS(=O)(=O)c1ccc(cc1)C(=O)O
CA124	121-34-6	COc1c(ccc(c1)C(=O)O)O
CA125	68986-76-5	c1csc(c1)C(=O)[O-].[Cu+]
CA126	471-25-0	C#CC(=O)O
CA127	25462-85-5	Cc1cc(cc(n1)Cl)C(=O)O
CA128	20924-05-4	Cc1cn(c(=O)[nH]c1=O)CC(=O)O
CA129	14381-41-0	OC(=O)C1CC2=C1C=CC=C2
CA130	1028077-12-4	CC(C)(C)OC(=O)NC1=NC2=C(N=CN2CC(O)=O)C(=O)N1

Supplementary Table 10. Overview of amines used in SEL 2

Amine (AM)	Cas no	SMILES
AM1	27578-60-5	C1CCN(CC1)CCN
AM2	5351-17-7	C1=CC(=CC=C1C(=O)NN)N
AM3	15996-76-6	C1=CC(=CC=C1CN)C#N.Cl
AM4	17768-41-1	C1C2CC3CC1CC(C2)(C3)CN
AM5	934-98-5	CN1CCN(CC1)CCN
AM6	7154-73-6	C1CCN(C1)CCN
AM7	23159-07-1	C1CCN(C1)CCCN
AM8	616-24-0	CCC(CC)N
AM9	5456-63-3	C1CC[C@H]([C@@H](C1)N)O.Cl
AM10	2975-41-9	C1C(CC2=CC=CC=C21)N
AM11	27489-62-9	C1CC(CCC1N)O
AM12	929-06-6	C(COCCO)N
AM13	60-32-2	C(CCC(=O)O)CCN
AM14	1002-57-9	C(CCCC(=O)O)CCCN
AM15	193269-78-2	CC(C)(C)OC(=O)N1CC(C1)N
AM16	35320-23-1	C[C@H](CO)N
AM17	104-94-9	COC1=CC=C(C=C1)N
AM18	22600-77-7	C1=CN=C(N1)CN.Cl.Cl
AM19	38041-19-9	C1COCCC1N
AM20	100-46-9	C1=CC=C(C=C1)CN
AM21	2835-68-9	NC(=O)c1ccc(N)cc1
AM22	104-13-2	CCCCC1=CC=C(C=C1)N
AM23	4152-90-3	C1=CC(=CC(=C1)Cl)CN
AM24	104-86-9	C1=CC(=CC=C1CN)Cl
AM25	108-91-8	C1CCC(CC1)N
AM26	3218-02-8	C1CCC(CC1)CN
AM27	5454-82-0	C1CC(C1)CN.Cl
AM28	3963-62-0	C1=CC=C(C=C1)C(CN)C2=CC=CC=C2
AM29	78710-55-1	CC1=CC(=CC(=C1)CN)C
AM30	95-64-7	CC1=C(C=C(C=C1)N)C
AM31	141-43-5	C(CO)N
AM32	589-16-2	CCC1=CC=C(C=C1)N
AM33	100-82-3	C1=CC(=CC(=C1)F)CN
AM34	140-75-0	C1=CC(=CC=C1CN)F
AM35	372-19-0	C1=CC(=CC(=C1)F)N
AM36	371-40-4	C1=CC(=CC=C1N)F
AM37	696-40-2	C1=CC(=CC(=C1)I)CN
AM38	78-81-9	CC(C)CN
AM39	89-93-0	CC1=CC=CC=C1CN
AM40	91-59-8	C1=CC=C2C=C(C=CC2=C1)N
AM41	13214-66-9	C1=CC=C(C=C1)CCCCN
AM42	35303-76-5	C1=CC(=CC=C1CCN)S(=O)(=O)N
AM43	4795-29-3	C1CC(OC1)CN
AM44	2740-83-2	C1=CC(=CC(=C1)C(F)(F)F)CN

AM45	3261-62-9	CC1=CC=C(C=C1)CCN
AM46	3048-01-9	C1=CC=C(C(=C1)CN)C(F)(F)F
AM47	3300-51-4	C1=CC(=CC=C1CN)C(F)(F)F
AM48	106-49-0	CC1=CC=C(C=C1)N
AM49	51-67-2	C1=CC(=CC=C1CCN)O
AM50	62-53-3	C1=CC=C(C=C1)N
AM51	536-90-3	COC1=CC=CC(=C1)N
AM52	62-31-7	C1=CC(=C(C=C1CCN)O)O.Cl

Supplementary Table 11. Overview of aldehydes used in SEL 2

Aldehyde (AL)	Cas no	SMILES
AL1	40663-68-1	C=CCOC1=CC=C(C=C1)C=O
AL2	1122-91-4	C1=CC(=CC=C1C=O)Br
AL3	4397-53-9	C1=CC=C(C=C1)COC2=CC=C(C=C2)C=O
AL4	587-04-2	C1=CC(=CC(=C1)Cl)C=O
AL5	85070-48-0	C1=CC(=C(C(=C1)Cl)F)C=O
AL6	105-07-7	C1=CC(=CC=C1C=O)C#N
AL7	874-42-0	C1=CC(=C(C=C1Cl)Cl)C=O
AL8	120-14-9	COC1=C(C=C(C=C1)C=O)OC
AL9	23074-10-4	CCC1=CC=C(O1)C=O
AL10	10031-82-0	CCOC1=CC=C(C=C1)C=O
AL11	459-57-4	C1=CC(=CC=C1C=O)F
AL12	446-52-6	C1=CC=C(C(=C1)C=O)F
AL13	98-01-1	C1=COC(=C1)C=O
AL14	100-83-4	C1=CC(=CC(=C1)O)C=O
AL15	621-59-0	COC1=C(C=C(C=C1)C=O)O
AL16	4221-03-8	C(CCO)CC=O
AL17	123-08-0	C1=CC(=CC=C1C=O)O
AL18	590-86-3	CC(C)CC=O
AL19	101-39-3	C/C(=C\C1=CC=CC=C1)/C=O
AL20	16251-77-7	CC(CC=O)C1=CC=CC=C1
AL21	620-23-5	CC1=CC(=CC=C1)C=O
AL22	104-87-0	CC1=CC=C(C=C1)C=O
AL23	52771-21-8	C1=CC(=CC(=C1)OC(F)(F)F)C=O
AL24	659-28-9	C1=CC(=CC=C1C=O)OC(F)(F)F
AL25	121-33-5	COC1=C(C=CC(=C1)C=O)O
AL26	123-11-5	COC1=CC=C(C=C1)C=O
AL27	100-52-7	C1=CC=C(C=C1)C=O
AL28	500-22-1	C1=CC(=CN=C1)C=O
AL29	6688-11-5	C1CCCC(CCC1)C=O
AL30	1963-36-6	COC1=CC=C(C=C1)/C=C/C=O
AL31	2579-22-8	C1=CC=C(C=C1)C#CC=O
AL32	22960-16-3	C1=CC=C2C(=C1)C=NC=C2C=O
AL33	98-03-3	C1=CSC(=C1)C=O
AL34	100-50-5	C1CC(CC=C1)C=O
AL35	139-85-5	C1=CC(=C(C=C1C=O)O)O
AL36	6287-38-3	C1=CC(=C(C=C1C=O)Cl)Cl
AL37	1192-58-1	CN1C=CC=C1C=O
AL38	27258-32-8	Cn1ccc(C=O)n1
AL39	208110-81-0	Fc1cccc(C=O)n1
AL40	55589-47-4	Cc1cccnc1C=O
AL41	25711-30-2	Cc1c(C=O)cnn1C
AL42	5780-66-5	O=Cc1cnccn1
AL43	155601-65-3	Fc1ccc(C=O)c(F)n1
AL44	177756-62-6	Cc1ccc(C=O)cc1F

AL45	21508-19-0	Clc1ccc(C=O)o1
AL46	175204-81-6	Cn1cc(Cl)c(C=O)n1
AL47	450-83-9	COc1cc(F)ccc1C=O
AL48	58551-83-0	Fc1cc(F)c(C=O)c(F)c1
AL49	18486-69-6	CC1(C)[C@@H]2C[C@H]1C(C=O)=CC2
AL50	176433-43-5	Cc1cc(C=O)cnc1Cl
AL51	84194-30-9	Fc1ccc(Cl)c(C=O)c1
AL52	256417-10-4	COc1cc(F)c(C=O)c(F)c1
AL53	446866-87-1	O=Cc1cccc(c1)N1CCOCC1
AL54	29866-54-4	COc1cccc(Cl)c1C=O
AL55	5305-40-8	Clc1ncnc(Cl)c1C=O
AL56	22065-85-6	O=CC1CCN(Cc2ccccc2)CC1
AL57	10338-57-5	O=Cc1ccc(cc1)N1CCCCC1
AL58	65373-52-6	O=Cc1ncco1
AL59	34246-54-3	CCc1cccc(C=O)c1
AL60	5198-79-8	Clc1nc(C=O)cs1
AL61	97455-61-3	CCOc1ccc(C=O)cn1
AL62	286474-59-7	O=CC1=C(Cl)C=CC(C)=C1F
AL63	99419-31-5	O=CC1(C)COC1
AL64	2043-61-0	O=CC1CCCCC1
AL65	467442-15-5	FC1=CC(C#N)=CC(F)=C1C=O
AL66	55745-70-5	O=CC1=CC2=C(C=C1)OCC2
AL67	113118-83-5	COC1=CN=CC(C=O)=C1

Supplementary Table 12. Overview of amino acids used in SEL 3

Amino acid (AA)	Cas no	SMILES
AA1	852288-18-7	CC1(C)OC2=CC=C(C[C@H](NC(=O)OCC3C4=CC=CC=C4C4=CC=CC=C34)C(O)=O)C=C2O1
AA2	173690-50-1	CN(C[C@H]1CC[C@@H](CC1)C(O)=O)C(=O)OCC1C2=CC=CC=C2C2=CC=CC=C12
AA4	215190-27-5	OC(=O)CN1CN(C2=CC=CC=C2)C2(CCN(CC2)C(=O)OCC2C3=CC=CC=C3C3=CC=CC=C23)C1=O
AA5	885951-77-9	OC(=O)C1(CCC1)NC(=O)OCC1C2=CC=CC=C2C2=CC=CC=C12
AA6	368866-30-2	OC(=O)C1(CCN(CC2=CC=CC=C2)CC1)NC(=O)OCC1C2=CC=CC=C2C2=CC=CC=C12
AA7	186320-22-9	OC(=O)C(NC(=O)OCC1C2=CC=CC=C2C2=CC=CC=C12)C1CCCCC1
AA8	1629658-27-0	OC(=O)[C@H](CC1=CC=C(Cl)C(F)=C1)NC(=O)OCC1C2=CC=CC=C2C2=CC=CC=C12
AA9	103478-58-6	CC(C)[C@@H](N(C)C(=O)OCC1C2=CC=CC=C2C2=CC=CC=C12)C(O)=O
AA10	185379-39-9	OC(=O)[C@@H](CC1=CC=CC=N1)NC(=O)OCC1C2=CC=CC=C2C2=CC=CC=C12
AA11	270063-55-3	OC(=O)C[C@H](CC1=CC=C(F)C(F)=C1)NC(=O)OCC1C2=CC=CC=C2C2=CC=CC=C12
AA12	198560-38-2	OC(=O)[C@@H]1CC2=CC=CC=C2N1C(=O)OCC1C2=CC=CC=C2C2=CC=CC=C12
AA13	372159-75-6	OC(=O)C1=CC2=CC=CC=C2C=C1NC(=O)OCC1C2=CC=CC=C2C2=CC=CC=C12
AA14	1986905-26-3	OC(=O)C1(CCC(F)(F)CC1)NC(=O)OCC1C2=CC=CC=C2C2=CC=CC=C12
AA15	204318-02-5	OC(=O)[C@@H]1CCCN1C1CCN(CC1)C(=O)OCC1C2=CC=CC=C2C2=CC=CC=C12
AA16	1212257-18-5	OC(=O)[C@@H](NC(=O)OCC1C2=CC=CC=C2C2=CC=CC=C12)C1CC1
AA17	117322-30-2	OC(=O)C1(CCCC1)NC(=O)OCC1C2=CC=CC=C2C2=CC=CC=C12
AA18	167690-53-1	OC(=O)[C@H]1CC[C@H](CNC(=O)OCC2C3=CC=CC=C3C3=CC=CC=C23)CC1
AA19	212783-75-0	OC(=O)COC1=CC=C2C(NC(=O)OCC3C4=CC=CC=C4C4=CC=CC=C34)C3=CC=CC=C3CCC2=C1
AA20	1335206-44-4	OC(=O)COC[C@H]1CCCN1C(=O)OCC1C2=CC=CC=C2C2=CC=CC=C12
AA21	84000-07-7	C[C@H](N(C)C(=O)OCC1C2=CC=CC=C2C2=CC=CC=C12)C(O)=O
AA22	478183-58-3	OC(=O)[C@H](CC1=CC=C(O)C(Cl)=C1)NC(=O)OCC1C2=CC=CC=C2C2=CC=CC=C12
AA23	166881-42-1	O=C(O)CNC(OCC1C2=CC=CC=C2C3=CC=CC=C13)=O
AA24	178119-94-3	OC(=O)[C@H](CC1=CNC2=C1C=C(O)C=C2)NC(=O)OCC1C2=C(C=CC=C2)C2=C1C=CC=C2
AA25	133054-21-4	OC(=O)[C@@H]1CSCN1C(=O)OCC1C2=CC=CC=C2C2=CC=CC=C12
AA26	193885-59-5	CCCCCCCC[C@H](NC(=O)OCC1C2=CC=CC=C2C2=CC=CC=C12)C(O)=O
AA27	252049-14-2	CC(C)(C)C1=CC=C(C[C@@H](NC(=O)OCC2C3=CC=CC=C3C3=CC=CC=C23)C(O)=O)C=C1
AA28	220497-66-5	OC(=O)[C@H]1CC[C@H](C1)NC(=O)OCC1C2=CC=CC=C2C2=CC=CC=C12
AA29	144701-25-7	OC(=O)[C@@H](CC1CCCCC1)NC(=O)OCC1C2=CC=CC=C2C2=CC=CC=C12
AA30	180576-05-0	OC(=O)CN1CCN(CC1)C(=O)OCC1C2=CC=CC=C2C2=CC=CC=C12
AA31	1380327-56-9	OC(=O)C1(COC1)NC(=O)OCC1C2=CC=CC=C2C2=CC=CC=C12
AA32	180181-05-9	OC(=O)CC1CCN(CC1)C(=O)OCC1C2=CC=CC=C2C2=CC=CC=C12
AA33	2170726-27-7	OC(=O)[C@@H]1CC2(CC2)CN1C(=O)OCC1C2=CC=CC=C2C2=CC=CC=C12
AA34	193693-64-0	OC(=O)C1CN(C1)C(=O)OCC1C2=CC=CC=C2C2=CC=CC=C12

AA36	203866-19-7	OC(=O)[C@@H]1C[C@H](F)CN1C(=O)OCC1C2=CC=CC=C2C2=CC=CC=C12
AA37	185116-43-2	OC(=O)C1=CC=C(NC(=O)OCC2C3=CC=CC=C3C3=CC=CC=C23)C=C1
AA38	203866-21-1	OC(=O)[C@@H]1CC(F)(F)CN1C(=O)OCC1C2=CC=CC=C2C2=CC=CC=C12
AA39	220497-85-8	OC(=O)[C@@H](CC1=CC=CO1)NC(=O)OCC1C2=CC=CC=C2C2=CC=CC=C12
AA40	84891-19-0	OC(=O)[C@H](CC1=CN=CN1COCC1=CC=CC=C1)NC(=O)OCC1C2=CC=CC=C2C2=CC=CC=C12
AA41	126705-22-4	OC(=O)C1(CC1)NC(=O)OCC1C2=CC=CC=C2C2=CC=CC=C12
AA42	77128-70-2	CN(CC(=O)O)C(=O)OCC1C2=CC=CC=C2C3=CC=CC=C13
AA43	1197020-22-6	CCCCC[C@@H](C(=O)O)NC(=O)OCC1C2=CC=CC=C2C3=CC=CC=C13
AA44	478183-62-9	OC(C(CC1CCC1)NC(OCC2C(C=CC=C3)=C3C4=C2C=CC=C4)=O)=O
AA45	371770-32-0	C1CCC(C1)C[C@@H](C(=O)O)NC(=O)OCC2C3=CC=CC=C3C4=CC=CC=C24
AA46	86069-86-5	C1CCN([C@@H](C1)C(=O)O)C(=O)OCC2C3=CC=CC=C3C4=CC=CC=C24
AA47	35661-39-3	O=C(O)[C@H](C)NC(OCC1C2=CC=CC=C2C3=CC=CC=C13)=O
AA48	71989-33-8	O=C(O)[C@@H](NC(OCC1C(C=CC=C2)=C2C3=C1C=CC=C3)=O)COC(C)(C)C
AA49	71989-31-6	O=C(O)[C@H]1N(C(OCC2C3=CC=CC=C3C4=CC=CC=C24)=O)CCC1
AA50	71989-35-0	O=C(O)[C@@H](NC(OCC1C(C=CC=C2)=C2C3=C1C=CC=C3)=O)C(C)OC(C)(C)C
AA51	68858-20-8	O=C(O)[C@H](C(C)C)NC(OCC1C2=CC=CC=C2C3=CC=CC=C13)=O
AA52	132388-59-1	O=C(O)[C@H](CC(NC(C1=CC=CC=C1)(C2=CC=CC=C2)C3=CC=CC=C3)=O)NC(OCC4C(C=CC=C5)=C5C6=C4C=CC=C6)=O
AA53	71989-14-5	O=C(O)[C@@H](NC(OCC1C(C=CC=C2)=C2C3=C1C=CC=C3)=O)CC(OC(C)(C)C)=O
AA54	35661-60-0	O=C(O)[C@H](CC(C)C)NC(OCC1C2=CC=CC=C2C3=CC=CC=C13)=O
AA55	71989-18-9	O=C(O)[C@H](CCC(OC(C)(C)C)=O)NC(OCC1C2=CC=CC=C2C3=CC=CC=C13)=O
AA56	132327-80-1	O=C(O)[C@H](CCC(NC(C1=CC=CC=C1)(C2=CC=CC=C2)C3=CC=CC=C3)=O)NC(OCC4C(C=CC=C5)=C5C6=C4C=CC=C6)=O
AA57	71989-26-9	O=C(O)[C@@H](NC(OCC1C(C=CC=C2)=C2C3=C1C=CC=C3)=O)CCCCNC(OC(C)(C)C)=O
AA58	109425-51-6	O=C(O)[C@@H](NC(OCC1C(C=CC=C2)=C2C3=C1C=CC=C3)=O)CC4=CN(C(C5=CC=CC=C5)(C6=CC=CC=C6)C7=CC=CC=C7)C=N4
AA59	154445-77-9	O=C(O)[C@@H](NC(OCC1C(C=CC=C2)=C2C3=C1C=CC=C3)=O)CCCNC(NS(C4=C(C)C(CC(C)(C)O5)=C5C(C)=C4C)(=O)=O)=N
AA60	35661-40-6	O=C(O)[C@H](CC1=CC=CC=C1)NC(OCC2C3=CC=CC=C3C4=CC=CC=C24)=O
AA61	71989-38-3	O=C(O)[C@@H](NC(OCC1C(C=CC=C2)=C2C3=C1C=CC=C3)=O)CC4=CC=C(OC(C)(C)C)C=C4
AA62	143824-78-6	O=C(O)[C@@H](NC(OCC1C(C=CC=C2)=C2C3=C1C=CC=C3)=O)CC4=CN(C(OC(C)(C)C)=O)C5=C4C=CC=C5

Supplementary Table 13. Overview of aryl bromides used in SEL 3

Aryl bromide (AB)	Cas no	SMILES
AB1	1878-68-8	OC(=O)CC1=CC=C(Br)C=C1
AB2	1643-30-7	OC(=O)CCC1=CC=C(Br)C=C1
AB3	586-76-5	OC(=O)C1=CC=C(Br)C=C1
AB4	585-76-2	OC(=O)C1=CC(Br)=CC=C1
AB5	1006-41-3	OC(=O)C1=C(Br)C=C(F)C=C1
AB6	1013-83-8	OC(=O)C1=CC2=CC=CC(Br)=C2C=C1
AB7	99199-54-9	OC(=O)C1CCC2=CC(Br)=CC=C2O1
AB8	714207-41-7	OC(=O)C1CN(C(=O)C1)C1=CC=CC(Br)=C1
AB9	345965-52-8	OC(=O)C1(CC1)C1=CC=C(Br)C=C1
AB10	198561-04-5	OC(=O)[C@H](CC1=CC=C(Br)C=C1)NC(=O)OCC1C2=C(C=CC=C2)C2=C1C=CC=C2

Supplementary Table 14. Overview of boronic acids used in SEL 3

Boronic acid (BA)	Cas no	SMILES
BA1	5122-94-1	B(C1=CC=C(C=C1)C2=CC=CC=C2)(O)O
BA2	182344-13-4	B(C1=CC(=C(C=C1)O)Cl)(O)O
BA3	144432-85-9	B(C1=CC(=C(C=C1)F)Cl)(O)O
BA4	166316-48-9	B(C1=CC=C(C=C1)CCC(=O)O)(O)O
BA5	63503-60-6	B(C1=CC(=CC=C1)Cl)(O)O
BA6	3900-89-8	B(C1=CC=CC=C1Cl)(O)O
BA7	1679-18-1	B(C1=CC=C(C=C1)Cl)(O)O
BA8	126747-14-6	B(C1=CC=C(C=C1)C#N)(O)O
BA9	151169-75-4	B(C1=CC(=C(C=C1)Cl)Cl)(O)O
BA10	68716-47-2	B(C1=C(C=C(C=C1)Cl)Cl)(O)O
BA11	67492-50-6	B(C1=CC(=CC(=C1)Cl)Cl)(O)O
BA12	168267-41-2	B(C1=CC(=C(C=C1)F)F)(O)O
BA13	156545-07-2	B(C1=CC(=CC(=C1)F)F)(O)O
BA14	122775-35-3	B(C1=CC(=C(C=C1)OC)OC)(O)O
BA15	133730-34-4	B(C1=C(C=C(C=C1)OC)OC)(O)O
BA16	1765-93-1	B(C1=CC=C(C=C1)F)(O)O
BA17	768-35-4	B(C1=CC(=CC=C1)F)(O)O
BA18	1993-03-9	OB(O)C1=CC=C(F)C=C1
BA19	87199-18-6	B(C1=CC(=CC=C1)O)(O)O
BA20	71597-85-8	B(C1=CC=C(C=C1)O)(O)O
BA21	153624-46-5	B(C1=CC=C(C=C1)OC(C)C)(O)O
BA22	99769-19-4	B(C1=CC(=CC=C1)C(=O)OC)(O)O
BA23	99768-12-4	B(C1=CC=C(C=C1)C(=O)OC)(O)O
BA24	374538-03-1	B(C1=CC=CC=C1C(=O)OC)(O)O
BA25	5720-06-9	COC1=CC=CC=C1B(O)O
BA26	10365-98-7	B(C1=CC(=CC=C1)OC)(O)O
BA27	5720-07-0	B(C1=CC=C(C=C1)OC)(O)O
BA28	5570-19-4	B(C1=CC=CC=C1[N+](=O)[O-])(O)O
BA29	24067-17-2	B(C1=CC=C(C=C1)[N+](=O)[O-])(O)O

BA30	13331-27-6	B(C1=CC(=CC=C1)[N+](=O)[O-])(O)O
BA31	98-80-6	B(C1=CC=CC=C1)(O)O
BA32	16419-60-6	B(C1=CC=CC=C1C)(O)O
BA33	17933-03-8	B(C1=CC(=CC=C1)C)(O)O
BA34	5720-05-8	COC1=CC=CC=C1B(O)O
BA35	139301-27-2	B(C1=CC=C(C=C1)OC(F)(F)F)(O)O
BA36	219735-99-6	B(C1=C(C=C(C=C1)OC)Cl)(O)O
BA37	138008-97-6	CC(OC1=CC=CC=C1B(O)O)C
BA38	175676-65-0	FC(F)(F)OC1=CC=CC=C1B(O)O
BA39	63139-21-9	B(C1=CC=C(C=C1)CC)(O)O
BA40	352535-82-1	B(C1=C(C(=CC=C1)Cl)F)(O)O
BA41	149507-26-6	B(C1=CC(=C(C=C1)OC)F)(O)O
BA42	208399-66-0	B(C1=C(C=C(C=C1)OC)C)(O)O
BA43	175883-62-2	B(C1=CC(=C(C=C1)OC)C)(O)O
BA44	22237-13-4	CCOC1=CC=C(B(O)O)C=C1
BA45	170981-26-7	CC1=CC=C(B(O)O)C(F)=C1
BA46	55499-43-9	CC1=CC=C(B(O)O)C=C1C
BA47	168267-99-0	CC1=CC=C(B(O)O)C=C1F
BA48	121219-16-7	FC1=C(F)C(B(O)O)=CC=C1
BA49	1423-27-4	B(C1=CC=CC=C1C(F)(F)F)(O)O
BA50	226396-32-3	B(C1=C(C(=C(C=C1)F)F)F)(O)O
BA51	279262-11-2	B(C1=CC=C(C=C1)COC)(O)O
BA52	145240-28-4	B(C1=CC=C(C=C1)CCCC)(O)O
BA53	208516-15-8	OB(C1=CC=C(S(=O)(NC(C)(C)C)=O)C=C1)O

Supplementary Table 15. Overview of amino acids used in SEL 4

Amino acid (AA)	Cas no	SMILES
AA1	852288-18-7	CC1(C)OC2=CC=C(C[C@H](NC(=O)OCC3C4=CC=CC=C4C4=CC=CC=C34)C(O)=O)C=C2O1
AA6	368866-30-2	OC(=O)C1(CCN(CC2=CC=CC=C2)CC1)NC(=O)OCC1C2=CC=CC=C2C2=CC=CC=C12
AA10	185379-39-9	OC(=O)[C@@H](CC1=CC=CC=N1)NC(=O)OCC1C2=CC=CC=C2C2=CC=CC=C12
AA12	198560-38-2	OC(=O)[C@@H]1CC2=CC=CC=C2N1C(=O)OCC1C2=CC=CC=C2C2=CC=CC=C12
AA15	204318-02-5	OC(=O)[C@@H]1CCCN1C1CCN(CC1)C(=O)OCC1C2=CC=CC=C2C2=CC=CC=C12
AA18	167690-53-1	OC(=O)[C@H]1CC[C@H](CNC(=O)OCC2C3=CC=CC=C3C3=CC=CC=C23)CC1
AA20	1335206-44-4	OC(=O)COC[C@H]1CCCN1C(=O)OCC1C2=CC=CC=C2C2=CC=CC=C12
AA30	180576-05-0	OC(=O)CN1CCN(CC1)C(=O)OCC1C2=CC=CC=C2C2=CC=CC=C12
AA33	2170726-27-7	OC(=O)[C@@H]1CC2(CC2)CN1C(=O)OCC1C2=CC=CC=C2C2=CC=CC=C12
AA38	203866-21-1	OC(=O)[C@@H]1CC(F)(F)CN1C(=O)OCC1C2=CC=CC=C2C2=CC=CC=C12
AA47	35661-39-3	O=C(O)[C@H](C)NC(OCC1C2=CC=CC=C2C3=CC=CC=C13)=O
AA48	71989-33-8	O=C(O)[C@@H](NC(OCC1C(C=CC=C2)=C2C3=C1C=CC=C3)=O)COC(C)(C)C

AA53	71989-14-5	O=C(O)[C@@H](NC(OCC1C(C=CC=C2)=C2C3=C1C=CC=C3)=O)CC(OC(C)(C)C)=O
AA54	35661-60-0	O=C(O)[C@H](CC(C)C)NC(OCC1C2=CC=CC=C2C3=CC=CC=C13)=O
AA58	109425-51-6	O=C(O)[C@@H](NC(OCC1C(C=CC=C2)=C2C3=C1C=CC=C3)=O)CC4=CN(C(C5=CC=CC=C5)(C6=CC=CC=C6)C7=CC=CC=C7)C=N4
AA59	154445-77-9	O=C(O)[C@@H](NC(OCC1C(C=CC=C2)=C2C3=C1C=CC=C3)=O)CCCNC(NS(C4=C(C)C(CC(C)(C)O5)=C5C(C)=C4C)(=O)=O)=N
AA60	35661-40-6	O=C(O)[C@H](CC1=CC=CC=C1)NC(OCC2C3=CC=CC=C3C4=CC=CC=C24)=O
AA62	143824-78-6	O=C(O)[C@@H](NC(OCC1C(C=CC=C2)=C2C3=C1C=CC=C3)=O)CC4=CN(C(OC(C)(C)C)=O)C5=C4C=CC=C5
AA63	155369-11-2	OC(=O)c1cccc(CNC(=O)OCC2c3ccccc3-c4ccccc24)c1
AA64	135673-97-1	N([C@@H](CC4CCCCC4)C(=O)O)C(=O)OCC1c2c(cccc2)c3c1cccc3

Supplementary Table 16. Overview of carboxylic acids used in SEL 4

Carboxylic acid (CA)	Cas no	SMILES
CA17	530-57-4	COC1=CC(=CC(=C1O)OC)C(=O)O
CA31	28440-13-3	CC1(O[C@H]2[C@@H](O1)[C@@H](O[C@@H]2C(=O)O)n3cnc4c3nc[nH]c4=O)C
CA76	186046-78-6	O=C1N(CC(O)=O)C=CC(NC(OC(C2=CC=CC=C2)C3=CC=CC=C3)=O)=N1
CA107	2207-75-2	c1(nc(=O)[nH]c(=O)[nH]1)C(=O)[O-].[K+]
CA117	65-86-1	c1c([nH]c(=O)[nH]c1=O)C(=O)O
CA124	121-34-6	COc1c(ccc(c1)C(=O)O)O
CA128	20924-05-4	Cc1cn(c(=O)[nH]c1=O)CC(=O)O
CA130	1028077-12-4	CC(C)(C)OC(=O)NC1=NC2=C(N=CN2CC(O)=O)C(=O)N1
CA131	-	O=C(C1=CC=CC=C1N2CC(O)=O)N(OC(C)(C)C)C2=O
CA132	-	O=C1N(OC(C)(C)C)C(C2=CC=C(C(O)=O)C=C21)=O

Methods references:

1. Rappsilber, J., Mann, M. & Ishihama, Y. Protocol for micro-purification, enrichment, pre-fractionation and storage of peptides for proteomics using StageTips. *Nature Protocols 2007 2:8* **2**, 1896–1906 (2007).

2. Chambers, M. C. *et al.* A cross-platform toolkit for mass spectrometry and proteomics. *Nat Biotechnol* **30**, 918–920 (2012).

3. Heinonen, M. *et al.* FiD: a software for ab initio structural identification of product ions from tandem mass spectrometric data. *Rapid Communications in Mass Spectrometry* **22**, 3043–3052 (2008).

4. Wolf, S., Schmidt, S., Müller-Hannemann, M. & Neumann, S. In silico fragmentation for computer assisted identification of metabolite mass spectra. *BMC Bioinformatics* **11**, 1–12 (2010).

5. Hill, A. W. & Mortishire-Smith, R. J. Automated assignment of high-resolution collisionally activated dissociation mass spectra using a systematic bond disconnection approach. *Rapid Communications in Mass Spectrometry* **19**, 3111–3118 (2005).

6. Ridder, L. *et al.* Substructure-based annotation of high-resolution multistage MSn spectral trees. *Rapid Communications in Mass Spectrometry* **26**, 2461–2471 (2012).

7. Allen, F., Pon, A., Wilson, M., Greiner, R. & Wishart, D. CFM-ID: a web server for annotation, spectrum prediction and metabolite identification from tandem mass spectra. *Nucleic Acids Res* **42**, W94–W99 (2014).

8. Mohimani, H. *et al.* Dereplication of microbial metabolites through database search of mass spectra. *Nat Commun* **9**, 1–12 (2018).

9. Dührkop, K. *et al.* SIRIUS 4: a rapid tool for turning tandem mass spectra into metabolite structure information. *Nature Methods 2019 16:4* **16**, 299–302 (2019).

10. Rauf, I., Rasche, F., Nicolas, F. & Böcker, S. Finding Maximum Colorful Subtrees in Practice. *Journal of Computational Biology* **20**, 311–321 (2013).

11. Dührkop, K., Lataretu, M. A., White, W. T. J. & Böcker, S. Heuristic algorithms for the Maximum Colorful Subtree problem. *Leibniz International Proceedings in Informatics, LIPIcs* **113**, 23:1-23:14 (2018).

11. Supplementary NMR data

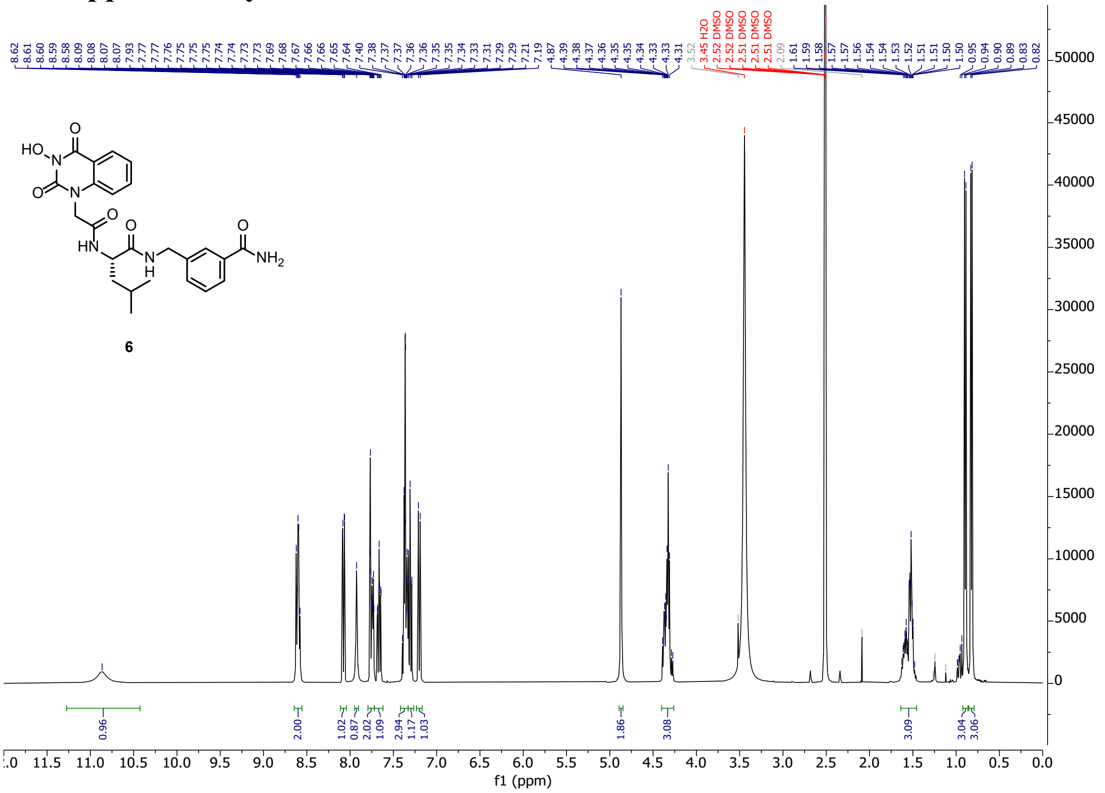

Supplementary Fig 45. ^{1}H **NMR spectrum of compound 6 (DMSO, 400 MHz).**

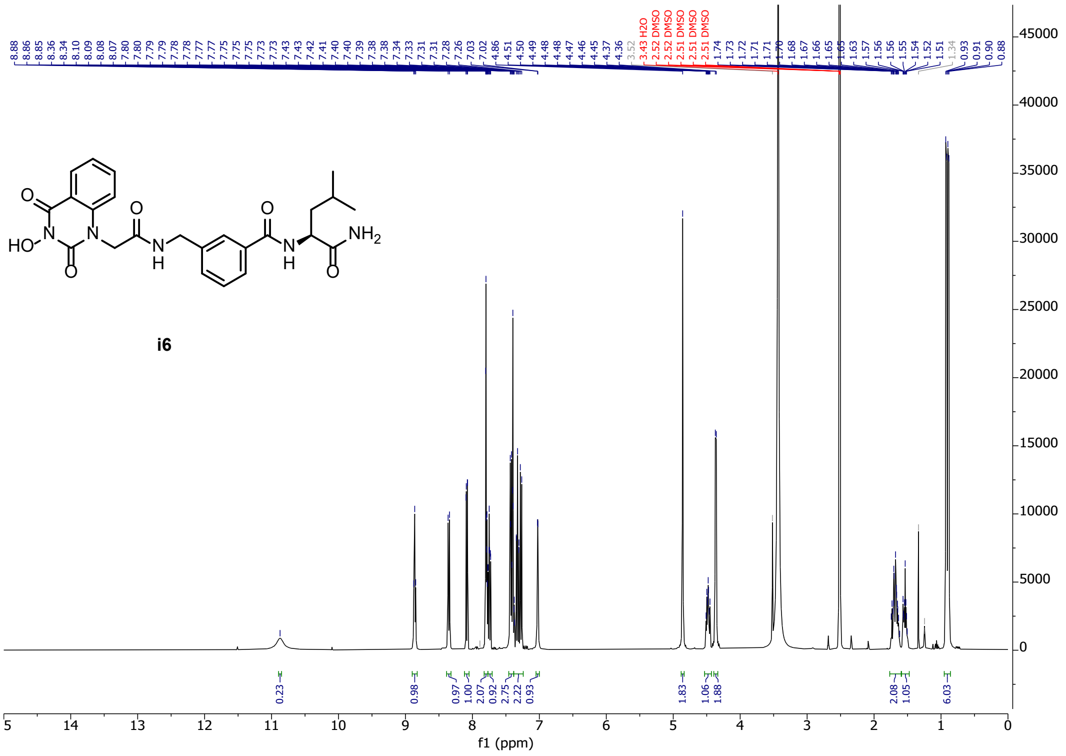

Supplementary Fig 46. ^{1}H **NMR spectrum of compound i6 (DMSO, 400 MHz).**

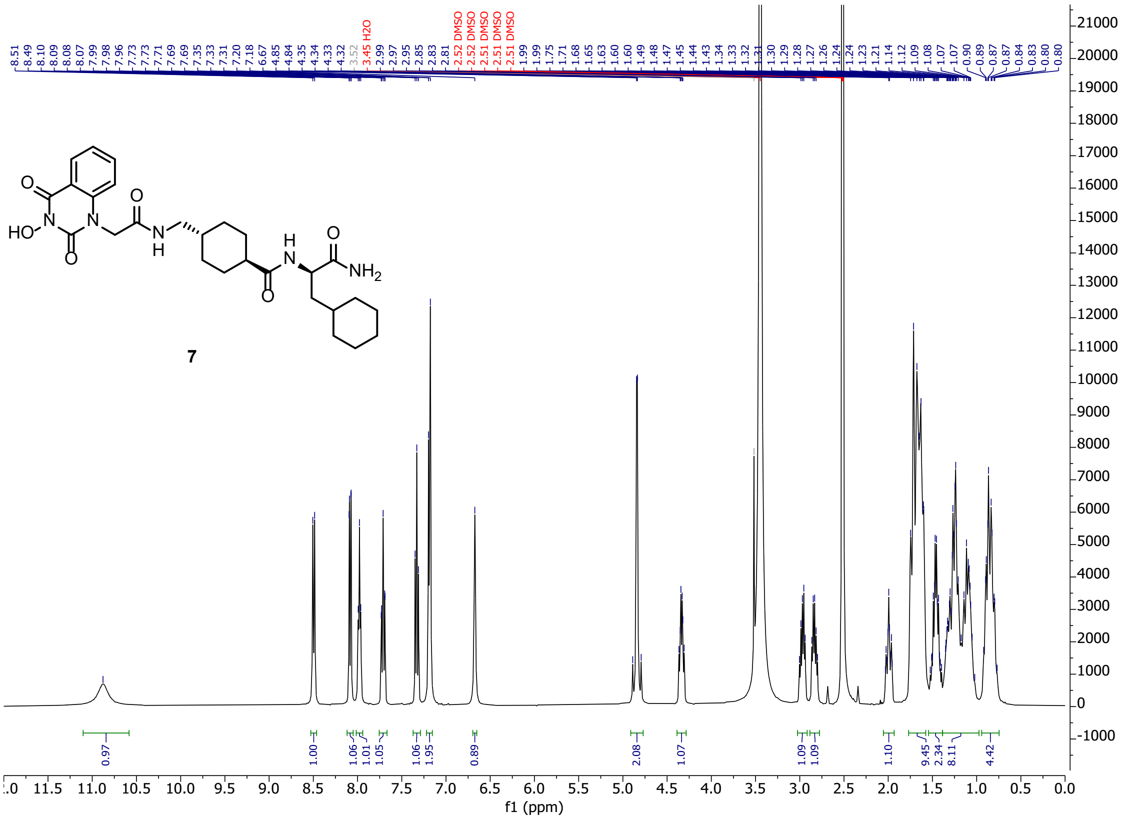

Supplementary Fig 47. ^{1}H NMR spectrum of compound 7 (DMSO, 400 MHz).

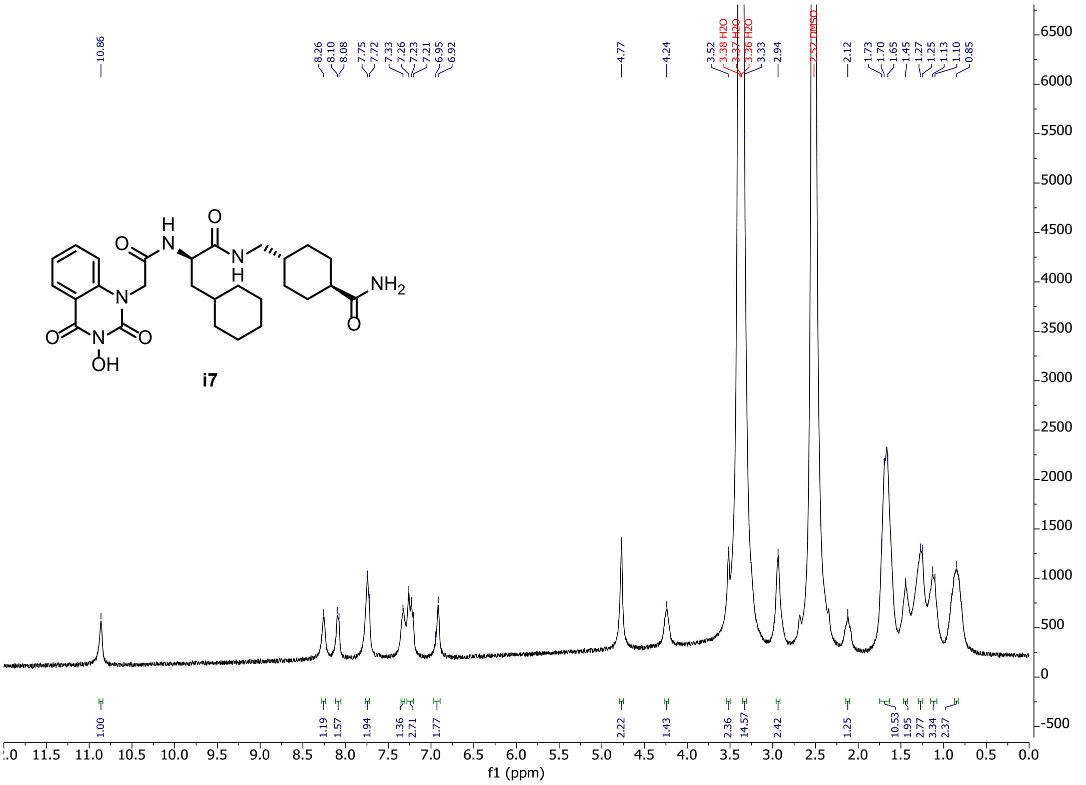

Supplementary Fig 48. ^{1}H NMR spectrum of compound i7 (DMSO, 400 MHz).

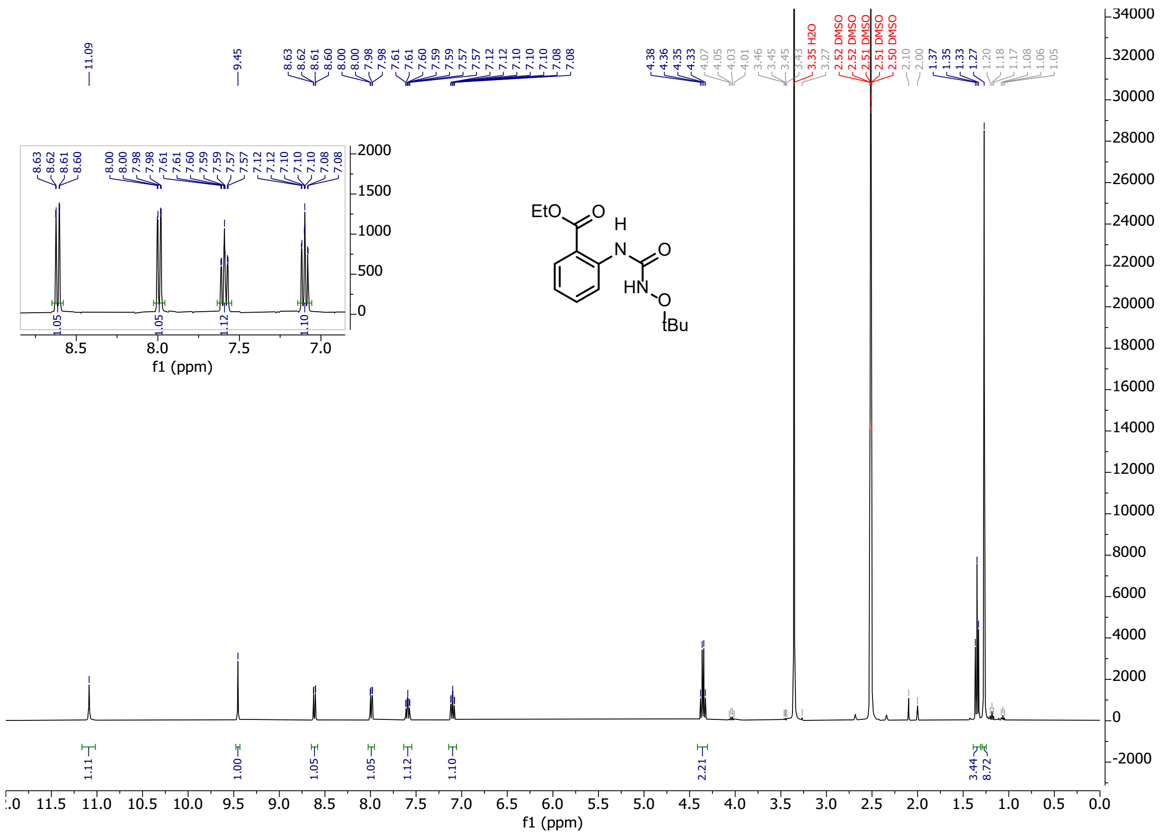

Supplementary Fig 49. ^{1}H NMR spectrum of compound 21 (DMSO, 400 MHz).

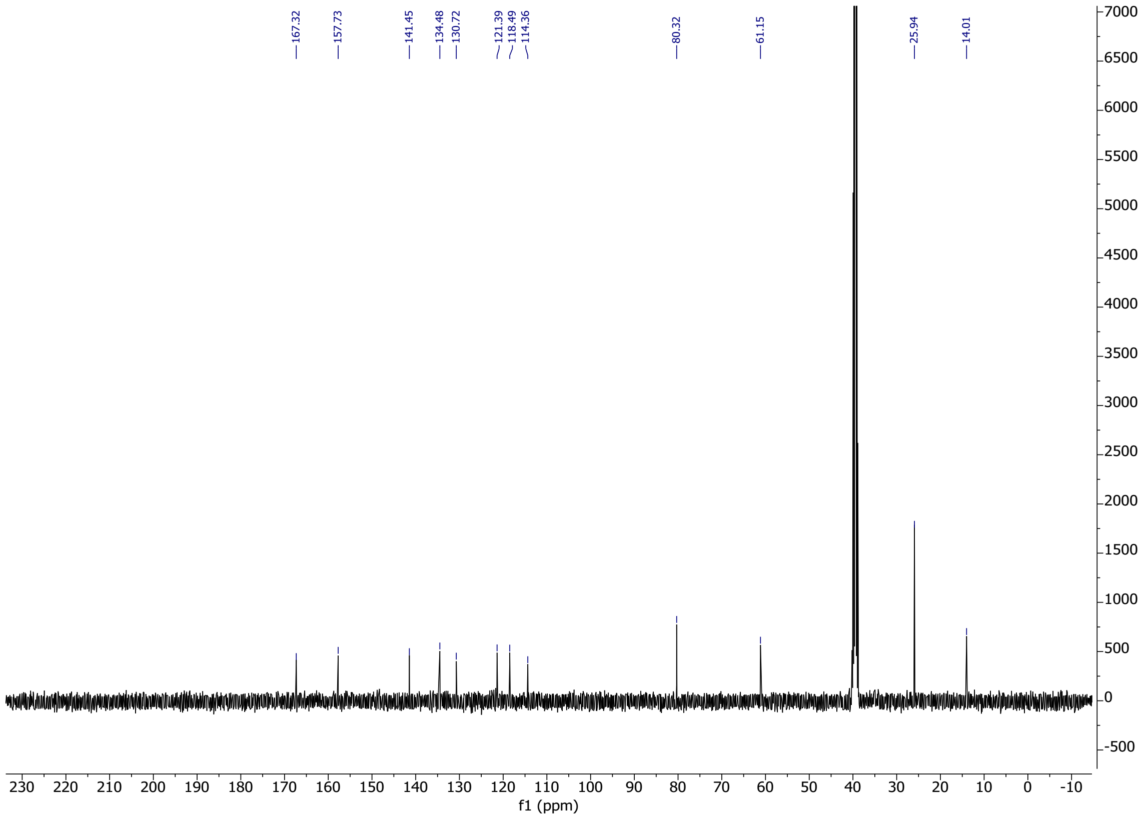

Supplementary Fig 50. ^{13}C NMR spectrum of compound 21 (DMSO, 400 MHz).

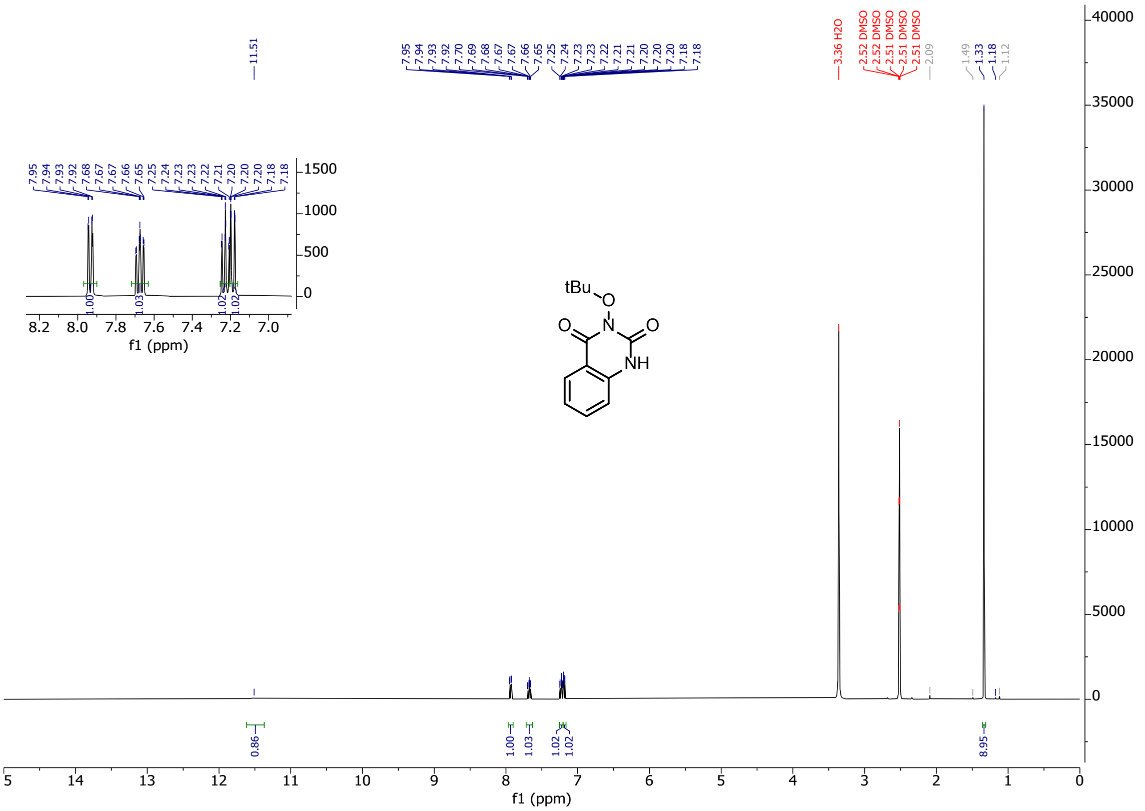

Supplementary Fig 51. ^{1}H NMR spectrum of compound 22 (DMSO, 400 MHz).

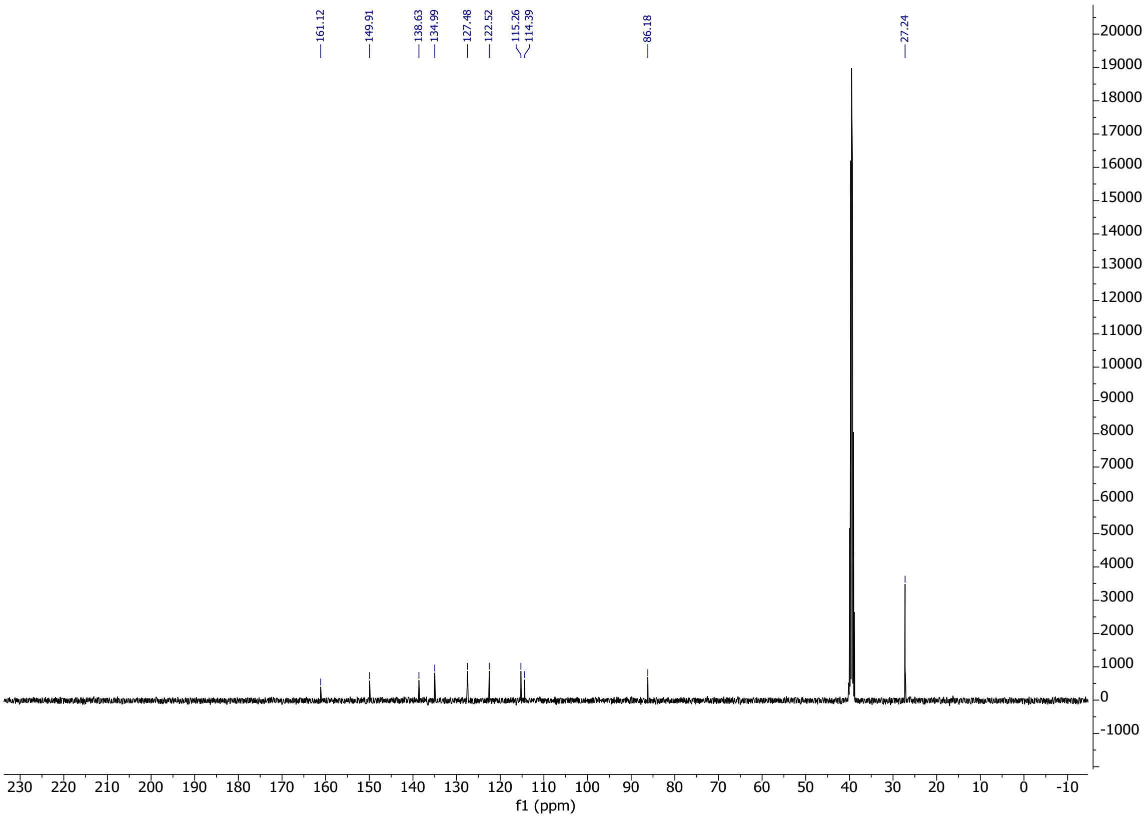

Supplementary Fig 52. ^{13}C NMR spectrum of compound 22 (DMSO, 400 MHz).

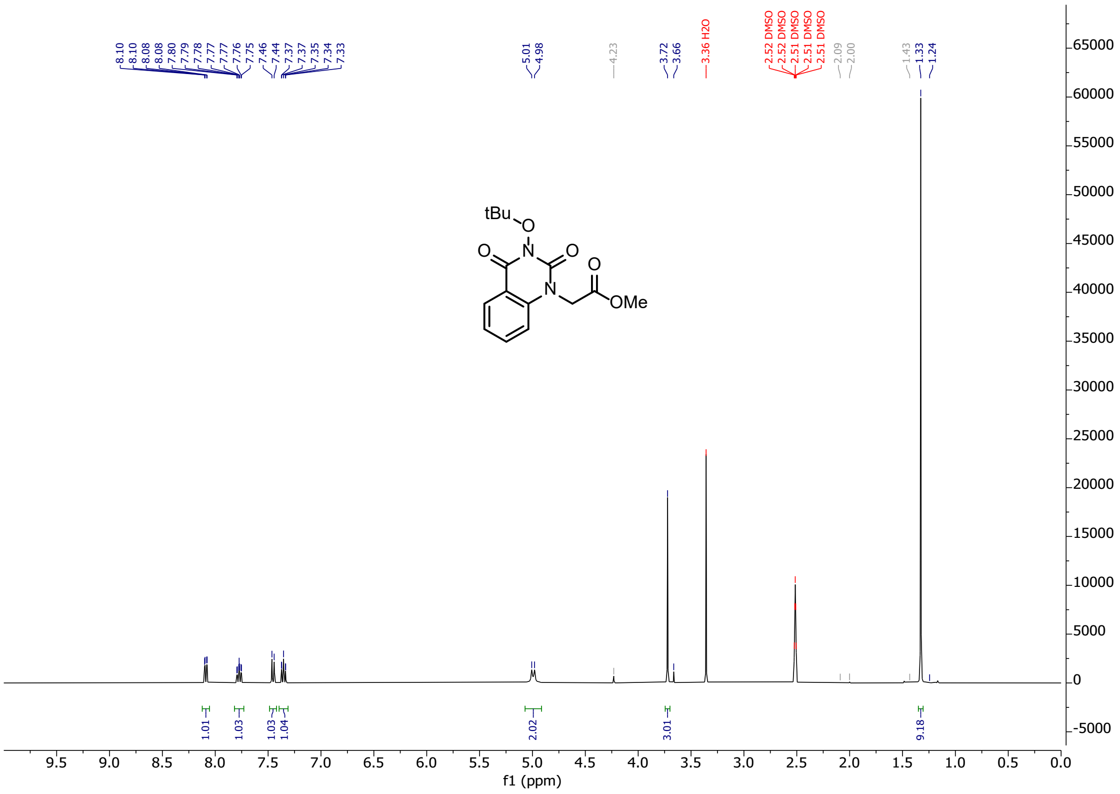

Supplementary Fig 53. ^{1}H NMR spectrum of compound 23 (DMSO, 400 MHz).

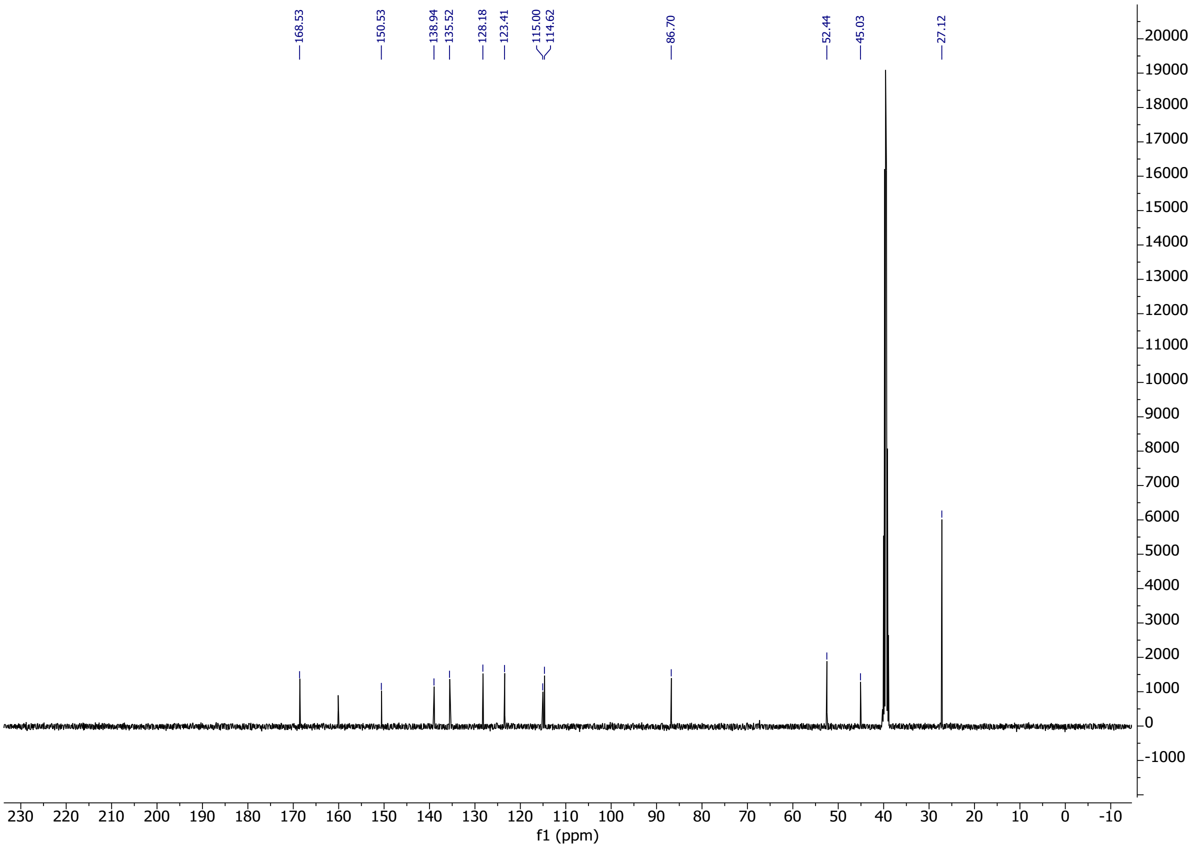

Supplementary Fig 54. ^{13}C NMR spectrum of compound 23 (DMSO, 400 MHz).

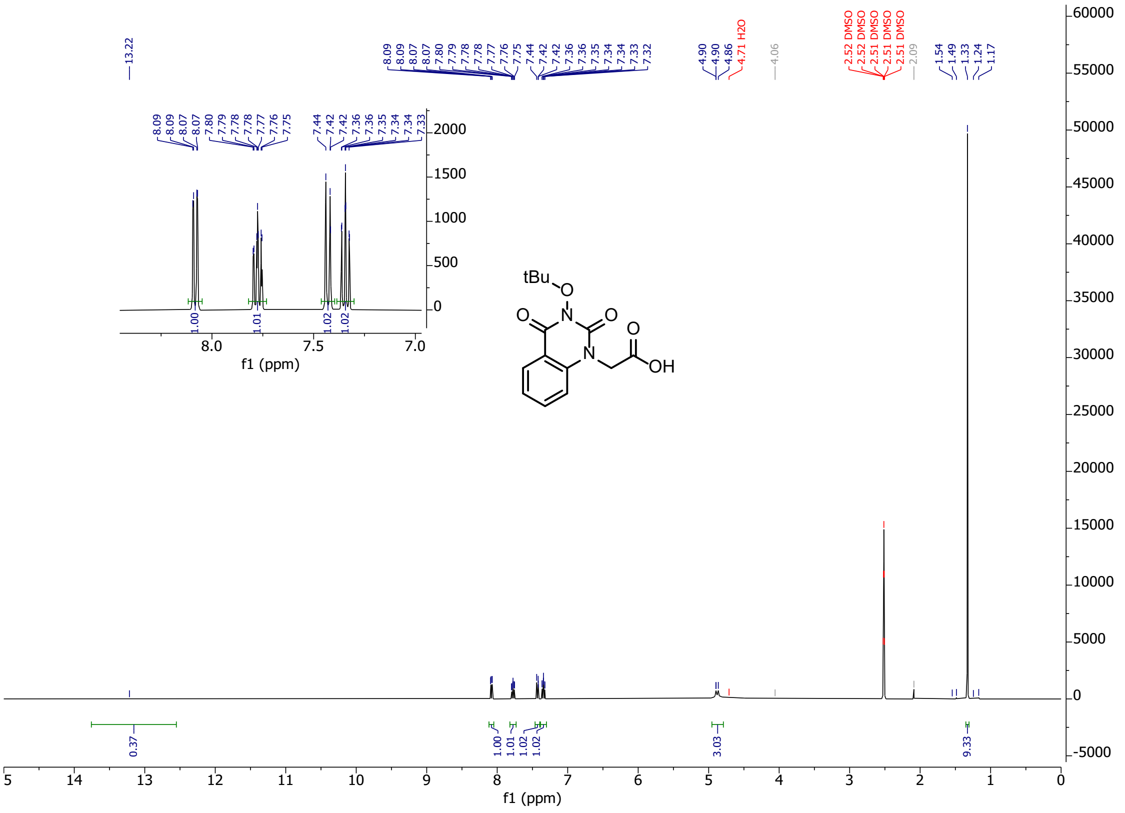

Supplementary Fig 55. ^{1}H NMR spectrum of compound 24 (DMSO, 400 MHz).

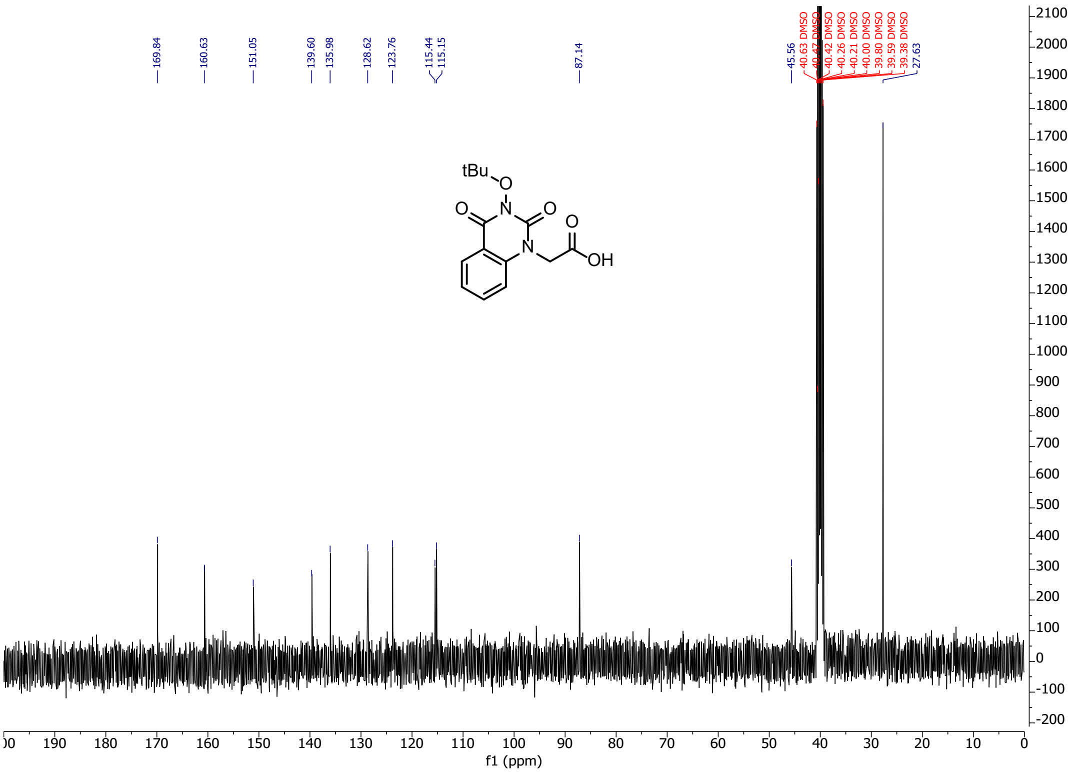

Supplementary Fig 56. ^{13}C NMR spectrum of compound 24 (DMSO, 400 MHz).

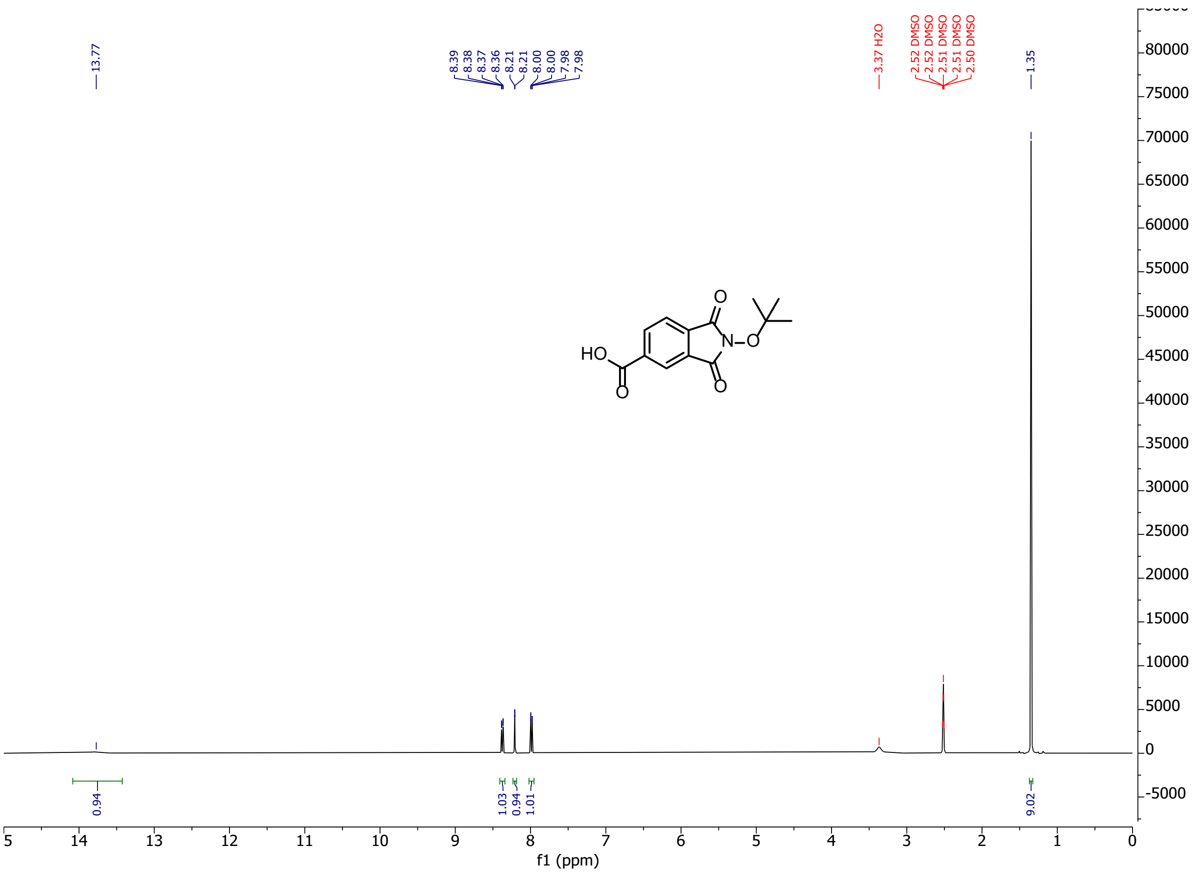

Supplementary Fig 57. ^{1}H NMR spectrum of compound 25 (DMSO, 400 MHz).

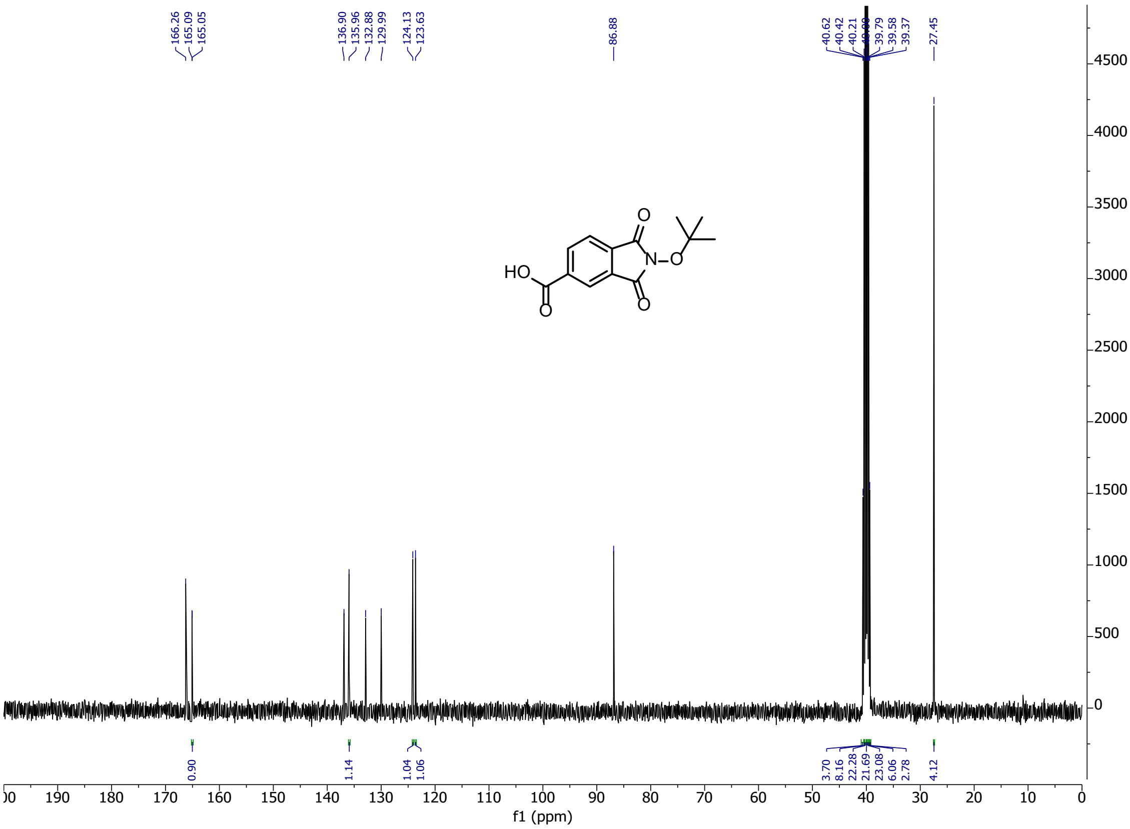

Supplementary Fig 58. ^{13}C NMR spectrum of compound 25 (DMSO, 400 MHz).

12. Supplementary LC-MS Data

12.1 LC-MS data from the scope of primary amines

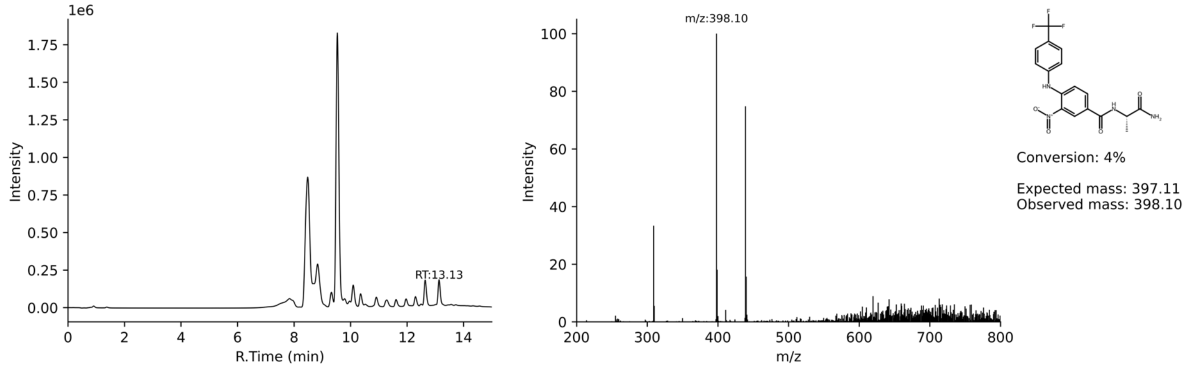

Supplementary Fig 59. LC-MS chromatogram obtained using 4-trifluoromethylaniline.

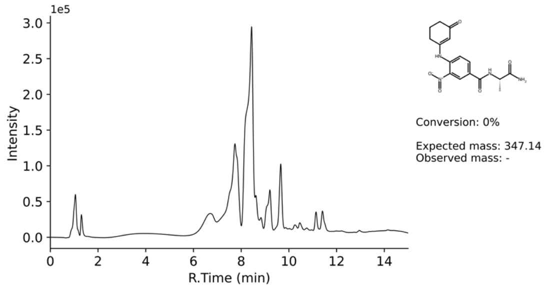

Supplementary Fig 60. LC-MS chromatogram obtained using 3-amino-2-cyclohexen-1-one.

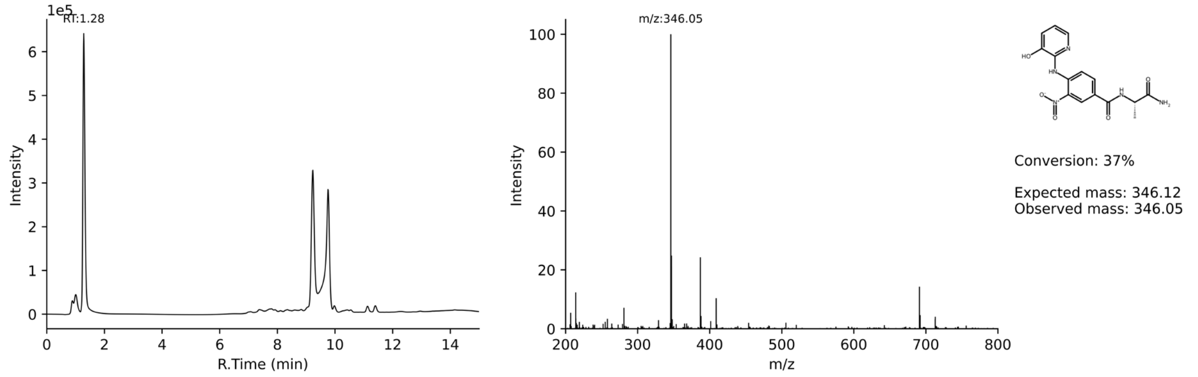

Supplementary Fig 61. LC-MS chromatogram obtained using 2-amino-3-hydroxy pyridine.

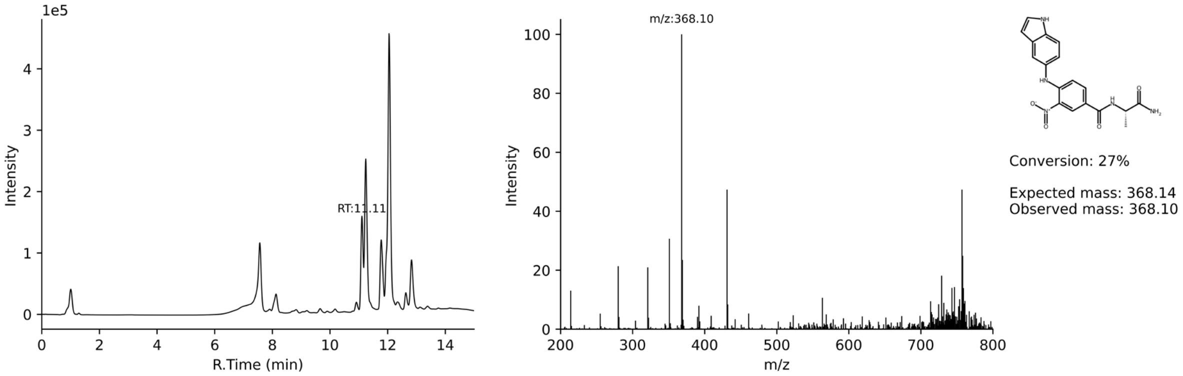

Supplementary Fig 62. LC-MS chromatogram obtained using 5-aminoindole.

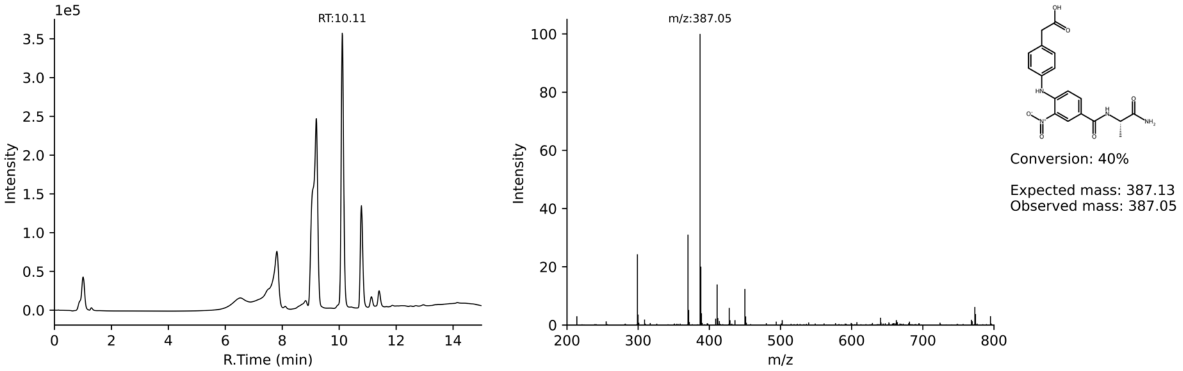

Supplementary Fig 63. LC-MS chromatogram obtained using 4-aminophenylacetic acid.

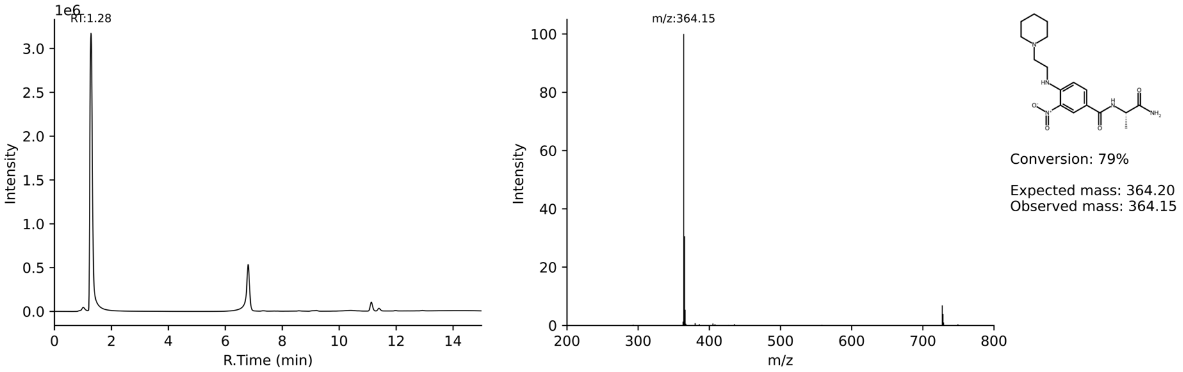

Supplementary Fig 64. LC-MS chromatogram obtained using 1-(2-aminoethyl)piperidine.

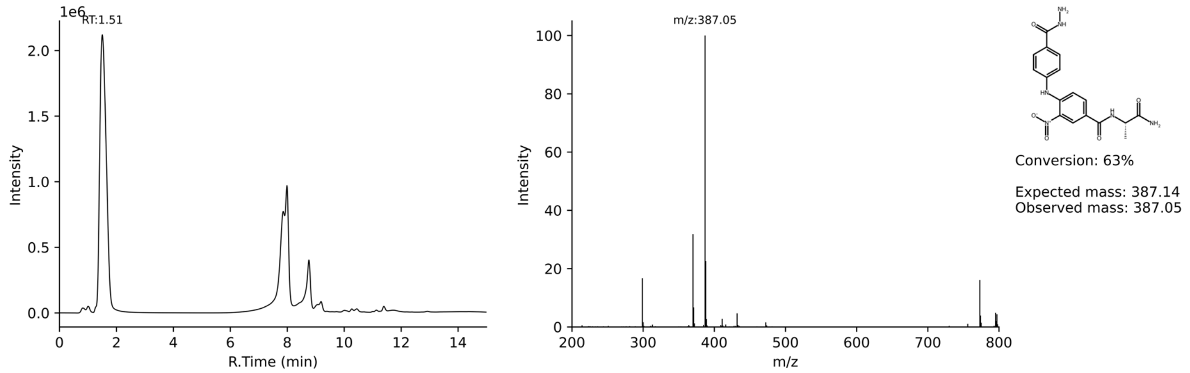

Supplementary Fig 65. LC-MS chromatogram obtained using 4-aminobenzoyl hydrazide.

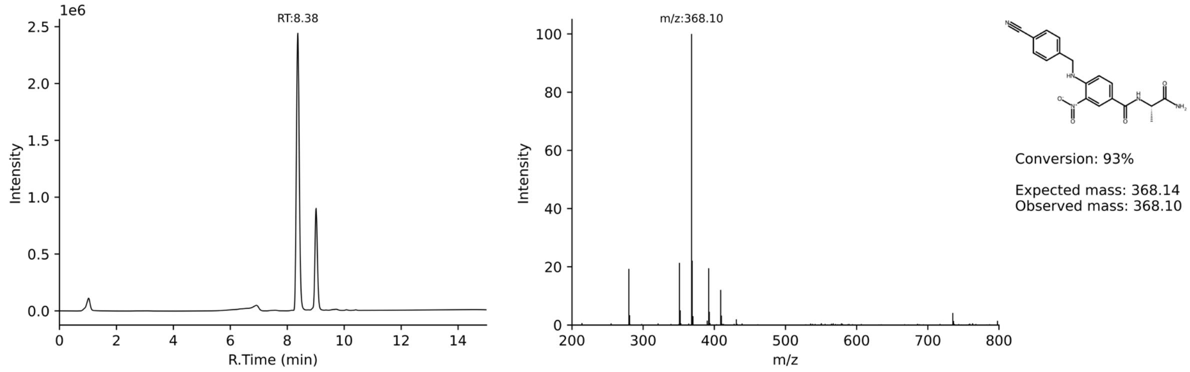

Supplementary Fig 66. LC-MS chromatogram obtained using 4-(aminomethyl)benzonitrile hydrochloride.

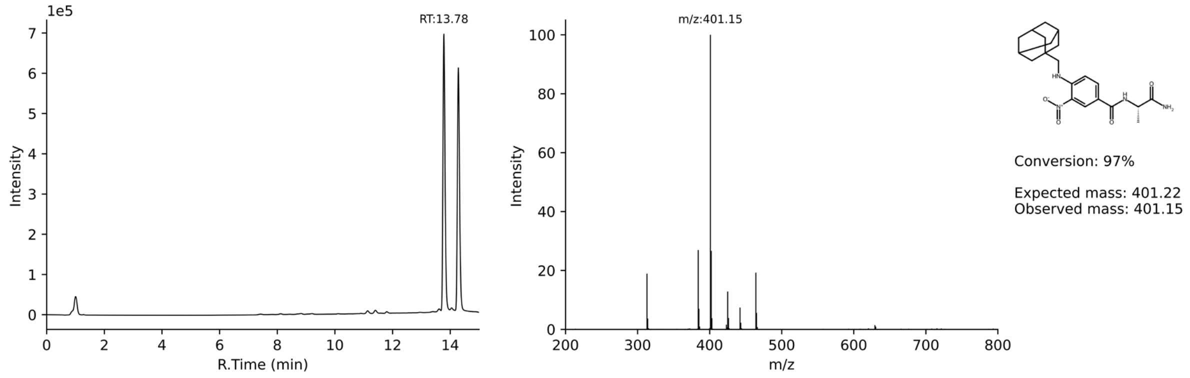

Supplementary Fig 67. LC-MS chromatogram obtained using 1-adamanthanemethylamine.

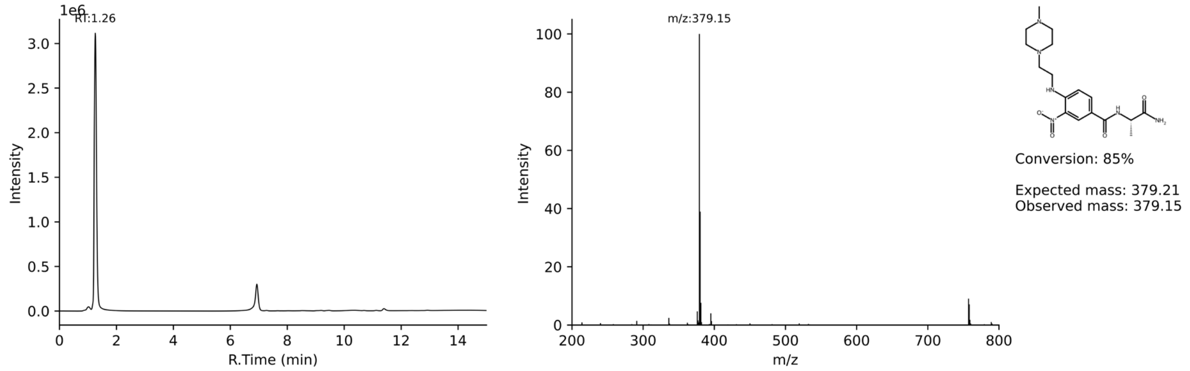

Supplementary Fig 68. LC-MS chromatogram obtained using 1-(2-aminoethyl)-4-methyl-piperazine.

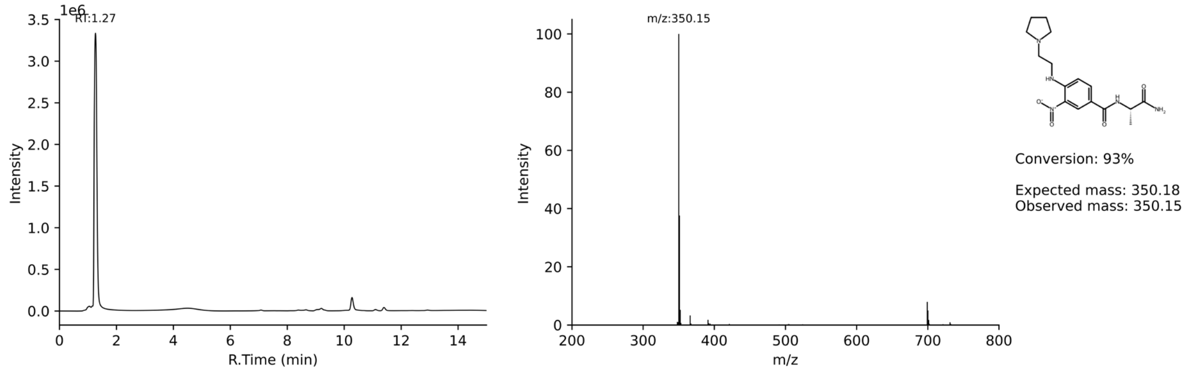

Supplementary Fig 69. LC-MS chromatogram obtained using 1(2-aminoethyl)pyrrolidine.

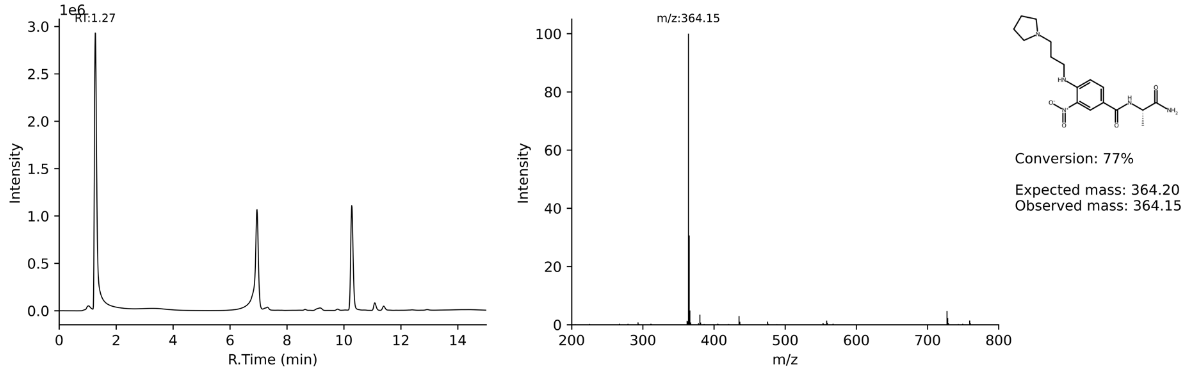

Supplementary Fig 70. LC-MS chromatogram obtained using 1-(3-aminopropyl)pyrrolidine.

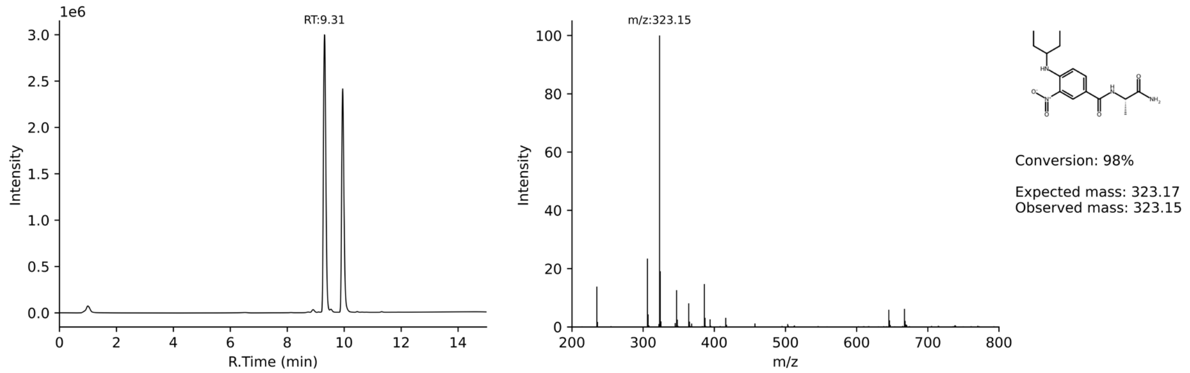

Supplementary Fig 71. LC-MS chromatogram obtained using 3-aminopentane.

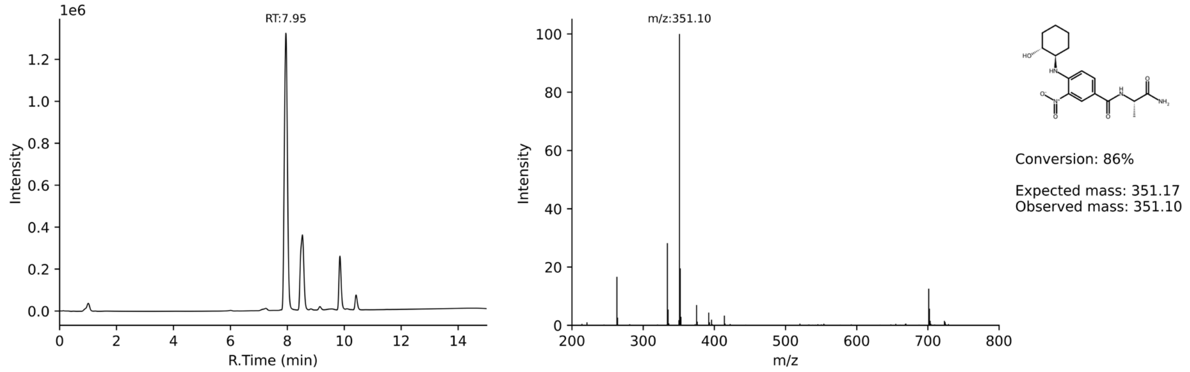

Supplementary Fig 72. LC-MS chromatogram obtained using trans-2-aminocyclohexanol.

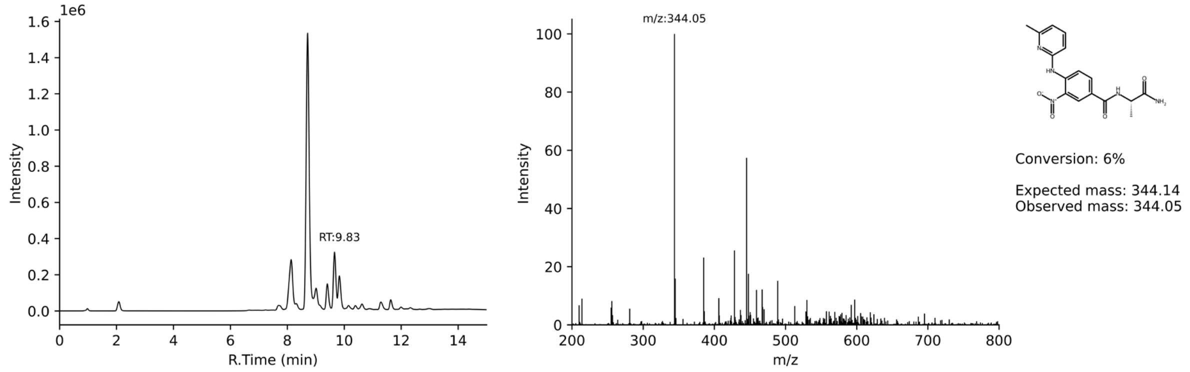

Supplementary Fig 73. LC-MS chromatogram obtained using 6-methyl-2-pyridylamine.

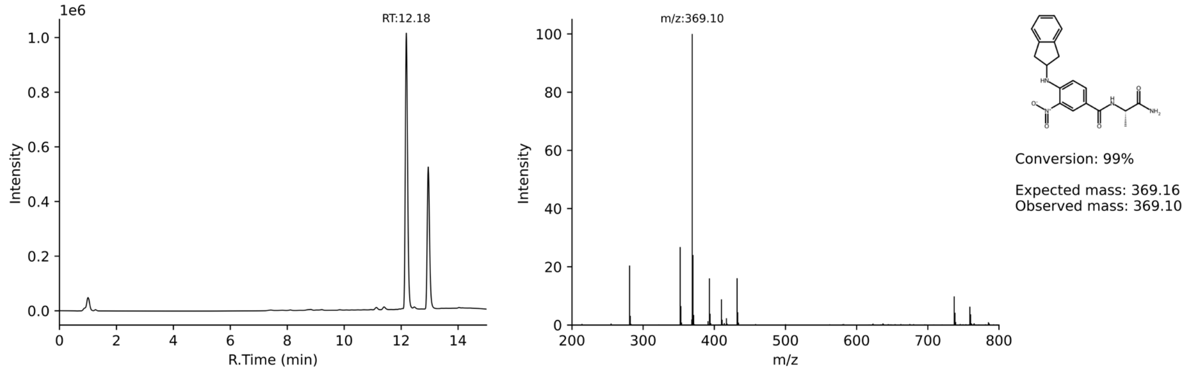

Supplementary Fig 74. LC-MS chromatogram obtained using 2-aminoindane.

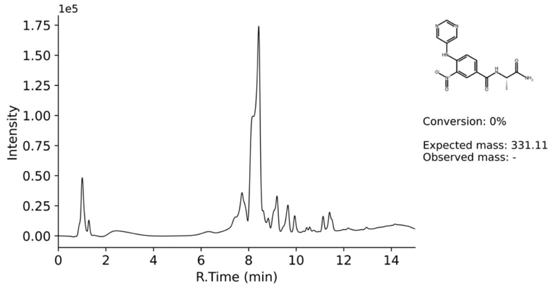

Supplementary Fig 75. LC-MS chromatogram obtained using 5-aminopyrimidine.

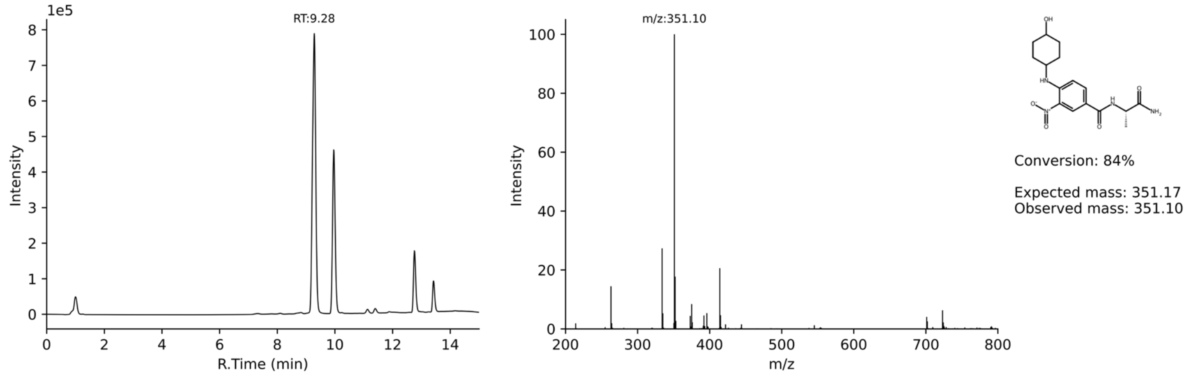

Supplementary Fig 76. LC-MS chromatogram obtained using trans-4-aminocyclohexanol.

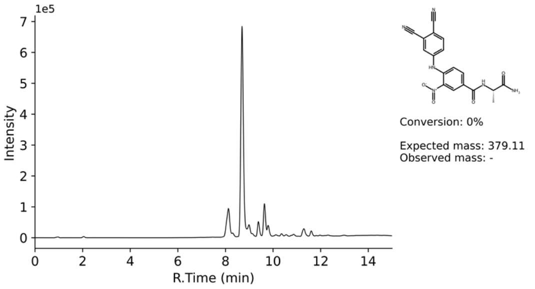

Supplementary Fig 77. LC-MS chromatogram obtained using 4-aminophthalonitrile.

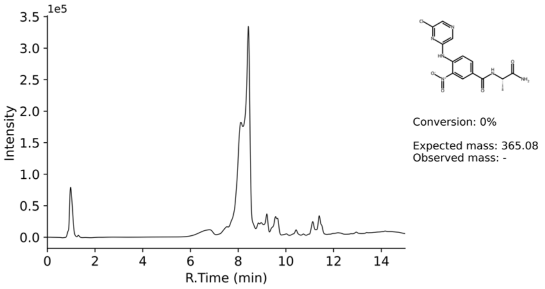

Supplementary Fig 78. LC-MS chromatogram obtained using 2-amino-6-chloropyrazine.

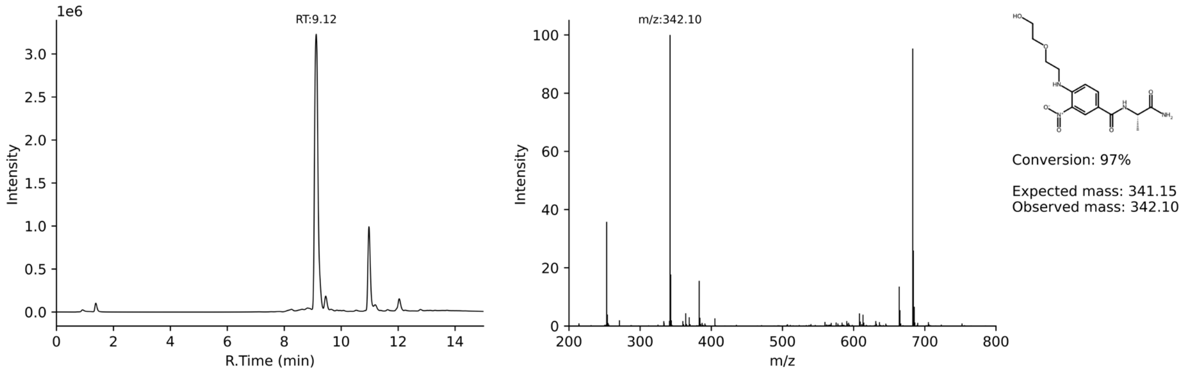

Supplementary Fig 79. LC-MS chromatogram obtained using 2(2-aminoethoxy)ethanol.

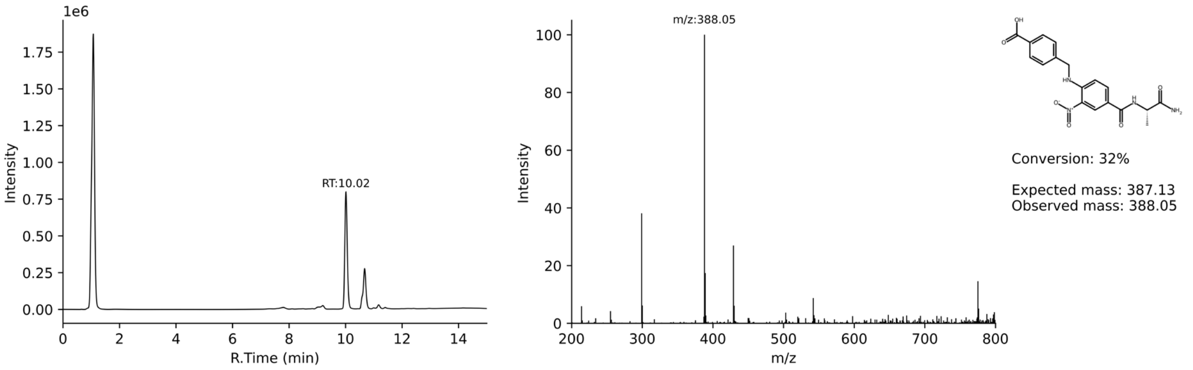

Supplementary Fig 80. LC-MS chromatogram obtained using 4-(aminomethyl)benzoic acid.

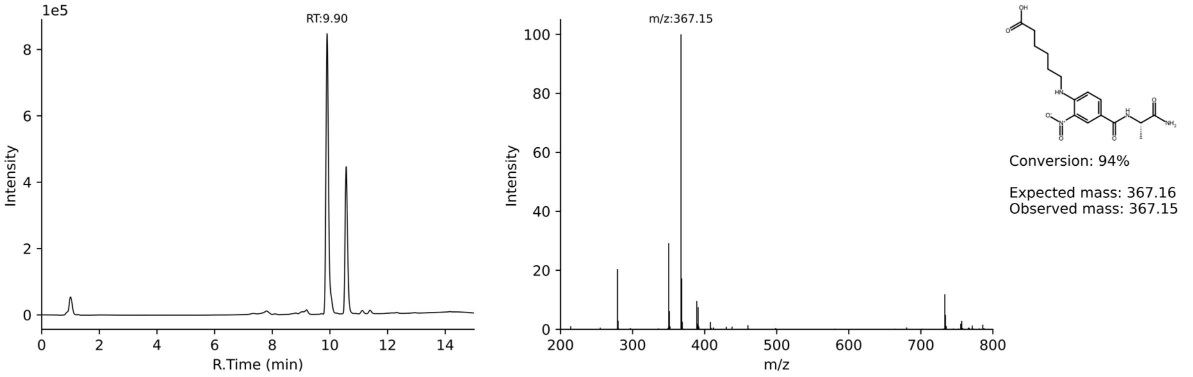

Supplementary Fig 81. LC-MS chromatogram obtained using 6-aminohexanoic acid.

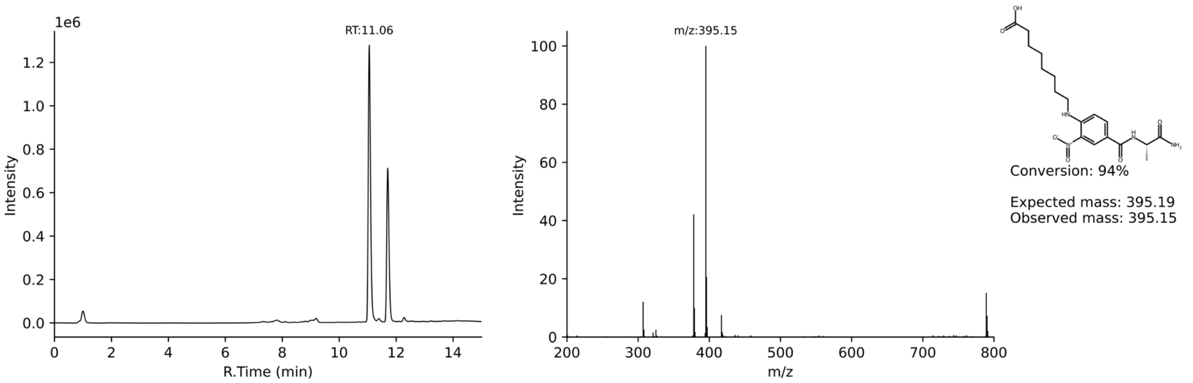

Supplementary Fig 82. LC-MS chromatogram obtained using 8-aminooctanoic acid.

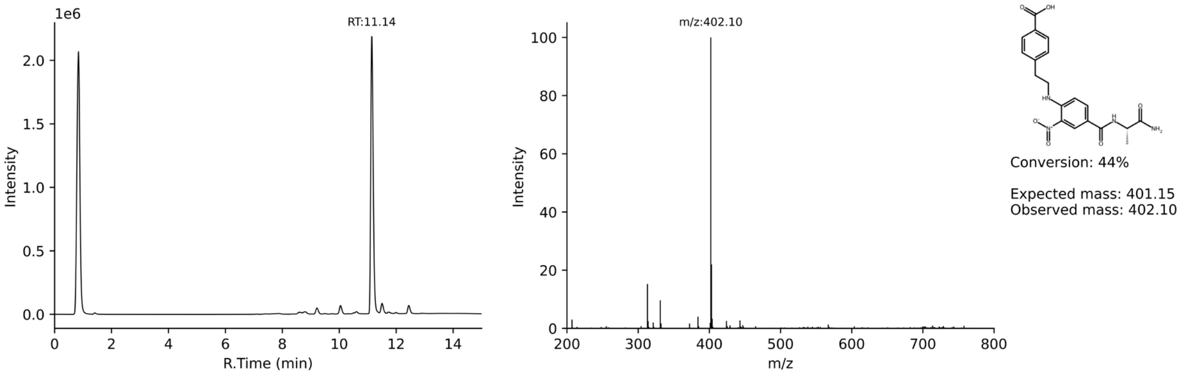

Supplementary Fig 83. LC-MS chromatogram obtained using 4-(2-aminoethyl)benzoic acid.

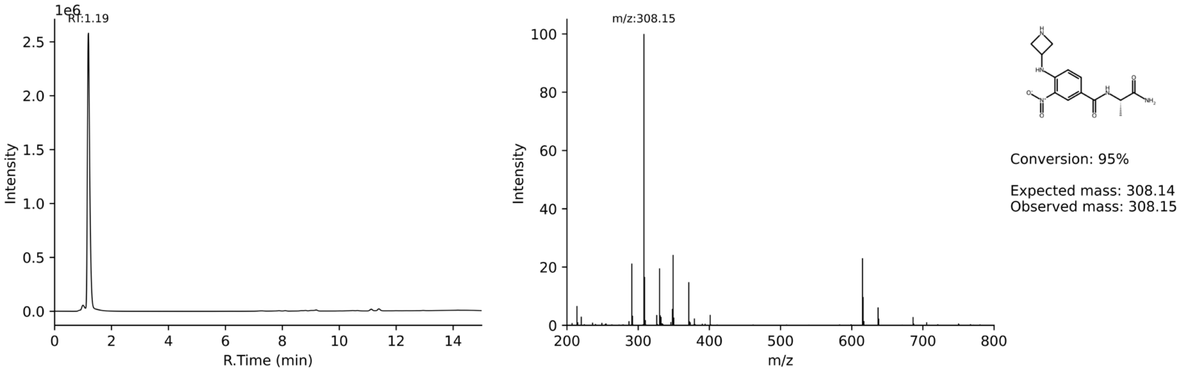

Supplementary Fig 84. LC-MS chromatogram obtained using 3-amino-1-n-boc-azetidine.

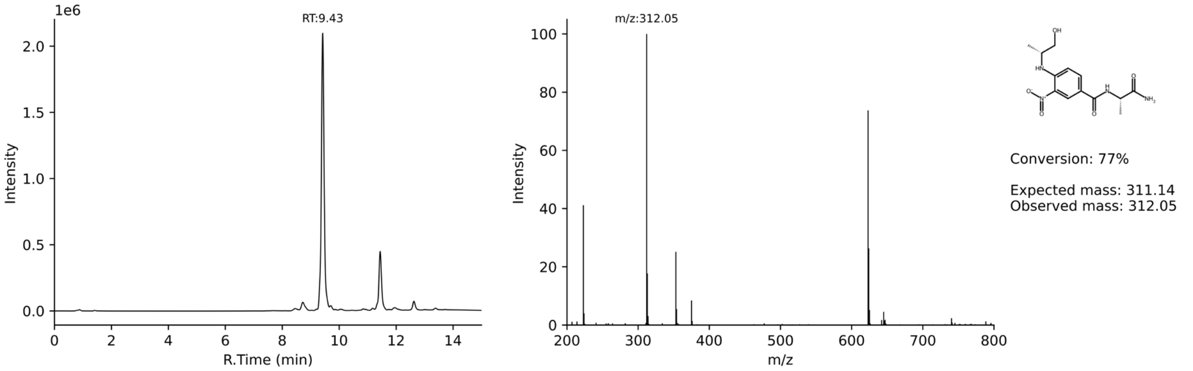

Supplementary Fig 85. LC-MS chromatogram obtained using (R)-(-)-2-amino-1-propanol.

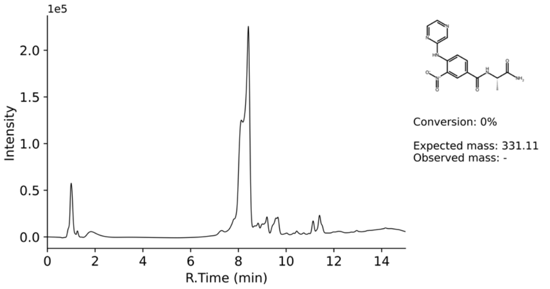

Supplementary Fig 86. LC-MS chromatogram obtained using aminopyrazine.

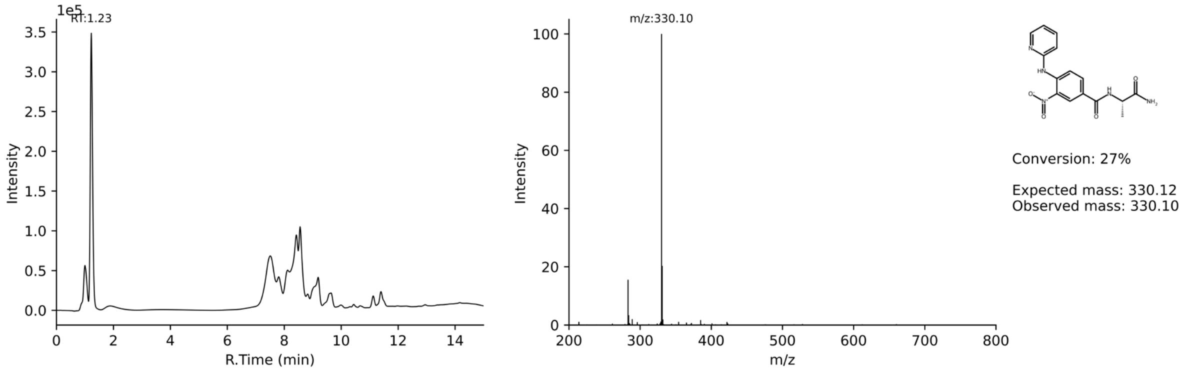

Supplementary Fig 87. LC-MS chromatogram obtained using 2-aminopyridine.

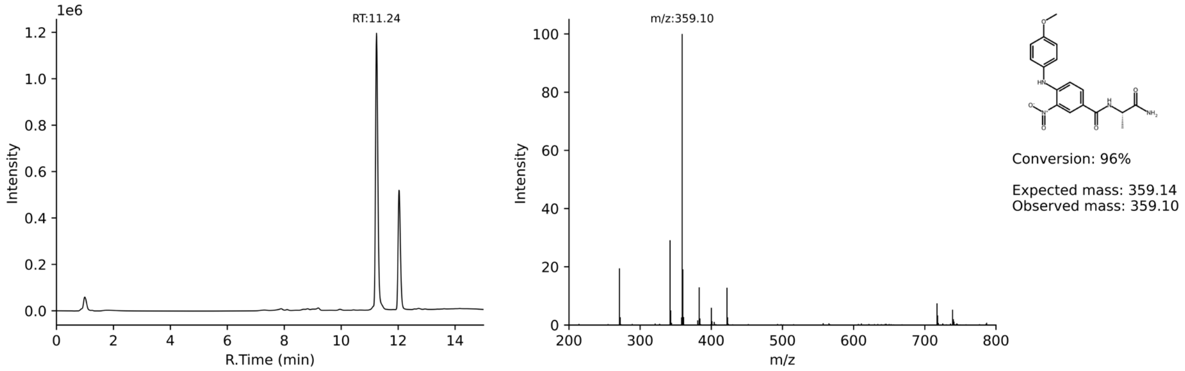

Supplementary Fig 88. LC-MS chromatogram obtained using p-anisidine.

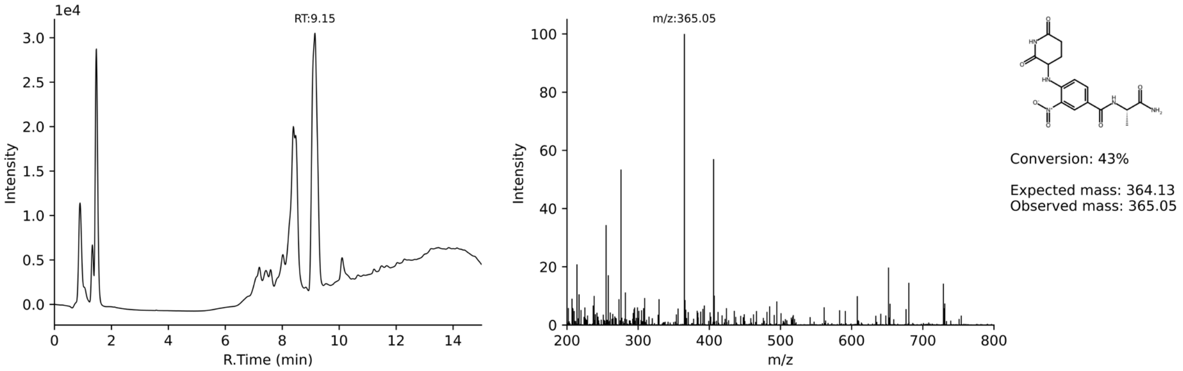

Supplementary Fig 89. LC-MS chromatogram obtained using 3-aminopiperidine-2,6-dione.

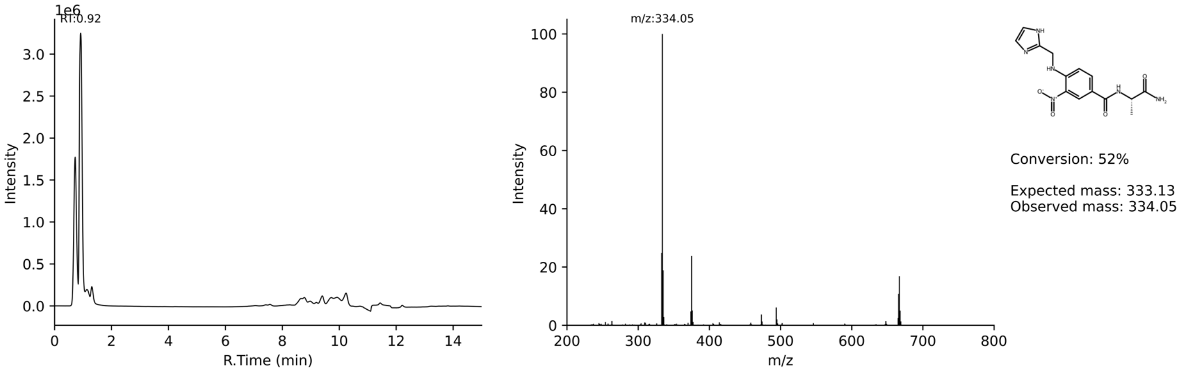

Supplementary Fig 90. LC-MS chromatogram obtained using 2-(aminomethyl)imidazole.

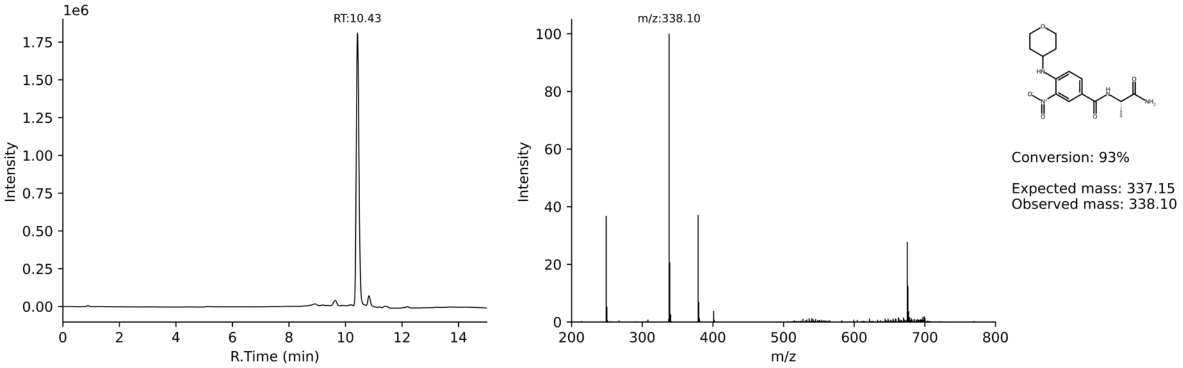

Supplementary Fig 91. LC-MS chromatogram obtained using 4-aminotetrahydropyran.

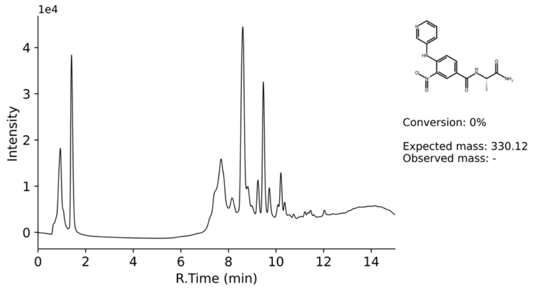

Supplementary Fig 92. LC-MS chromatogram obtained using 3-aminopyridine.

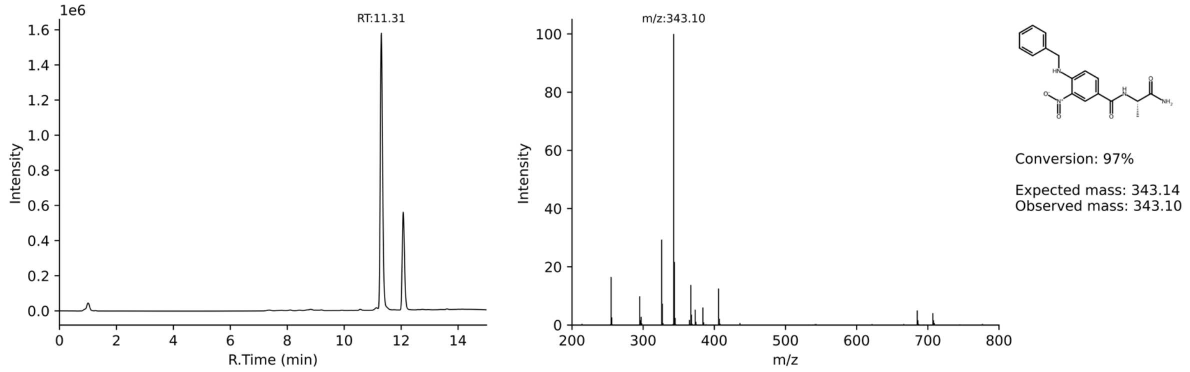

Supplementary Fig 93. LC-MS chromatogram obtained using benzylamine.

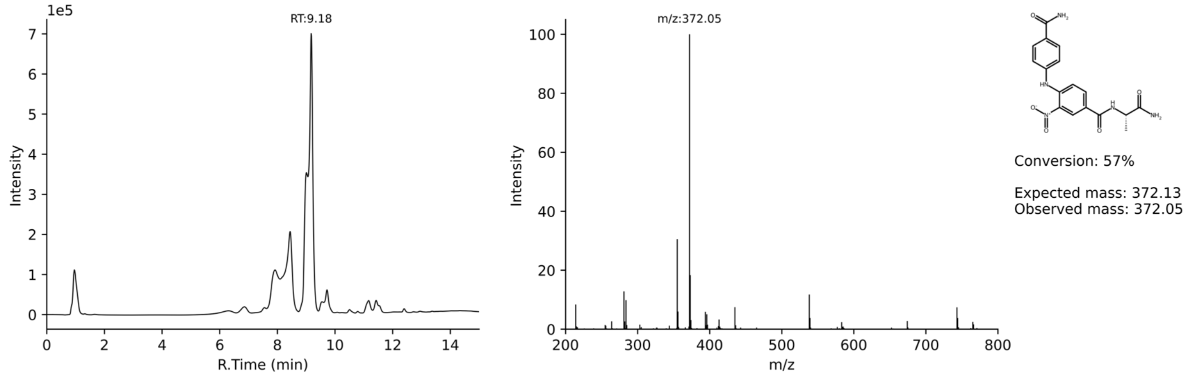

Supplementary Fig 94. LC-MS chromatogram obtained using p-aminobenzamide.

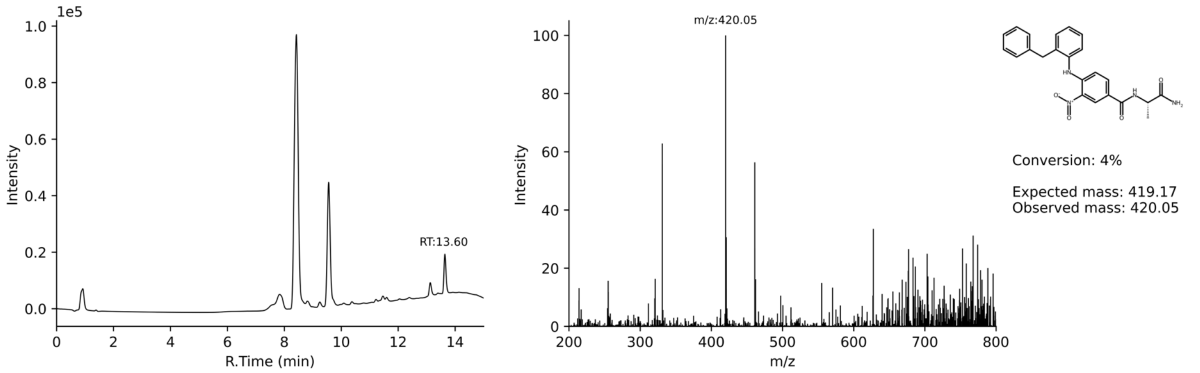

Supplementary Fig 95. LC-MS chromatogram obtained using 2-benzylaniline.

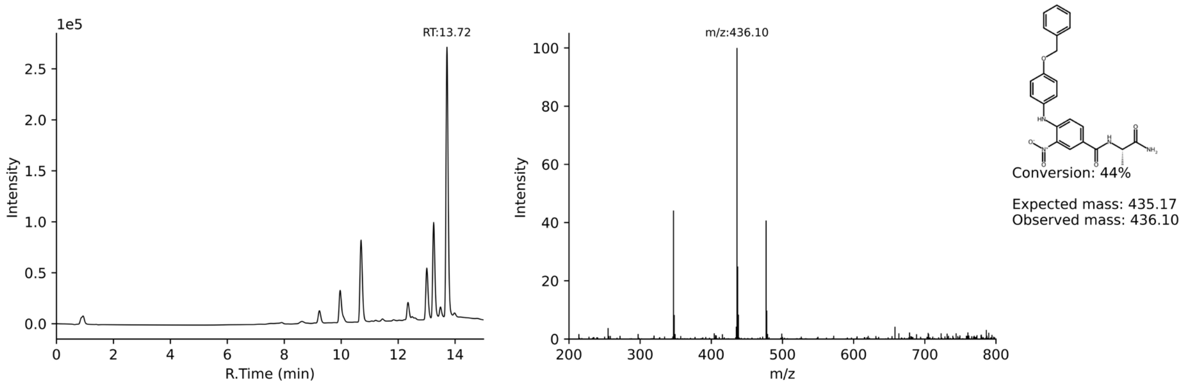

Supplementary Fig 96. LC-MS chromatogram obtained using 4-(benzyloxy)aniline.

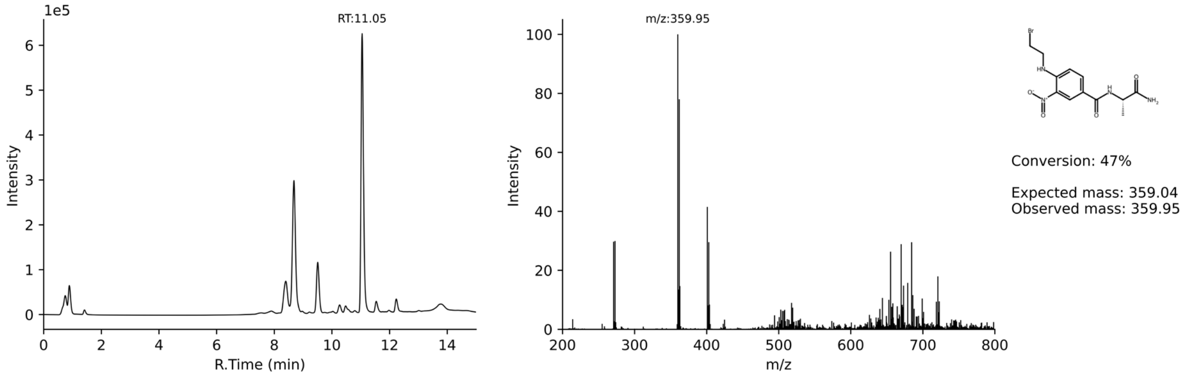

Supplementary Fig 97. LC-MS chromatogram obtained using 2-bromoethylammonium bromide.

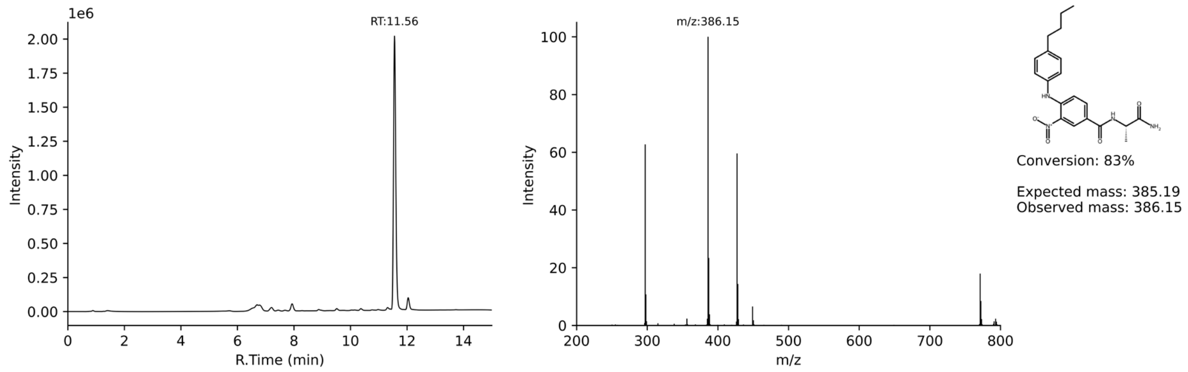

Supplementary Fig 98. LC-MS chromatogram obtained using 4-butylaniline.

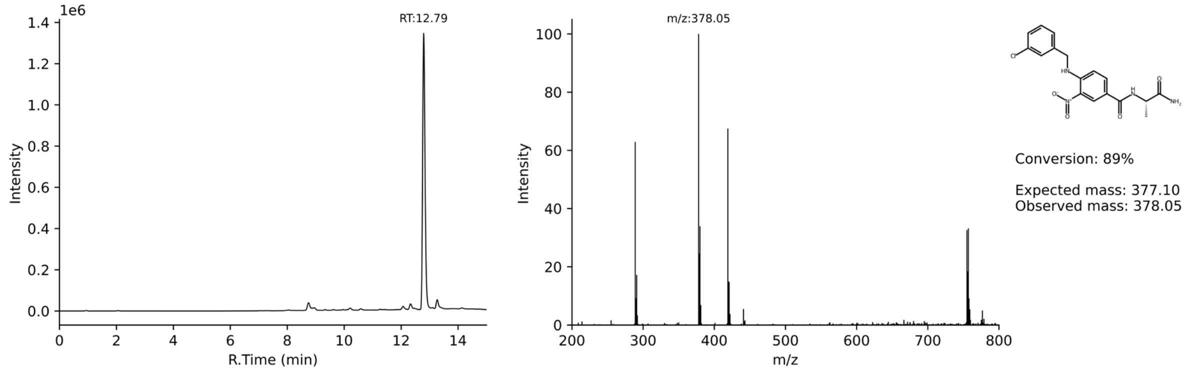

Supplementary Fig 99. LC-MS chromatogram obtained using 3-chlorobenzylamine.

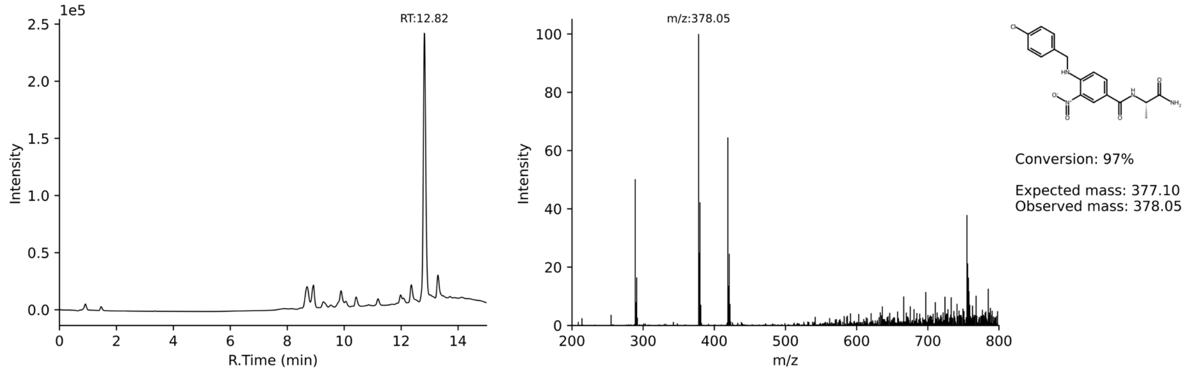

Supplementary Fig 100. LC-MS chromatogram obtained using 4-chlorobenzylamine.

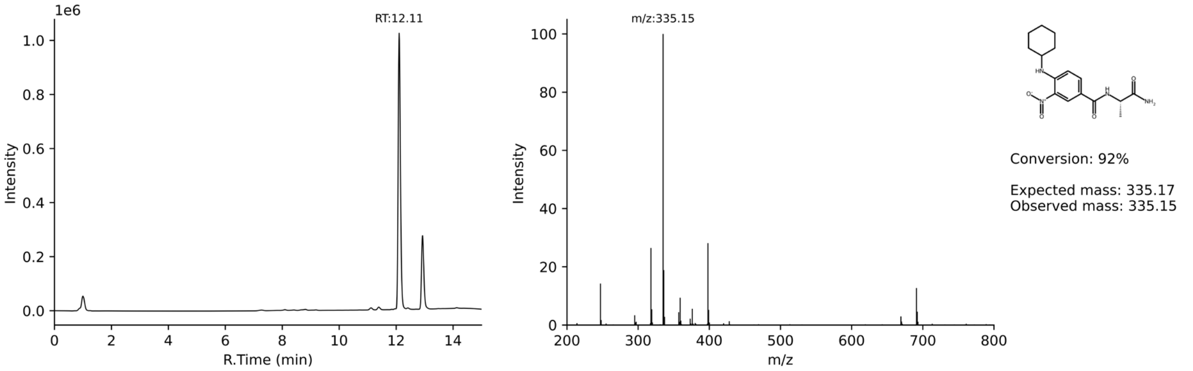

Supplementary Fig 101. LC-MS chromatogram obtained using cyclohexylamine.

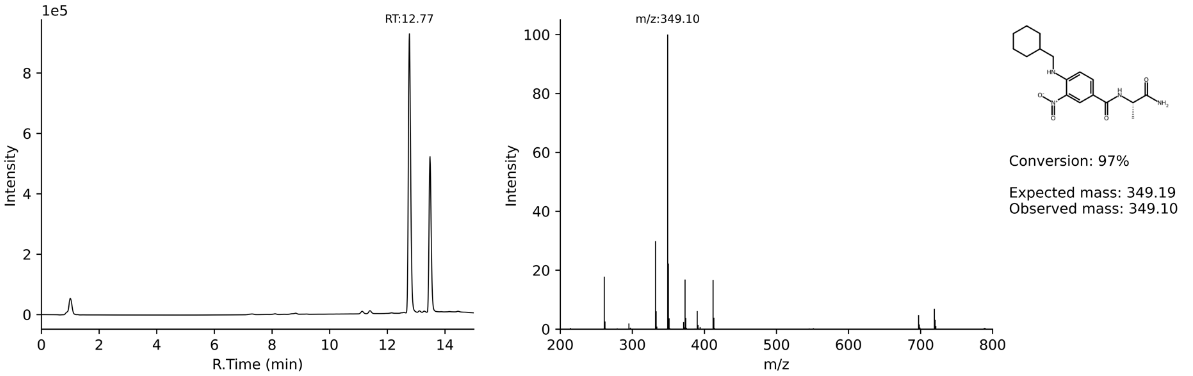

Supplementary Fig 102. LC-MS chromatogram obtained using cyclohexane methylamine.

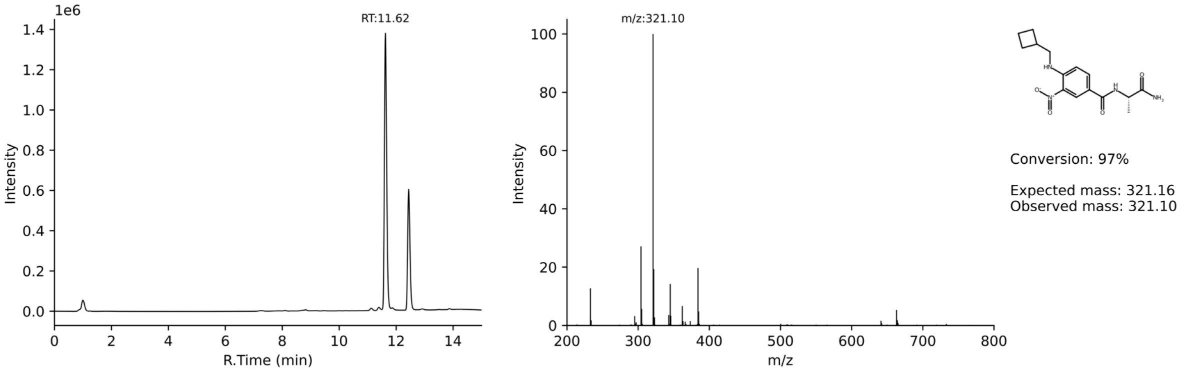

Supplementary Fig 103. LC-MS chromatogram obtained using cyclobutylmethylamine.

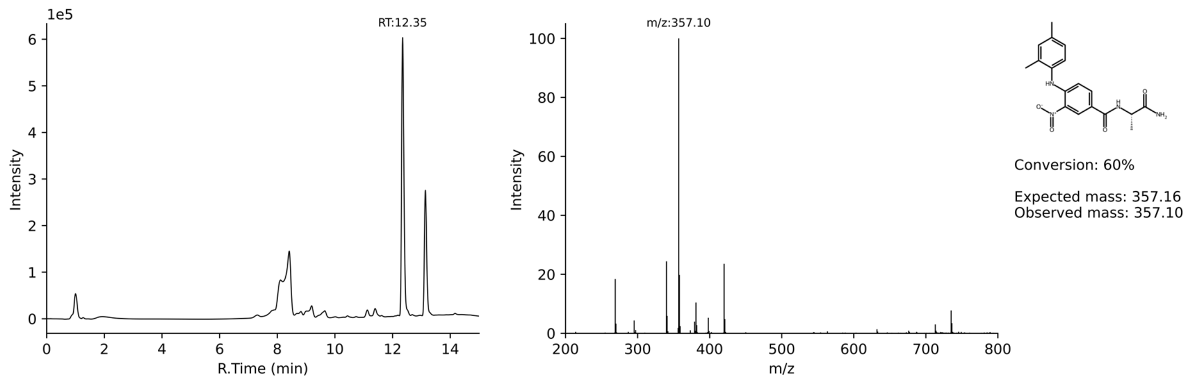

Supplementary Fig 104. LC-MS chromatogram obtained using2,4-dimethylaniline.

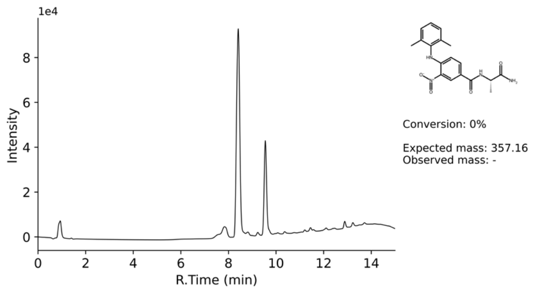

Supplementary Fig 105. LC-MS chromatogram obtained using 2,6-dimethylaniline.

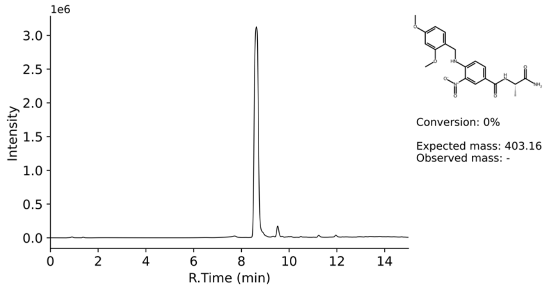

Supplementary Fig 106. LC-MS chromatogram obtained using 2,4-dimethoxybenzylamine.

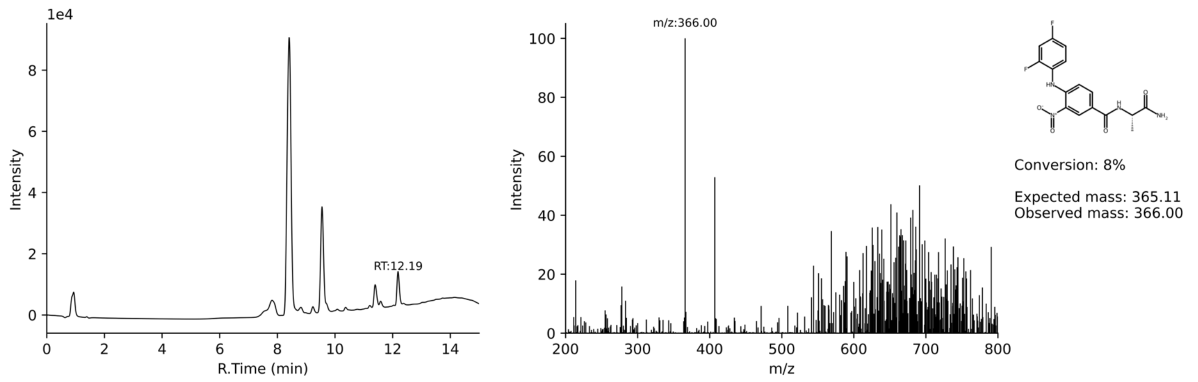

Supplementary Fig 107. LC-MS chromatogram obtained using 2,4-difluoroaniline.

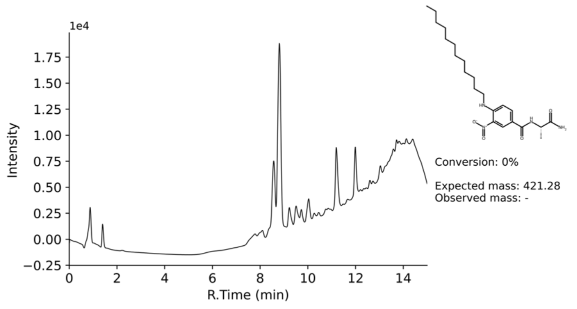

Supplementary Fig 108. LC-MS chromatogram obtained using 2,2-diphenylethylamine.

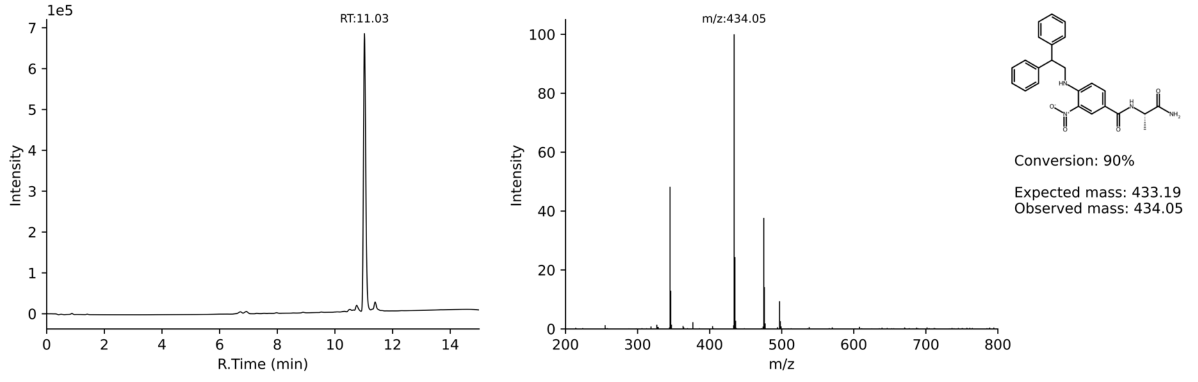

Supplementary Fig 109. LC-MS chromatogram obtained using 3,5-dimethylbenzylamine.

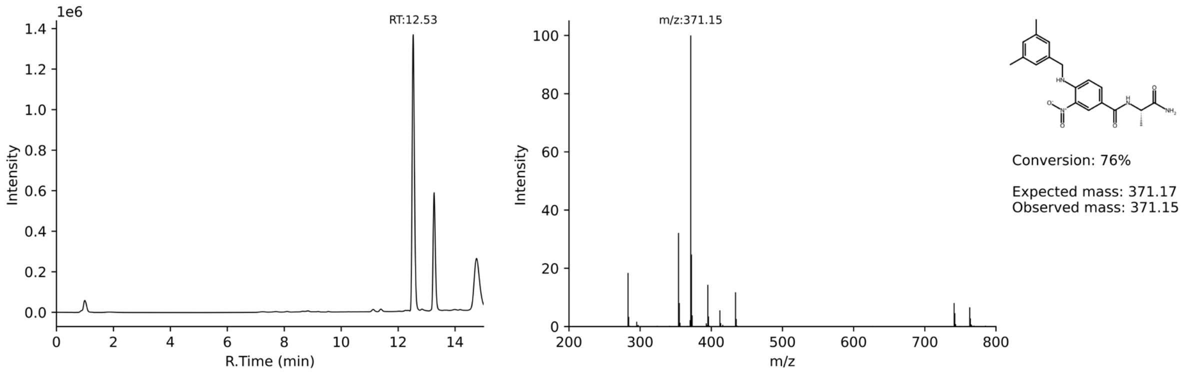

Supplementary Fig 110. LC-MS chromatogram obtained using 3,5-dimethylbenzylamine.

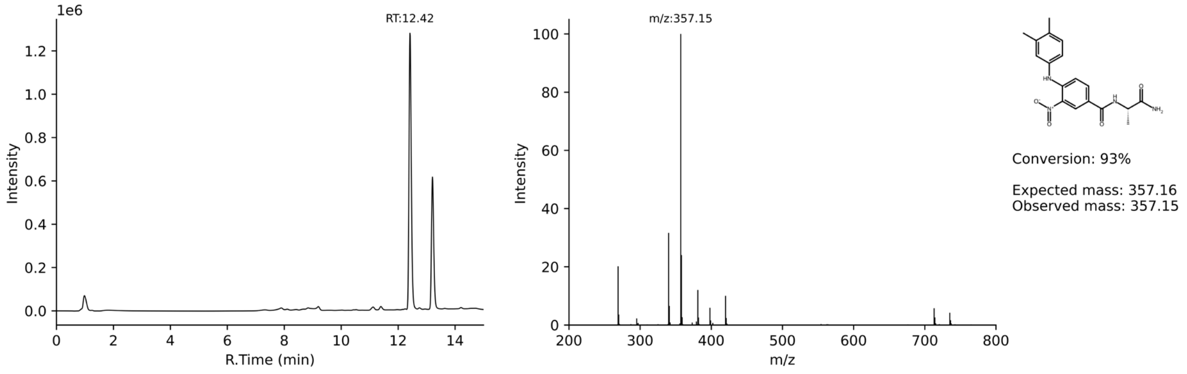

Supplementary Fig 111. LC-MS chromatogram obtained using 3,4-dimethylaniline.

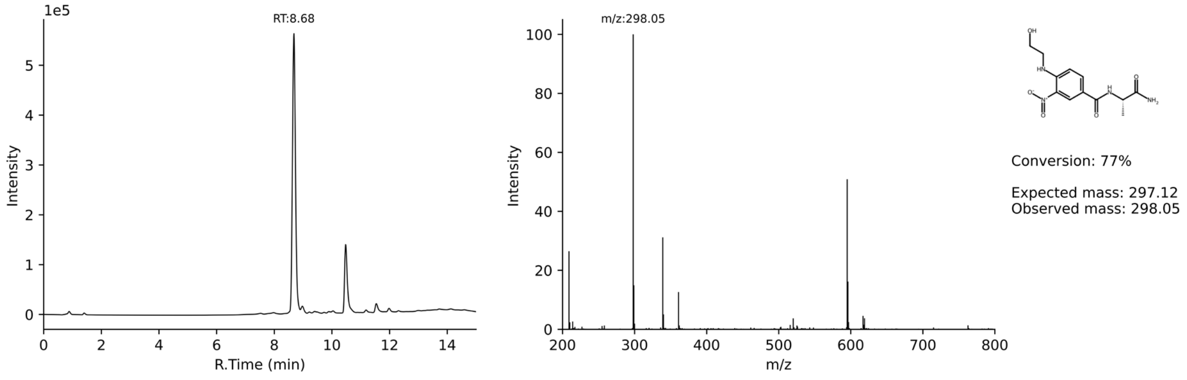

Supplementary Fig 112. LC-MS chromatogram obtained using 2-aminoethanol.

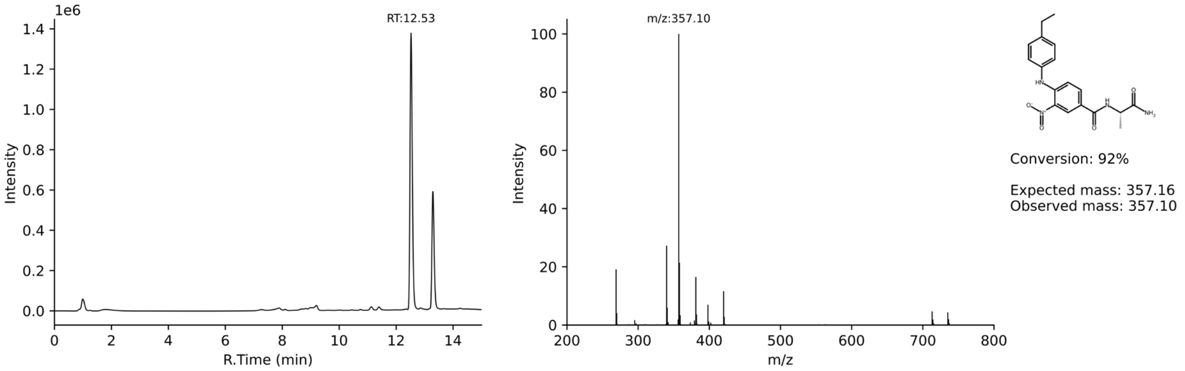

Supplementary Fig 113. LC-MS chromatogram obtained using 4-ethylaniline.

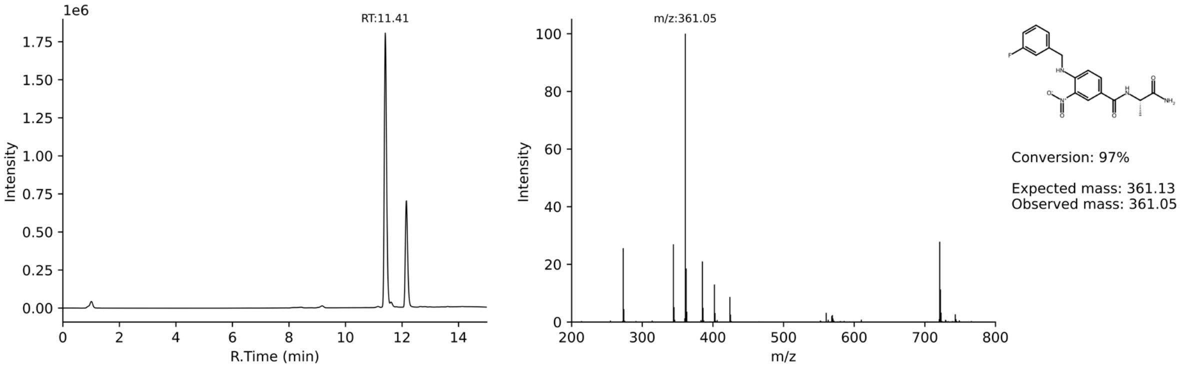

Supplementary Fig 114. LC-MS chromatogram obtained using 3-fluorobenzylamine.

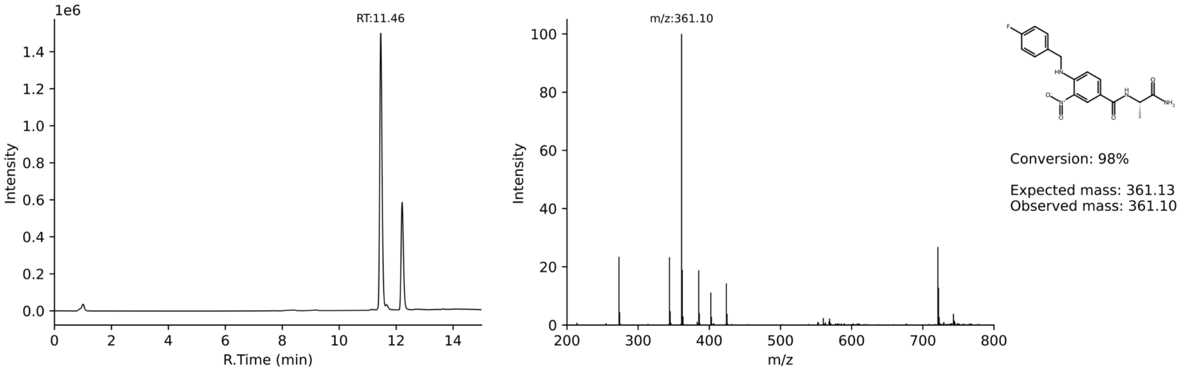

Supplementary Fig 115. LC-MS chromatogram obtained using 4-fluorobenzylamine.

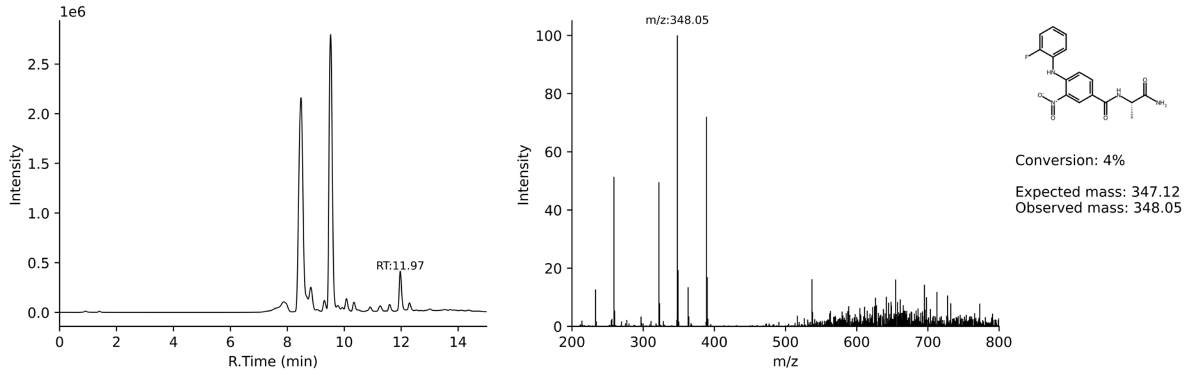

Supplementary Fig 116. LC-MS chromatogram obtained using 2-fluorobenzenamine.

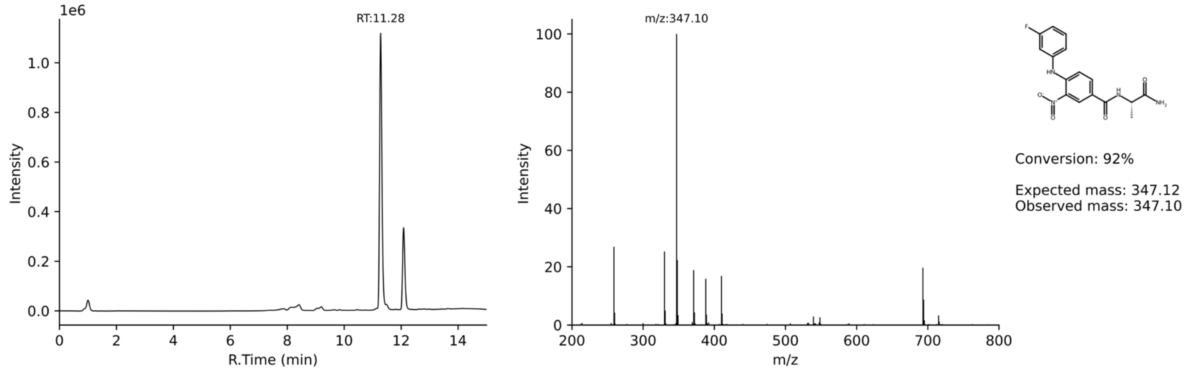

Supplementary Fig 117. LC-MS chromatogram obtained using 3-fluoroaniline.

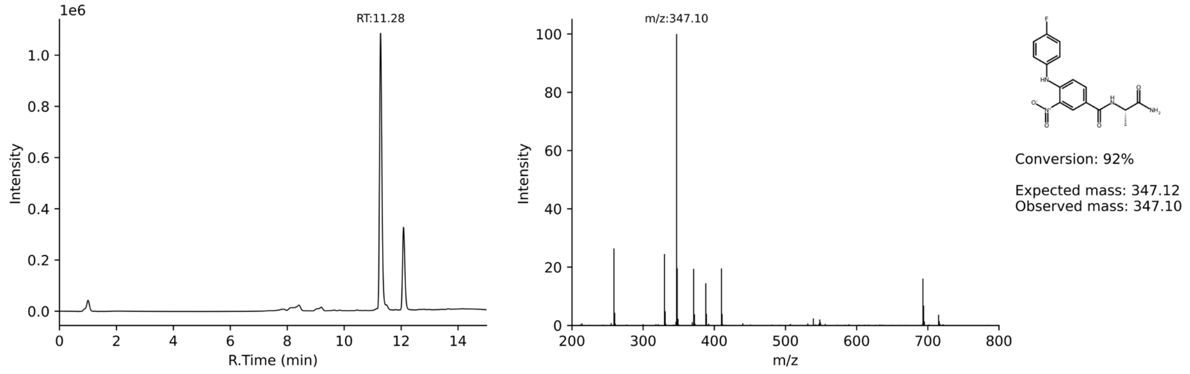

Supplementary Fig 118. LC-MS chromatogram obtained using 4-fluorobenzenamine.

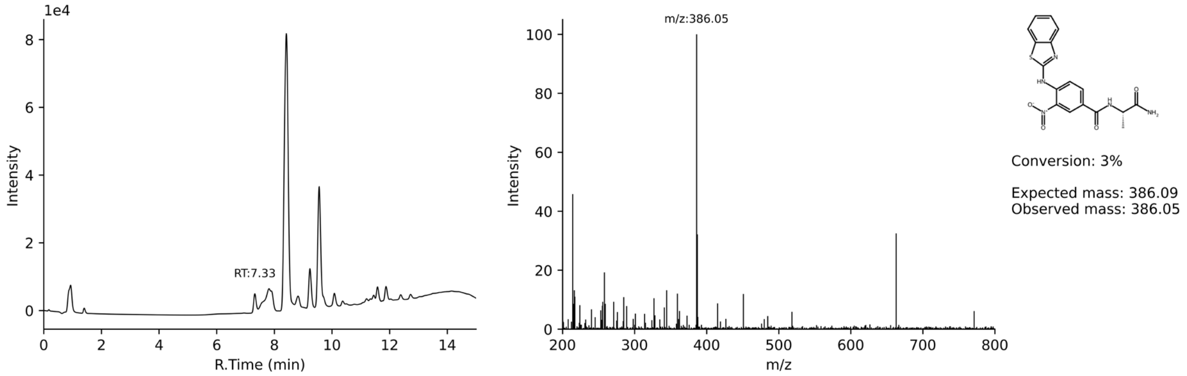

Supplementary Fig 119. LC-MS chromatogram obtained using 2-Aminobenzothiazole.

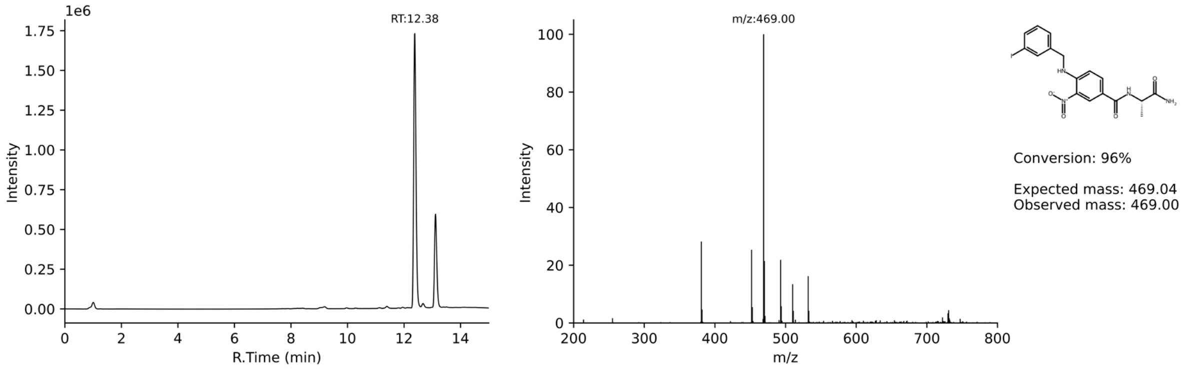

Supplementary Fig 120. LC-MS chromatogram obtained using 3-iodobenzylamine.

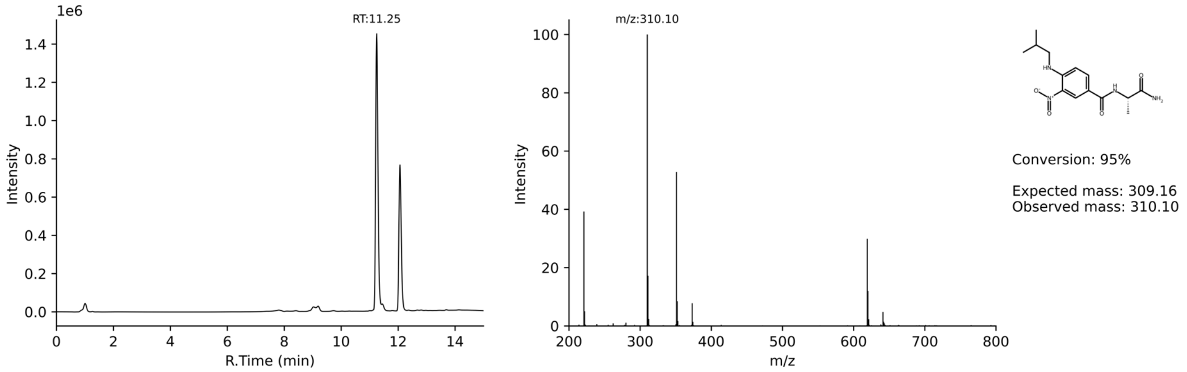

Supplementary Fig 121. LC-MS chromatogram obtained using isobutylamine.

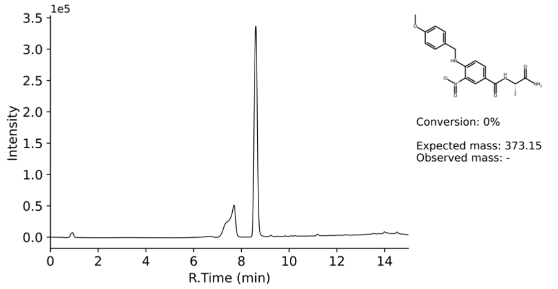

Supplementary Fig 122. LC-MS chromatogram obtained using 4-methoxybenzylamine.

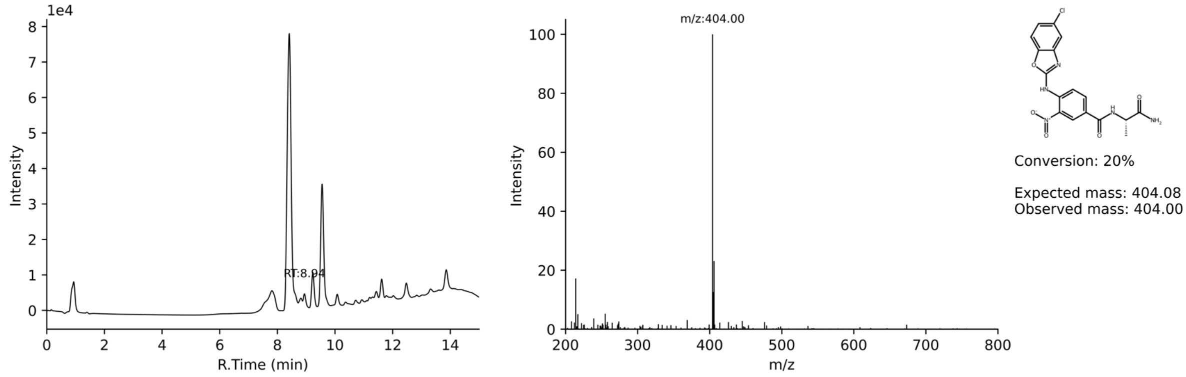

Supplementary Fig 123. LC-MS chromatogram obtained using 2-amino-5-chlorobenzoxazole.

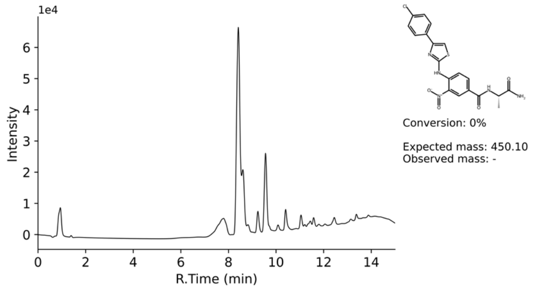

Supplementary Fig 124. LC-MS chromatogram obtained using 2-amino-4-(4-chlorophenyl)thiazole.

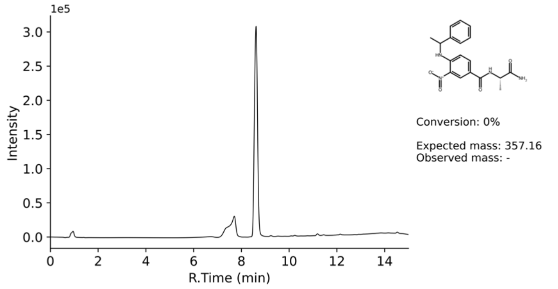

Supplementary Fig 125. LC-MS chromatogram obtained using alpha-methylbenzylamine.

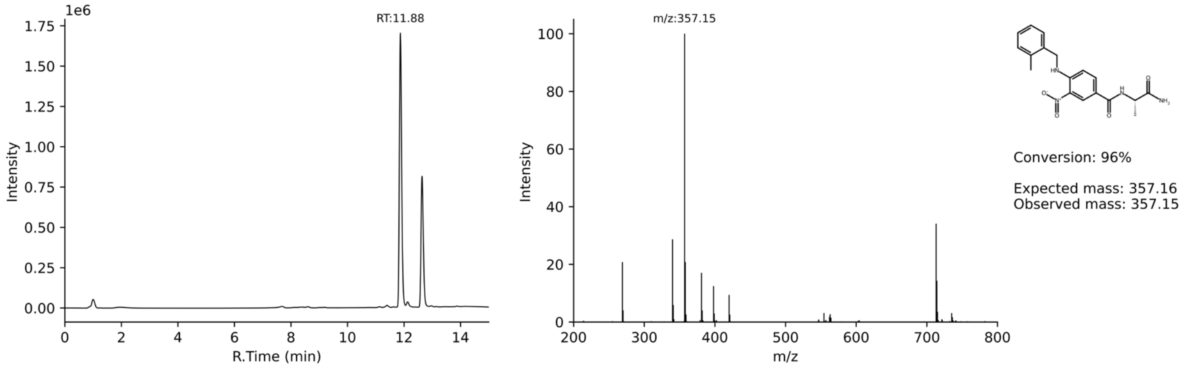

Supplementary Fig 126. LC-MS chromatogram obtained using 2-methylbenzylamine.

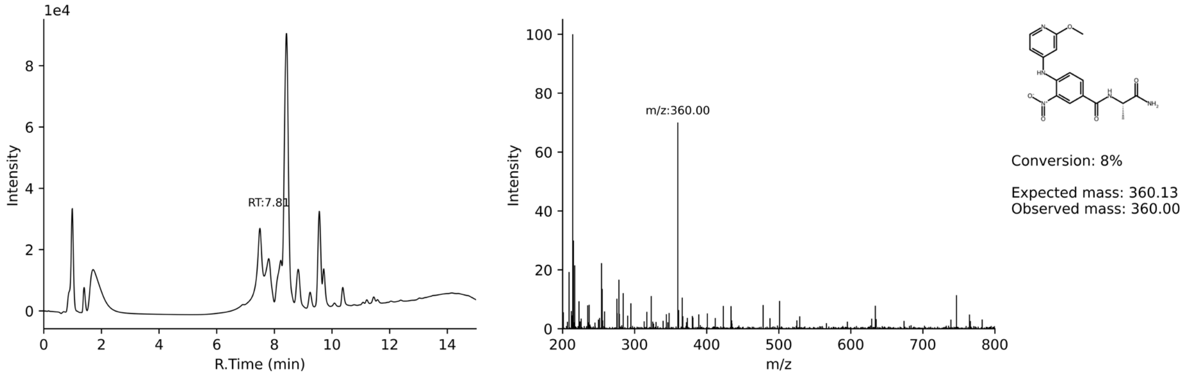

Supplementary Fig 127. LC-MS chromatogram obtained using 2-methoxypyridin-4-amine.

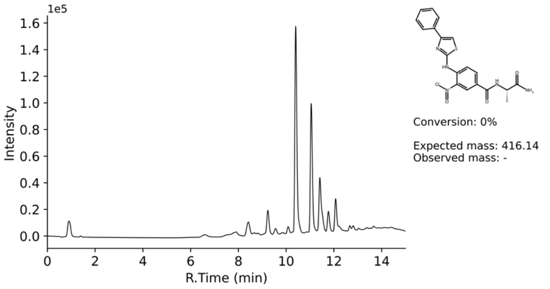

Supplementary Fig 128. LC-MS chromatogram obtained using 2-amino-4-phenylthiazole.

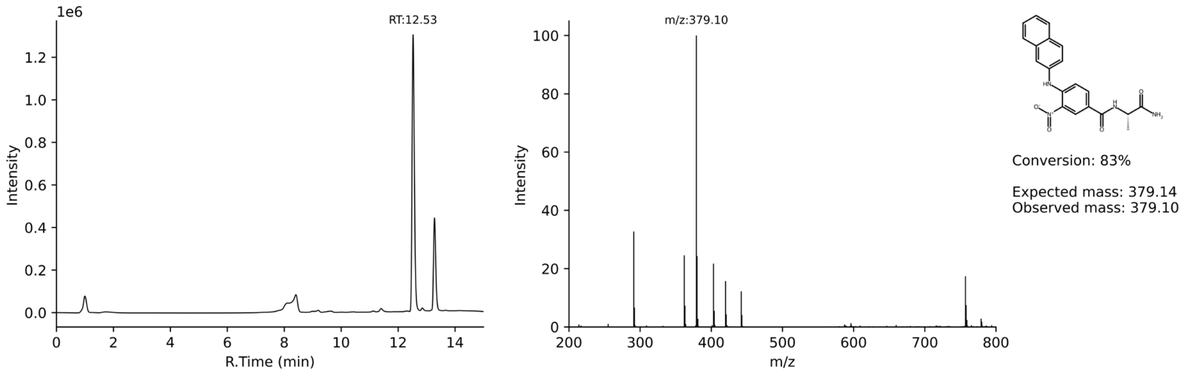

Supplementary Fig 129. LC-MS chromatogram obtained using 2-naphthylamine.

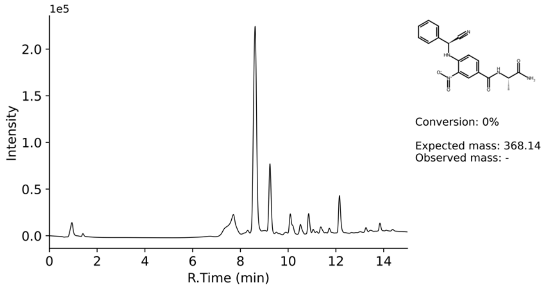

Supplementary Fig 130. LC-MS chromatogram obtained using 2-phenylglycinonitrile.

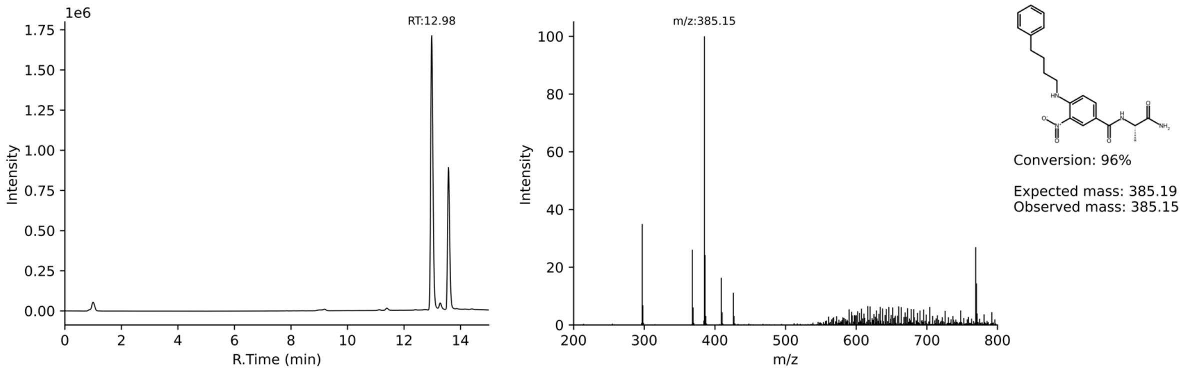

Supplementary Fig 131. LC-MS chromatogram obtained using 4-phenylbutylamine.

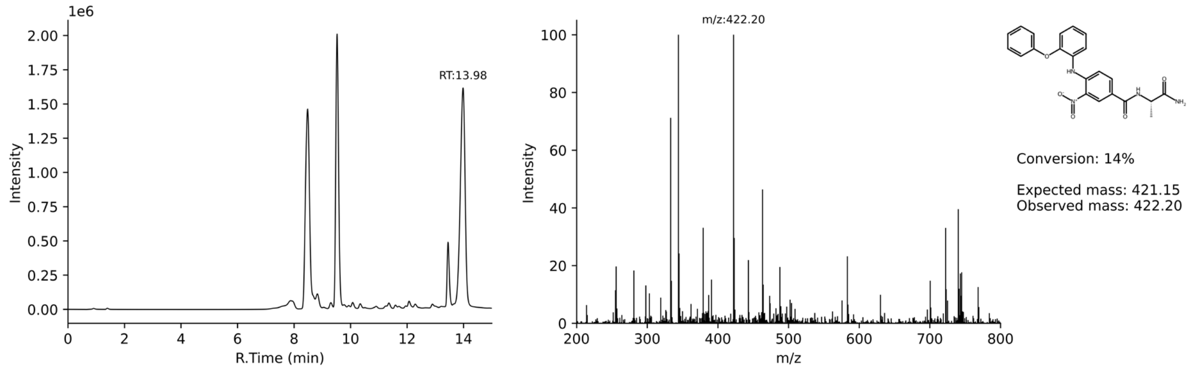

Supplementary Fig 132. LC-MS chromatogram obtained using 2-phenoxyaniline.

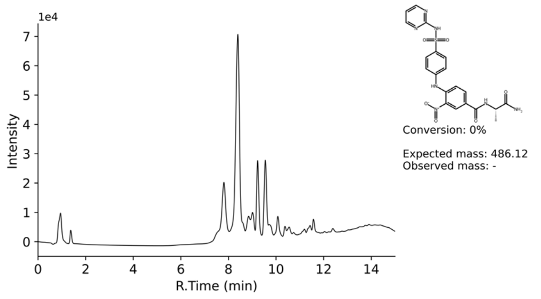

Supplementary Fig 133. LC-MS chromatogram obtained using sulfadiazine.

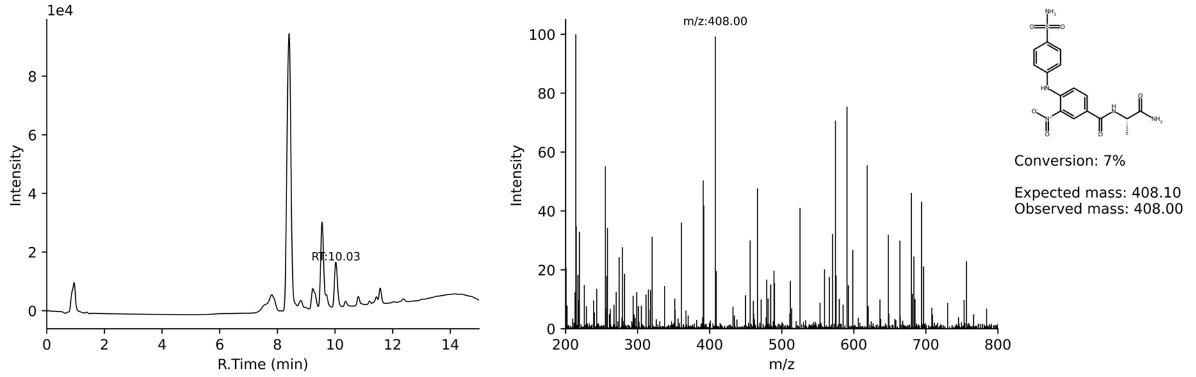

Supplementary Fig 134. LC-MS chromatogram obtained using sulfanilamide.

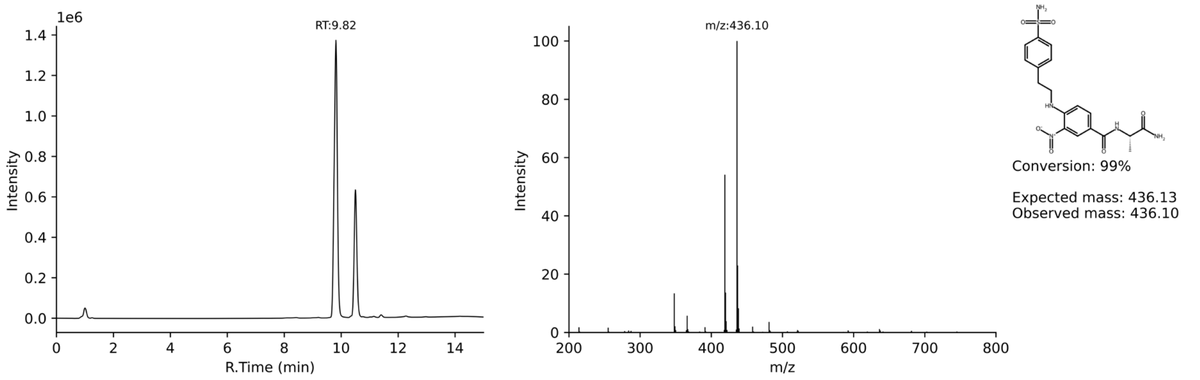

Supplementary Fig 135. LC-MS chromatogram obtained using 4-(2-aminoethyl)benzenesulfonamide.

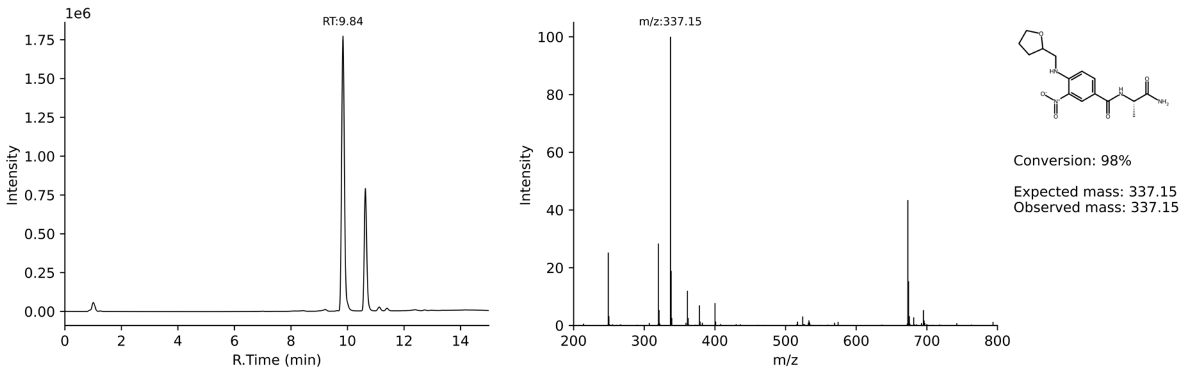

Supplementary Fig 136. LC-MS chromatogram obtained using tetrahydrofurfurylamine.

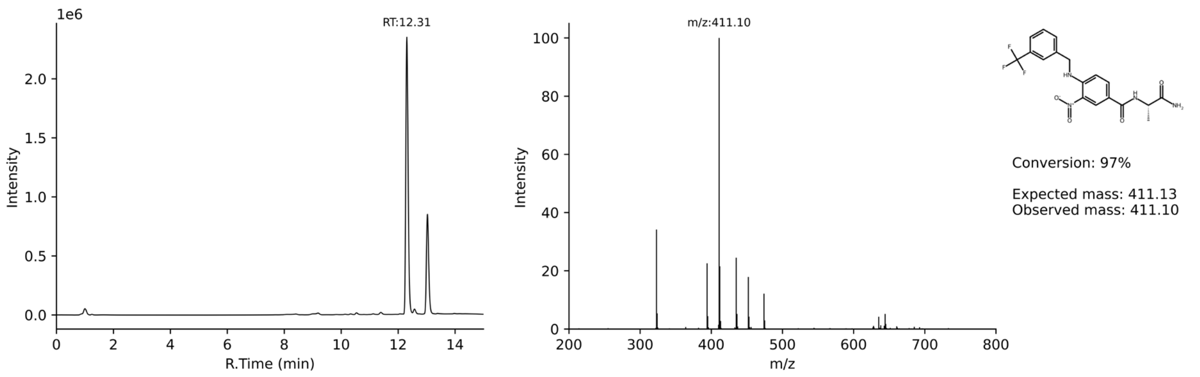

Supplementary Fig 137. LC-MS chromatogram obtained using 3-(trifluoromethyl)benzylamine.

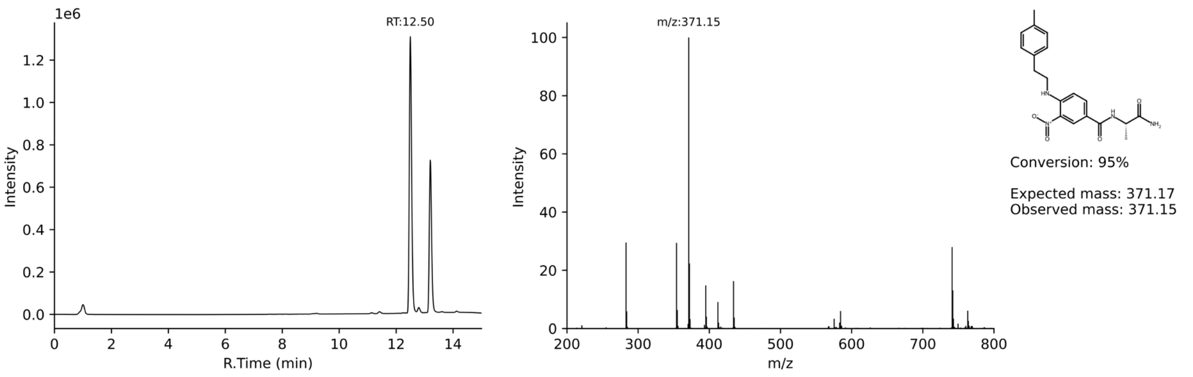

Supplementary Fig 138. LC-MS chromatogram obtained using 2-(4-tolyl) ethylamine.

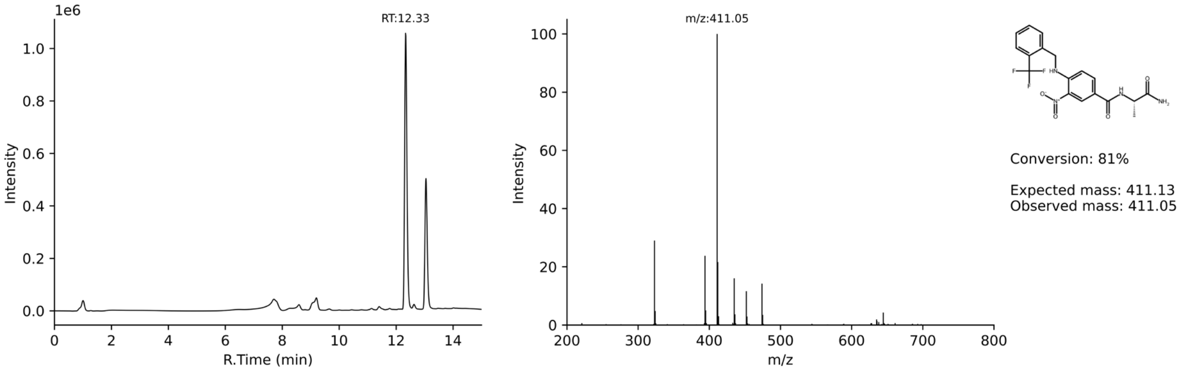

Supplementary Fig 139. LC-MS chromatogram obtained using 2-(trifluoromethyl)benzylamine.

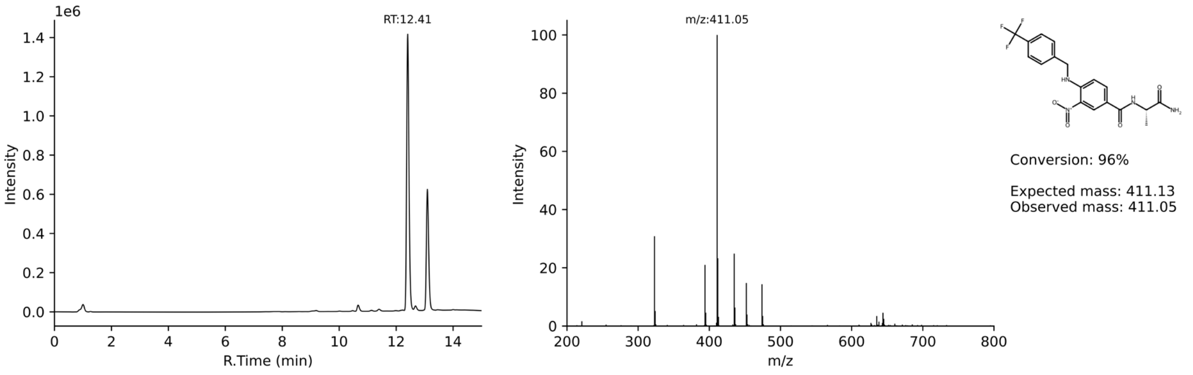

Supplementary Fig 140. LC-MS chromatogram obtained using 4-(trifluoromethyl)benzylamine.

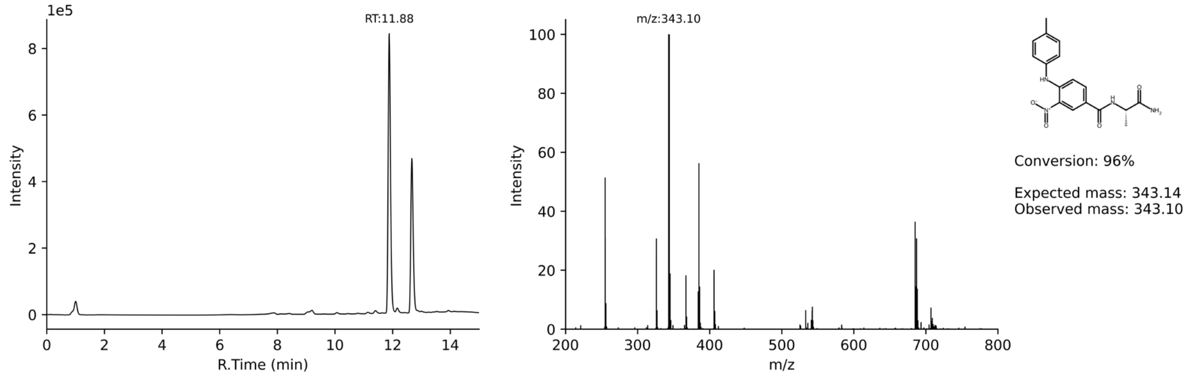

Supplementary Fig 141. LC-MS chromatogram obtained using p-toluidine.

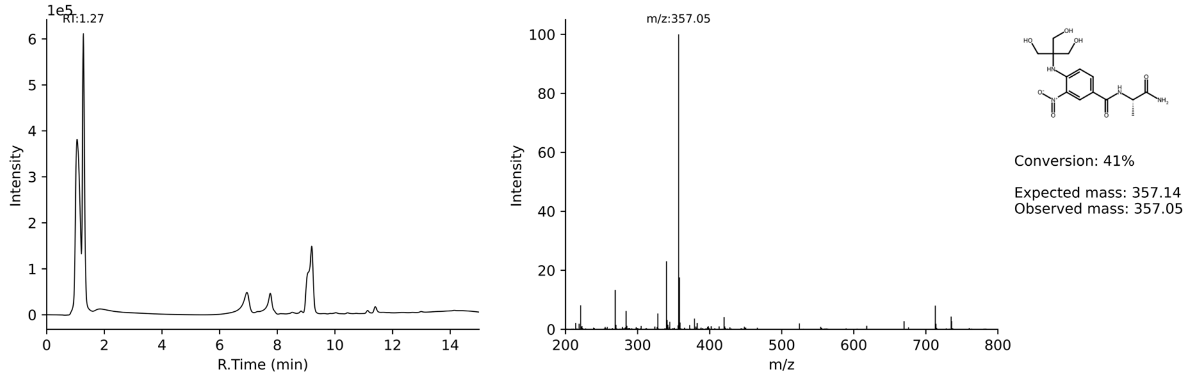

Supplementary Fig 142. LC-MS chromatogram obtained using tris(hydroxymethyl)aminomethane.

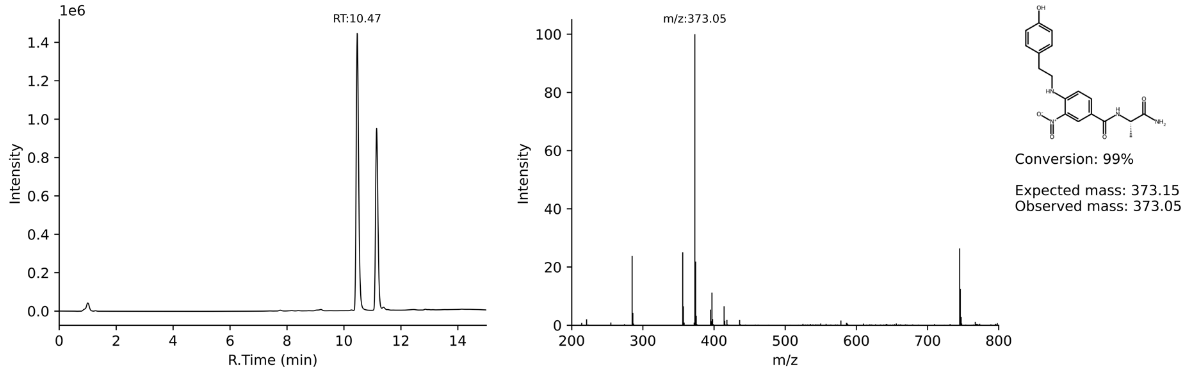

Supplementary Fig 143. LC-MS chromatogram obtained using tyramine.

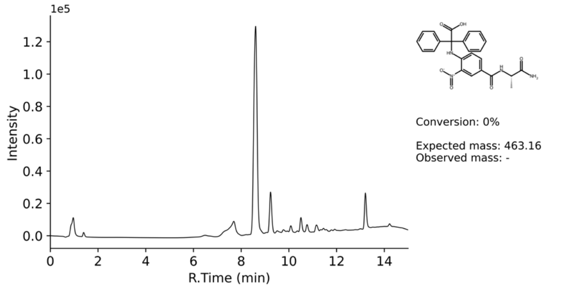

Supplementary Fig 144. LC-MS chromatogram obtained using 2,2-diphenylglycine.

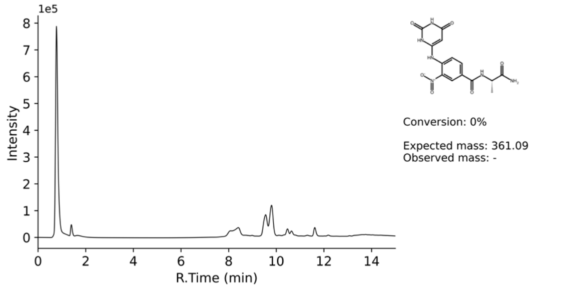

Supplementary Fig 145. LC-MS chromatogram obtained using 6-aminouracil.

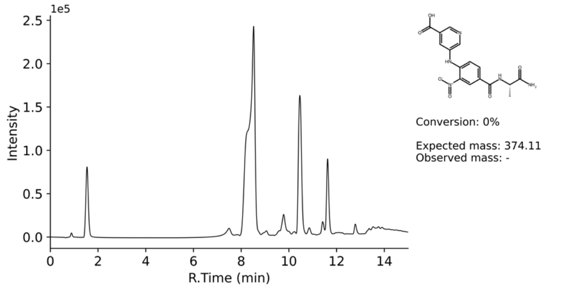

Supplementary Fig 146. LC-MS chromatogram obtained using 5-aminonicotinic acid.

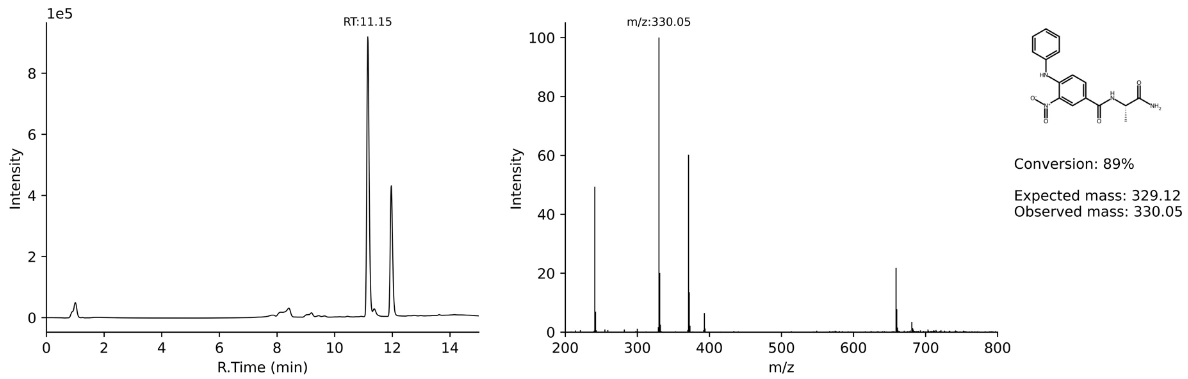

Supplementary Fig 147. LC-MS chromatogram obtained using aniline.

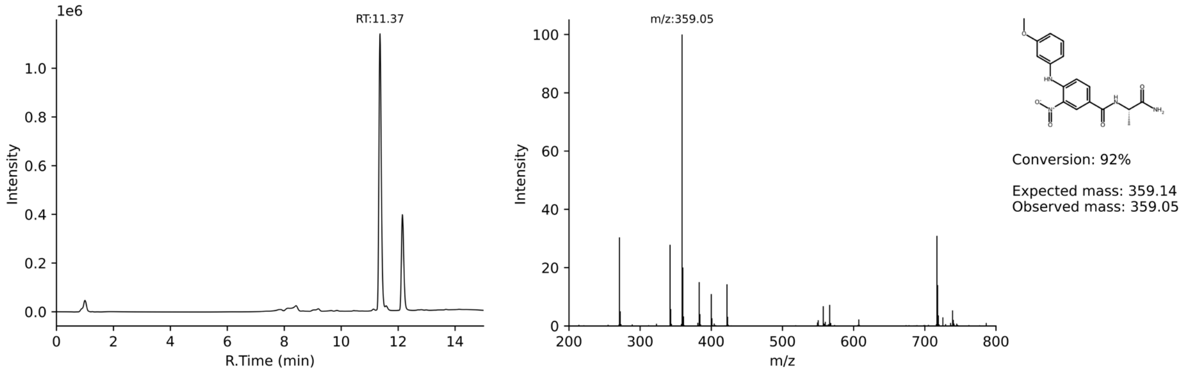

Supplementary Fig 148. LC-MS chromatogram obtained using 3-anisidine.

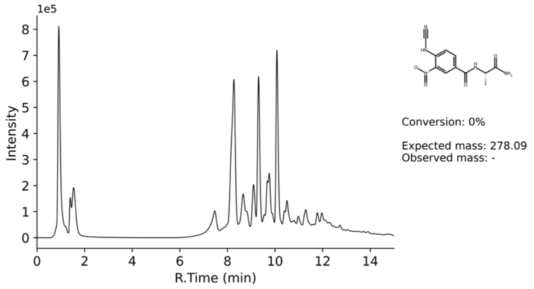

Supplementary Fig 149. LC-MS chromatogram obtained using cyanamide.

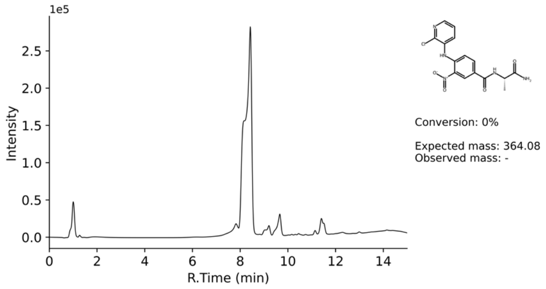

Supplementary Fig 150. LC-MS chromatogram obtained using 3-amino-2-chloropyridine.

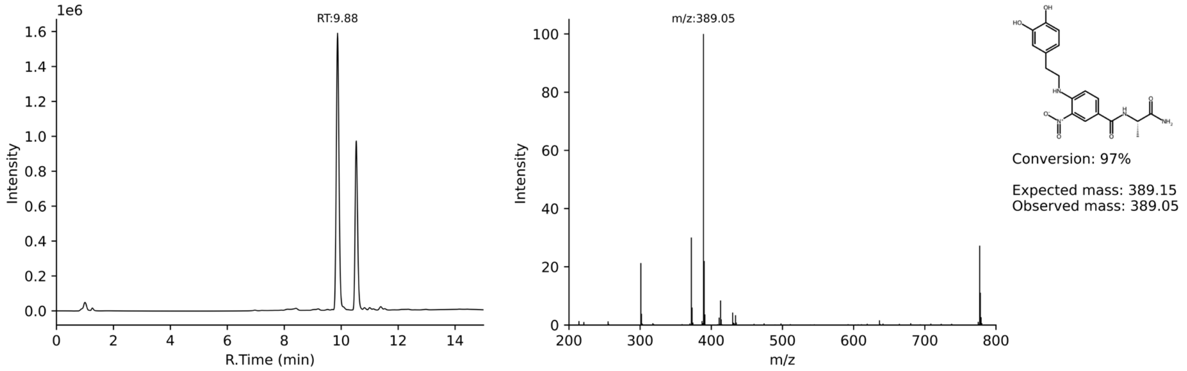

Supplementary Fig 151. LC-MS chromatogram obtained using 3-hydroxy-tyramine.

12.2 LC-MS data from the scope of aldehydes

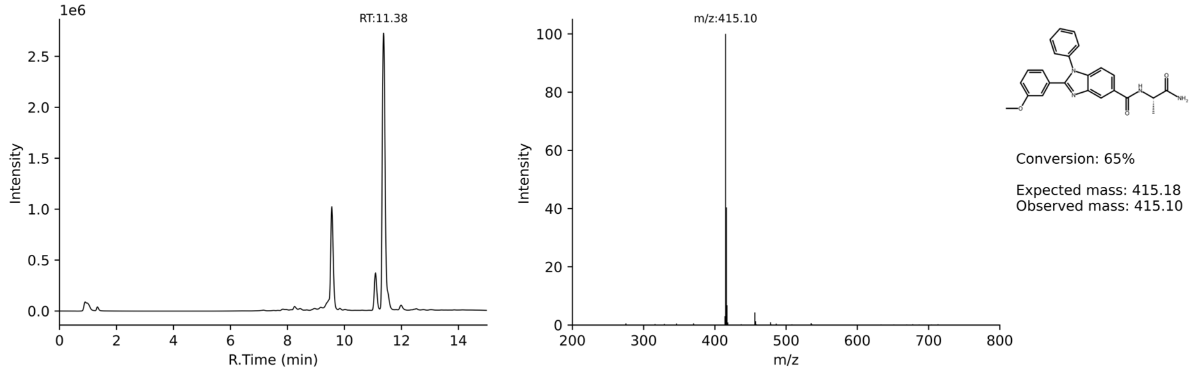

Supplementary Fig 152. LC-MS chromatogram obtained using 3-methoxybenzaldehyde.

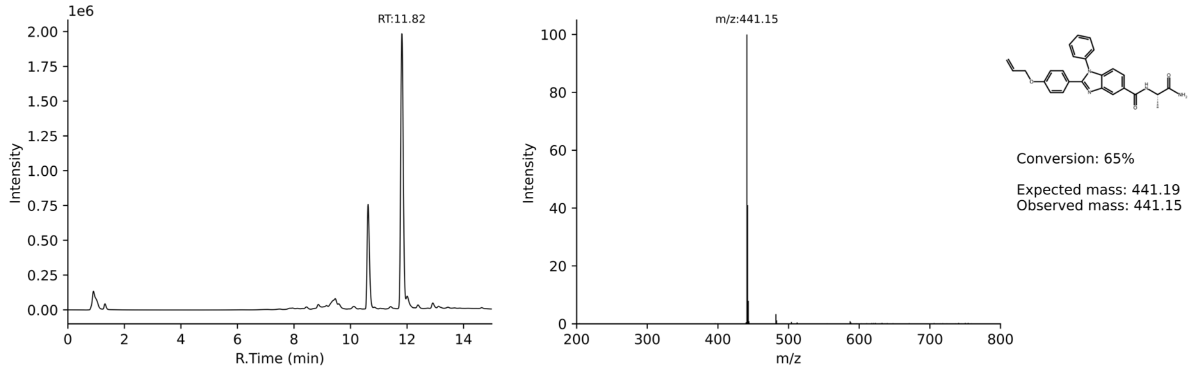

Supplementary Fig 153. LC-MS chromatogram obtained using 4-allyloxybenzaldehyde.

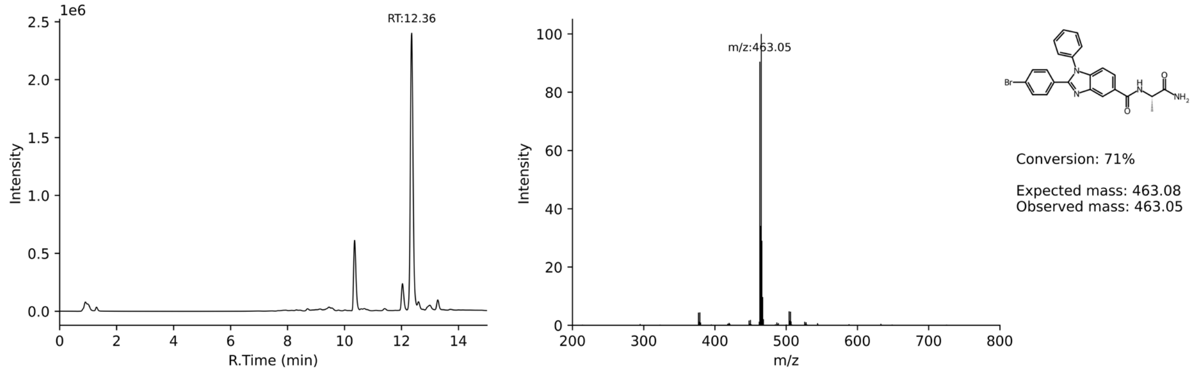

Supplementary Fig 154. LC-MS chromatogram obtained using 4-bromobenzaldehyde.

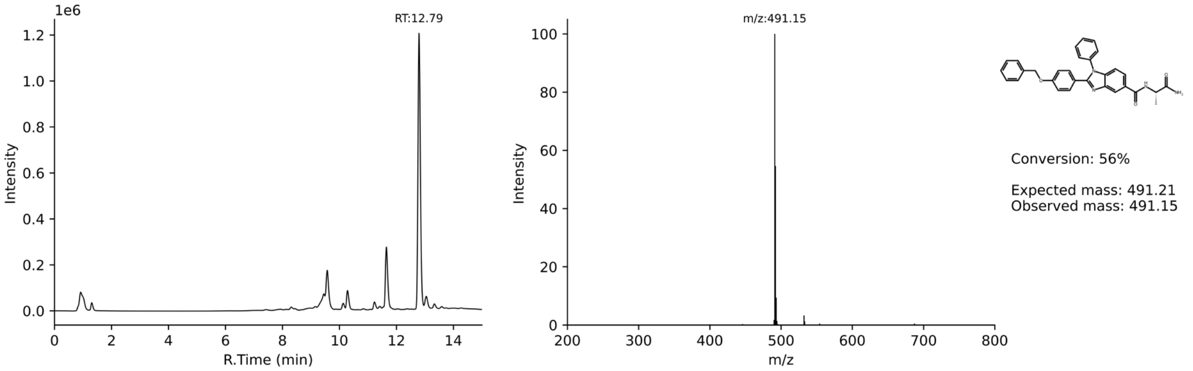

Supplementary Fig 155. LC-MS chromatogram obtained using 4-benzyloxybenzaldehyde.

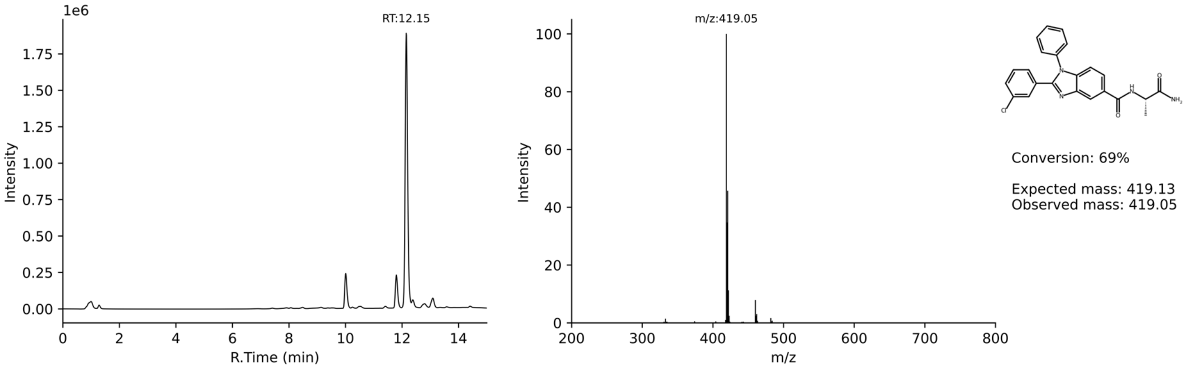

Supplementary Fig 156. LC-MS chromatogram obtained using 3-chlorobenzaldehyde.

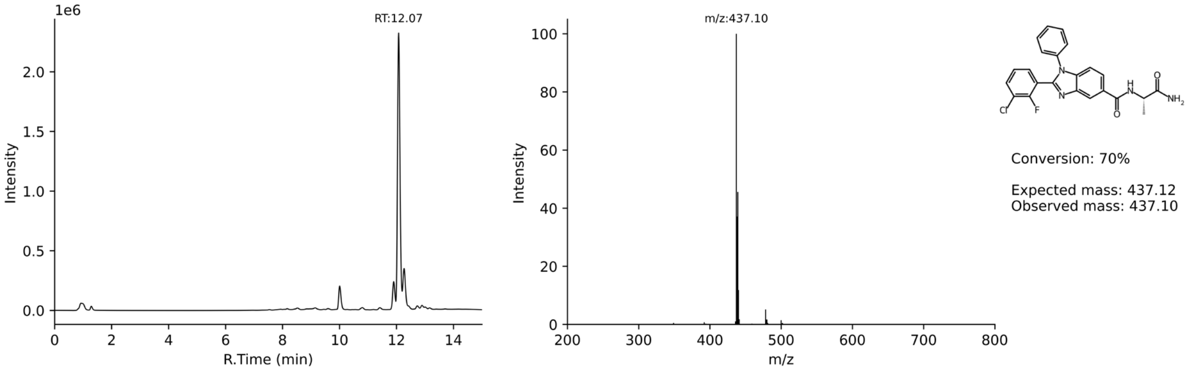

Supplementary Fig 157. LC-MS chromatogram obtained using 3-chloro-2-fluorobenzaldehyde.

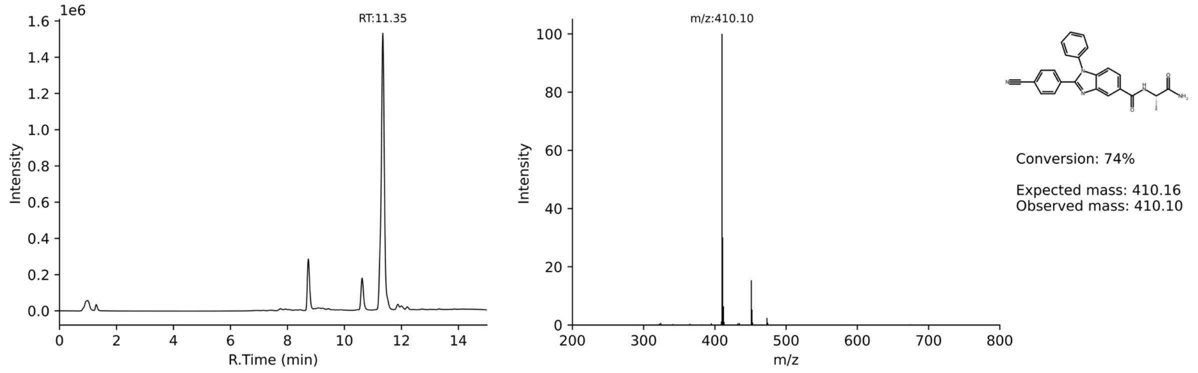

Supplementary Fig 158. LC-MS chromatogram obtained using 4-cyanobenzaldehyde.

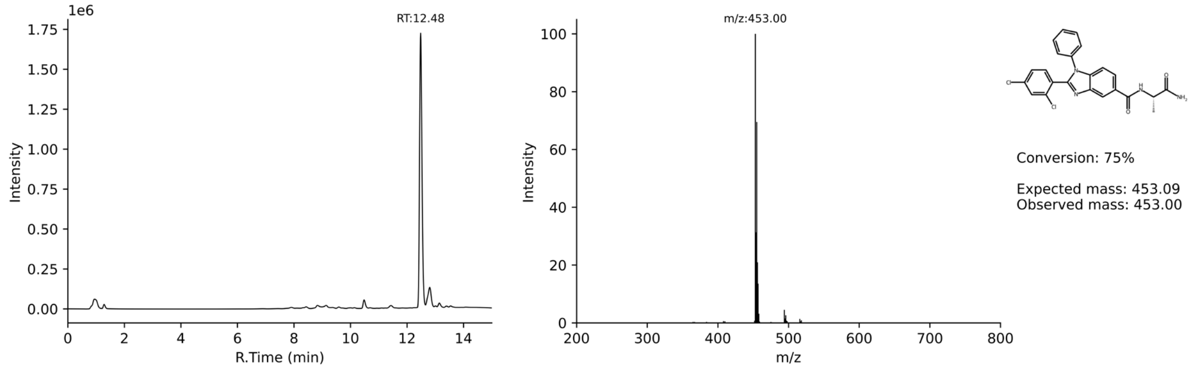

Supplementary Fig 159. LC-MS chromatogram obtained using 2,4-dichlorobenzaldehyde.

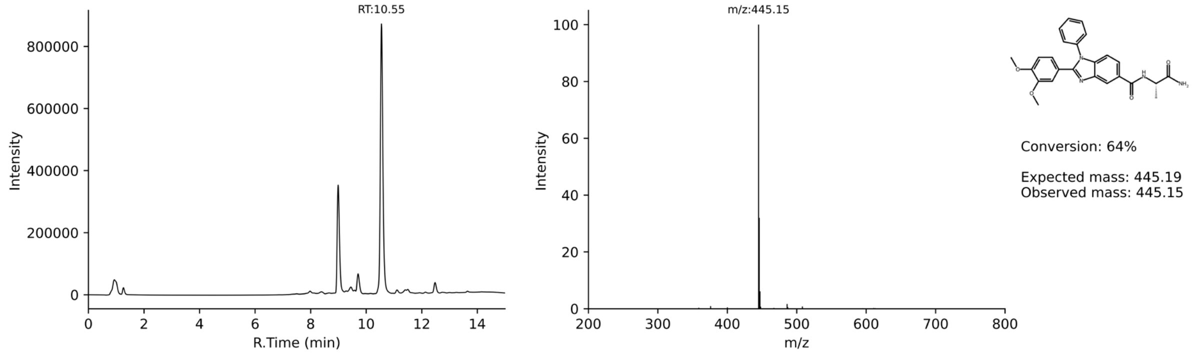

Supplementary Fig 160. LC-MS chromatogram obtained using 3,4-dimethoxybenzaldehyde.

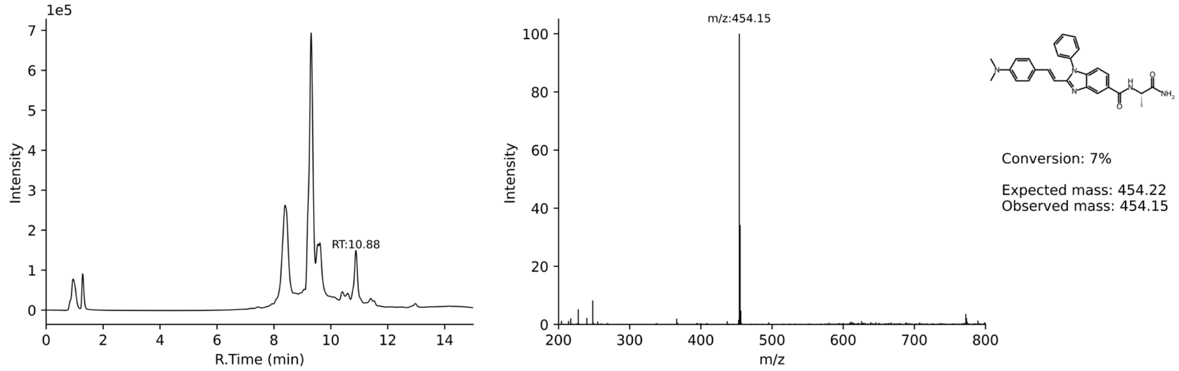

Supplementary Fig 161. LC-MS chromatogram obtained using 4-dimethylaminocinnamaldehyde.

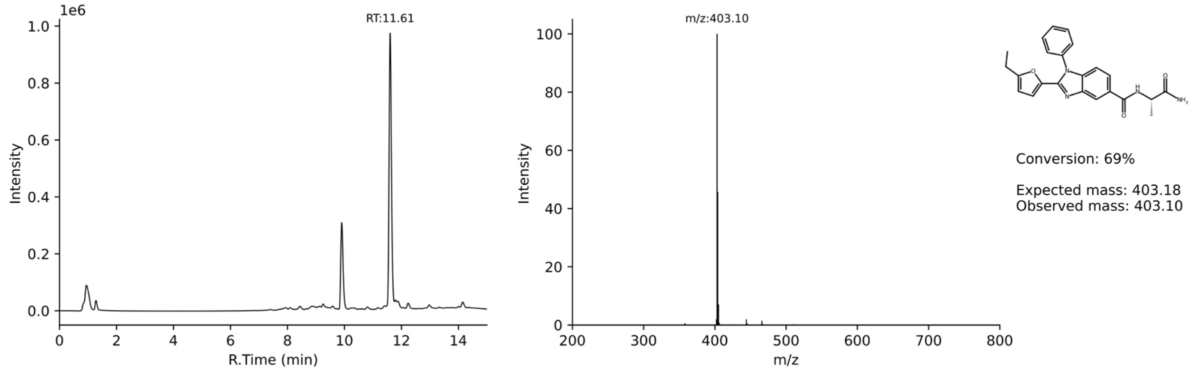

Supplementary Fig 162. LC-MS chromatogram obtained using 5-ethyl-2-furaldehyde.

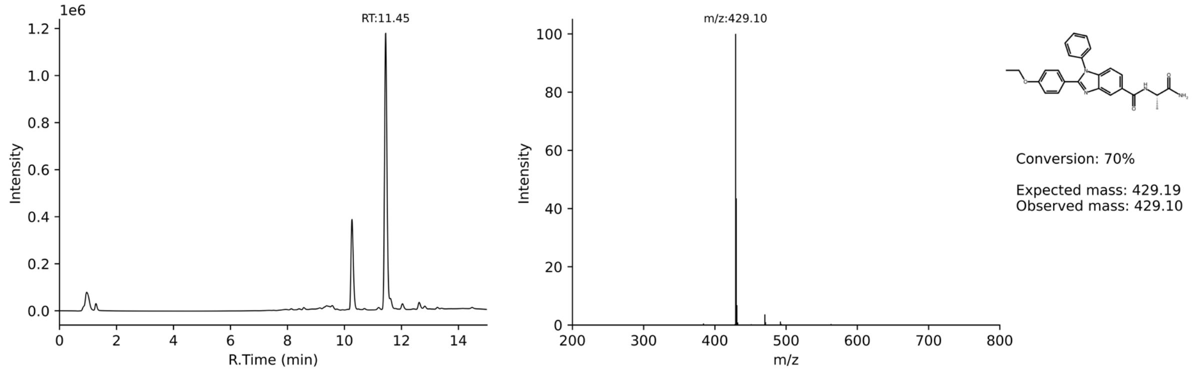

Supplementary Fig 163. LC-MS chromatogram obtained using 4-ethoxybenzaldehyde.

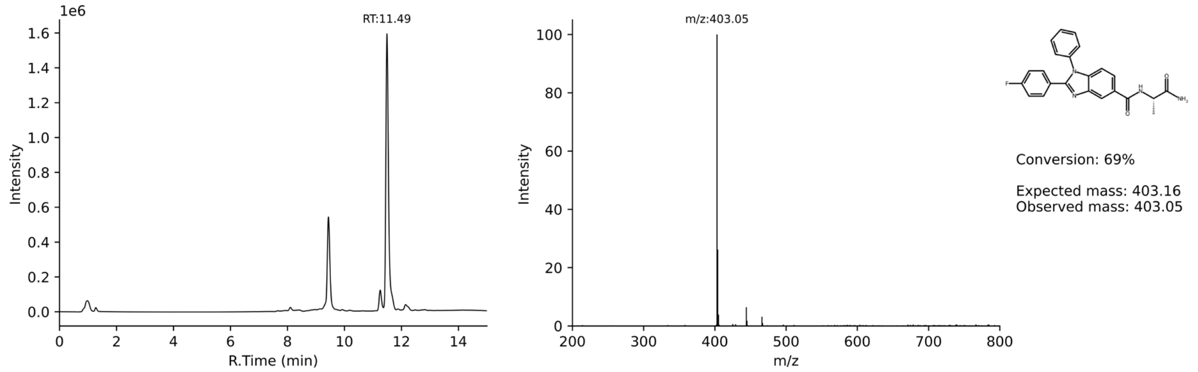

Supplementary Fig 164. LC-MS chromatogram obtained using 4-fluorobenzaldehyde.

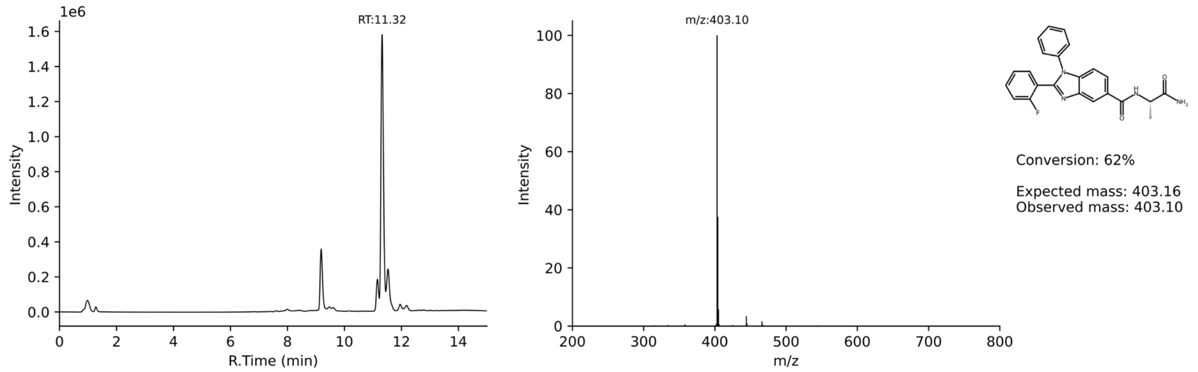

Supplementary Fig 165. LC-MS chromatogram obtained using 2-fluorobenzaldehyde.

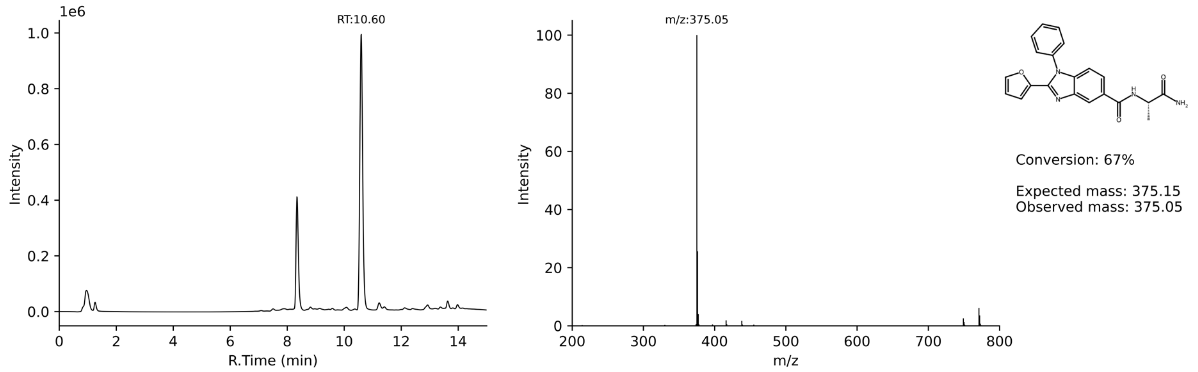

Supplementary Fig 166. LC-MS chromatogram obtained using furfural.

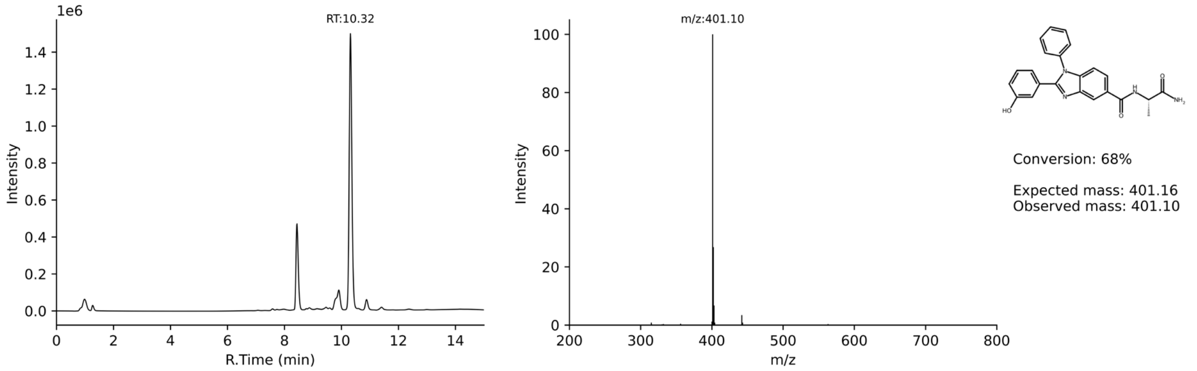

Supplementary Fig 167. LC-MS chromatogram obtained using 3-hydroxy benzaldehyde.

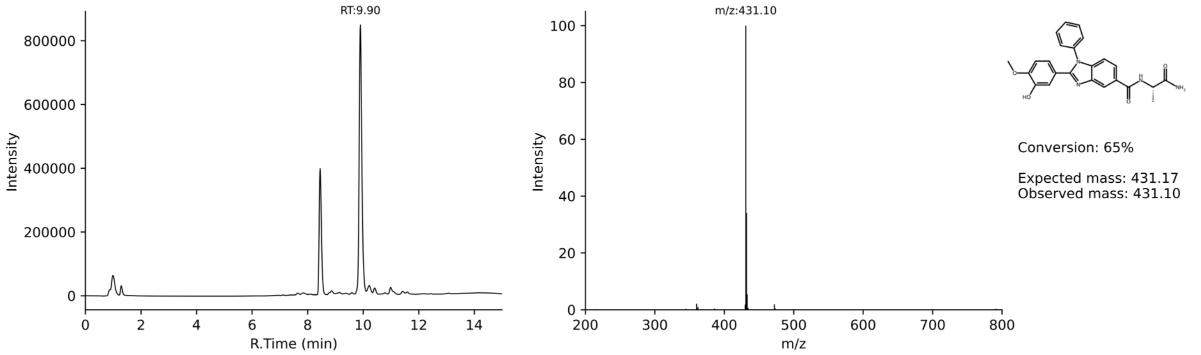

Supplementary Fig 168. LC-MS chromatogram obtained using 3-hydroxy-4-methoxybenzaldehyde.

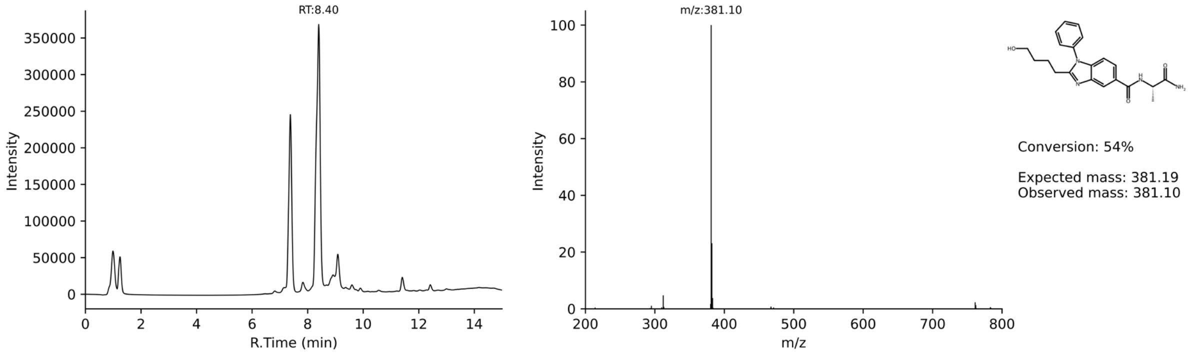

Supplementary Fig 169. LC-MS chromatogram obtained using 5-hydroxypentanal.

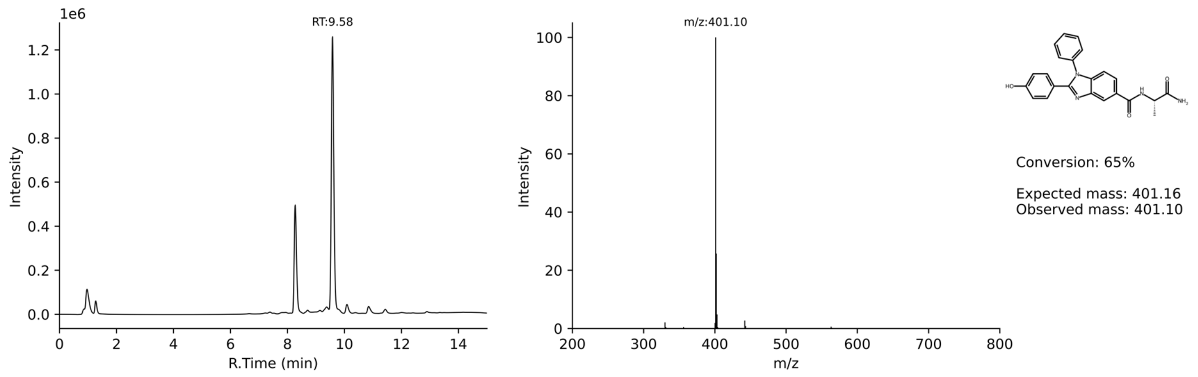

Supplementary Fig 170. LC-MS chromatogram obtained using 4-hydroxybenzaldehyde.

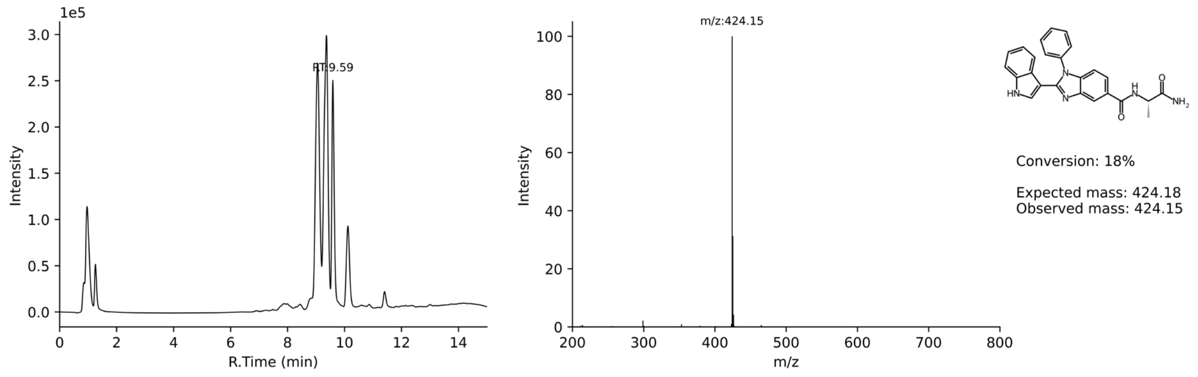

Supplementary Fig 171. LC-MS chromatogram obtained using indole-3-carboxaldehyde.

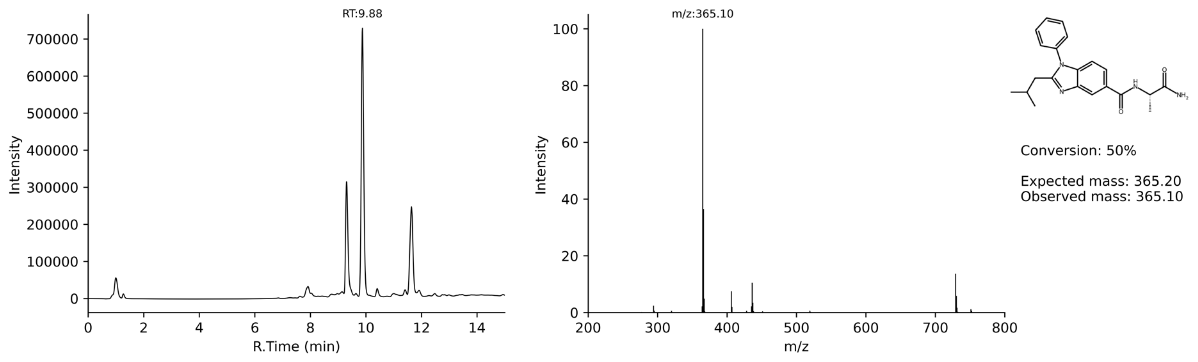

Supplementary Fig 172. LC-MS chromatogram obtained using isovaleraldehyde.

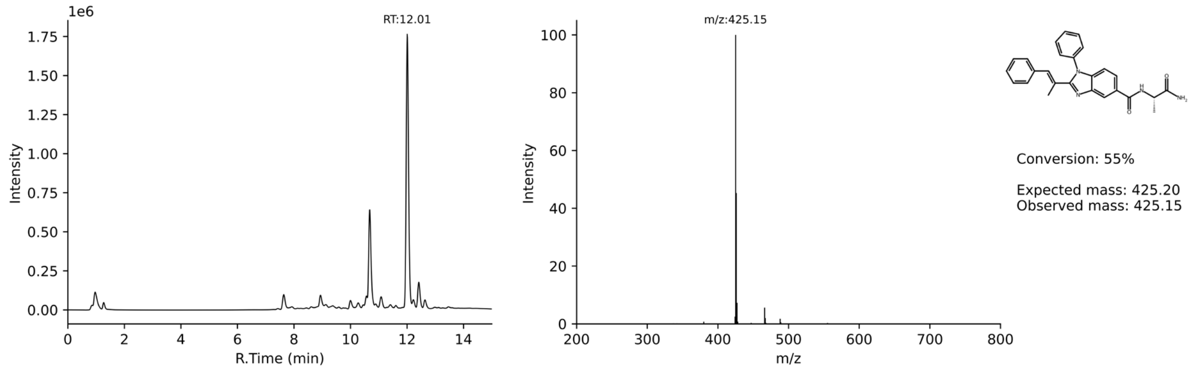

Supplementary Fig 173. LC-MS chromatogram obtained using alpha-methylcinnamaldehyde.

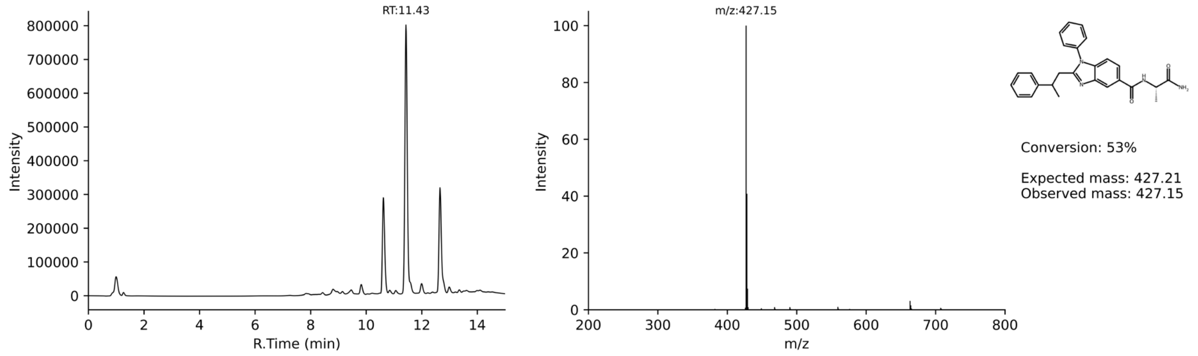

Supplementary Fig 174. LC-MS chromatogram obtained using 3-phenylbutyaldehyde.

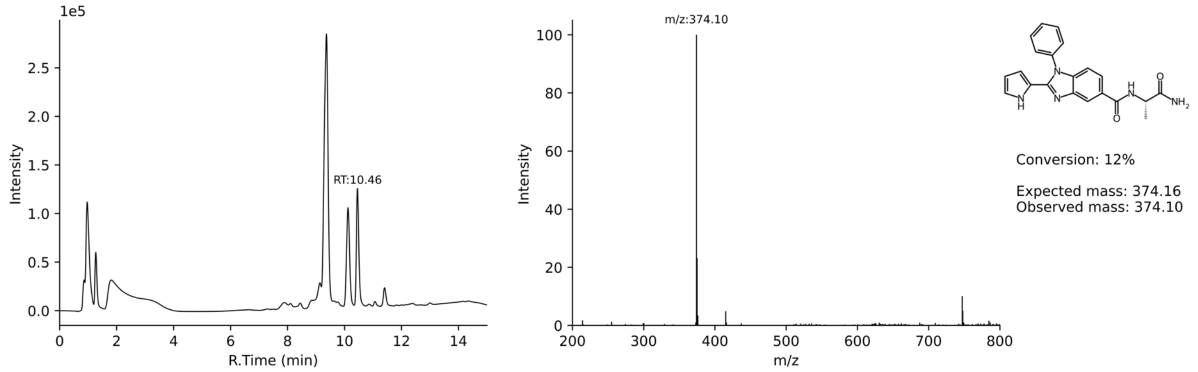

Supplementary Fig 175. LC-MS chromatogram obtained using pyrrole-2-carboxaldehyde.

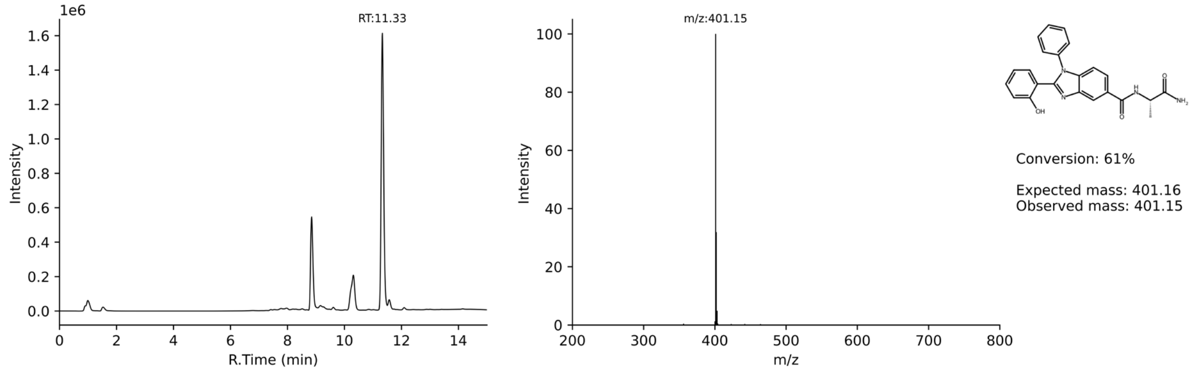

Supplementary Fig 176. LC-MS chromatogram obtained using salicylaldehyde.

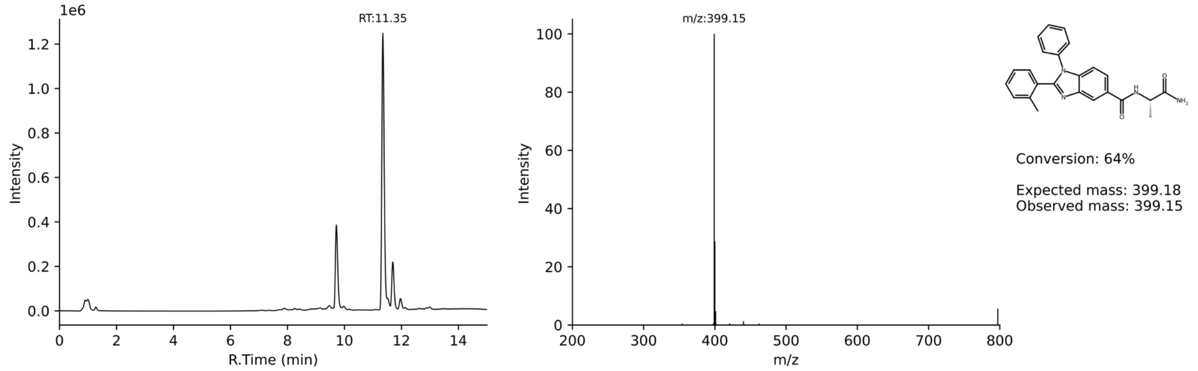

Supplementary Fig 177. LC-MS chromatogram obtained using 2-methyl benzaldehyde.

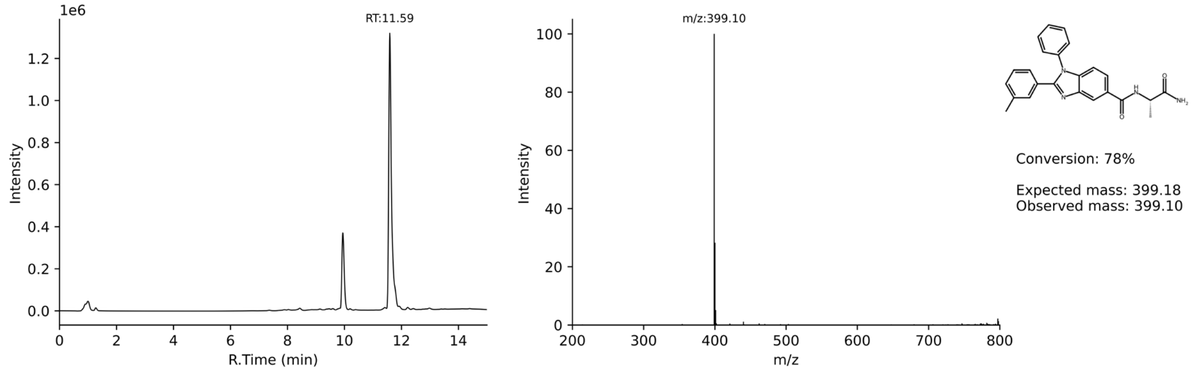

Supplementary Fig 178. LC-MS chromatogram obtained using 3-methyl benzaldehyde.

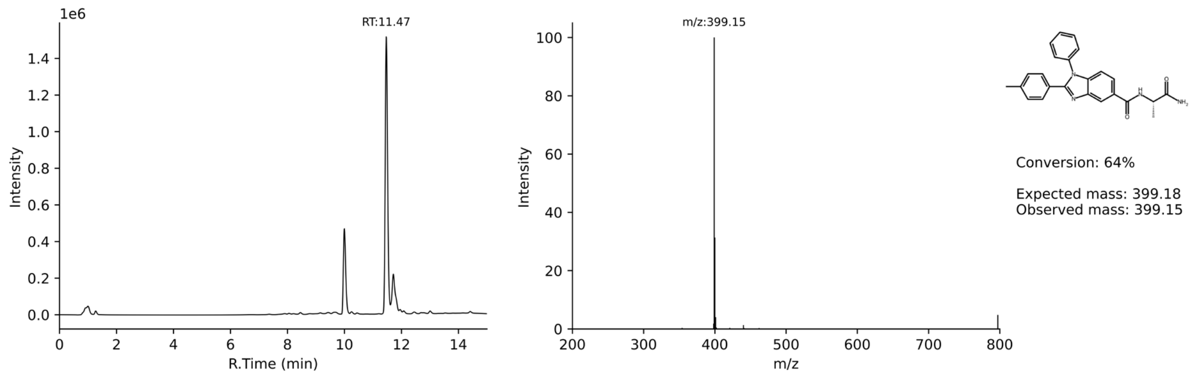

Supplementary Fig 179. LC-MS chromatogram obtained using 4-methyl benzaldehyde.

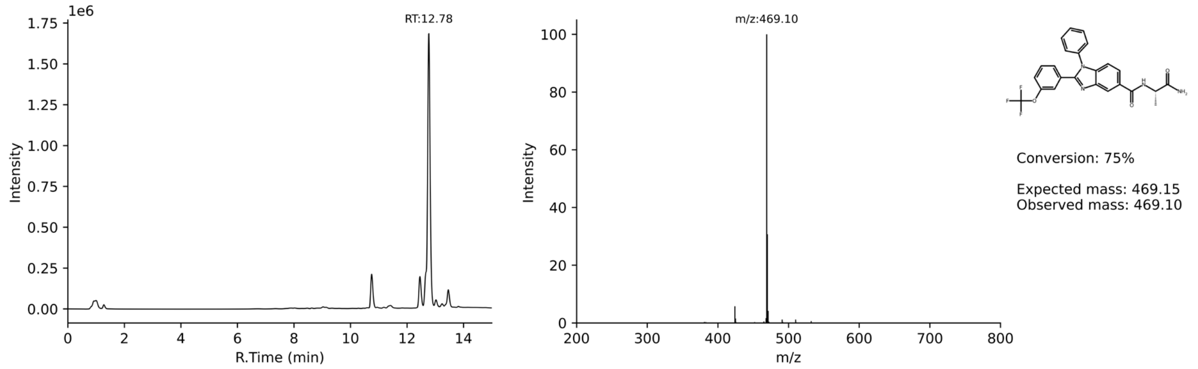

Supplementary Fig 180. LC-MS chromatogram obtained using 3-trifluoromethoxy benzaldehyde.

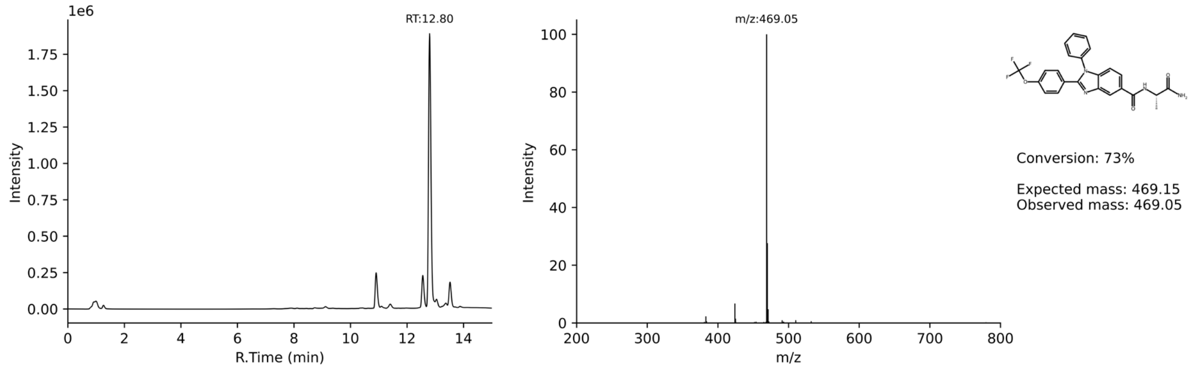

Supplementary Fig 181. LC-MS chromatogram obtained using 4-trifluoromethoxy benzaldehyde.

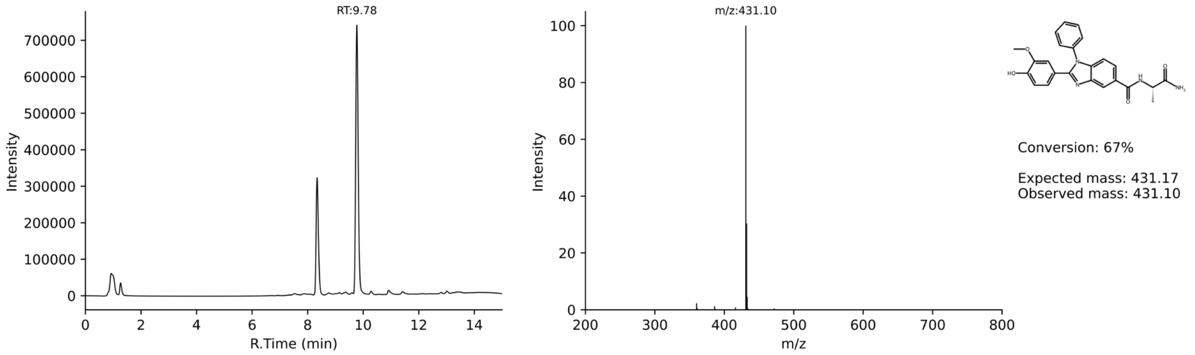

Supplementary Fig 182. LC-MS chromatogram obtained using vanillin.

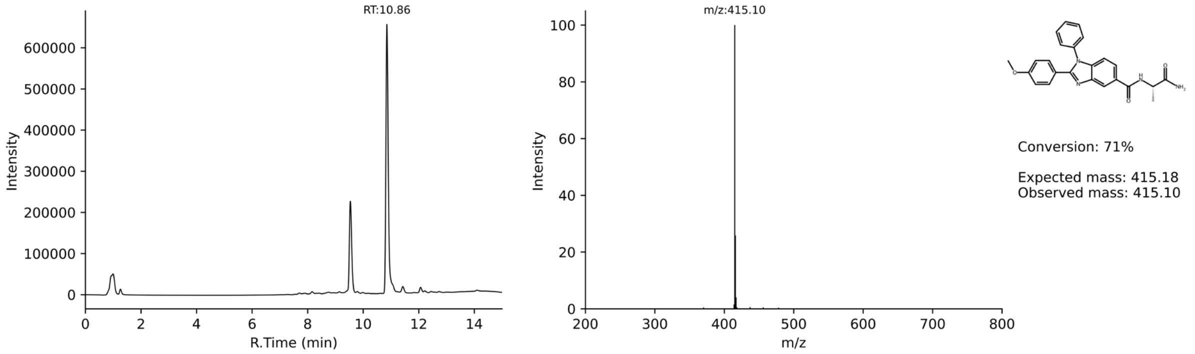

Supplementary Fig 183. LC-MS chromatogram obtained using 4-methoxybenzaldehyde.

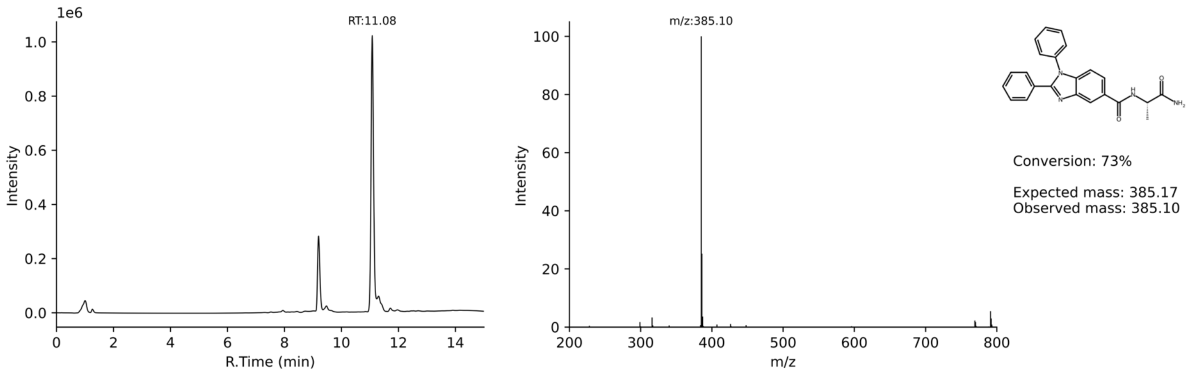

Supplementary Fig 184. LC-MS chromatogram obtained using benzaldehyde.

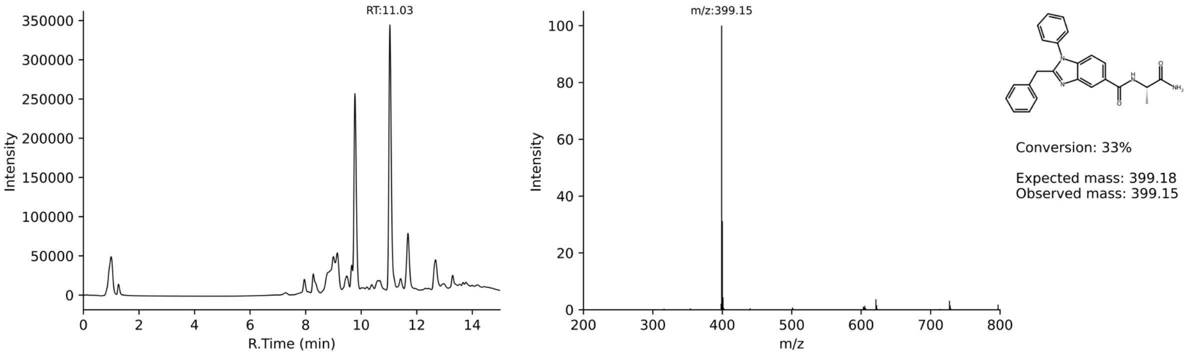

Supplementary Fig 185. LC-MS chromatogram obtained using phenylacetaldehyde.

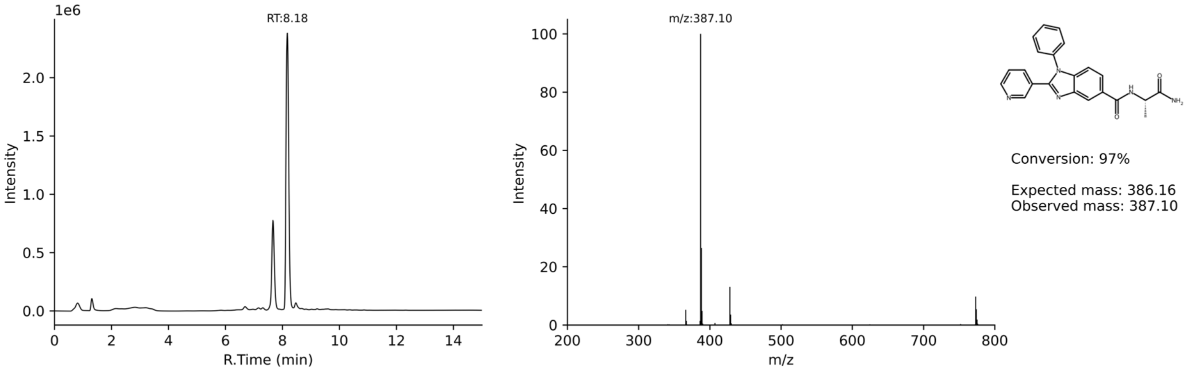

Supplementary Fig 186. LC-MS chromatogram obtained using nicotinaldehyde.

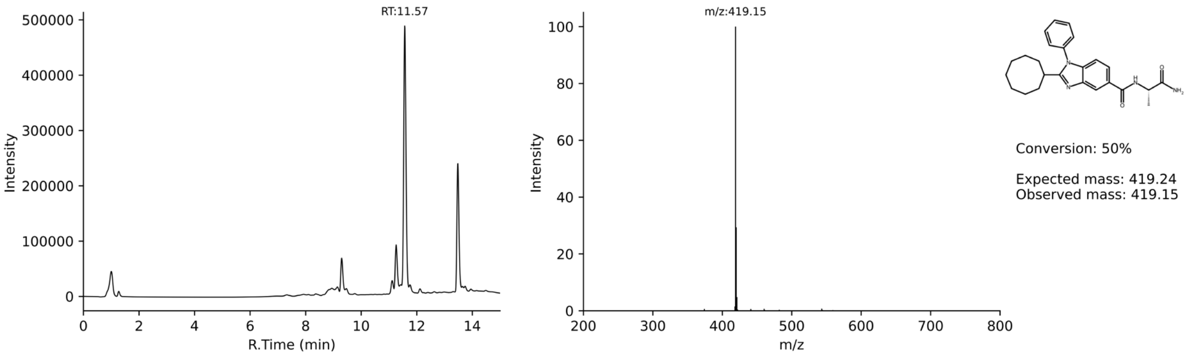

Supplementary Fig 187. LC-MS chromatogram obtained using cyclooctane carboxaldehyde.

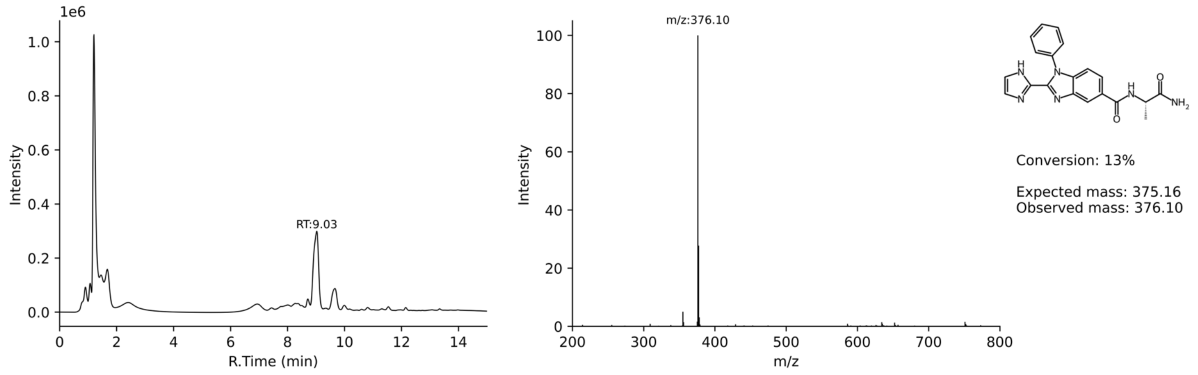

Supplementary Fig 188. LC-MS chromatogram obtained using 1h-imidazole-2-carbaldehyde.

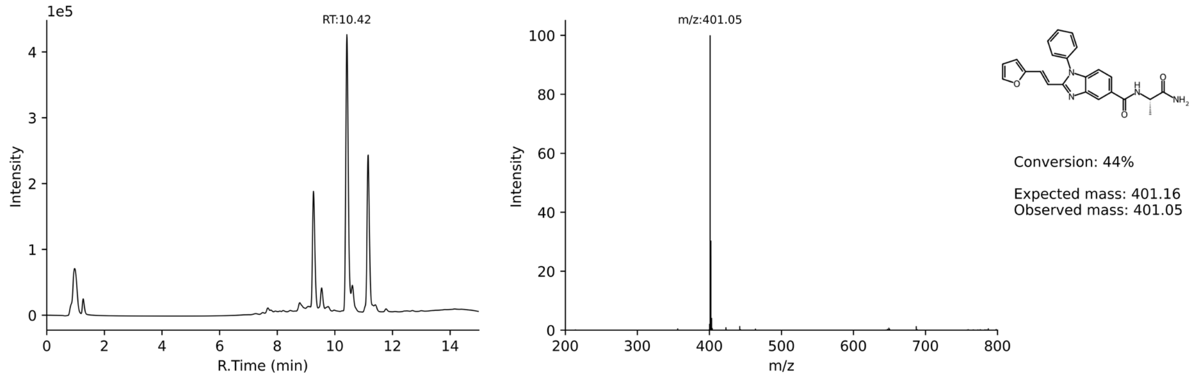

Supplementary Fig 189. LC-MS chromatogram obtained using 3-(2-furyl)-acrolein.

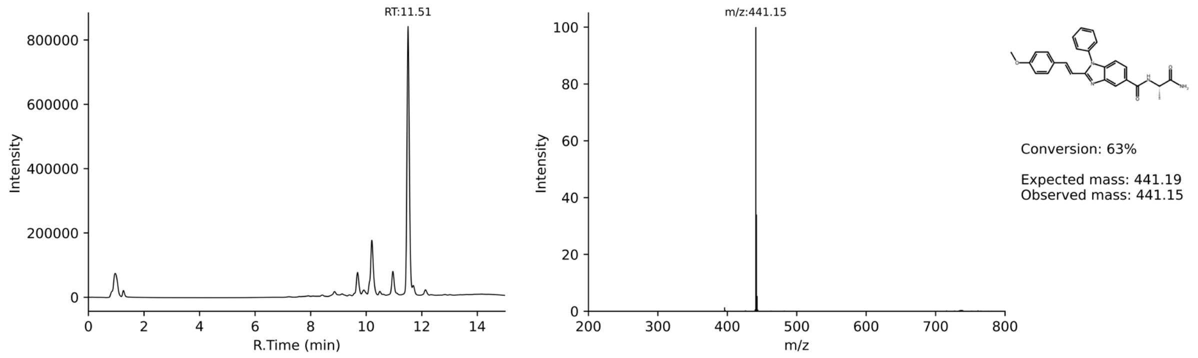

Supplementary Fig 190. LC-MS chromatogram obtained using 4-methoxycinnamaldehyde.

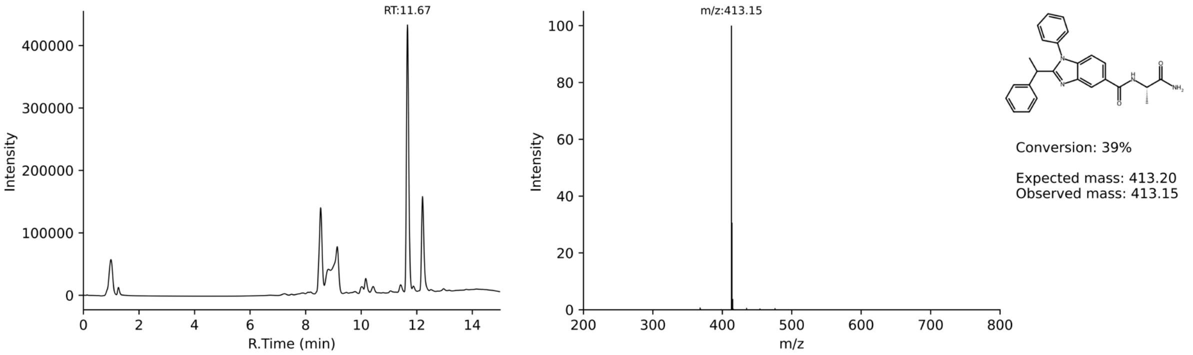

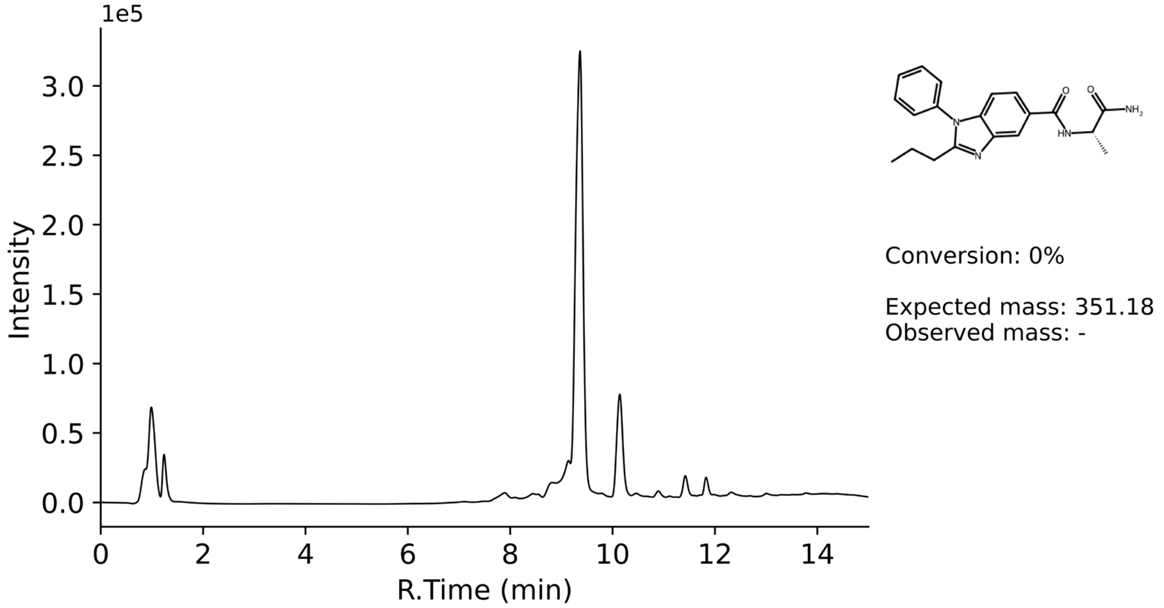

Supplementary Fig 191. LC-MS chromatogram obtained using 2-phenylpropionaldehyde.

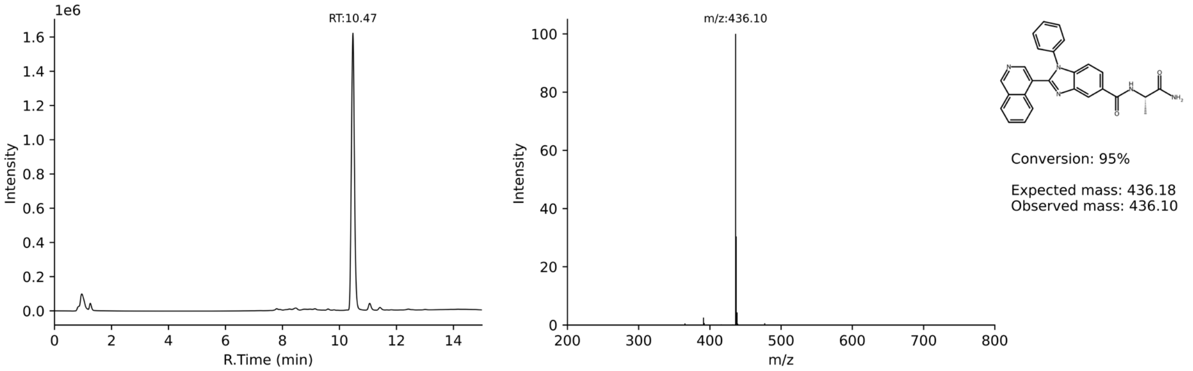

Supplementary Fig 192. LC-MS chromatogram obtained using isoquinoline-4-carboxaldehyde.

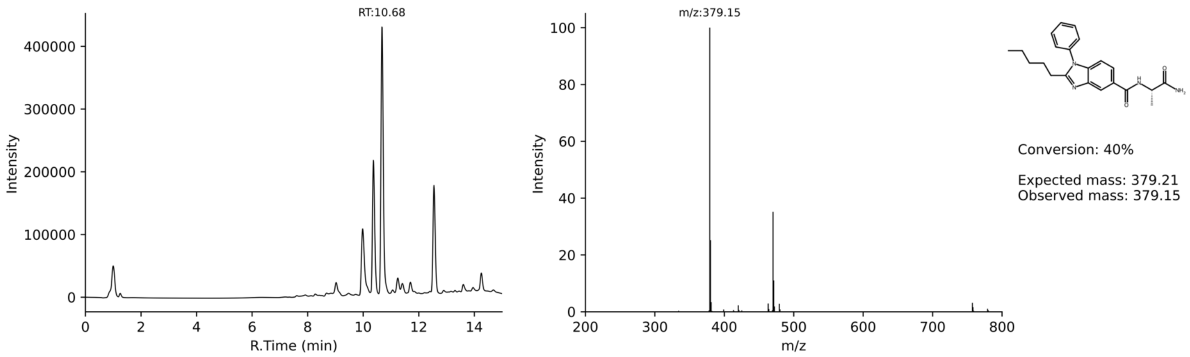

Supplementary Fig 193. LC-MS chromatogram obtained using hexanal.

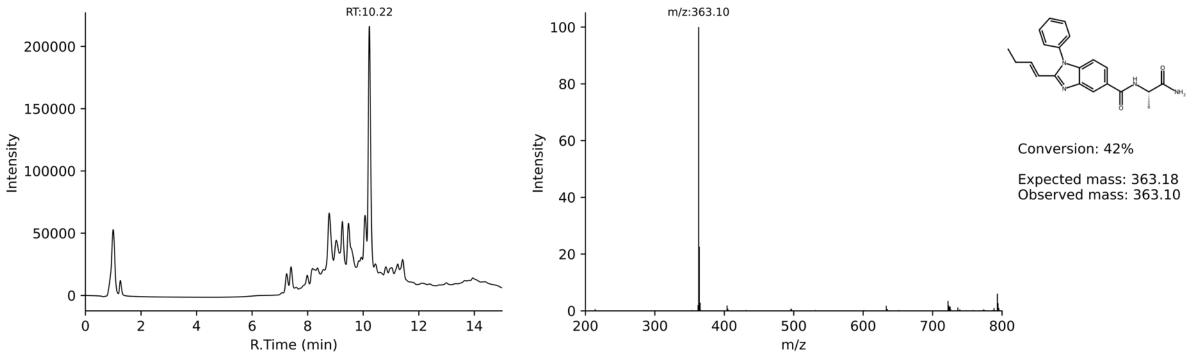

Supplementary Fig 194. LC-MS chromatogram obtained using trans 2-pentenal.

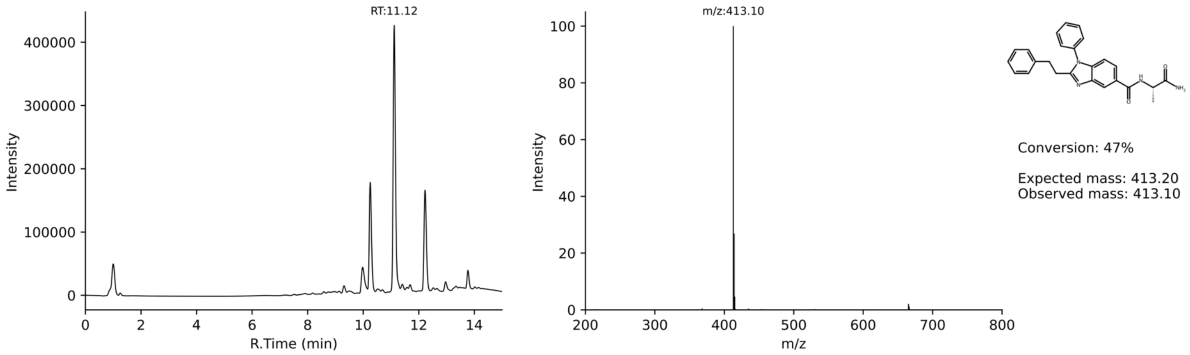

Supplementary Fig 195. LC-MS chromatogram obtained using 3-phenylpropionaldehyde.

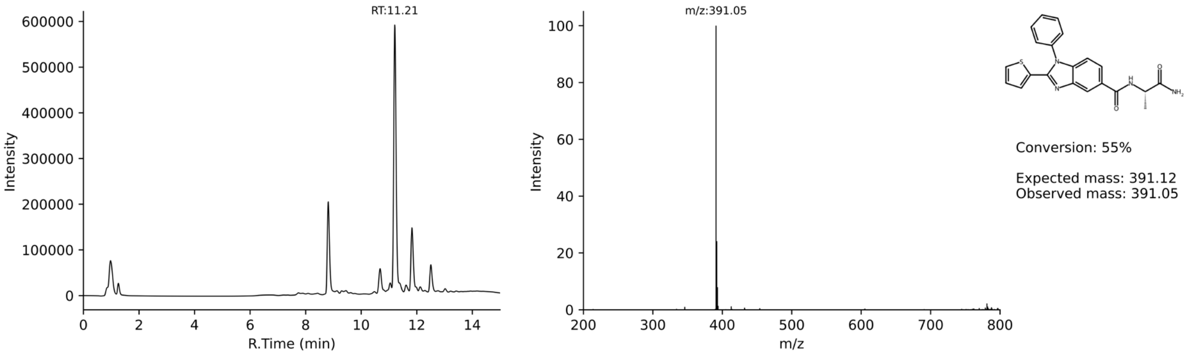

Supplementary Fig 196. LC-MS chromatogram obtained using 2-thiophene-carboxaldehyde.

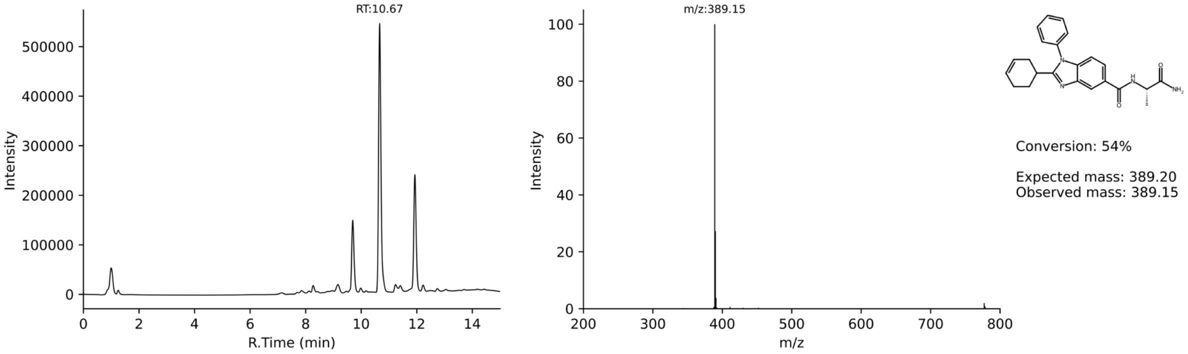

Supplementary Fig 197. LC-MS chromatogram obtained using 1,2,3,6-tetrahydrobenzaldehyde.

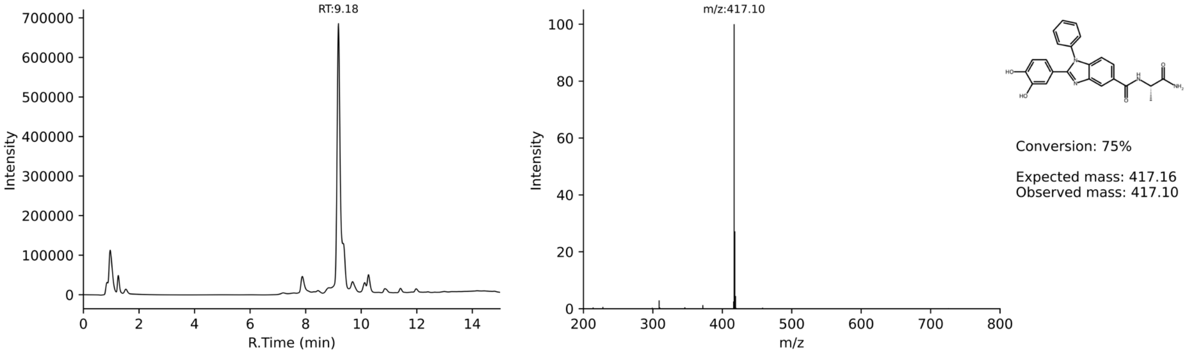

Supplementary Fig 198. LC-MS chromatogram obtained using 3,4-dihydroxybenzaldehyde.

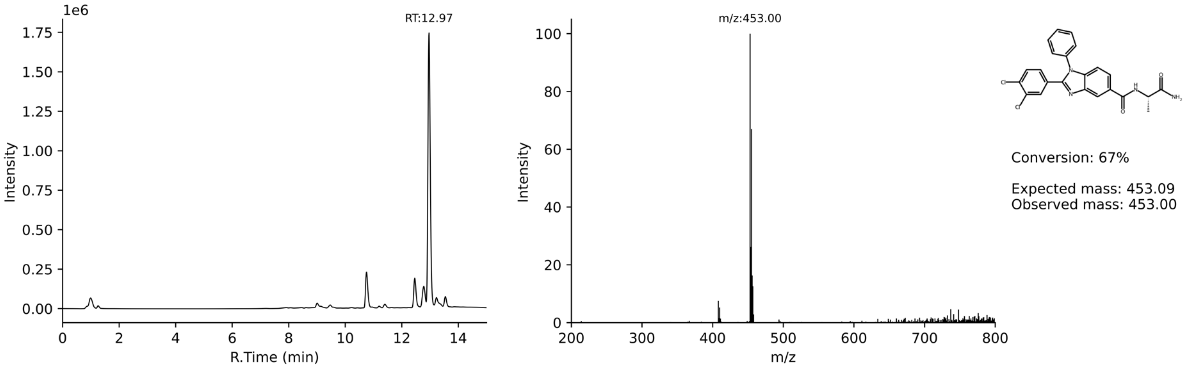

Supplementary Fig 199. LC-MS chromatogram obtained using 3,4-dichlorobenzaldehyde.

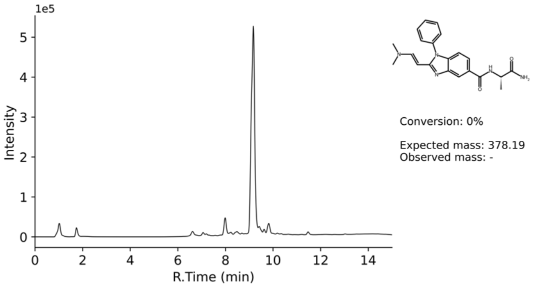

Supplementary Fig 200. LC-MS chromatogram obtained using (e)-3-(dimethylamino)acrylaldehyde.

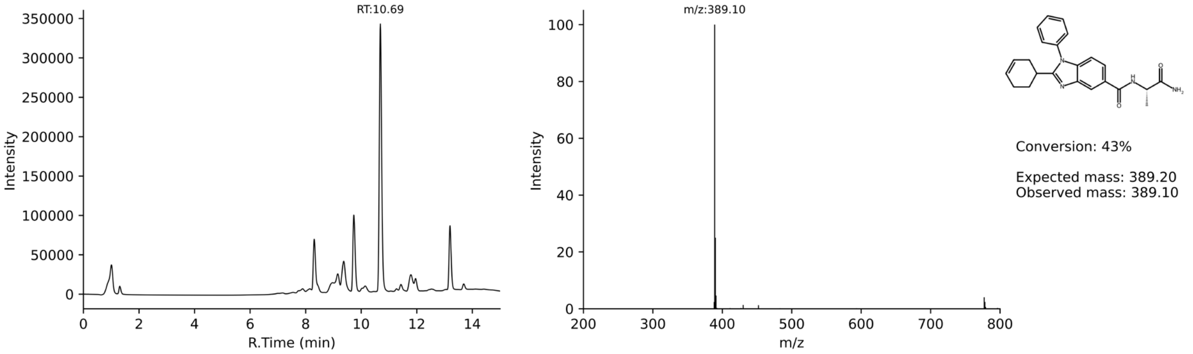

Supplementary Fig 201. LC-MS chromatogram obtained using 3-cyclohexene-1-carboxaldehyde.

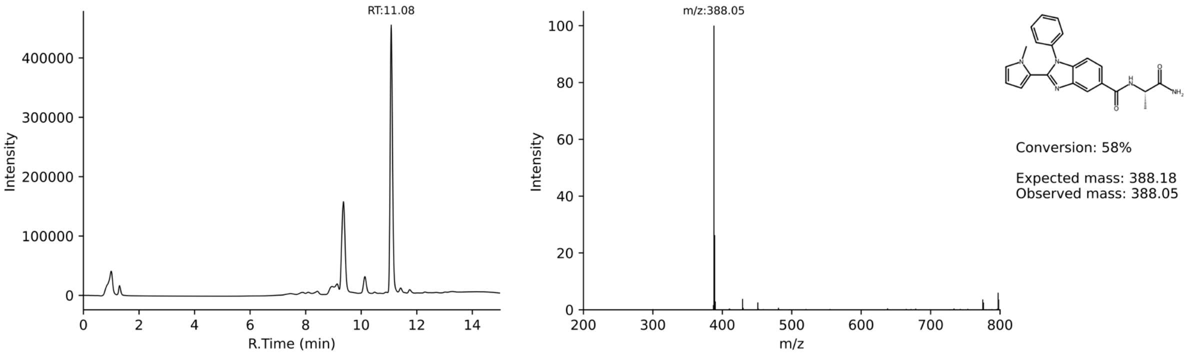

Supplementary Fig 202. LC-MS chromatogram obtained using 1-methyl-1h-pyrrole-3-carbaldehyde.

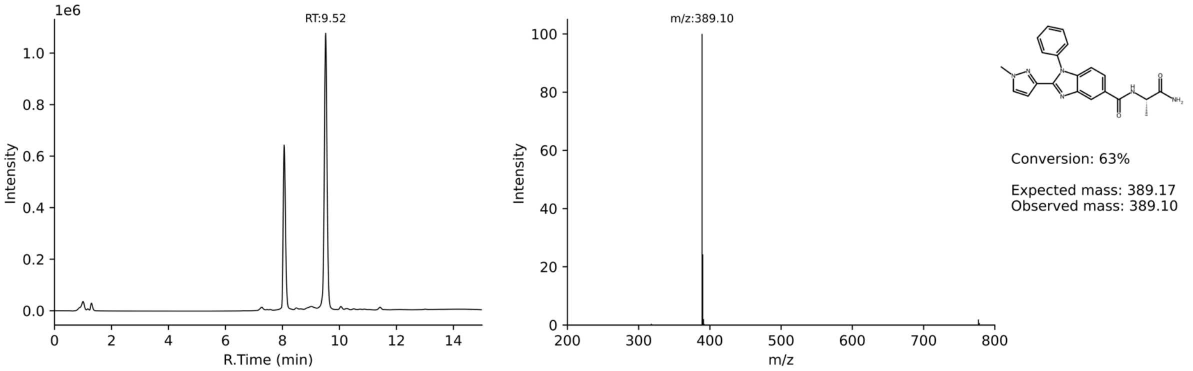

Supplementary Fig 203. LC-MS chromatogram obtained using 1-methyl-1h-pyrazole-3-carbaldehyde.

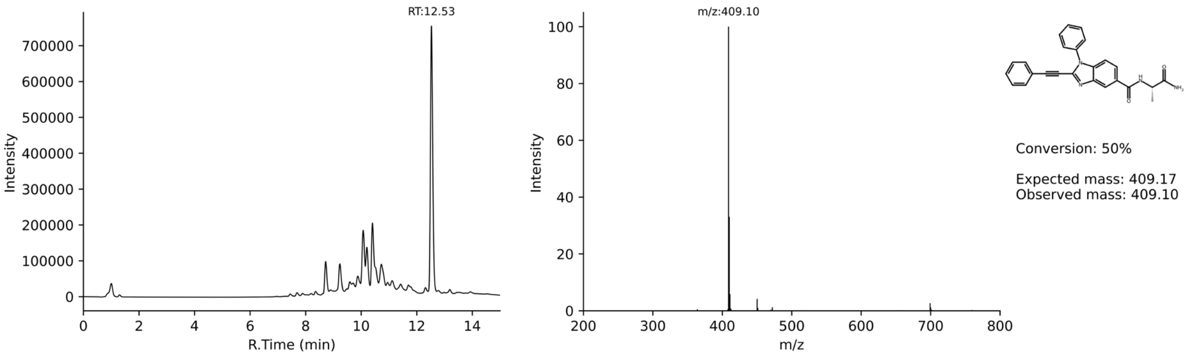

Supplementary Fig 204. LC-MS chromatogram obtained using 3-phenylpropiolaldehyde.

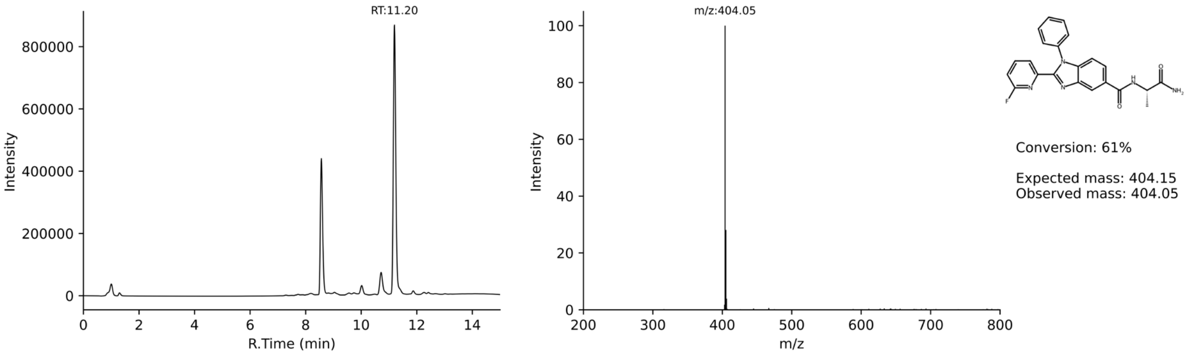

Supplementary Fig 205. LC-MS chromatogram obtained using 6-fluoropicolinaldehyde.

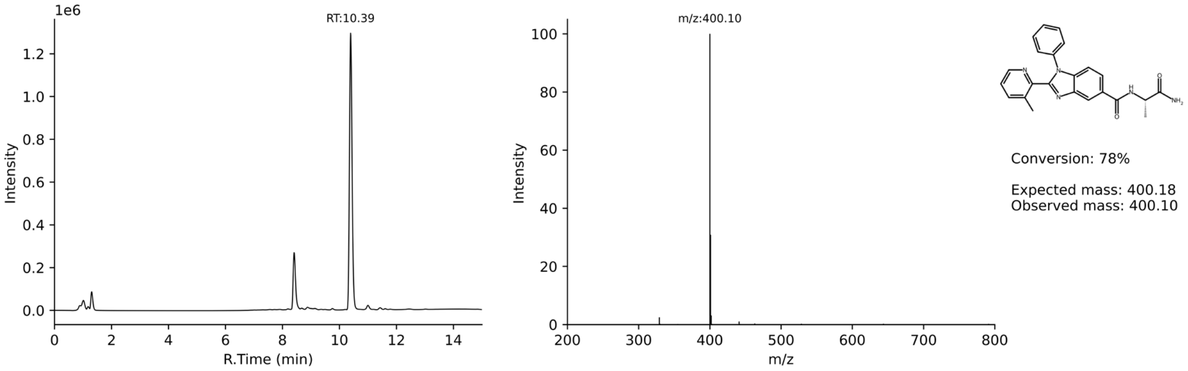

Supplementary Fig 206. LC-MS chromatogram obtained using 3-methylpicolinaldehyde.

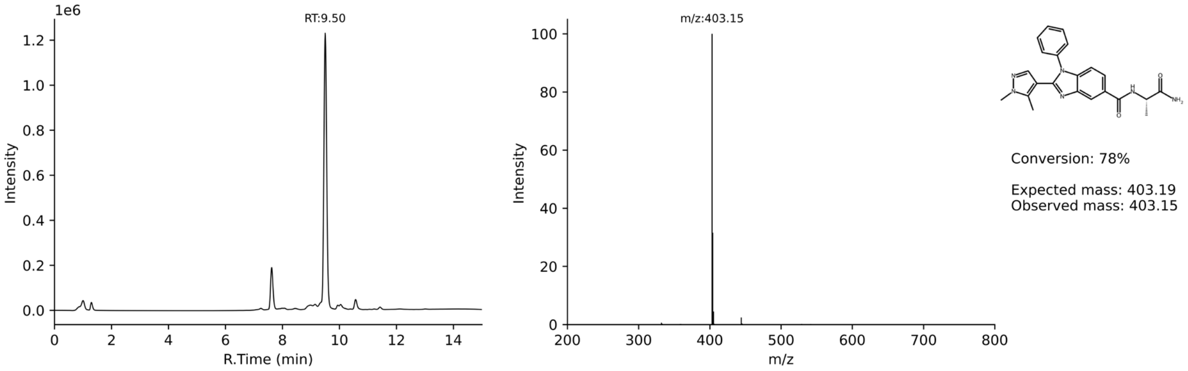

Supplementary Fig 207. LC-MS chromatogram obtained using 1,5-dimethyl-1h-pyrazole-4-carbaldehyde.

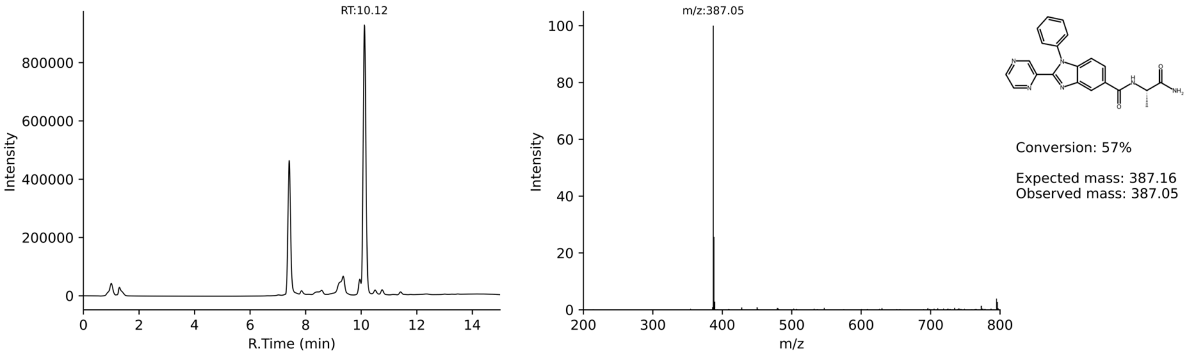

Supplementary Fig 208. LC-MS chromatogram obtained using pyrazine-2-carbaldehyde.

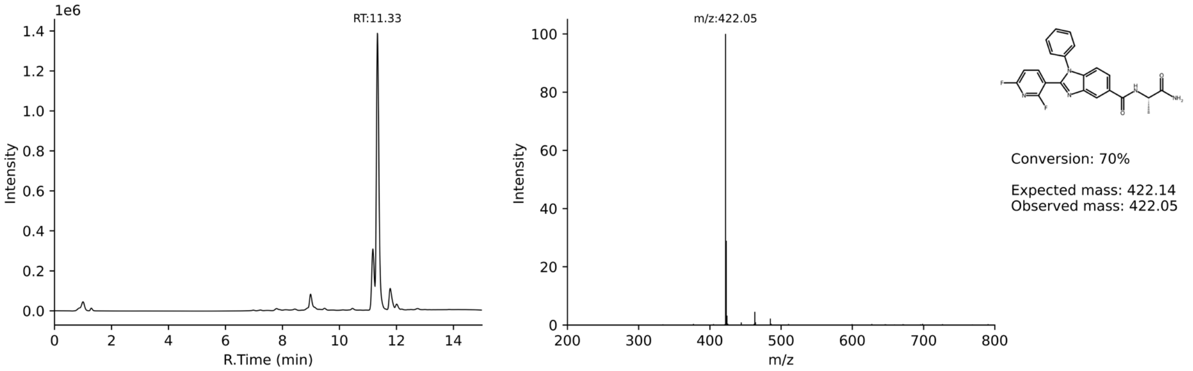

Supplementary Fig 209. LC-MS chromatogram obtained using 2,6-difluoronicotinaldehyde.

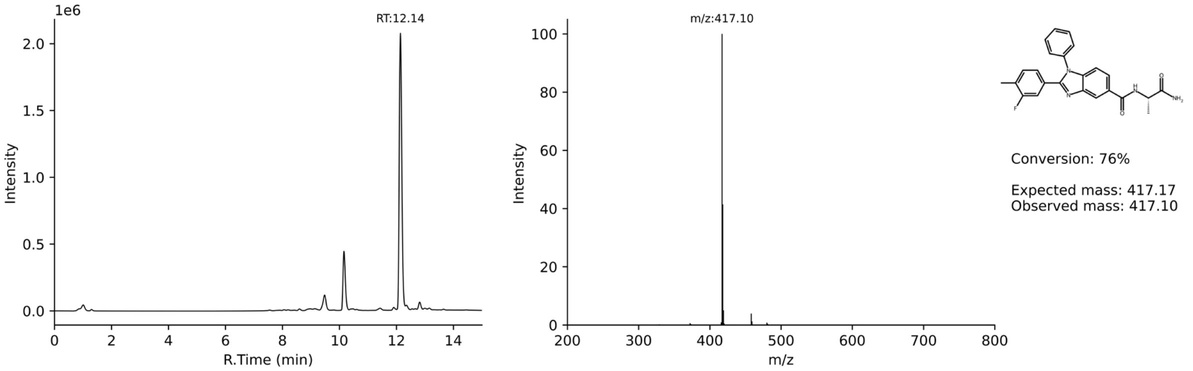

Supplementary Fig 210. LC-MS chromatogram obtained using 3-fluoro-4-methylbenzaldehyde.

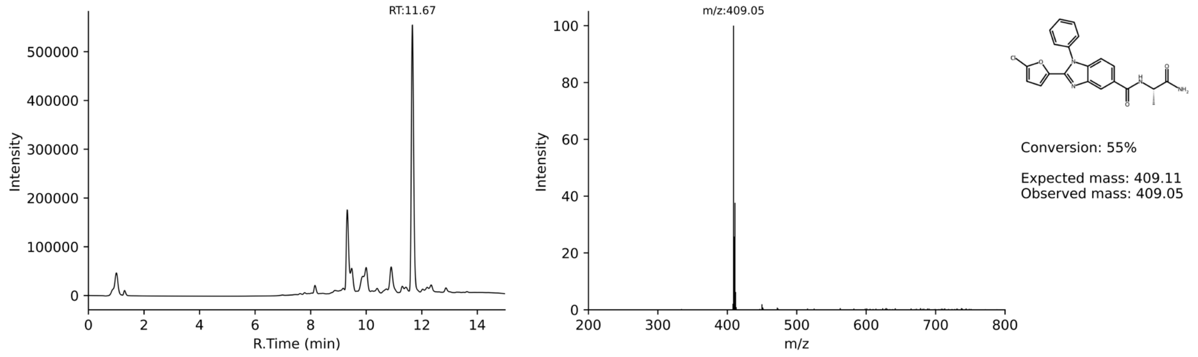

Supplementary Fig 211. LC-MS chromatogram obtained using 5-chlorofuran-2-carbaldehyde.

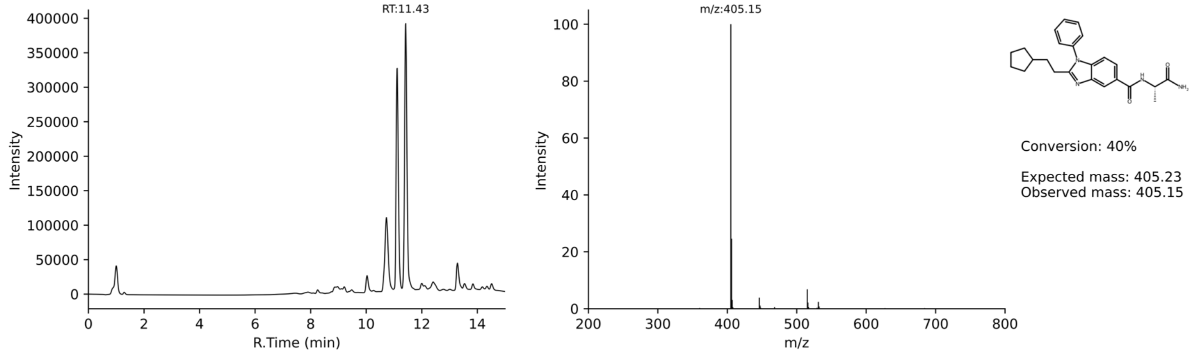

Supplementary Fig 212. LC-MS chromatogram obtained using 3-cyclopentylpropanal.

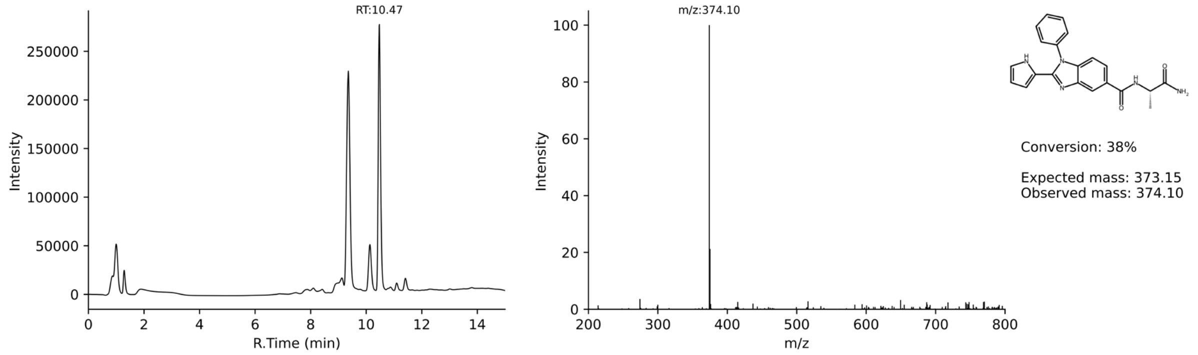

Supplementary Fig 213. LC-MS chromatogram obtained using 1h-pyrrole-2-carbaldehyde.

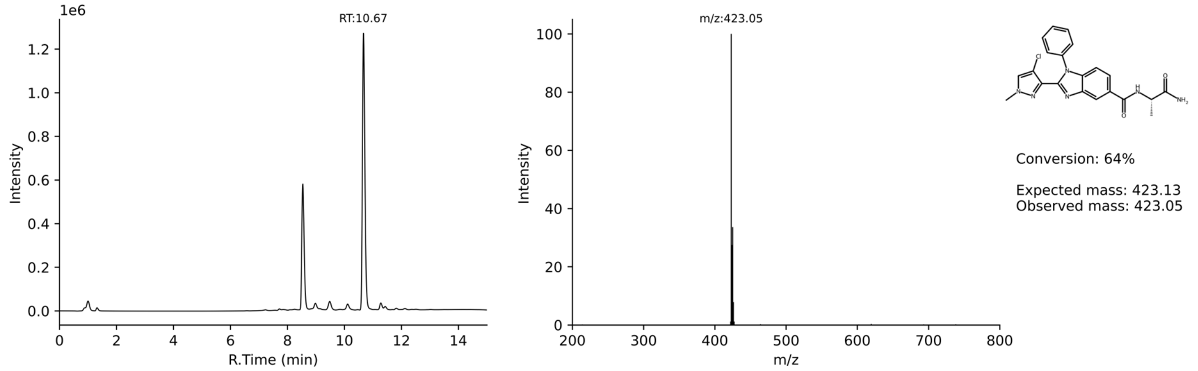

Supplementary Fig 214. LC-MS chromatogram obtained using 4-chloro-1-methyl-1h-pyrazole-3-carbaldehyde.

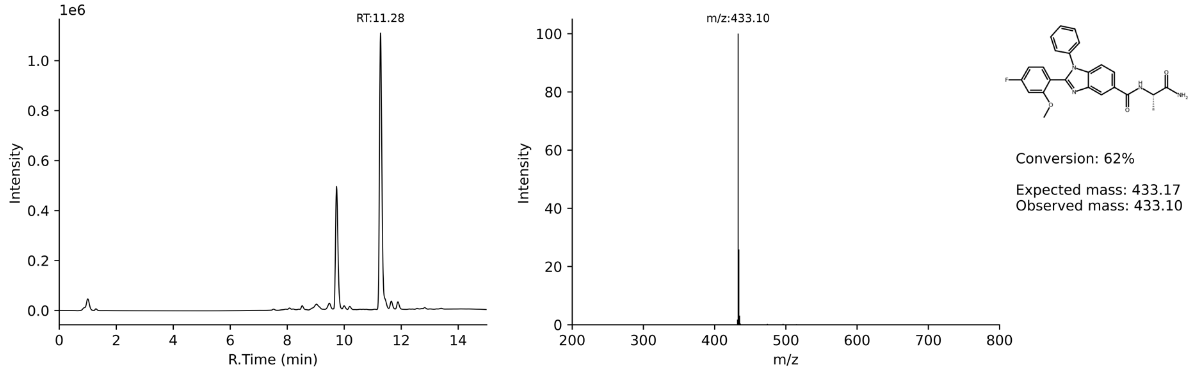

Supplementary Fig 215. LC-MS chromatogram obtained using 4-fluoro-2-methoxybenzaldehyde.

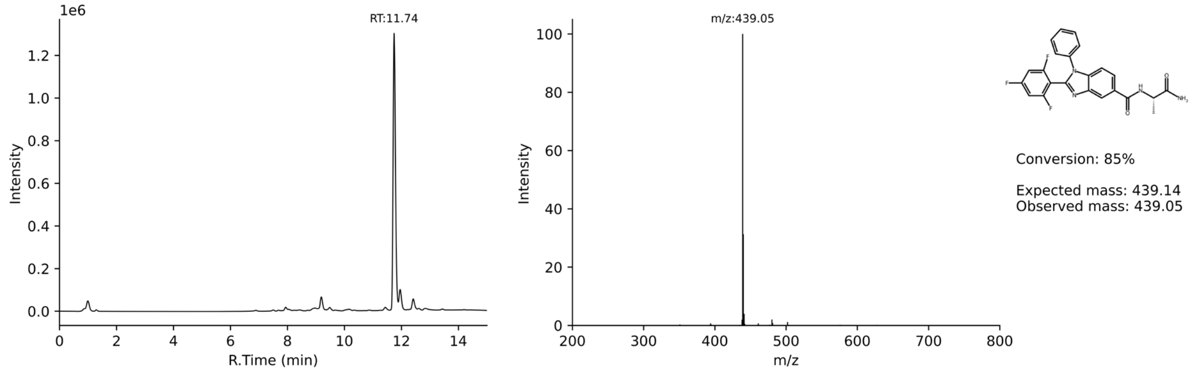

Supplementary Fig 216. LC-MS chromatogram obtained using 2,4,6-trifluorobenzaldehyde.

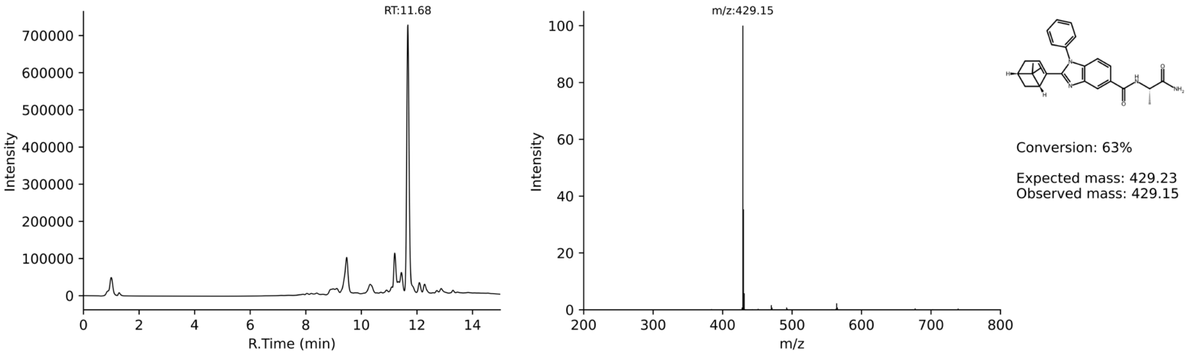

Supplementary Fig 217. LC-MS chromatogram obtained using (1r,5s)-6,6-dimethylbicyclo[3.1.1]hept-2-ene-2-carbaldehyde.

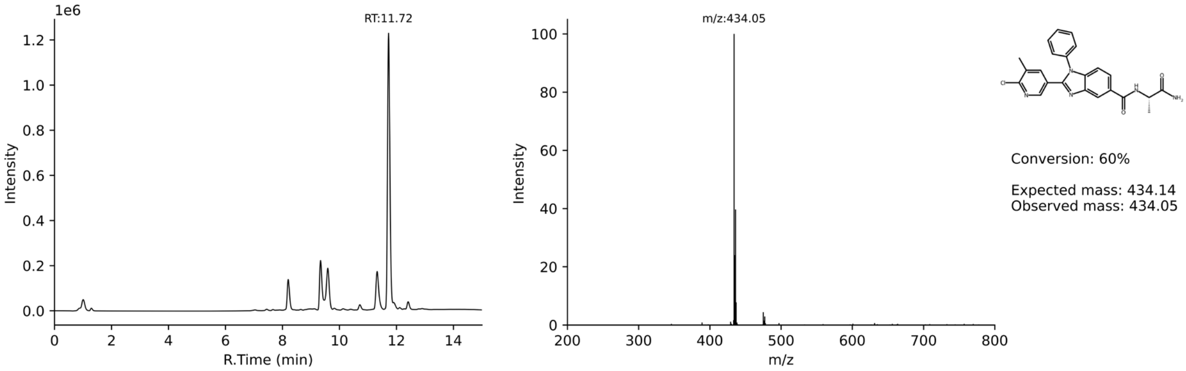

Supplementary Fig 218. LC-MS chromatogram obtained using 6-chloro-5-methylnicotinaldehyde.

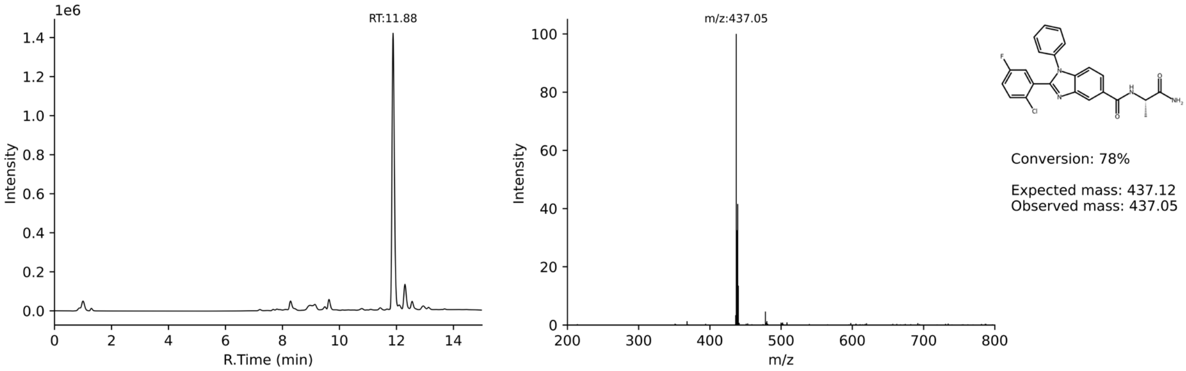

Supplementary Fig 219. LC-MS chromatogram obtained using 2-chloro-5-fluorobenzaldehyde.

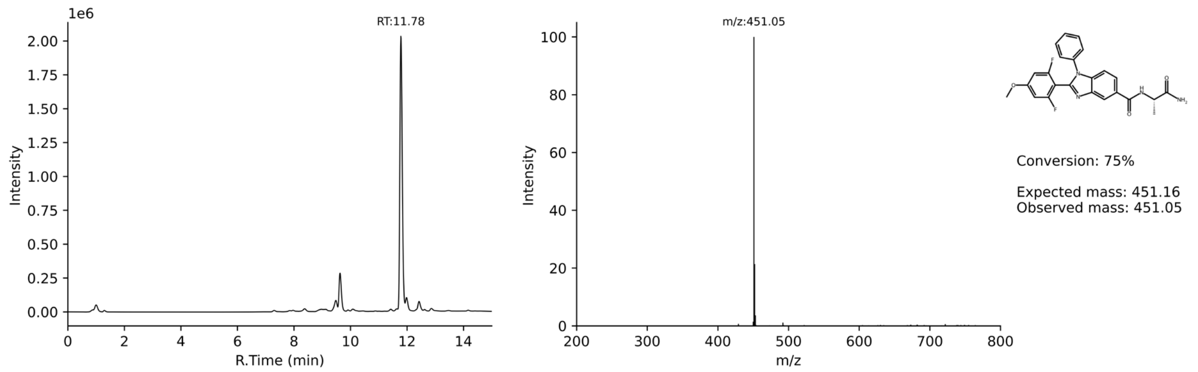

Supplementary Fig 220. LC-MS chromatogram obtained using 2,6-difluoro-4-methoxybenzaldehyde.

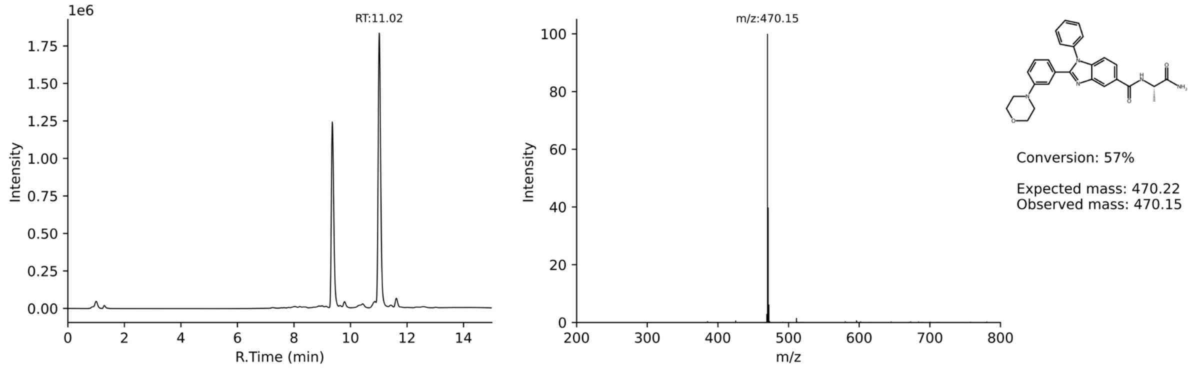

Supplementary Fig 221. LC-MS chromatogram obtained using 3-morpholinobenzaldehyde.

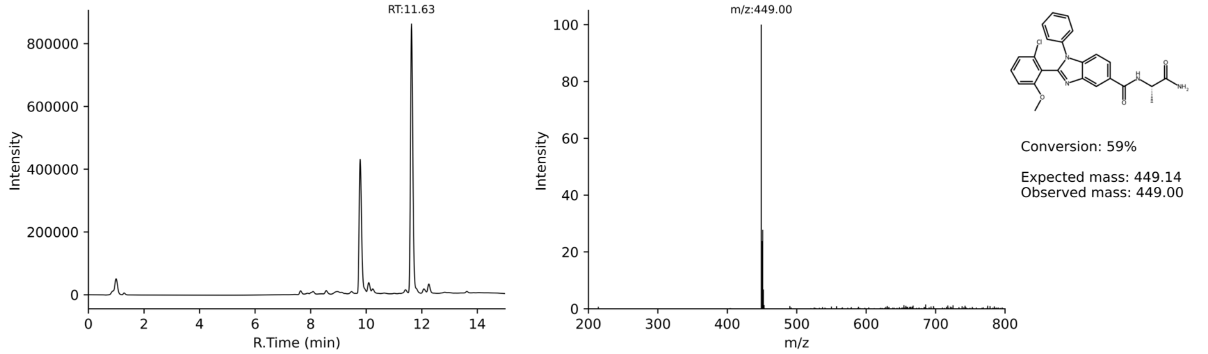

Supplementary Fig 222. LC-MS chromatogram obtained using 2-chloro-6-methoxybenzaldehyde.

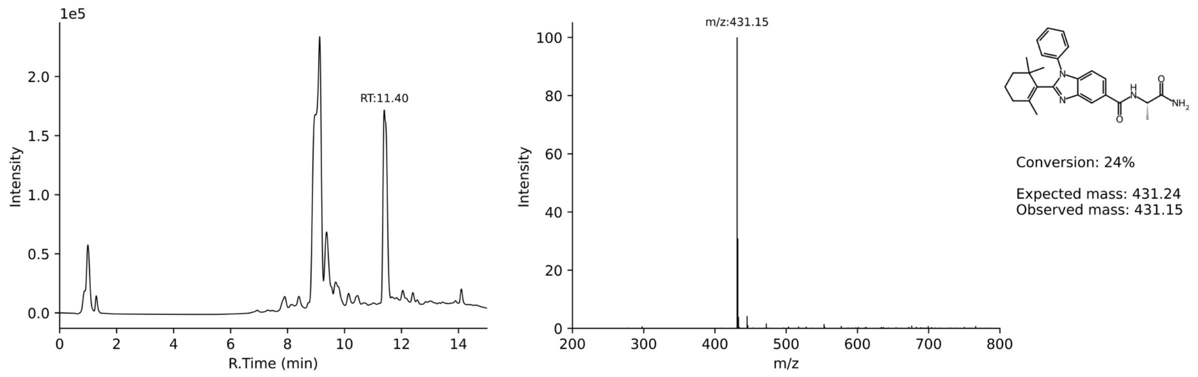

Supplementary Fig 223. LC-MS chromatogram obtained using 2,6,6-trimethylcyclohex-1-ene-1-carbaldehyde.

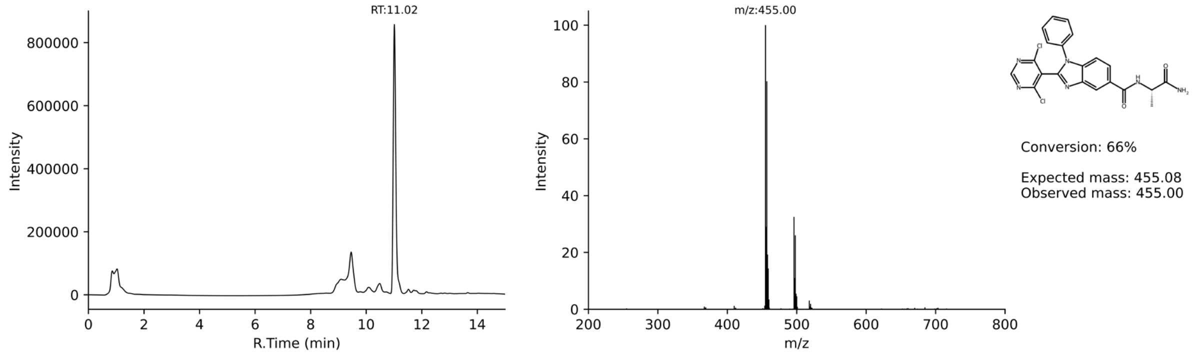

Supplementary Fig 224. LC-MS chromatogram obtained using 4,6-dichloropyrimidine-5-carbaldehyde.

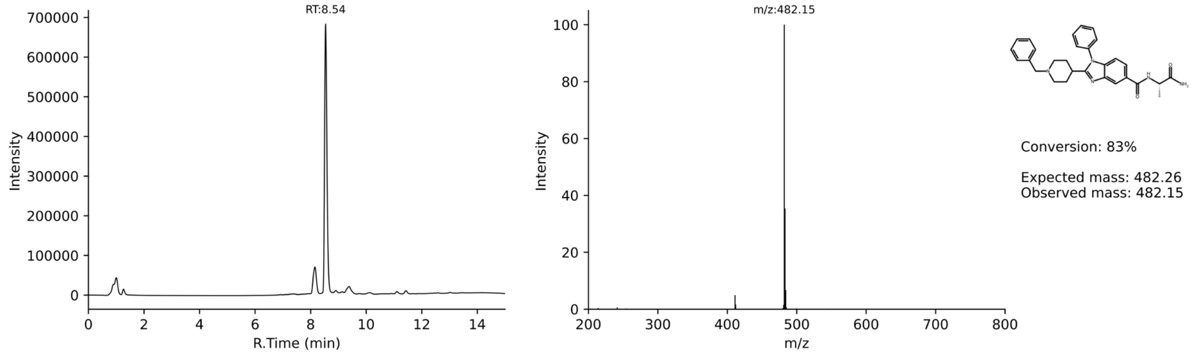

Supplementary Fig 225. LC-MS chromatogram obtained using 1-benzylpiperidine-4-carbaldehyde.

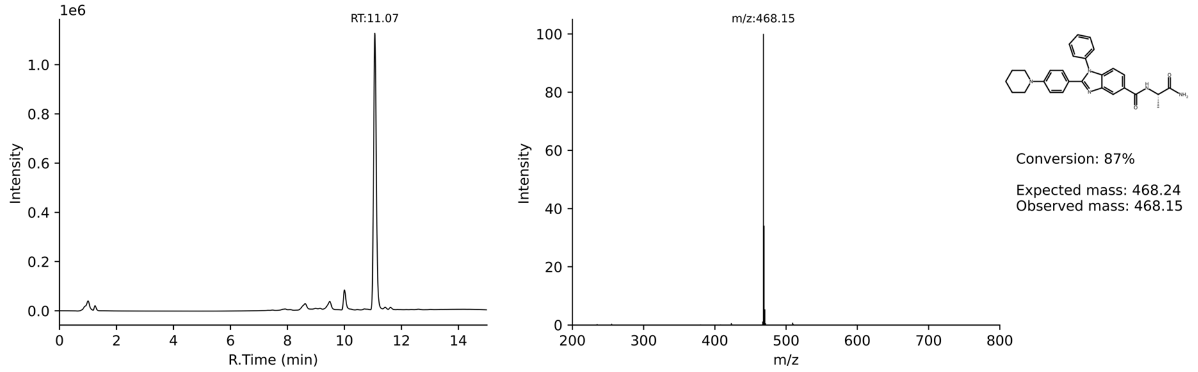

Supplementary Fig 226. LC-MS chromatogram obtained using 4-(piperidin-1-yl)benzaldehyde.

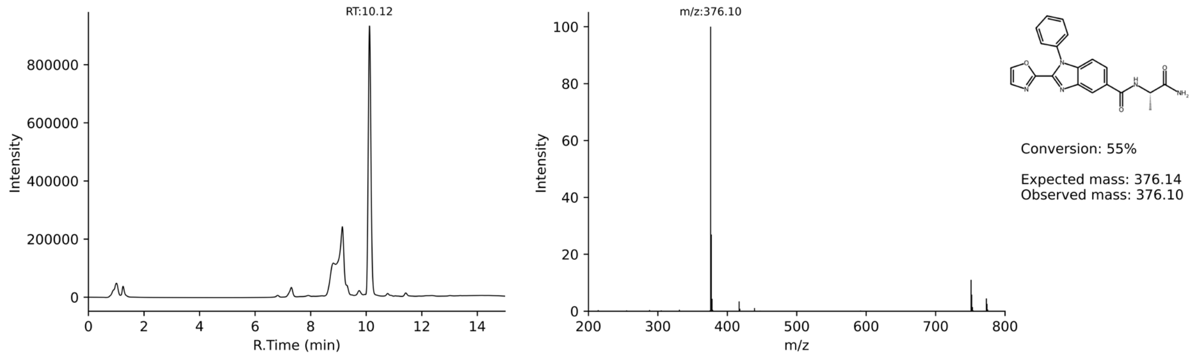

Supplementary Fig 227. LC-MS chromatogram obtained using oxazole-2-carbaldehyde.

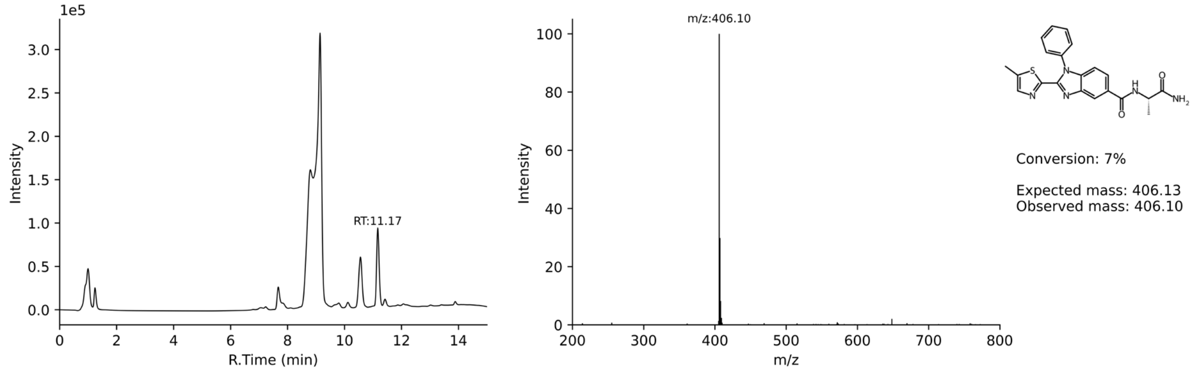

Supplementary Fig 228. LC-MS chromatogram obtained using 5-methylthiazole-2-carbaldehyde.

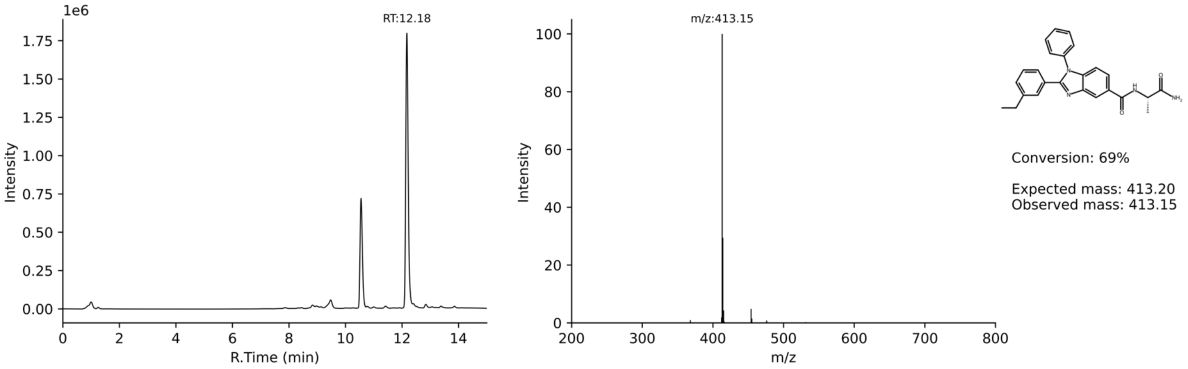

Supplementary Fig 229. LC-MS chromatogram obtained using 3-ethylbenzaldehyde.

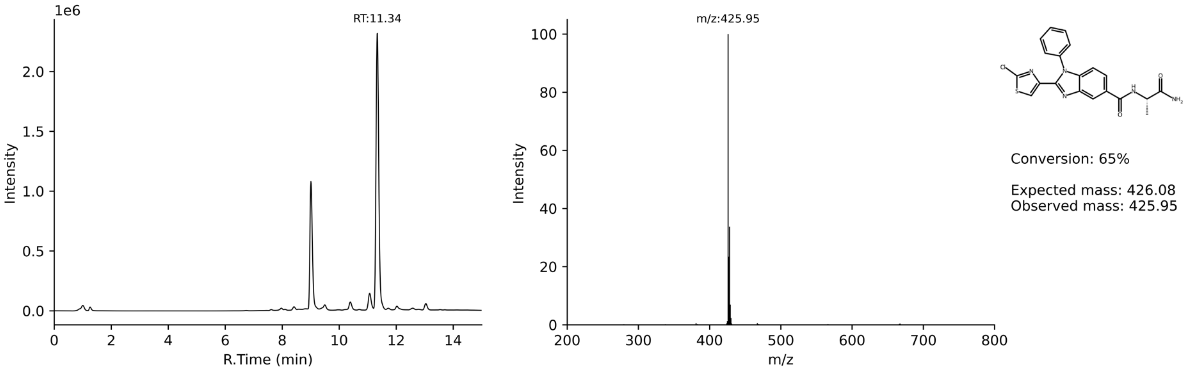

Supplementary Fig 230. LC-MS chromatogram obtained using 2-chlorothiazole-4-carbaldehyde.

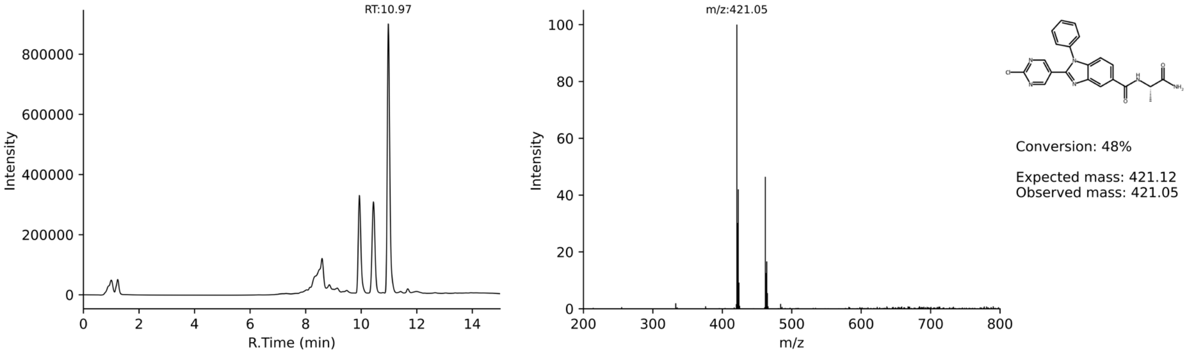

Supplementary Fig 231. LC-MS chromatogram obtained using 2-chloropyrimidine-5-carbaldehyde.

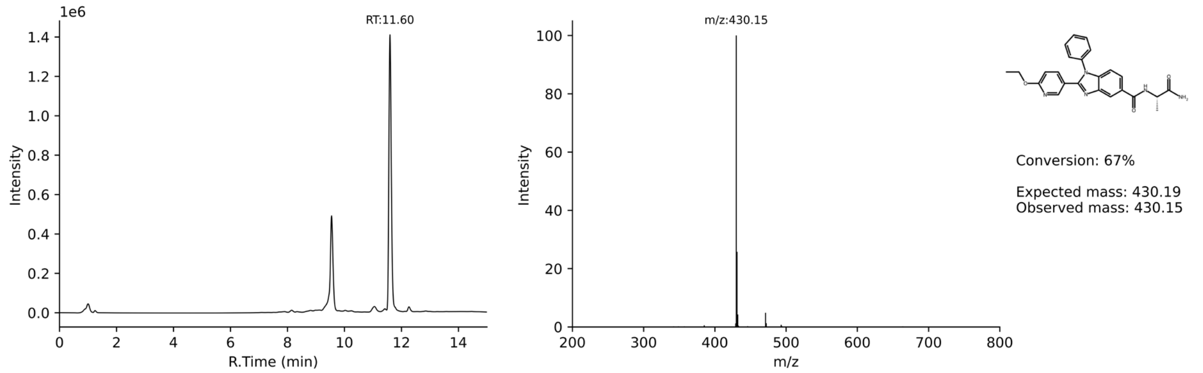

Supplementary Fig 232. LC-MS chromatogram obtained using 6-ethoxynicotinaldehyde.

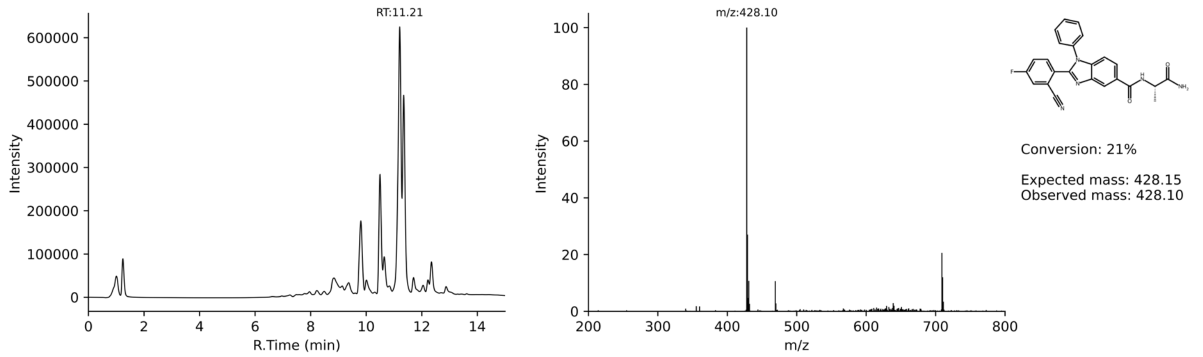

Supplementary Fig 233. LC-MS chromatogram obtained using 5-fluoro-2-formylbenzonitrile.

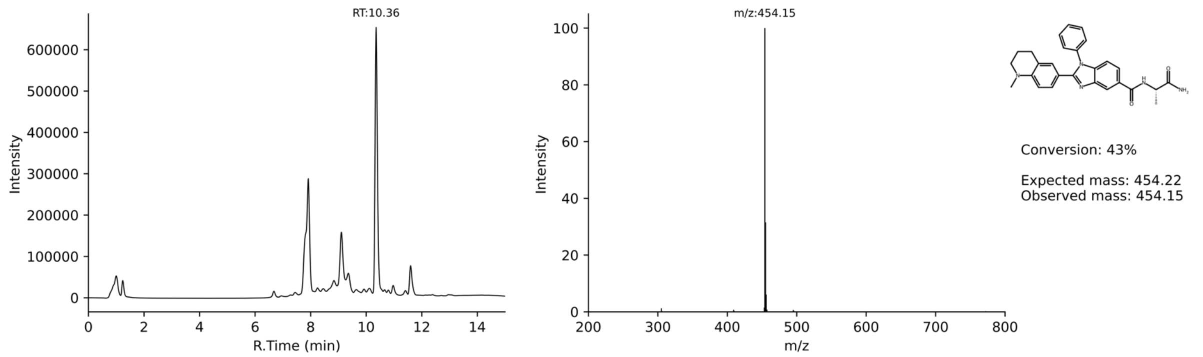

Supplementary Fig 234. LC-MS chromatogram obtained using 1-methyl-1,2,3,4-tetrahydroquinoline-6-carbaldehyde.

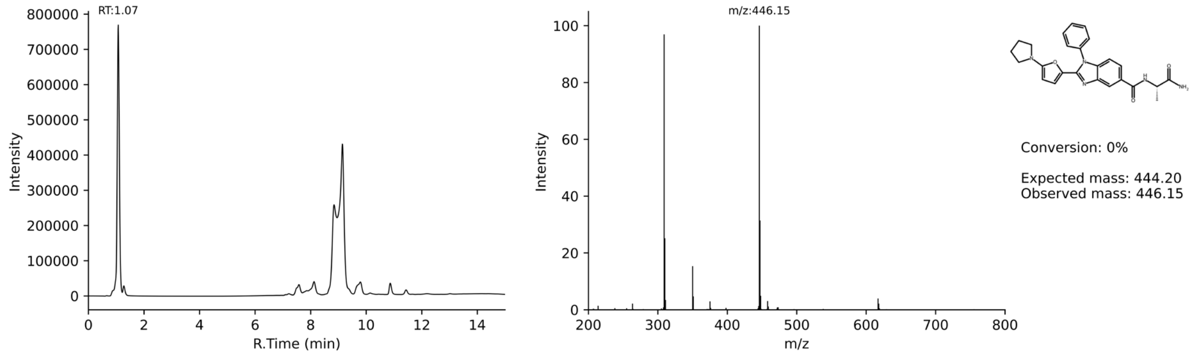

Supplementary Fig 235. LC-MS chromatogram obtained using 5-(pyrrolidin-1-yl)furan-2-carbaldehyde.

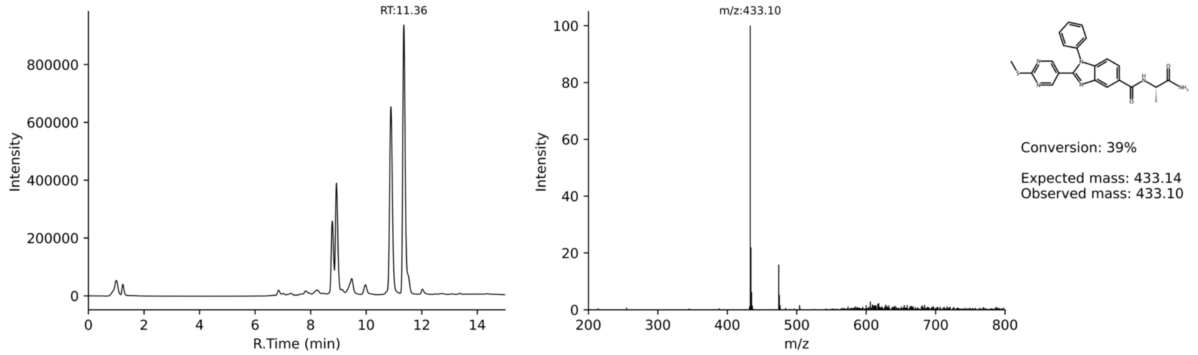

Supplementary Fig 236. LC-MS chromatogram obtained using 2-(methylthio)pyrimidine-5-carbaldehyde.

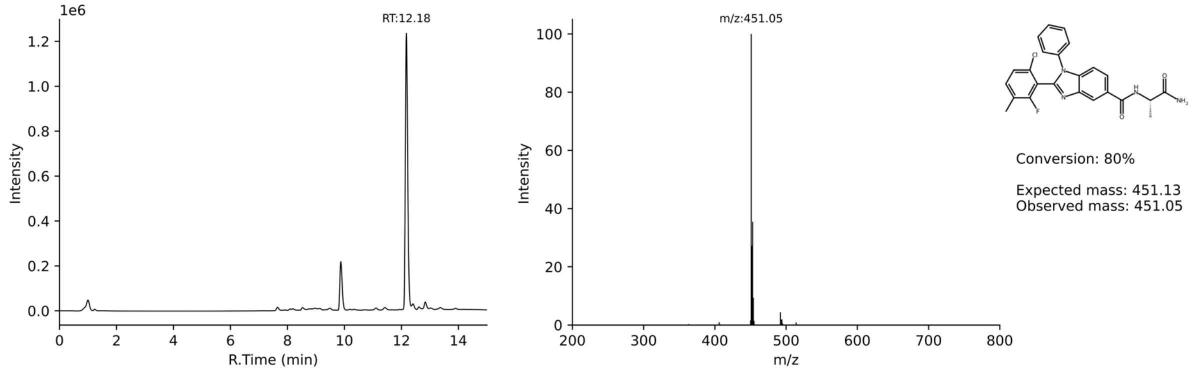

Supplementary Fig 237. LC-MS chromatogram obtained using 6-chloro-2-fluoro-3-methylbenzaldehyde.

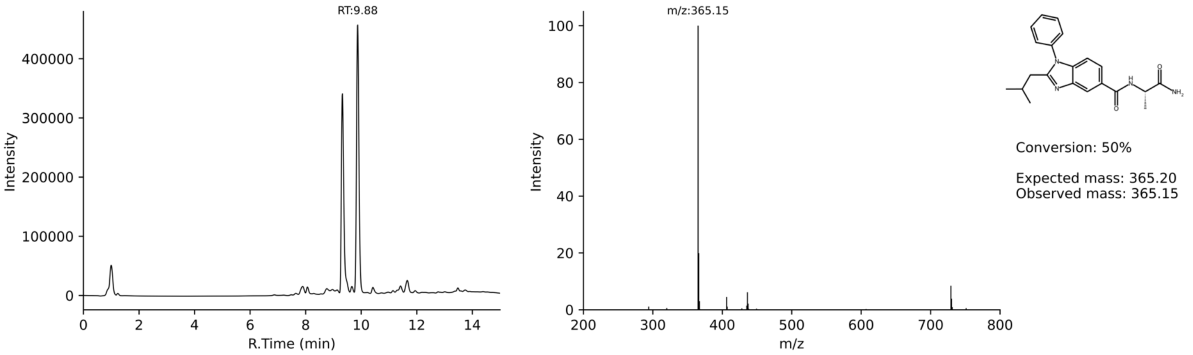

Supplementary Fig 238. LC-MS chromatogram obtained using 3-methylbutanal.

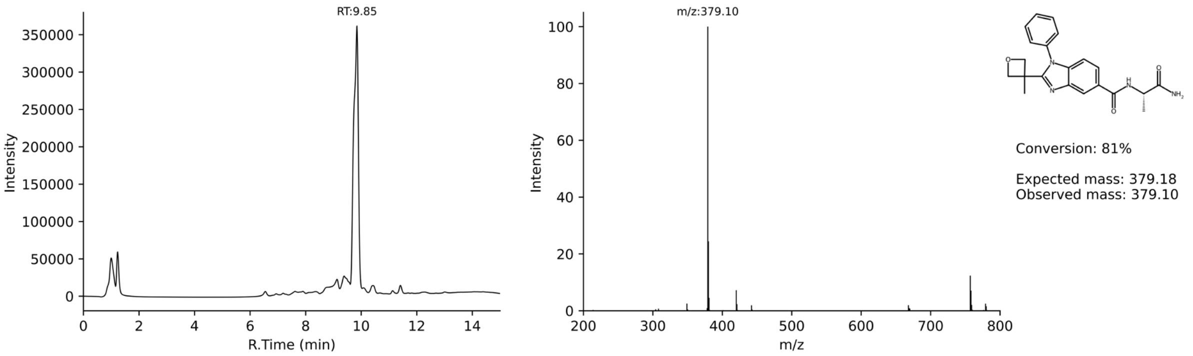

Supplementary Fig 239. LC-MS chromatogram obtained using 3-methyloxetane-3-carbaldehyde.

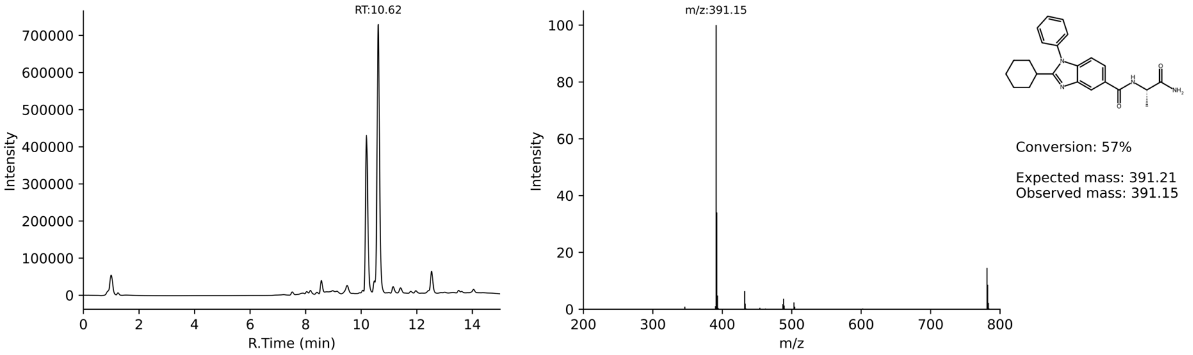

Supplementary Fig 240. LC-MS chromatogram obtained using cyclohexanecarbaldehyde.

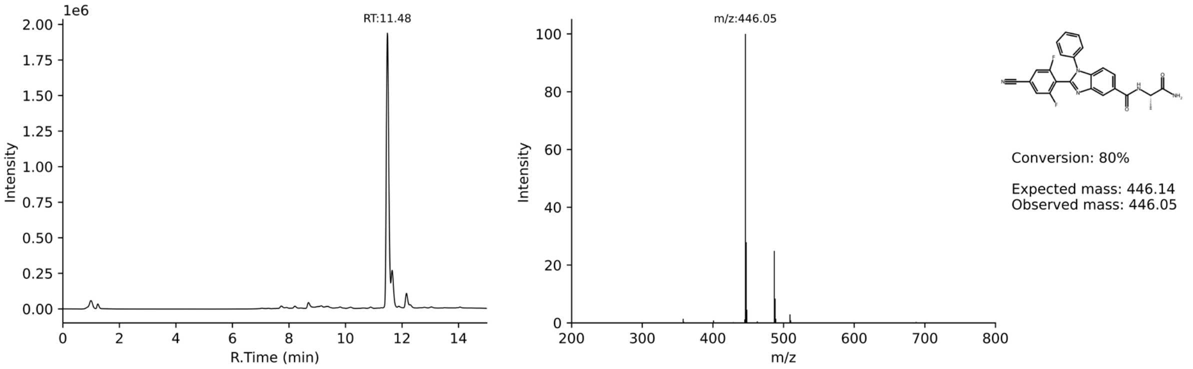

Supplementary Fig 241. LC-MS chromatogram obtained using 3,5-difluoro-4-formylbenzonitrile.

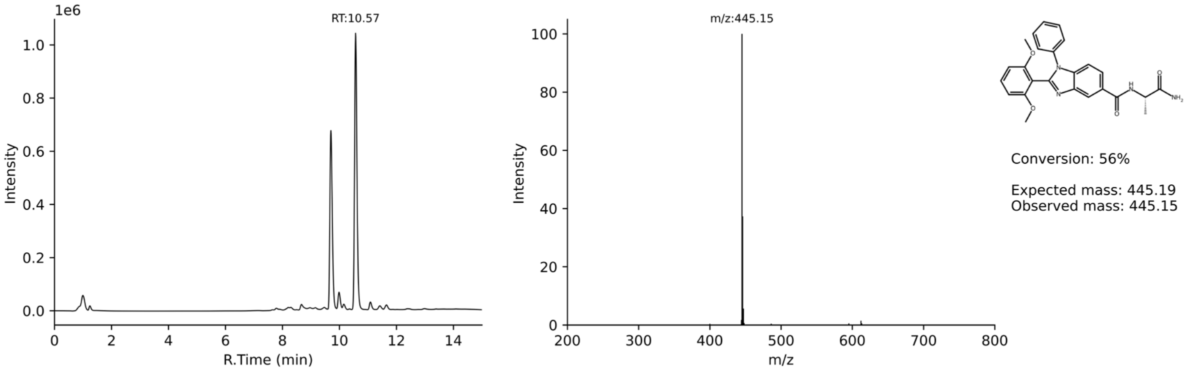

Supplementary Fig 242. LC-MS chromatogram obtained using 2,6-dimethoxybenzaldehyde.

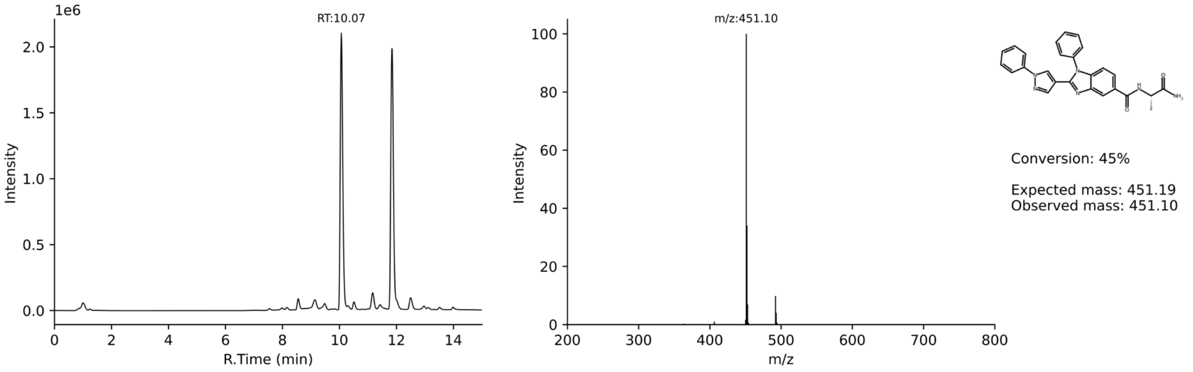

Supplementary Fig 243. LC-MS chromatogram obtained using 1-phenyl-1h-pyrazole-4-carbaldehyde.

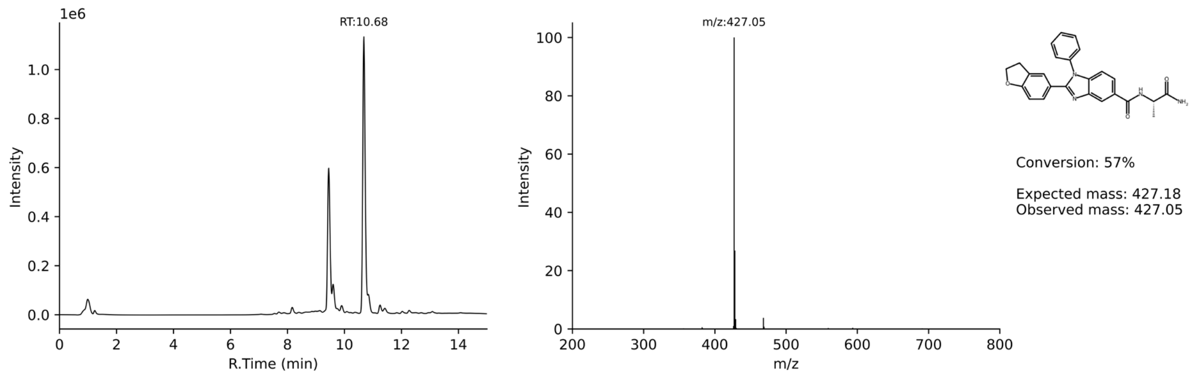

Supplementary Fig 244. LC-MS chromatogram obtained using 2,3-dihydrobenzofuran-5-carbaldehyde.

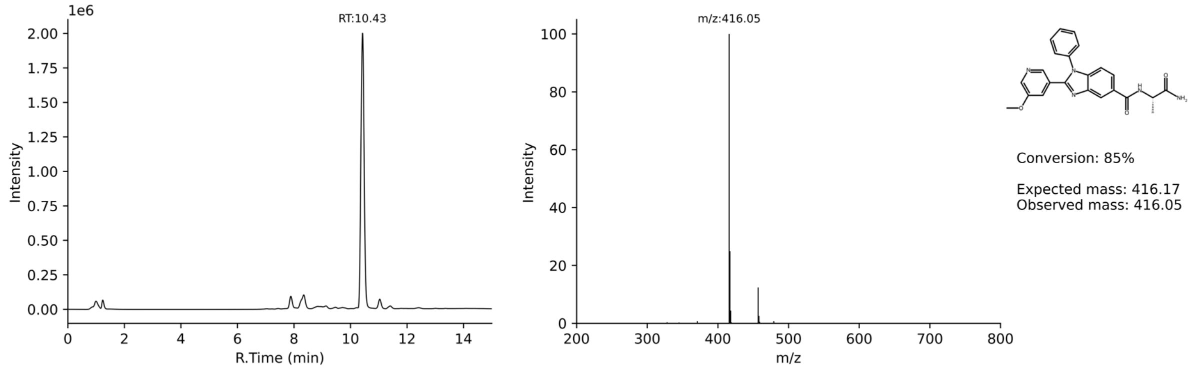

Supplementary Fig 245. LC-MS chromatogram obtained using 5-methoxynicotinaldehyde.

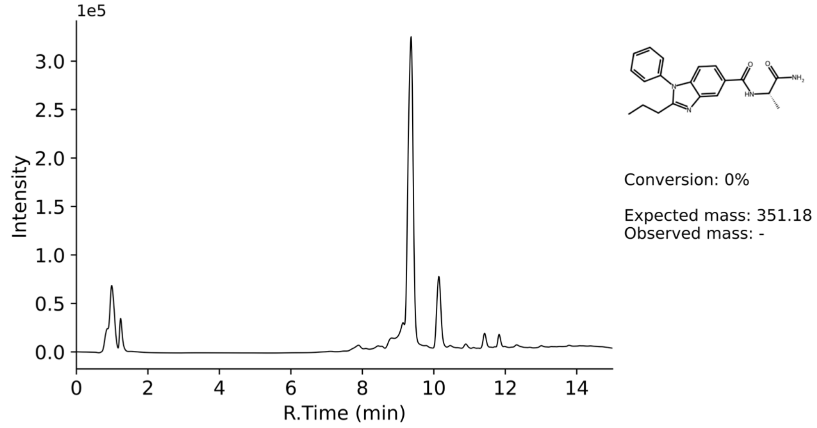

Supplementary Fig 246. LC-MS chromatogram obtained using butyraldehyde.

12.3 LC-MS data from the scope of aryl bromides

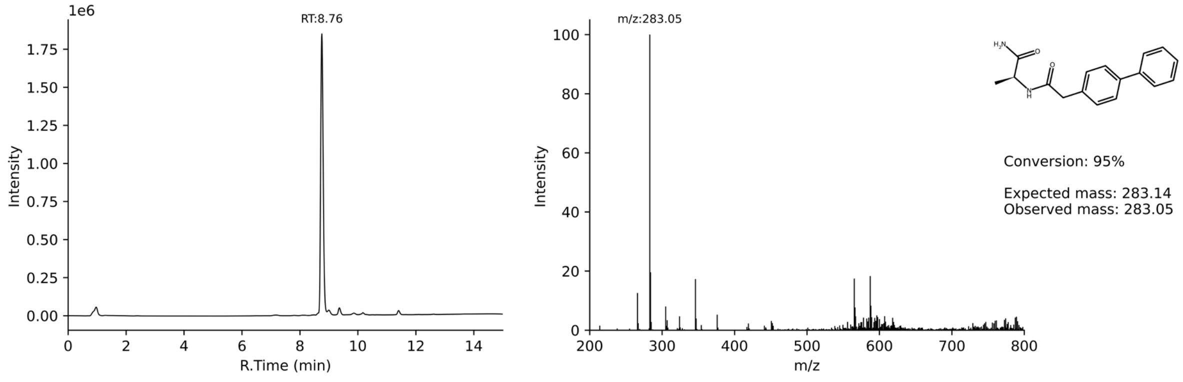

Supplementary Fig 247. LC-MS chromatogram obtained using 4-bromo phenyl acetic acid.

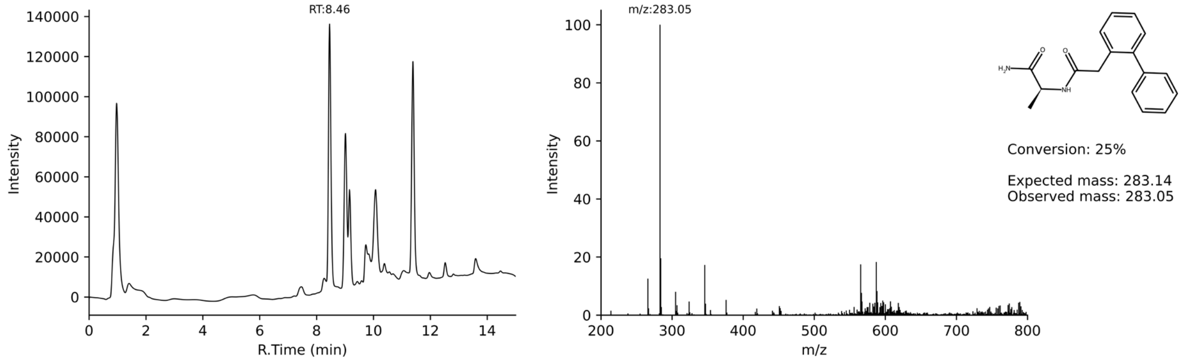

Supplementary Fig 248. LC-MS chromatogram obtained using 2-Bromophenylacetic acid.

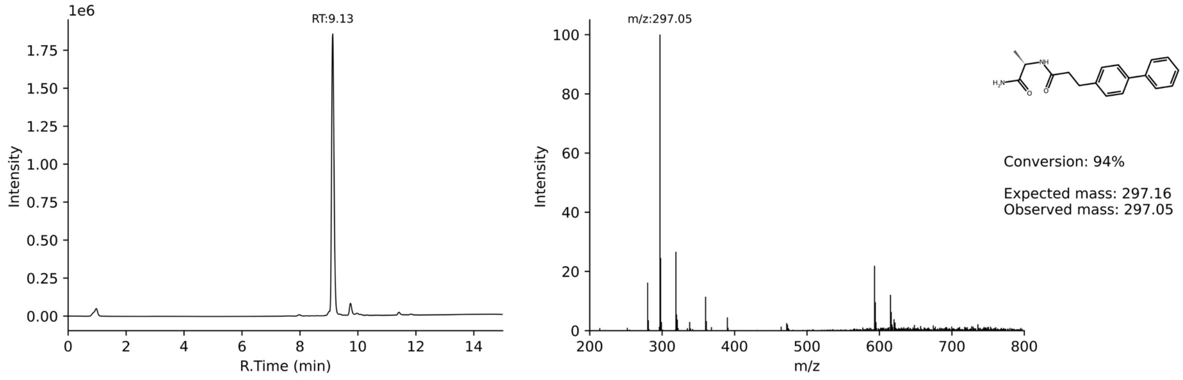

Supplementary Fig 249. LC-MS chromatogram obtained using 3-(4-bromophenyl)propanoic acid.

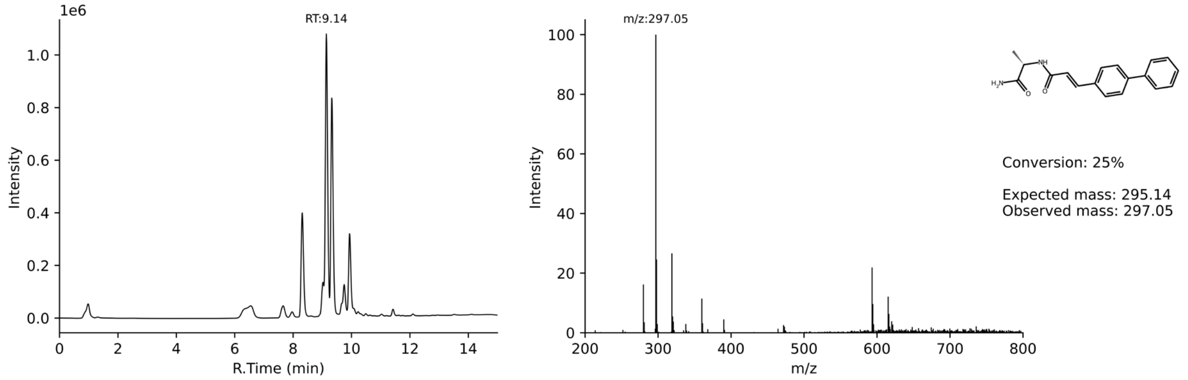

Supplementary Fig 250. LC-MS chromatogram obtained using 4-Bromocinnamic acid.

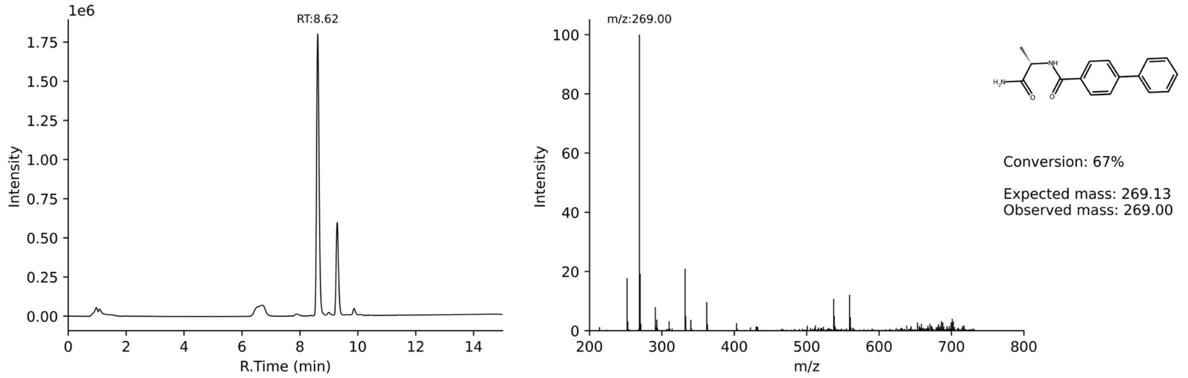

Supplementary Fig 251. LC-MS chromatogram obtained using 4-bromobenzoic acid.

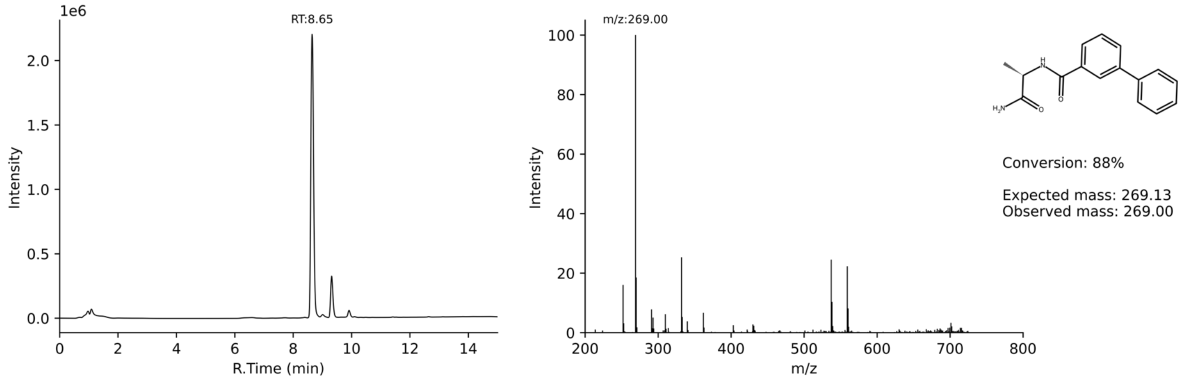

Supplementary Fig 252. LC-MS chromatogram obtained using 3-bromobenzoic acid.

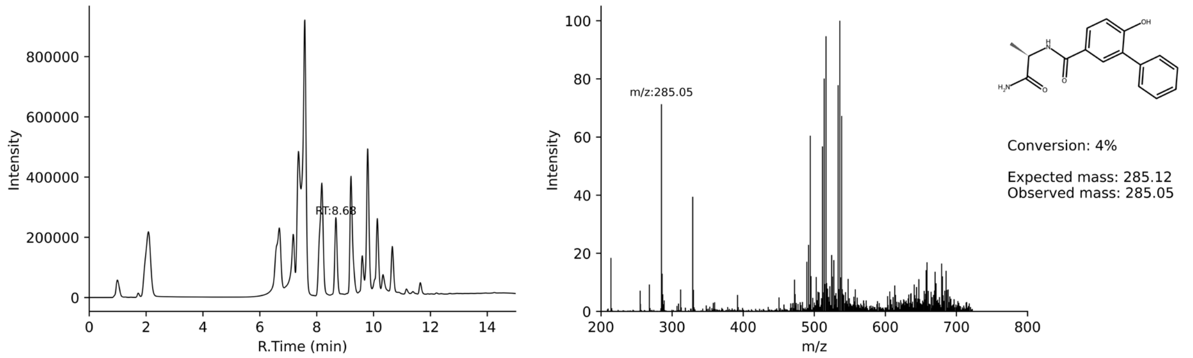

Supplementary Fig 253. LC-MS chromatogram obtained using 3-bromo-4-hydroxybenzoic acid.

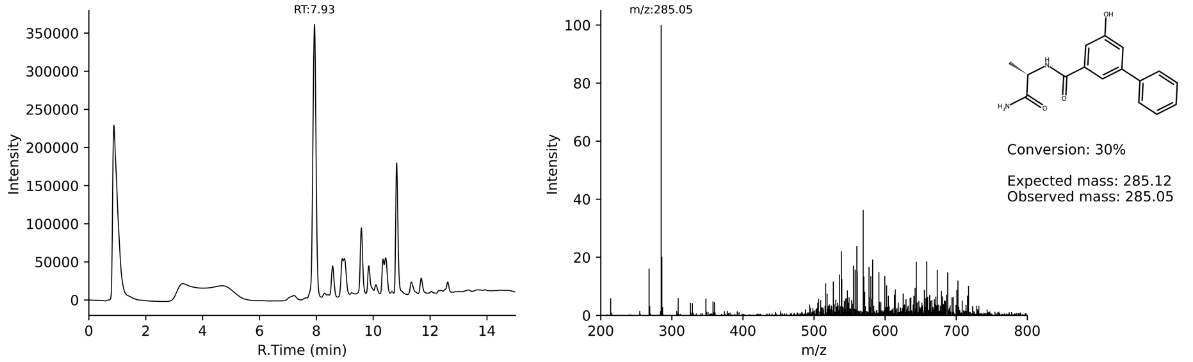

Supplementary Fig 254. LC-MS chromatogram obtained using 3-bromo-5-hydroxybenzoic acid.

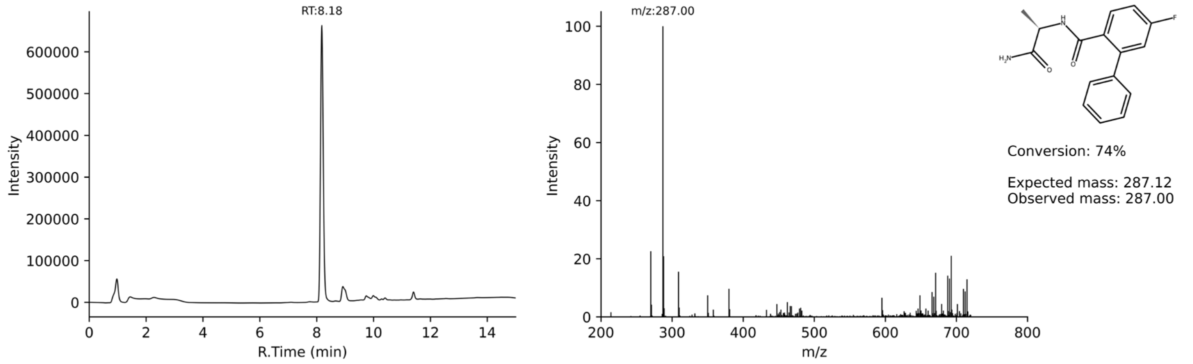

Supplementary Fig 255. LC-MS chromatogram obtained using 2-bromo-4-fluorobenzoic acid.

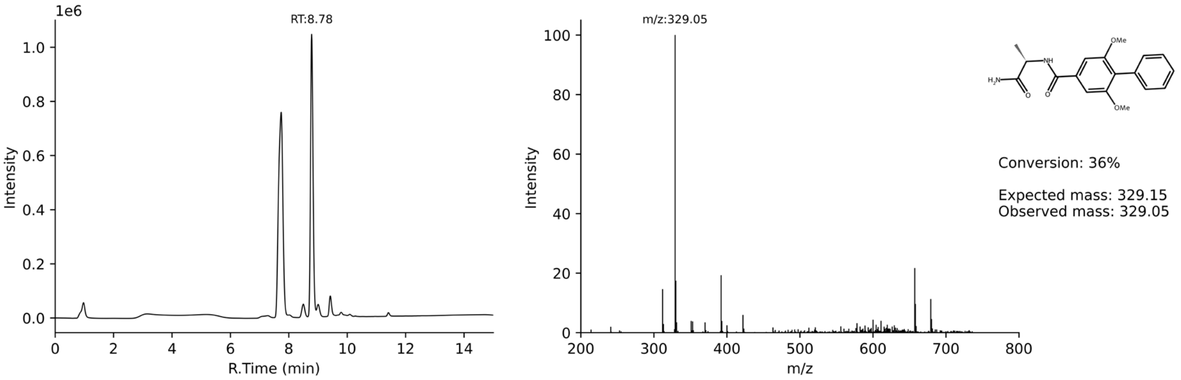

Supplementary Fig 256. LC-MS chromatogram obtained using 4-bromo-3,5-dimethoxybenzoic acid.

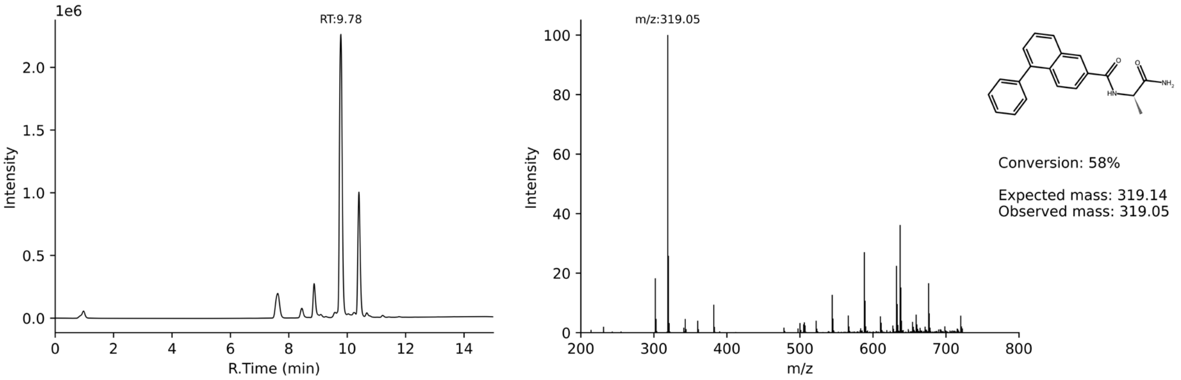

Supplementary Fig 257. LC-MS chromatogram obtained using 5-bromo-2-naphtoic acid.

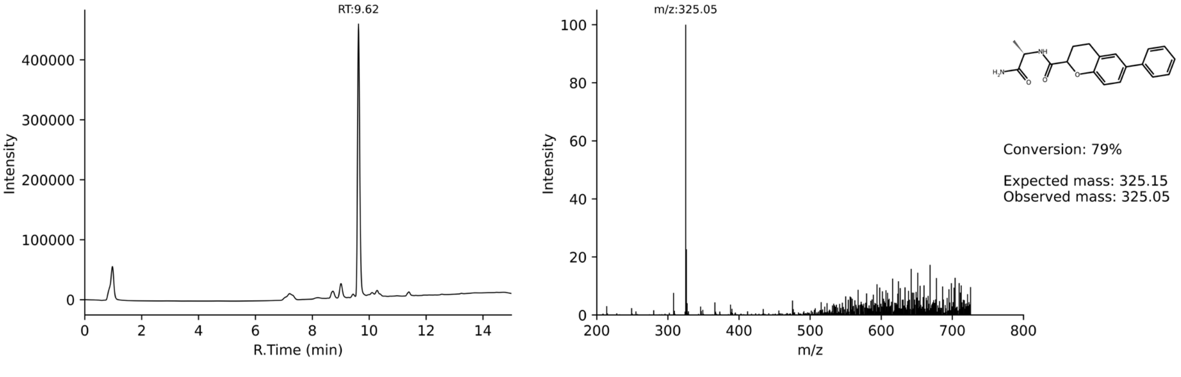

Supplementary Fig 258. LC-MS chromatogram obtained using 6-bromochroman-2-carboxylic acid.

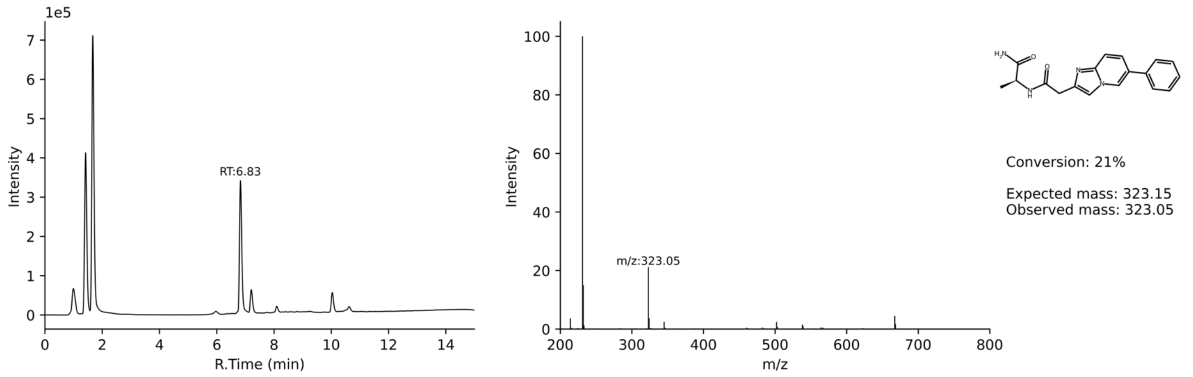

Supplementary Fig 259. LC-MS chromatogram obtained using 2-(6-Bromoimidazo[1,2-a]pyridin-2-yl)acetic acid.

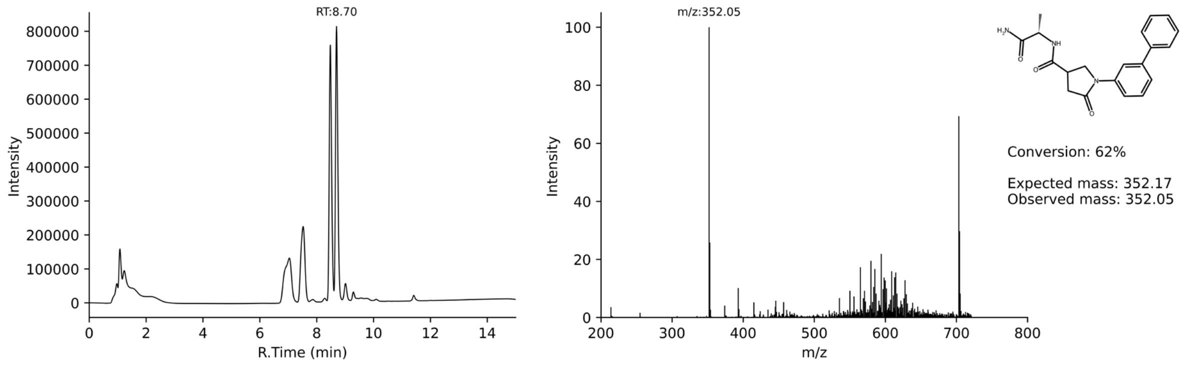

Supplementary Fig 260. LC-MS chromatogram obtained using 1-(3-bromophenyl)-5-oxopyrrolidine-3-carboxylic acid.

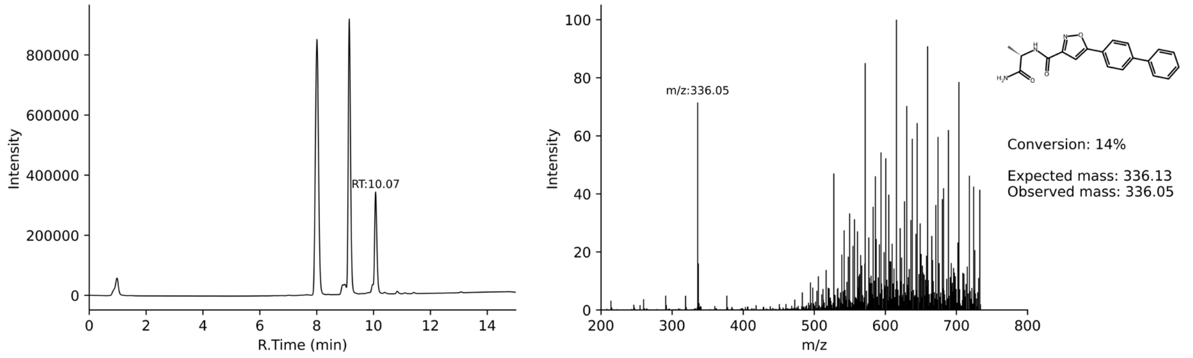

Supplementary Fig 261. LC-MS chromatogram obtained using 5-(4-bromphenyl)isoxazole-3-carboxylic acid.

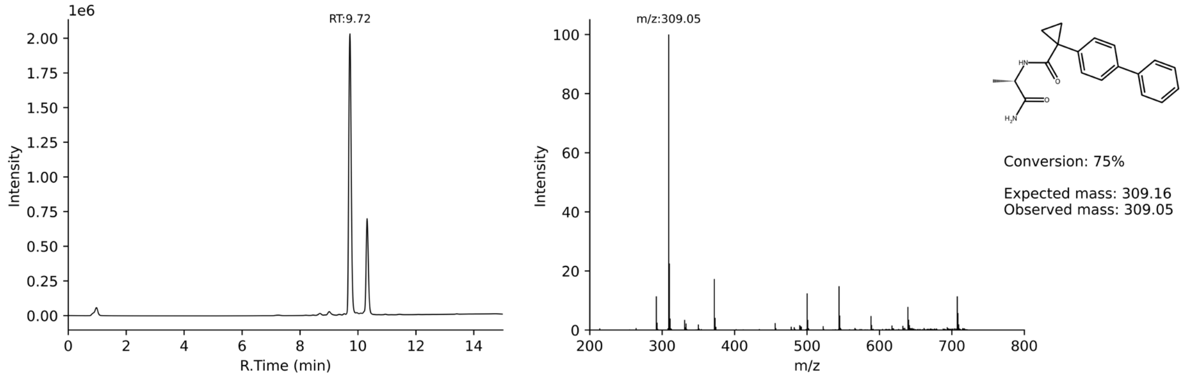

Supplementary Fig 262. LC-MS chromatogram obtained using 1-(4-bromophenyl)-cyclopropane carboxylic acid.

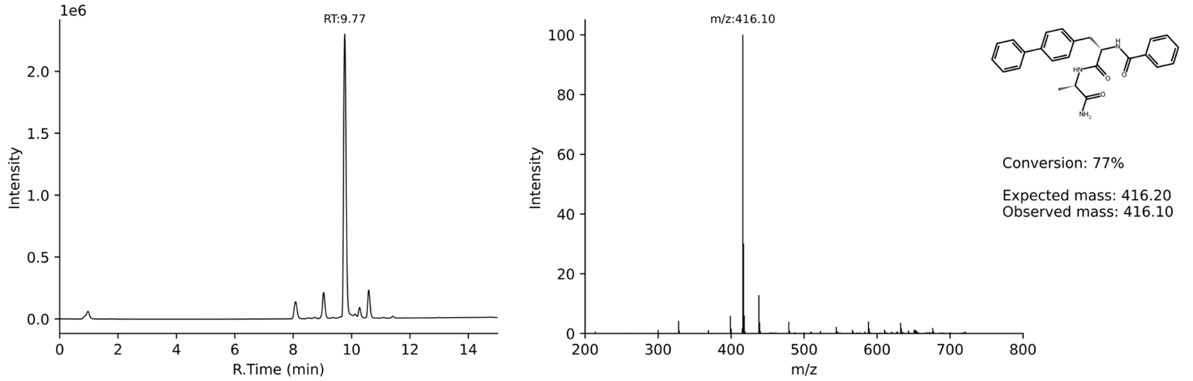

Supplementary Fig 263. LC-MS chromatogram obtained using L-fmoc-phe(4-br)-oh.

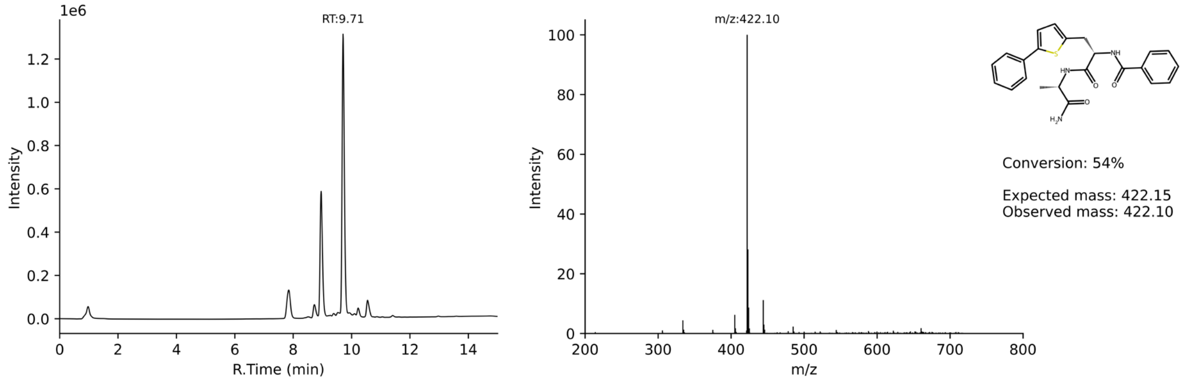

Supplementary Fig 264. LC-MS chromatogram obtained using Fmoc-(5-bromo-1H-indol-3-yl) propanoic acid.

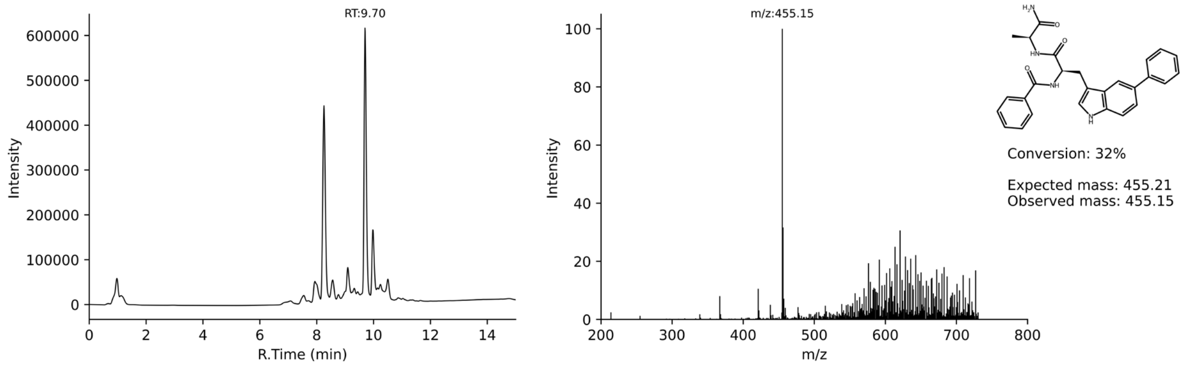

Supplementary Fig 265. LC-MS chromatogram obtained using Fmoc-(5-bromothiophen-2-yl)propanoic acid.

12.4 LC-MS data from the scope of boronic acids

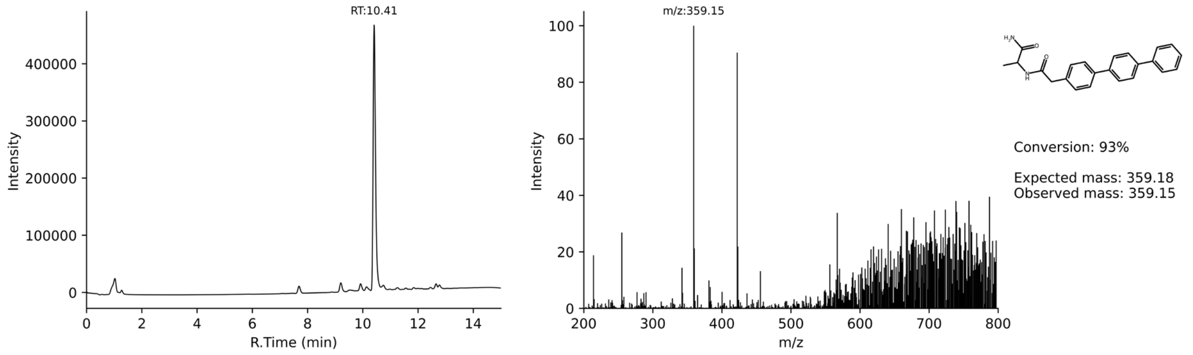

Supplementary Fig 266. LC-MS chromatogram obtained using (4-phenylphenyl)boronic acid.

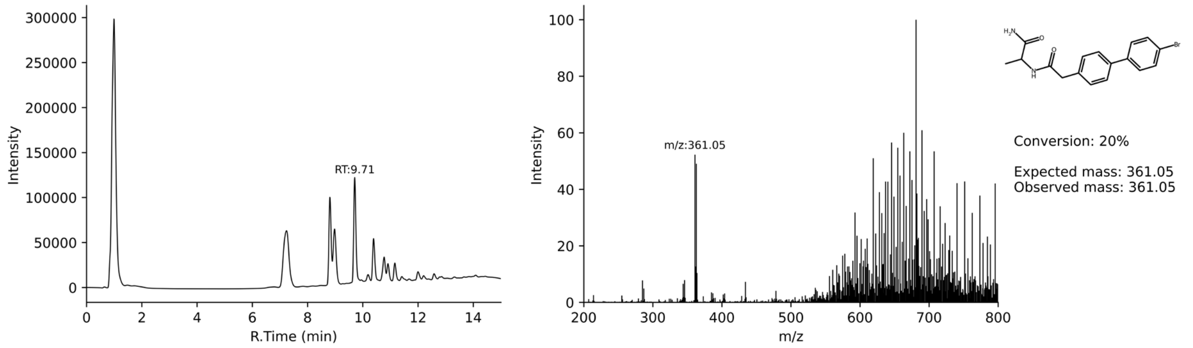

Supplementary Fig 267. LC-MS chromatogram obtained using (4-bromophenyl)boronic acid.

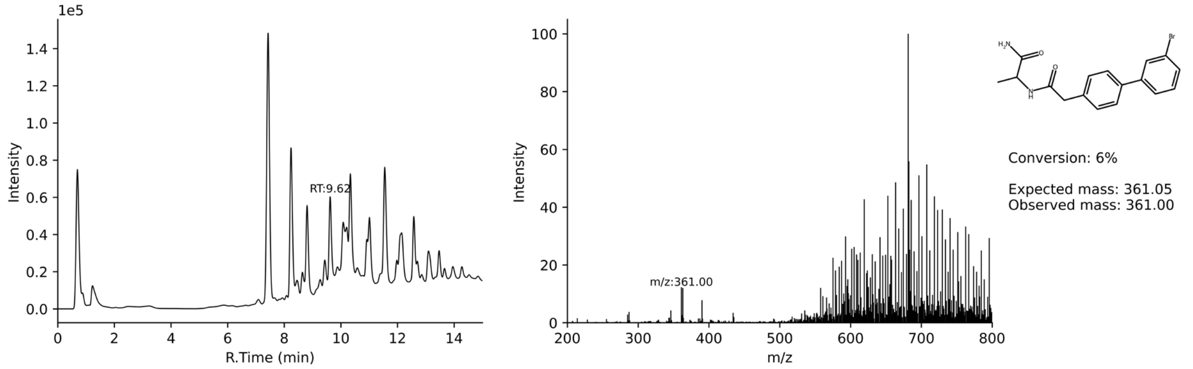

Supplementary Fig 268. LC-MS chromatogram obtained using (3-bromophenyl)boronic acid.

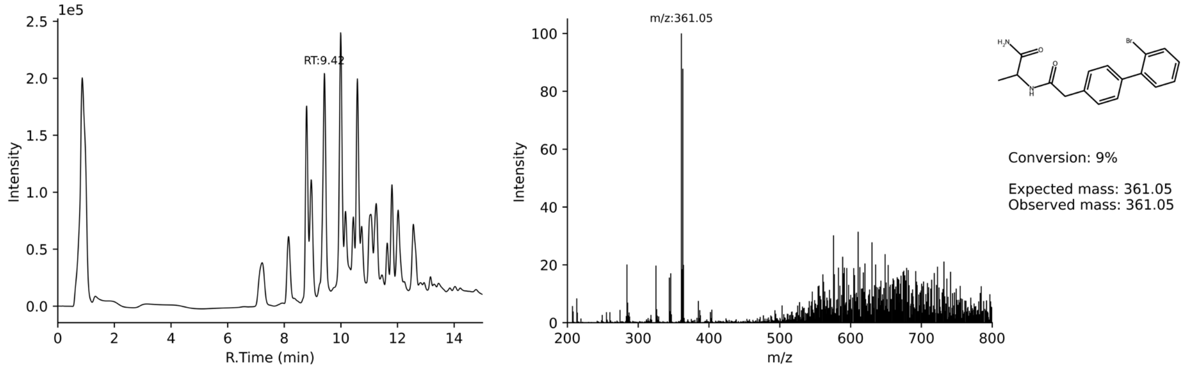

Supplementary Fig 269. LC-MS chromatogram obtained using (2-bromophenyl)boronic acid.

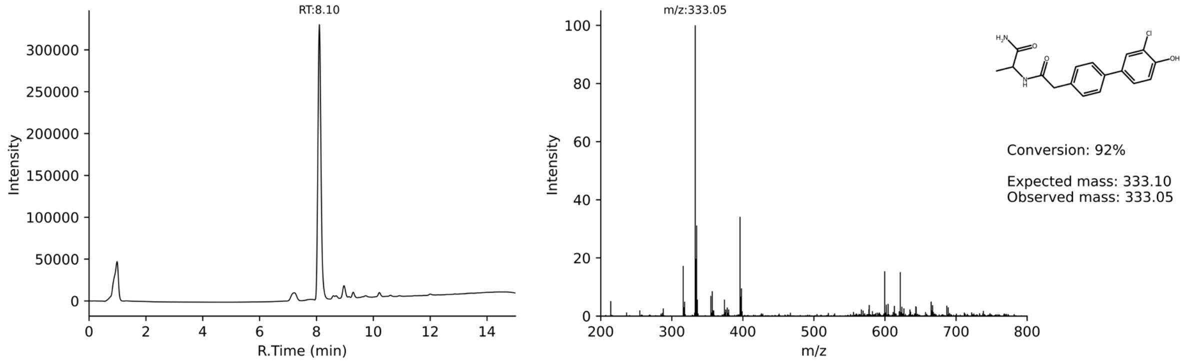

Supplementary Fig 270. LC-MS chromatogram obtained using (3-chloro-4-hydroxyphenyl)boronic acid.

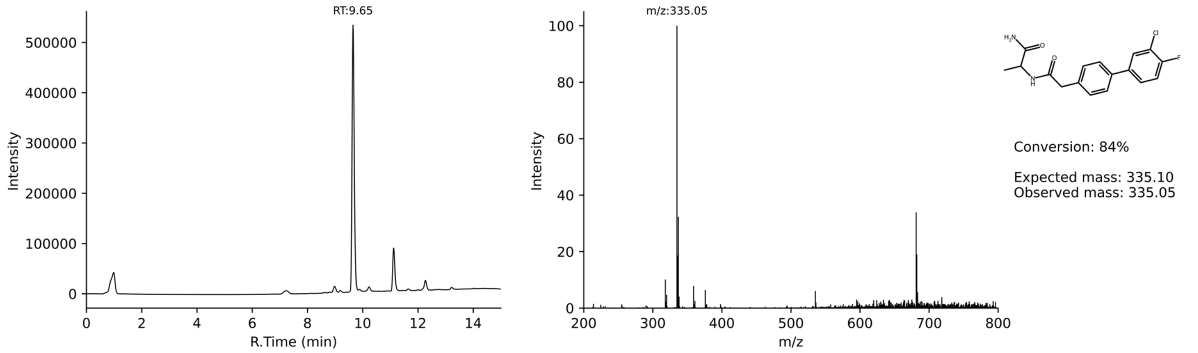

Supplementary Fig 271. LC-MS chromatogram obtained using (3-chloro-4-fluorophenyl)boronic acid.

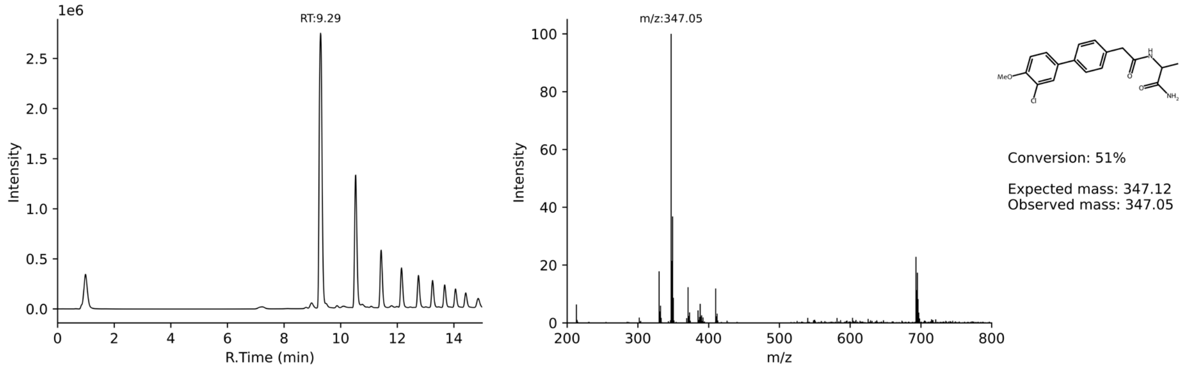

Supplementary Fig 272. LC-MS chromatogram obtained using (3-chloro-4-methoxyphenyl)boronic acid.

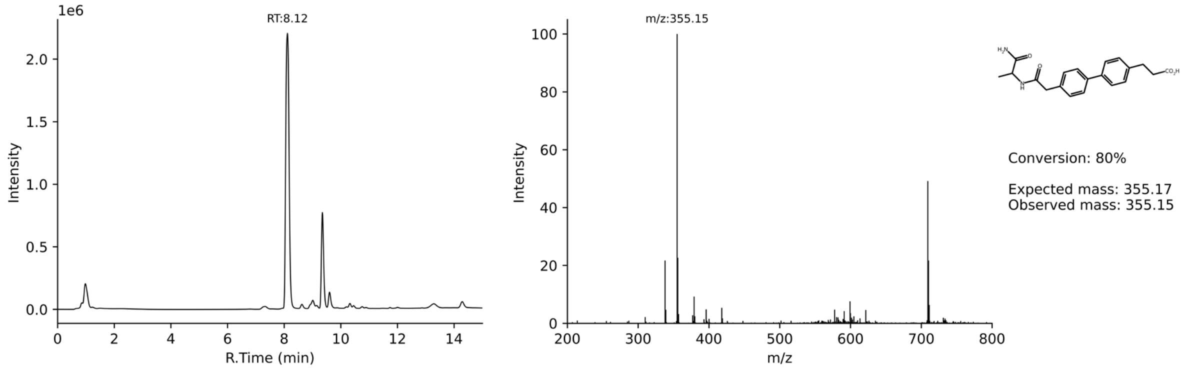

Supplementary Fig 273. LC-MS chromatogram obtained using 3-(4-boronophenyl)propanoic acid.

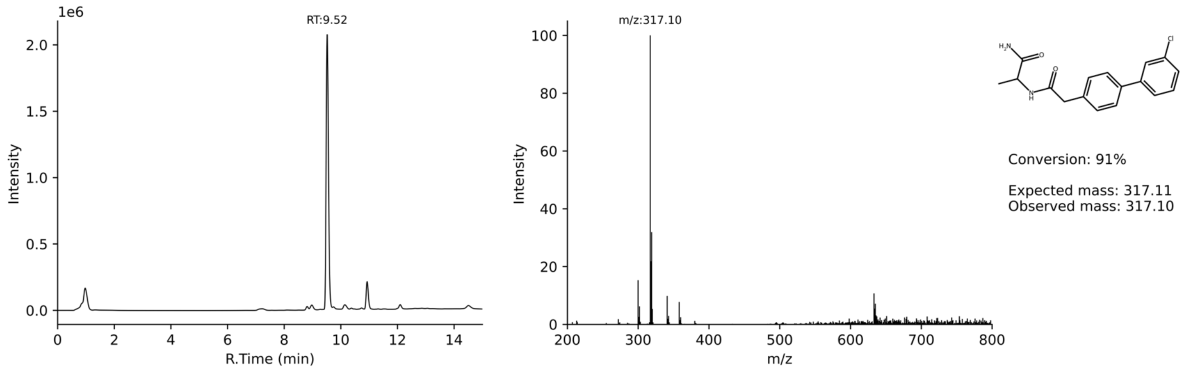

Supplementary Fig 274. LC-MS chromatogram obtained using (3-chlorophenyl)boronic acid.

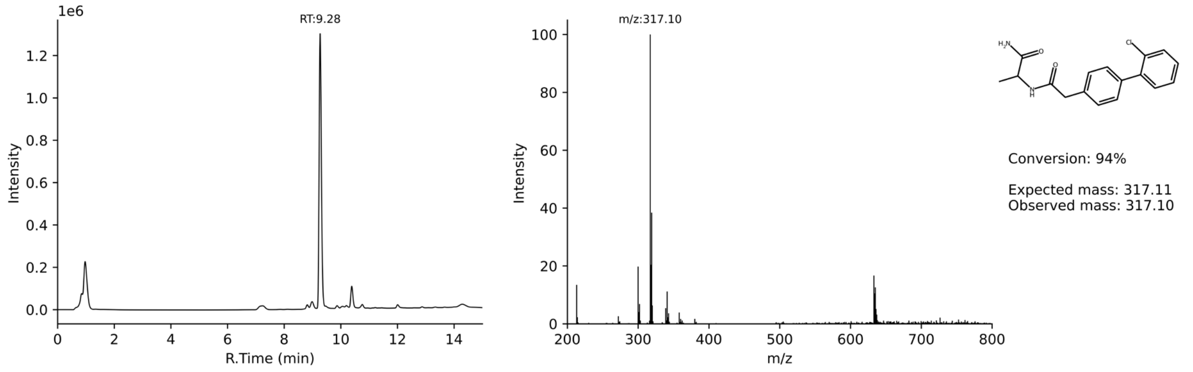

Supplementary Fig 275. LC-MS chromatogram obtained using (2-chlorophenyl)boronic acid.

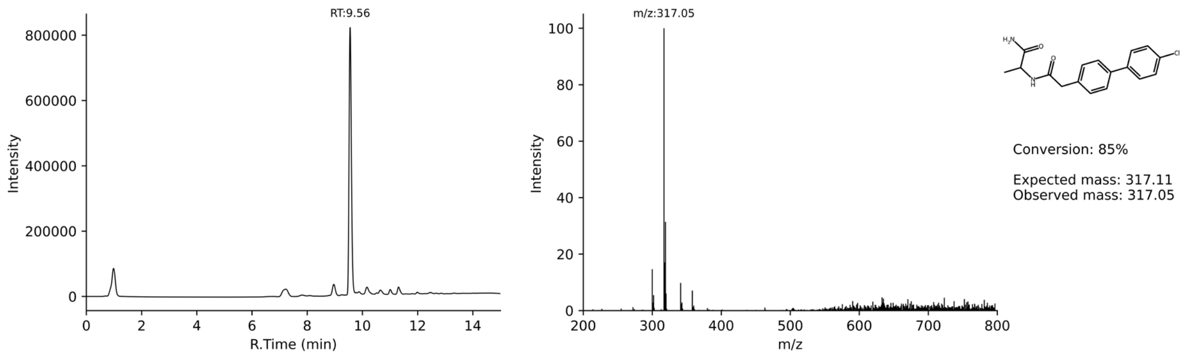

Supplementary Fig 276. LC-MS chromatogram obtained using (4-chlorophenyl)boronic acid.

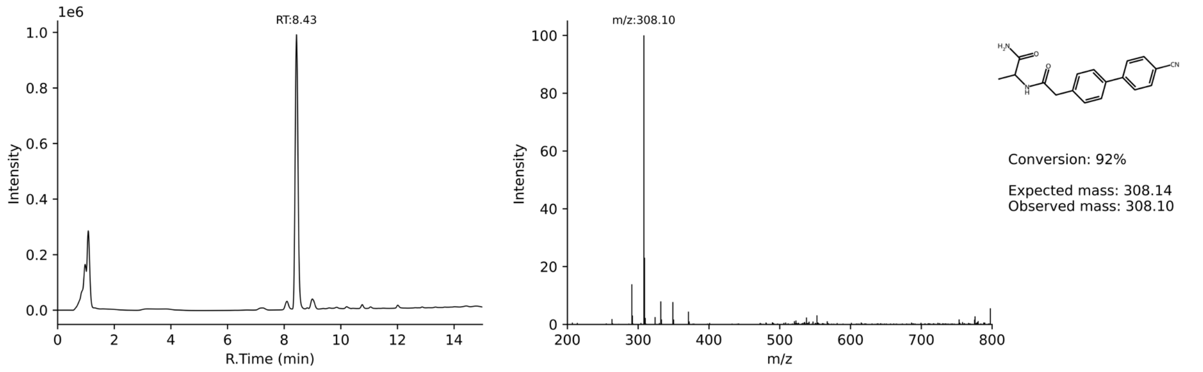

Supplementary Fig 277. LC-MS chromatogram obtained using (4-cyanophenyl)boronic acid.

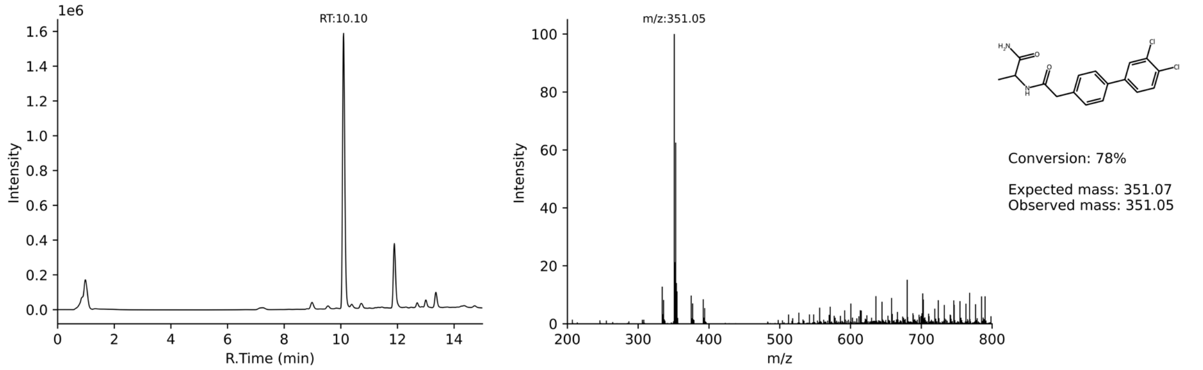

Supplementary Fig 278. LC-MS chromatogram obtained using (3,4-dichlorophenyl)boronic acid.

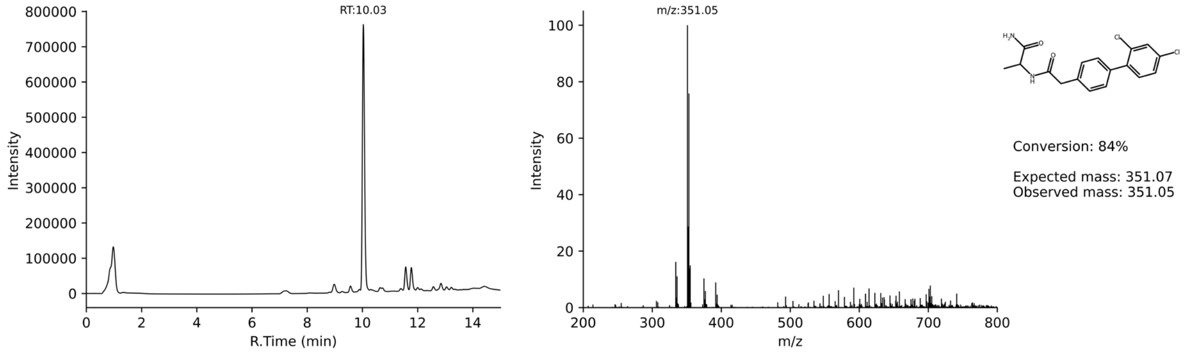

Supplementary Fig 279. LC-MS chromatogram obtained using (2,4-dichlorophenyl)boronic acid.

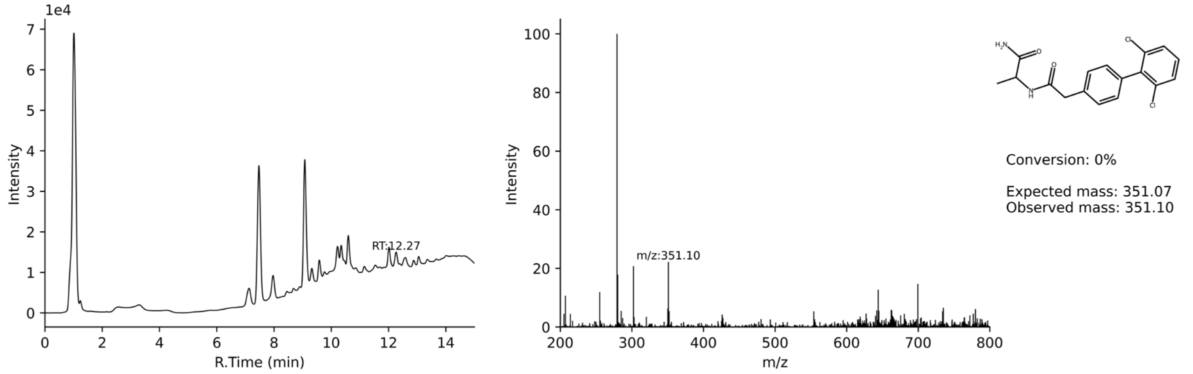

Supplementary Fig 280. LC-MS chromatogram obtained using (2,6-dichlorophenyl)boronic acid.

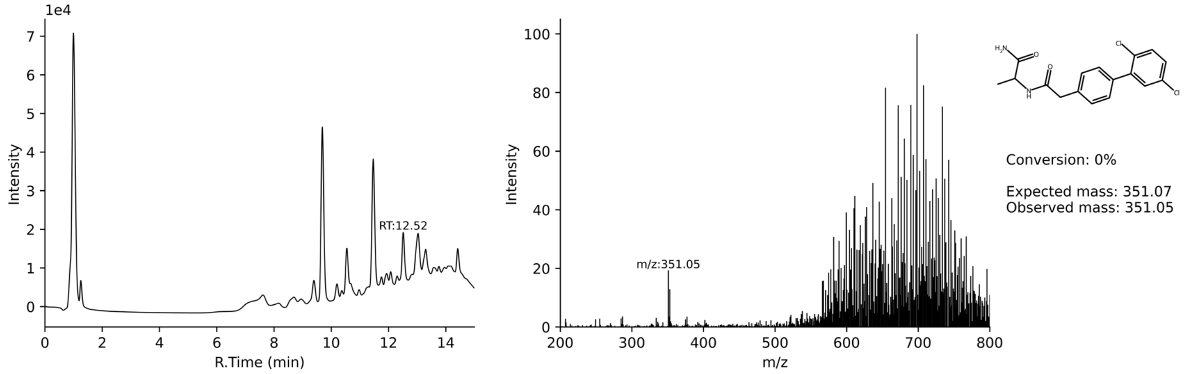

Supplementary Fig 281. LC-MS chromatogram obtained using (2,5-dichlorophenyl)boronic acid.

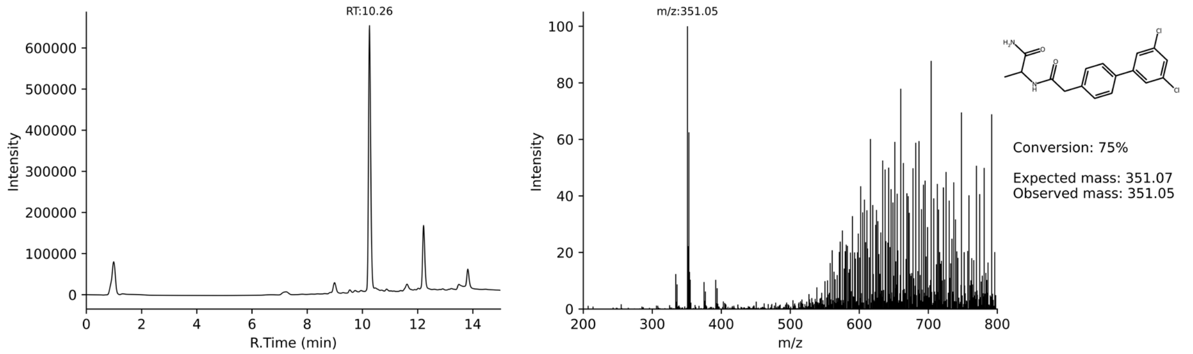

Supplementary Fig 282. LC-MS chromatogram obtained using (3,5-dichlorophenyl)boronic acid.

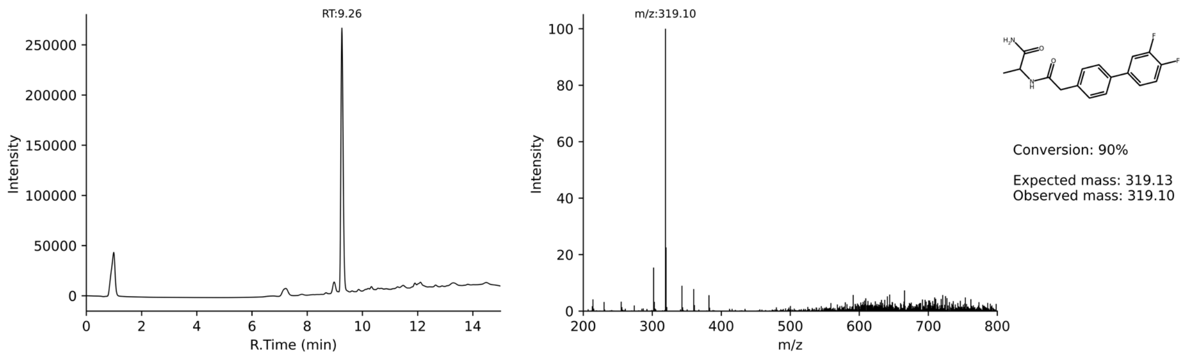

Supplementary Fig 283. LC-MS chromatogram obtained using (3,4-difluorophenyl)boronic acid.

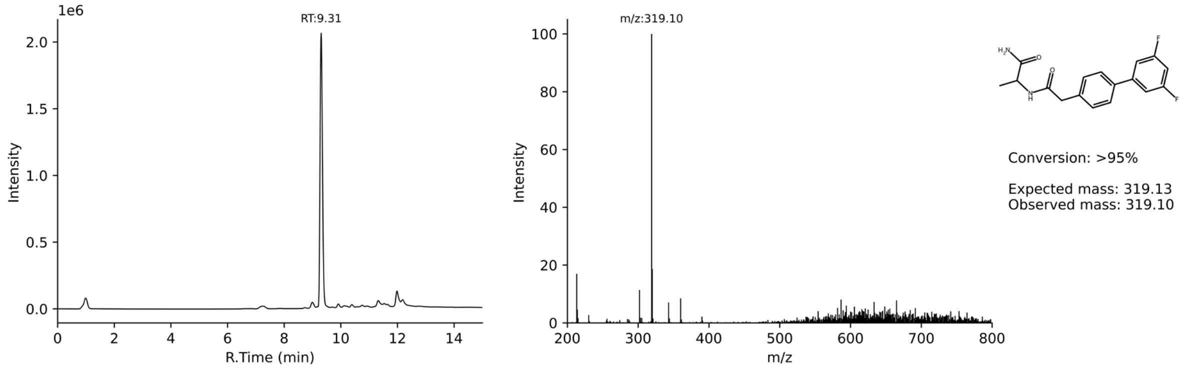

Supplementary Fig 284. LC-MS chromatogram obtained using (3,5-difluorophenyl)boronic acid.

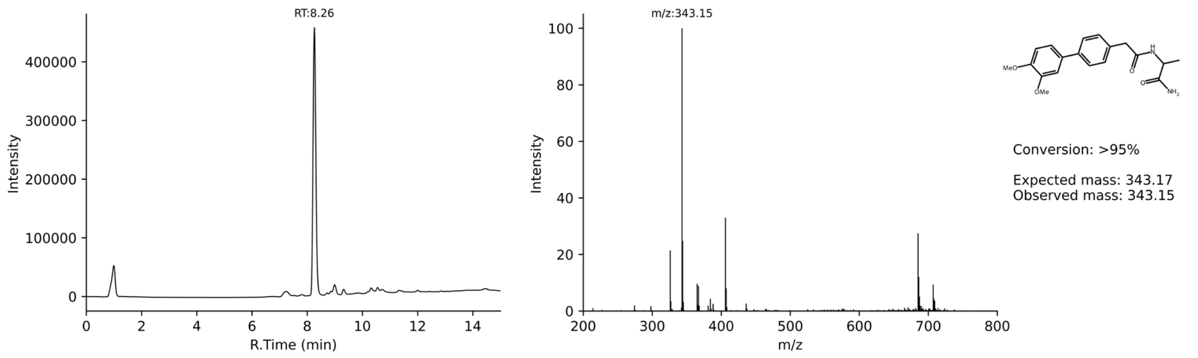

Supplementary Fig 285. LC-MS chromatogram obtained using (3,4-dimethoxyphenyl)boronic acid.

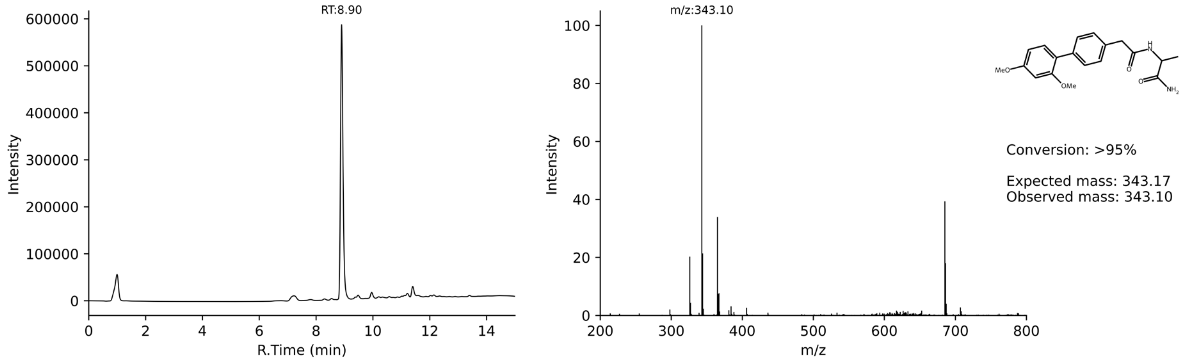

Supplementary Fig 286. LC-MS chromatogram obtained using (2,4-dimethoxyphenyl)boronic acid.

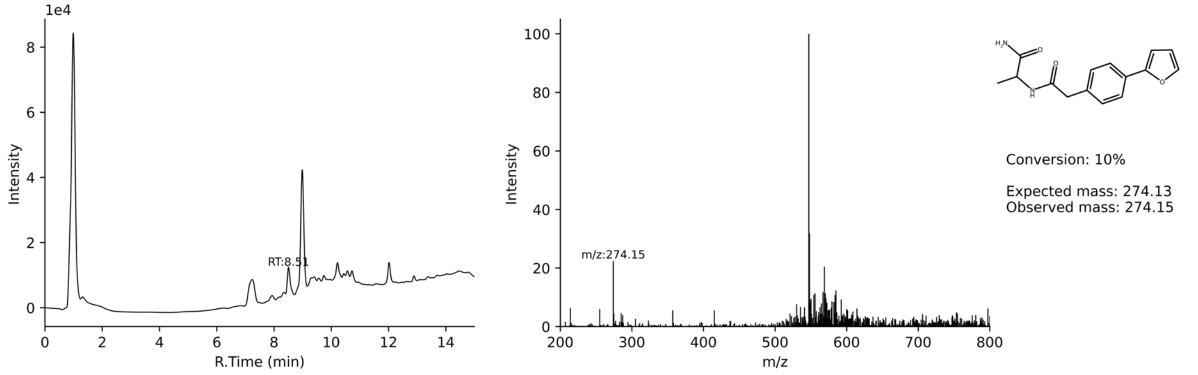

Supplementary Fig 287. LC-MS chromatogram obtained using furan-2-ylboronic acid.

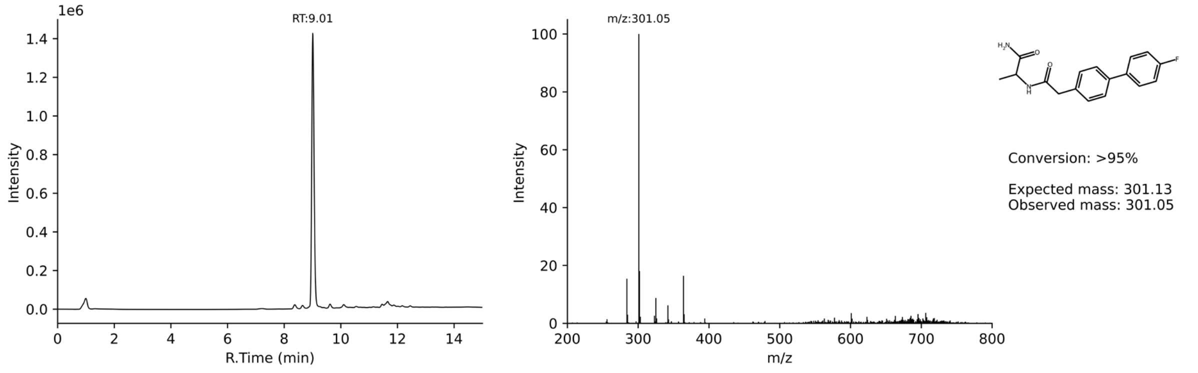

Supplementary Fig 288. LC-MS chromatogram obtained using (4-fluorophenyl)boronic acid.

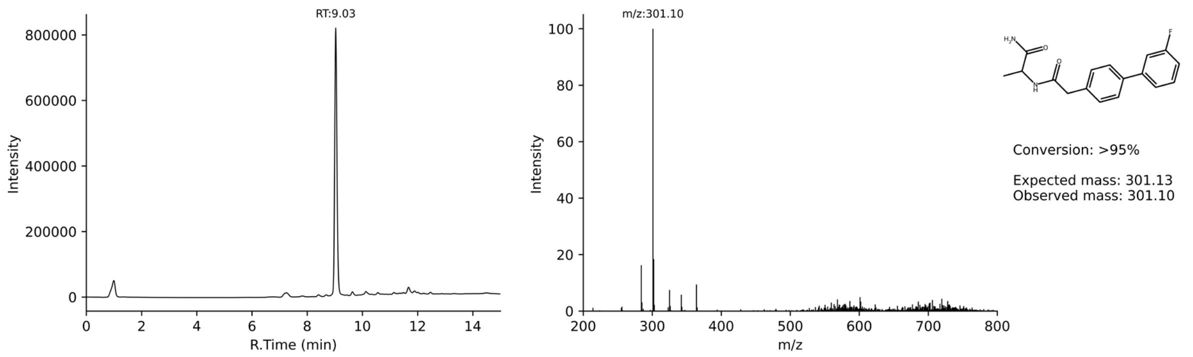

Supplementary Fig 289. LC-MS chromatogram obtained using (3-fluorophenyl)boronic acid.

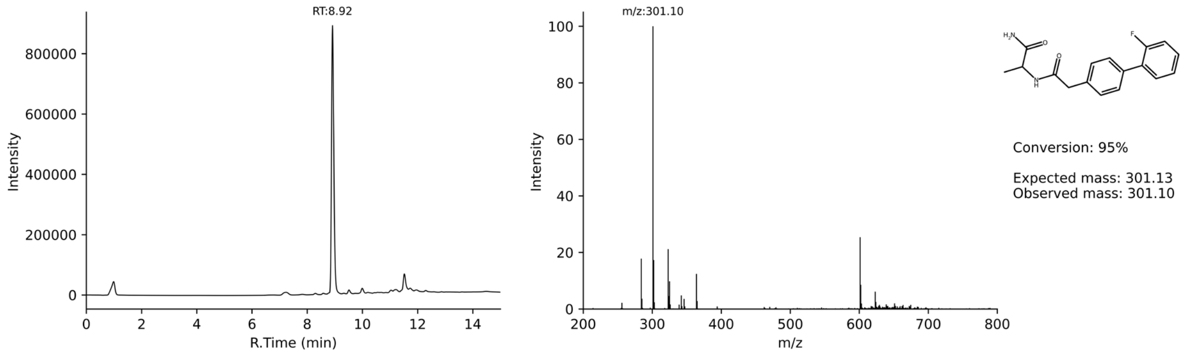

Supplementary Fig 290. LC-MS chromatogram obtained using (2-fluorophenyl)boronic acid.

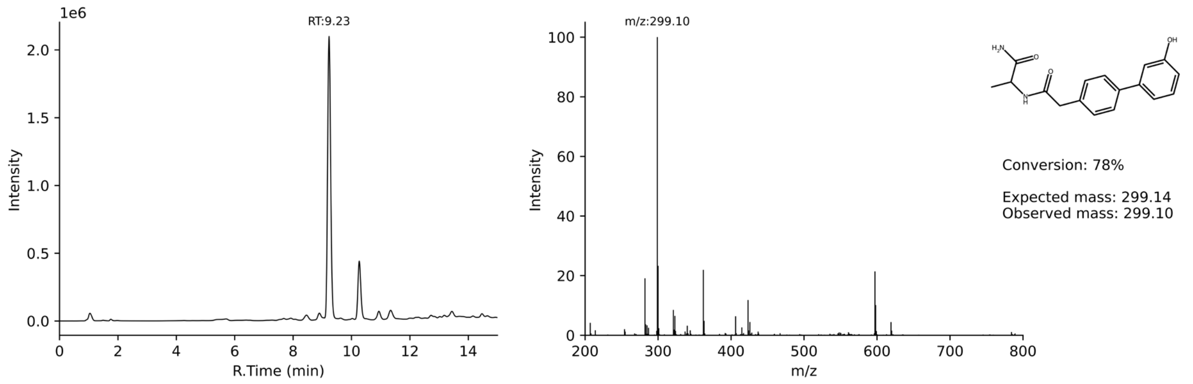

Supplementary Fig 291. LC-MS chromatogram obtained using (3-hydroxyphenyl)boronic acid.

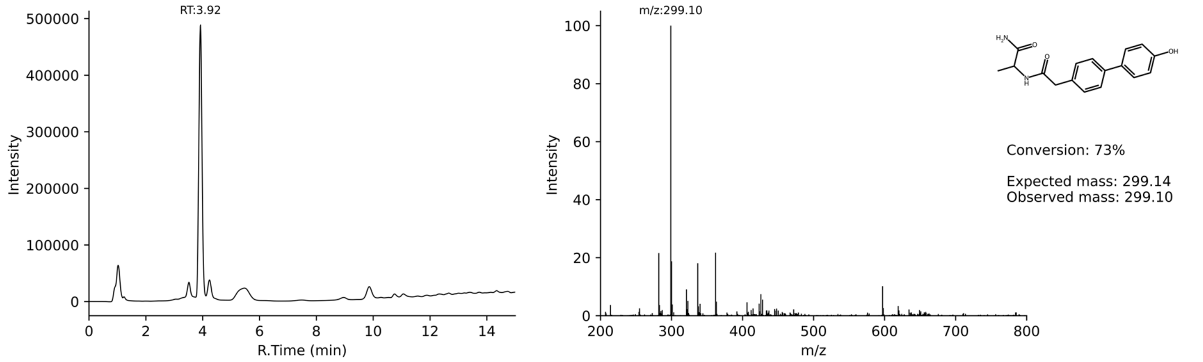

Supplementary Fig 292. LC-MS chromatogram obtained using (4-hydroxyphenyl)boronic acid.

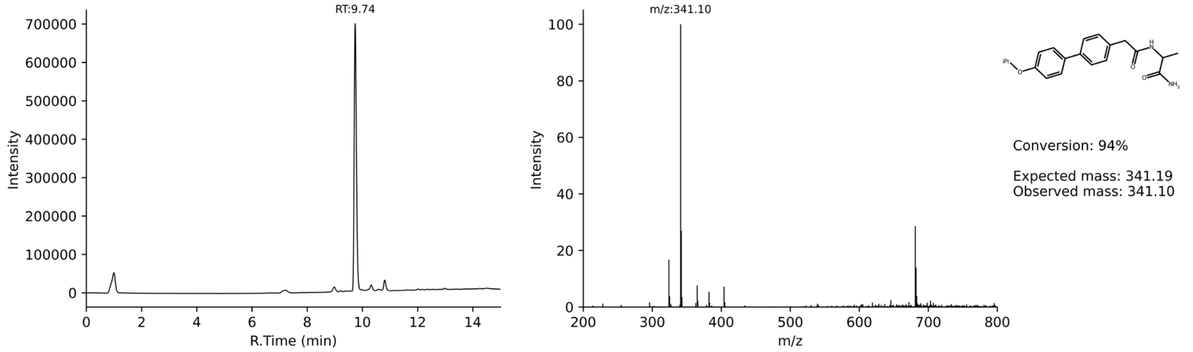

Supplementary Fig 293. LC-MS chromatogram obtained using (4-propan-2-yloxyphenyl)boronic acid.

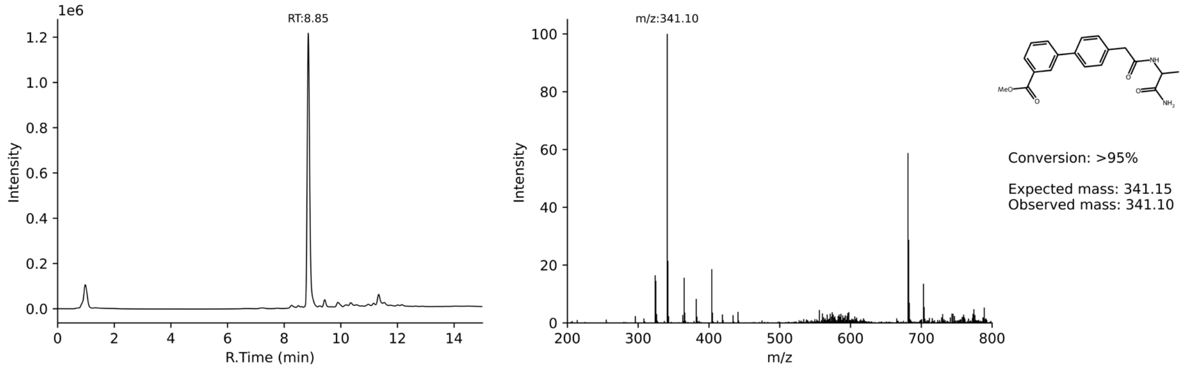

Supplementary Fig 294. LC-MS chromatogram obtained using (3-methoxycarbonylphenyl)boronic acid.

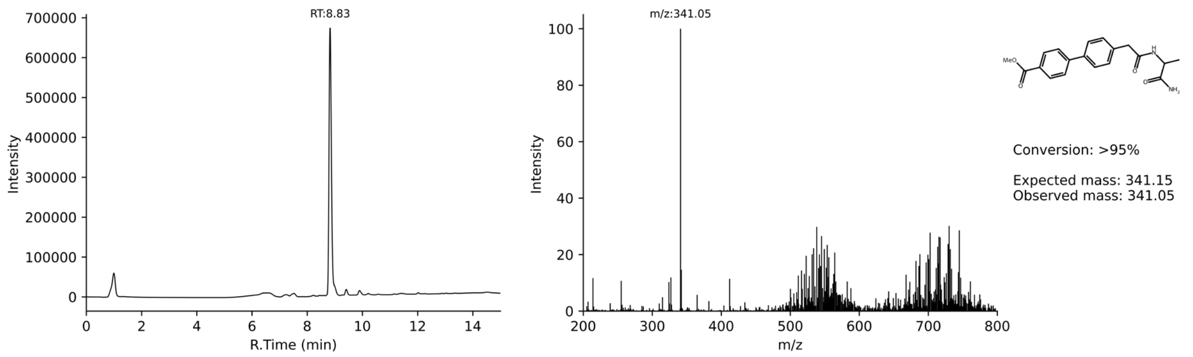

Supplementary Fig 295. LC-MS chromatogram obtained using (4-methoxycarbonylphenyl)boronic acid.

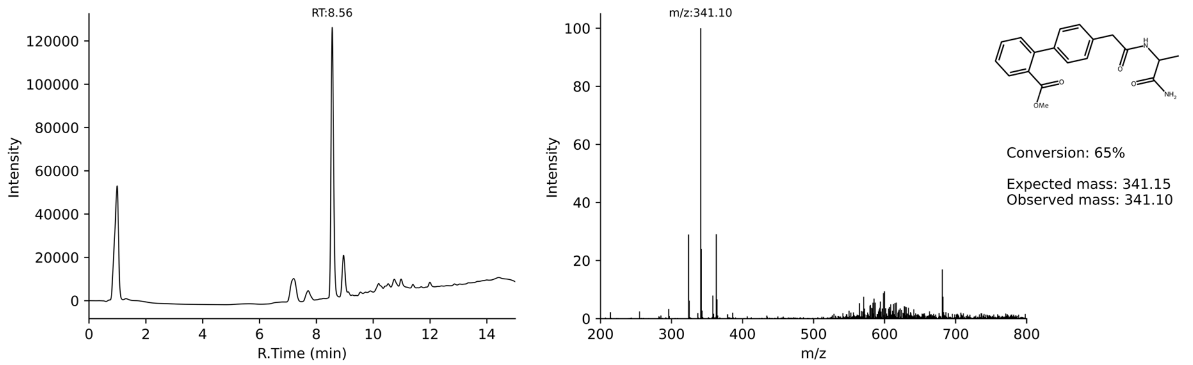

Supplementary Fig 296. LC-MS chromatogram obtained using (2-methoxycarbonylphenyl)boronic acid.

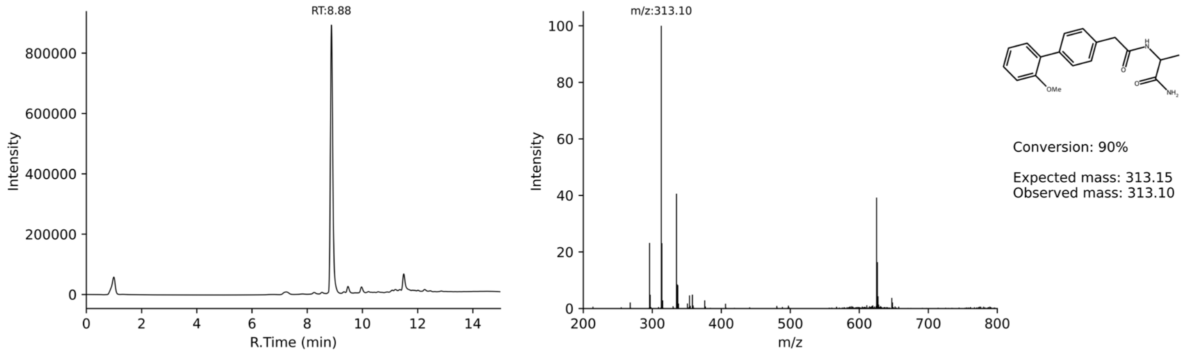

Supplementary Fig 297. LC-MS chromatogram obtained using (2-methoxyphenyl)boronic acid.

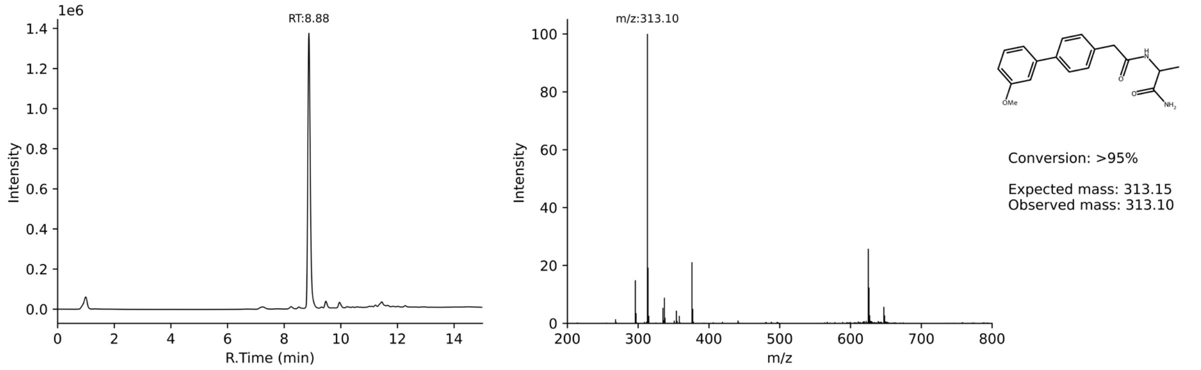

Supplementary Fig 298. LC-MS chromatogram obtained using (3-methoxyphenyl)boronic acid.

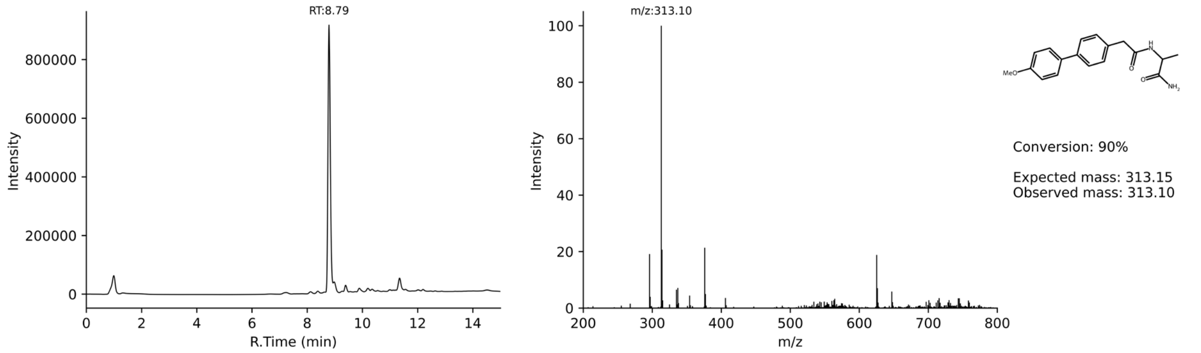

Supplementary Fig 299. LC-MS chromatogram obtained using (4-methoxyphenyl)boronic acid.

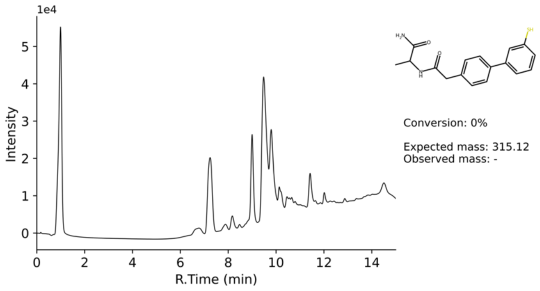

Supplementary Fig 300. LC-MS chromatogram obtained using (3-sulfanylphenyl)boronic acid.

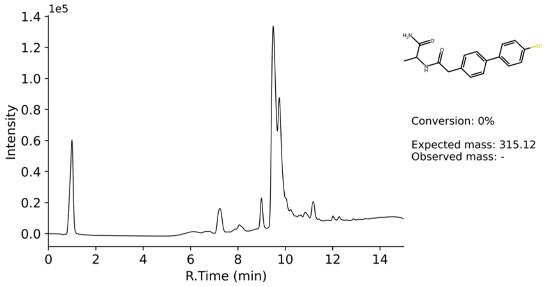

Supplementary Fig 301. LC-MS chromatogram obtained using (4-sulfanylphenyl)boronic acid.

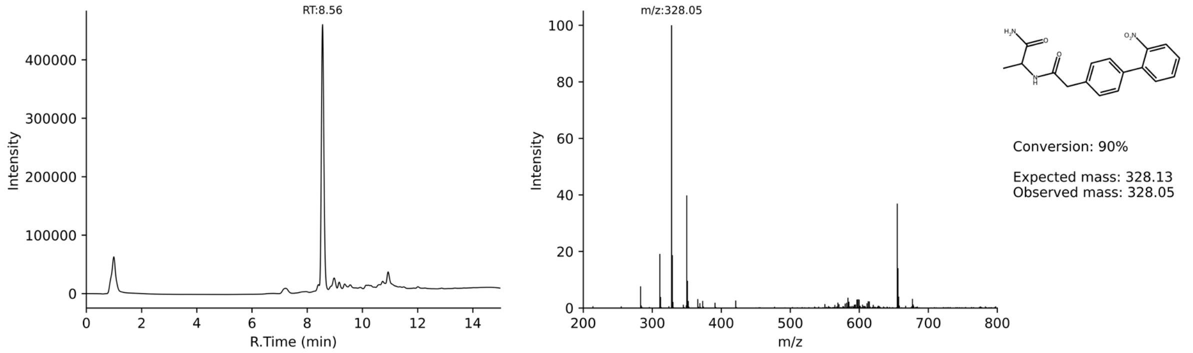

Supplementary Fig 302. LC-MS chromatogram obtained using (2-nitrophenyl)boronic acid.

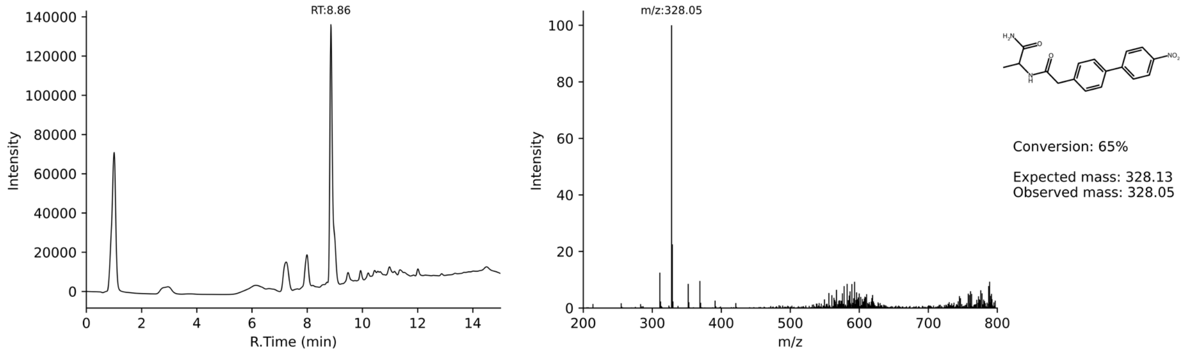

Supplementary Fig 303. LC-MS chromatogram obtained using (4-nitrophenyl)boronic acid.

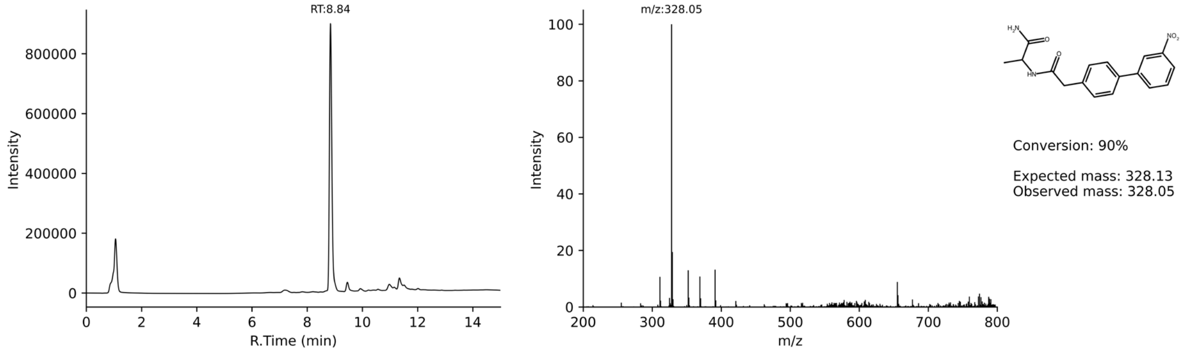

Supplementary Fig 304. LC-MS chromatogram obtained using (3-nitrophenyl)boronic acid.

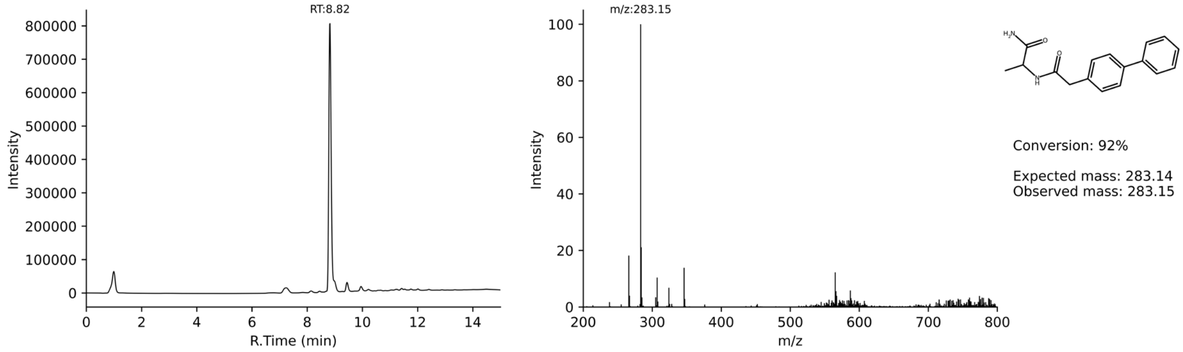

Supplementary Fig 305. LC-MS chromatogram obtained using phenylboronic acid.

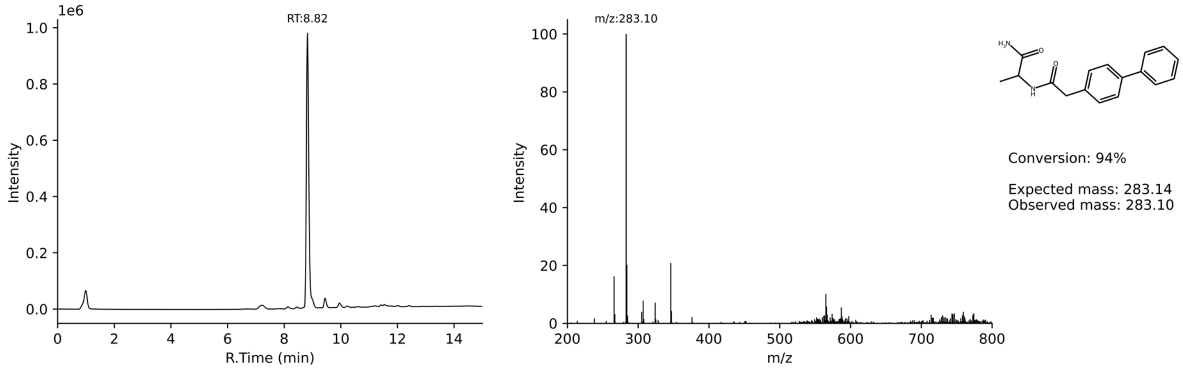

Supplementary Fig 306. LC-MS chromatogram obtained using 4,4,5,5-tetramethyl-2-phenyl-1,3,2-dioxaborolane.

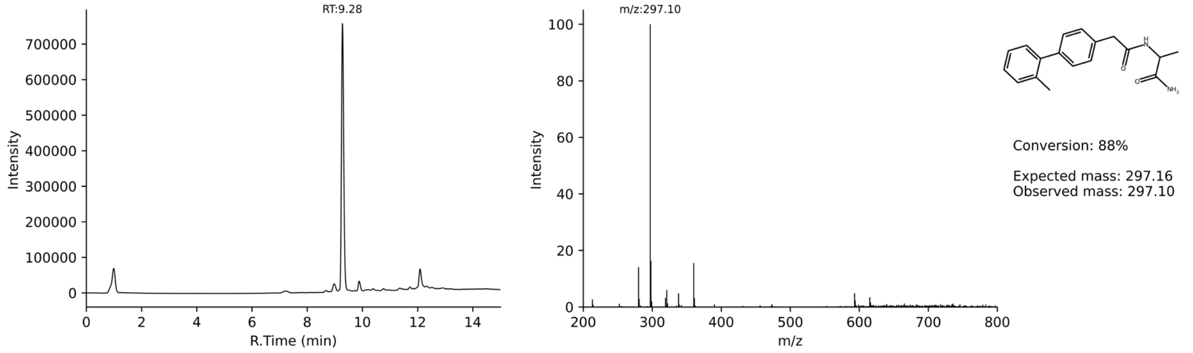

Supplementary Fig 307. LC-MS chromatogram obtained using (2-methylphenyl)boronic acid.

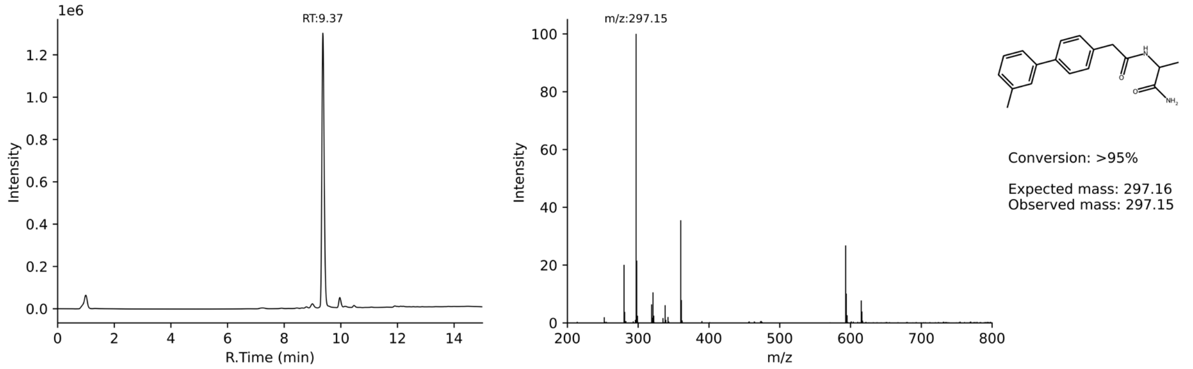

Supplementary Fig 308. LC-MS chromatogram obtained using (3-methylphenyl)boronic acid.

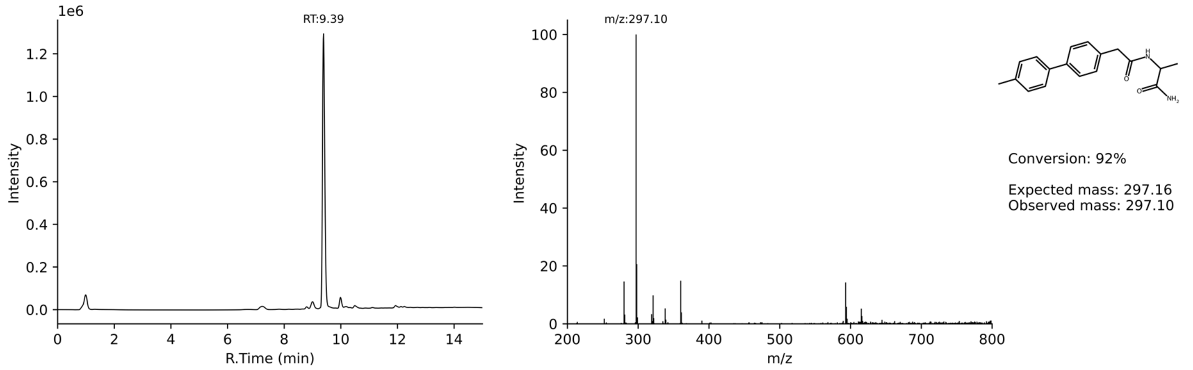

Supplementary Fig 309. LC-MS chromatogram obtained using (4-methylphenyl)boronic acid.

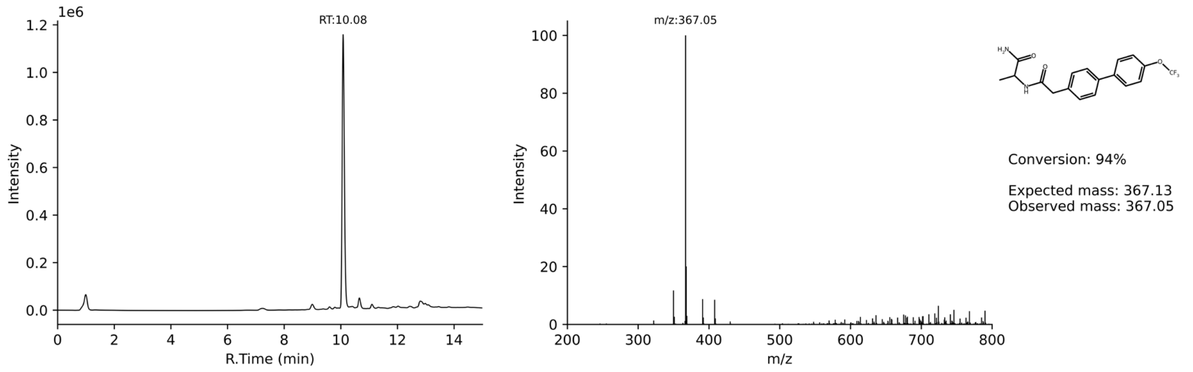

Supplementary Fig 310. LC-MS chromatogram obtained using [4-(trifluoromethoxy)phenyl]boronic acid.

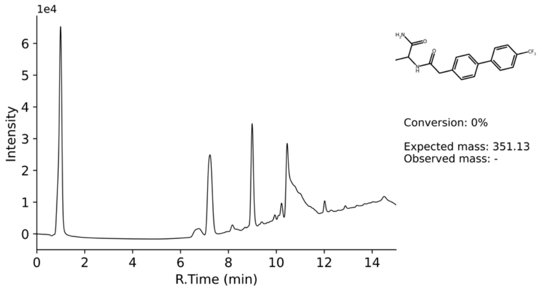

Supplementary Fig 311. LC-MS chromatogram obtained using [4-(trifluoromethyl)phenyl]boronic acid.

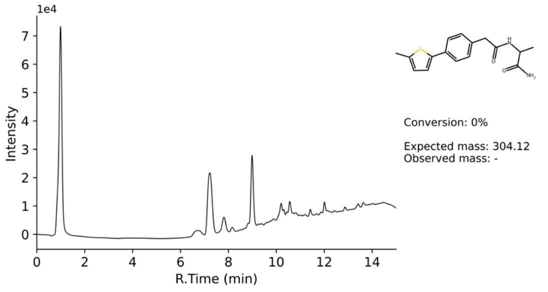

Supplementary Fig 312. LC-MS chromatogram obtained using (5-methylthiophen-2-yl)boronic acid.

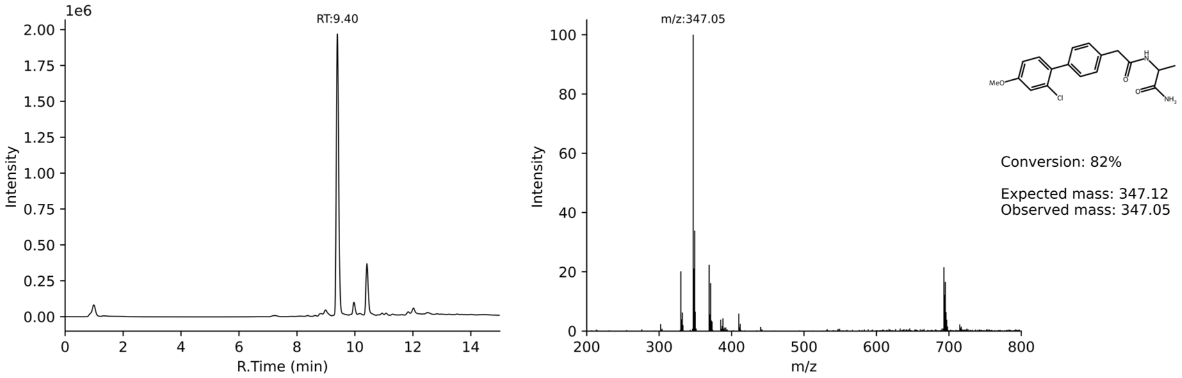

Supplementary Fig 313. LC-MS chromatogram obtained using (2-chloro-4-methoxyphenyl)boronic acid.

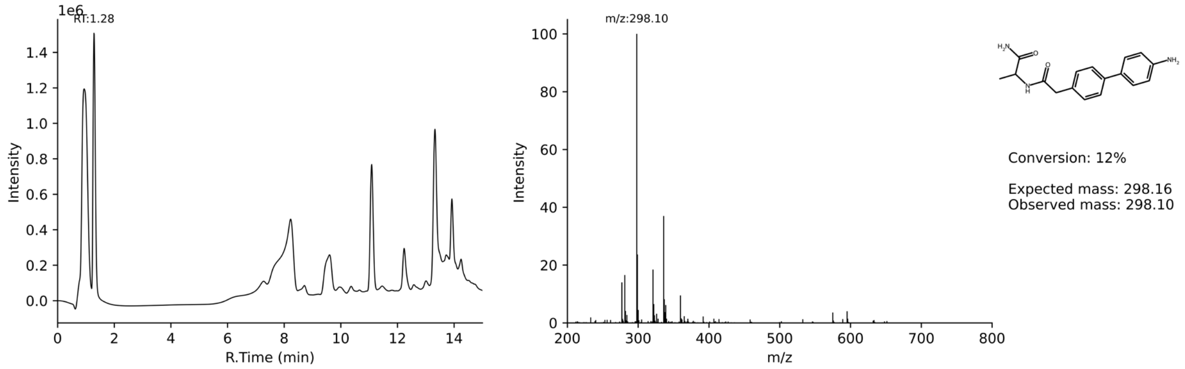

Supplementary Fig 314. LC-MS chromatogram obtained using 4-(4,4,5,5-tetramethyl-1,3,2-dioxaborolan-2-yl)aniline.

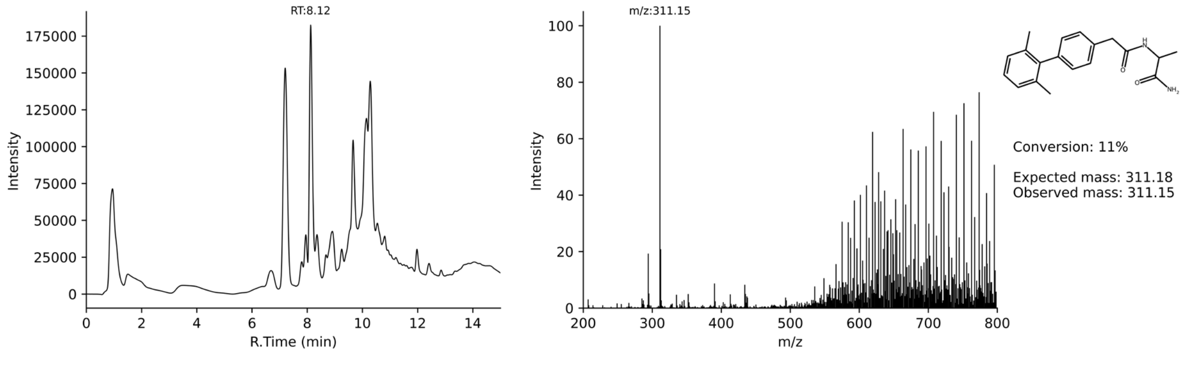

Supplementary Fig 315. LC-MS chromatogram obtained using (2,6-dimethylphenyl)boronic acid.

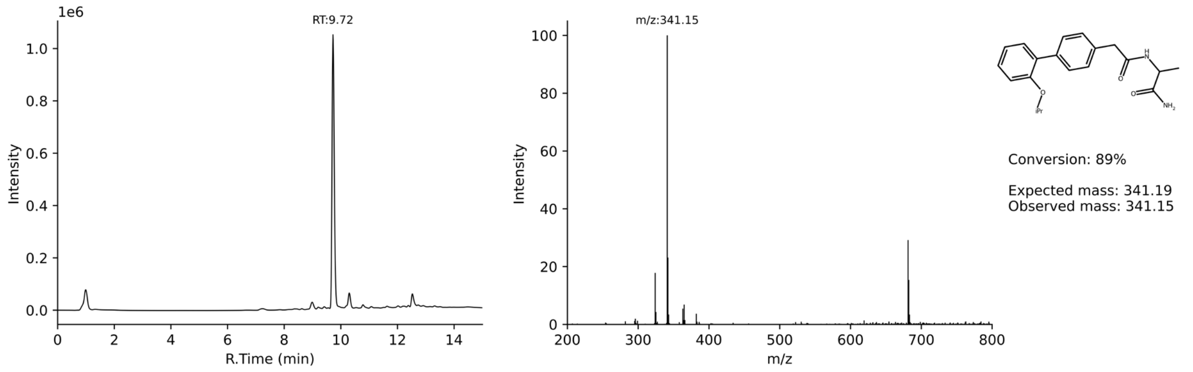

Supplementary Fig 316. LC-MS chromatogram obtained using (2-propan-2-yloxyphenyl)boronic acid.

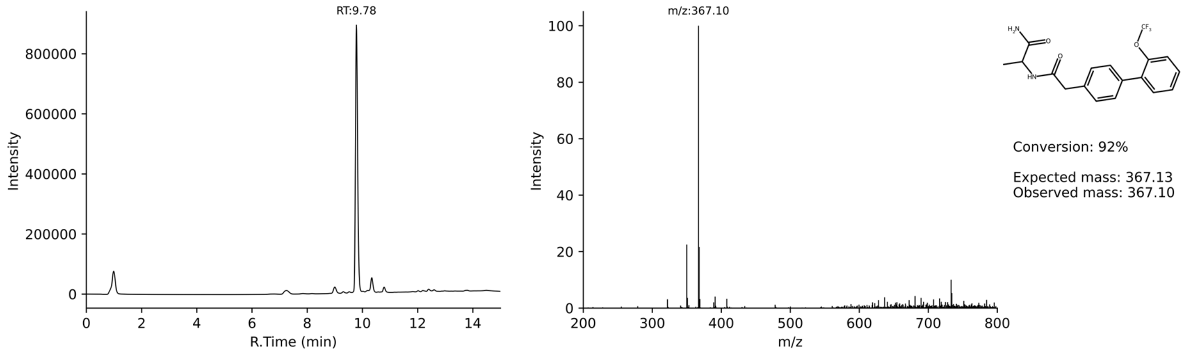

Supplementary Fig 317. LC-MS chromatogram obtained using [2-(trifluoromethoxy)phenyl]boronic acid.

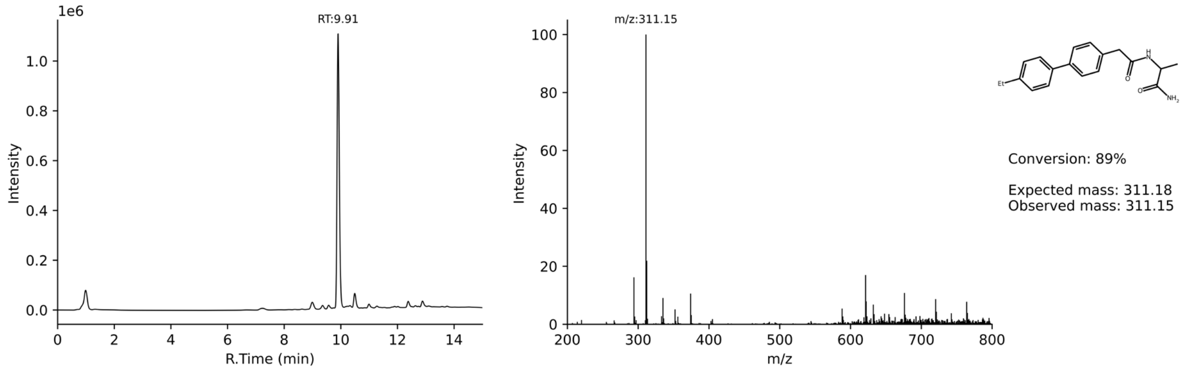

Supplementary Fig 318. LC-MS chromatogram obtained using (4-ethylphenyl)boronic acid.

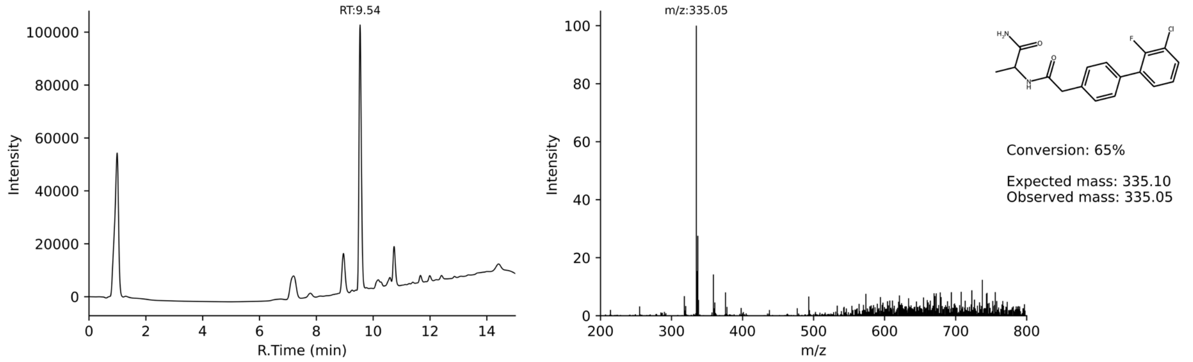

Supplementary Fig 319. LC-MS chromatogram obtained using (3-chloro-2-fluorophenyl)boronic acid.

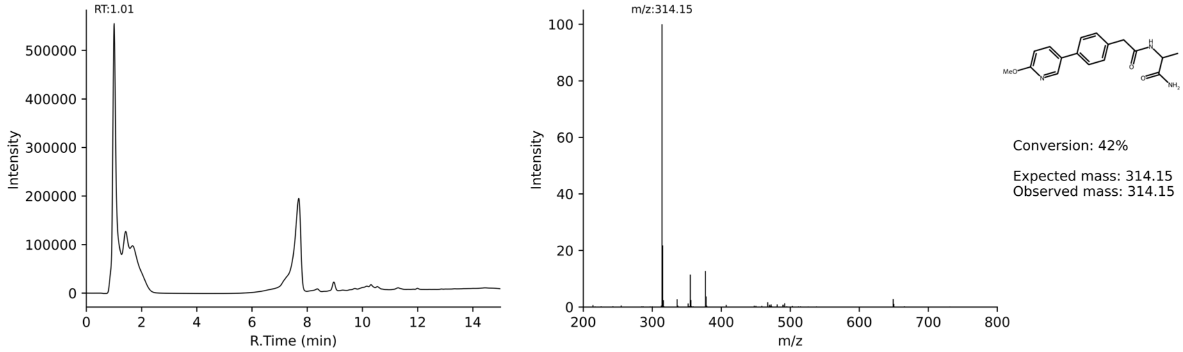

Supplementary Fig 320. LC-MS chromatogram obtained using (6-methoxypyridin-3-yl)boronic acid.

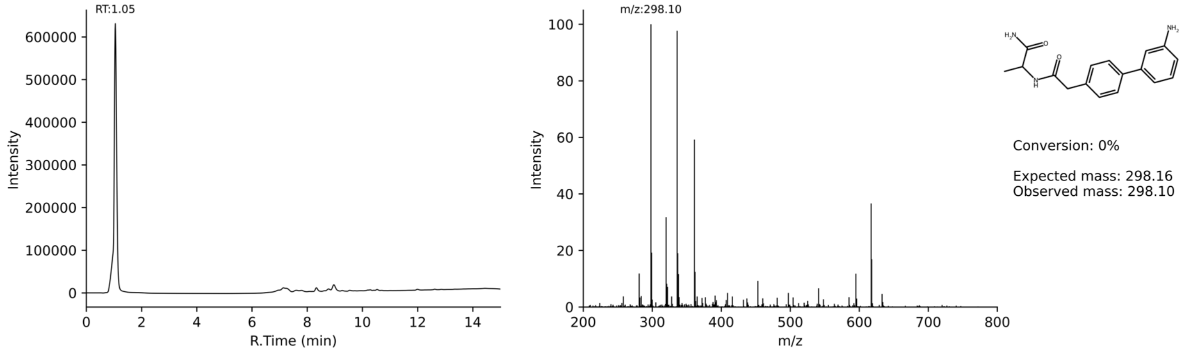

Supplementary Fig 321. LC-MS chromatogram obtained using (3-aminophenyl)boronic acid.

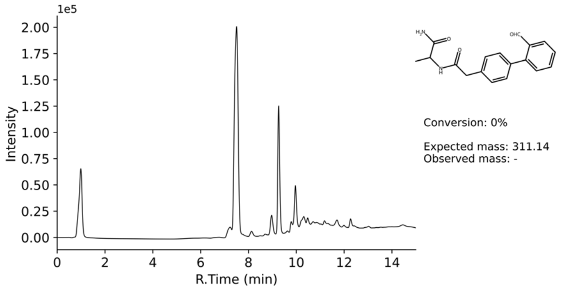

Supplementary Fig 322. LC-MS chromatogram obtained using (2-formylphenyl)boronic acid.

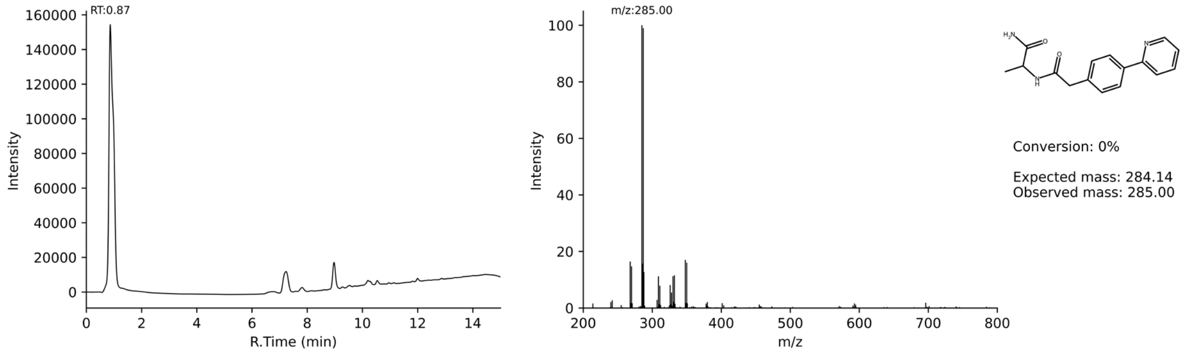

Supplementary Fig 323. LC-MS chromatogram obtained using pyridin-2-ylboronic acid.

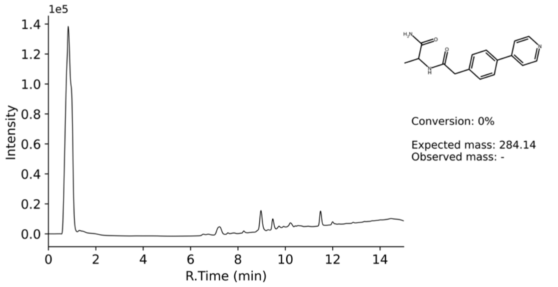

Supplementary Fig 324. LC-MS chromatogram obtained using pyridin-4-ylboronic acid.

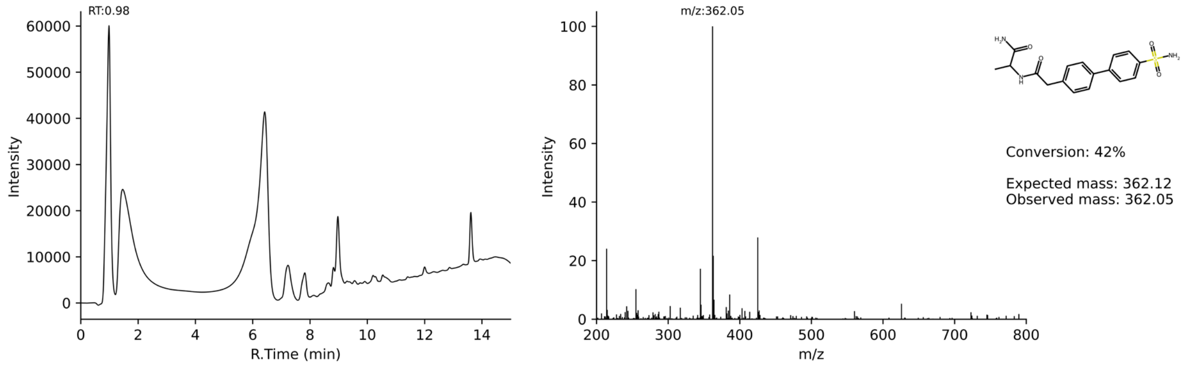

Supplementary Fig 325. LC-MS chromatogram obtained using (4-sulfamoylphenyl)boronic acid.

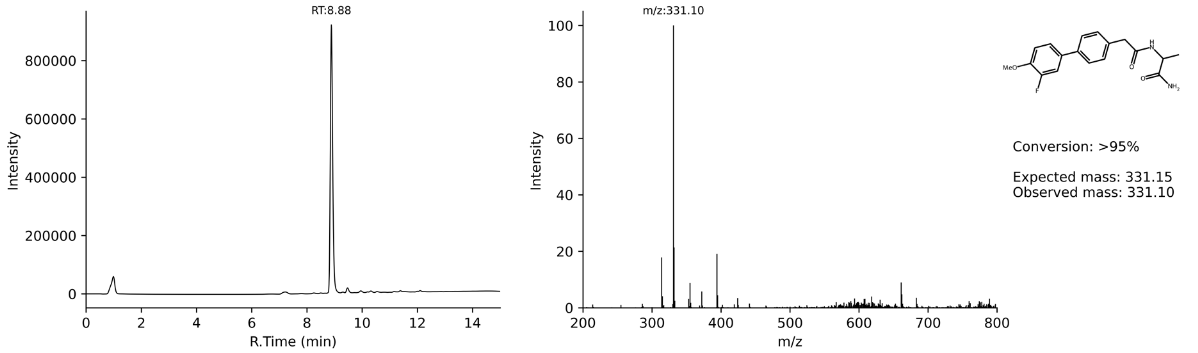

Supplementary Fig 326. LC-MS chromatogram obtained using (3-fluoro-4-methoxyphenyl)boronic acid.

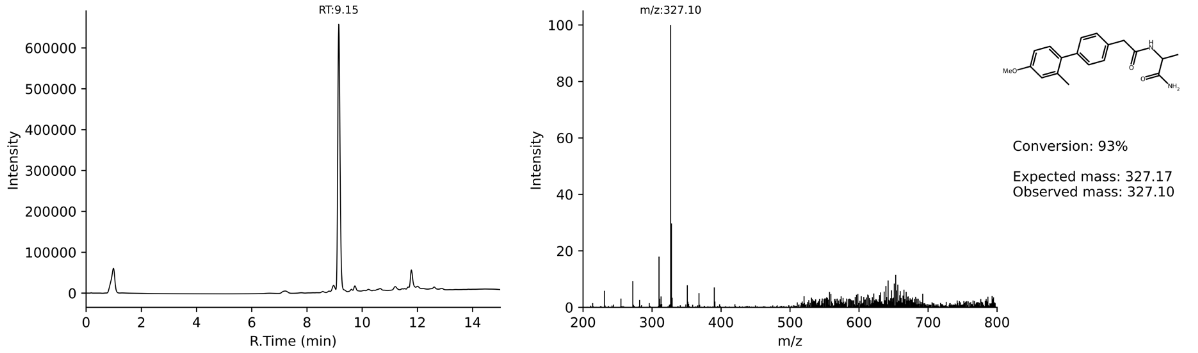

Supplementary Fig 327. LC-MS chromatogram obtained using (4-methoxy-2-methylphenyl)boronic acid.

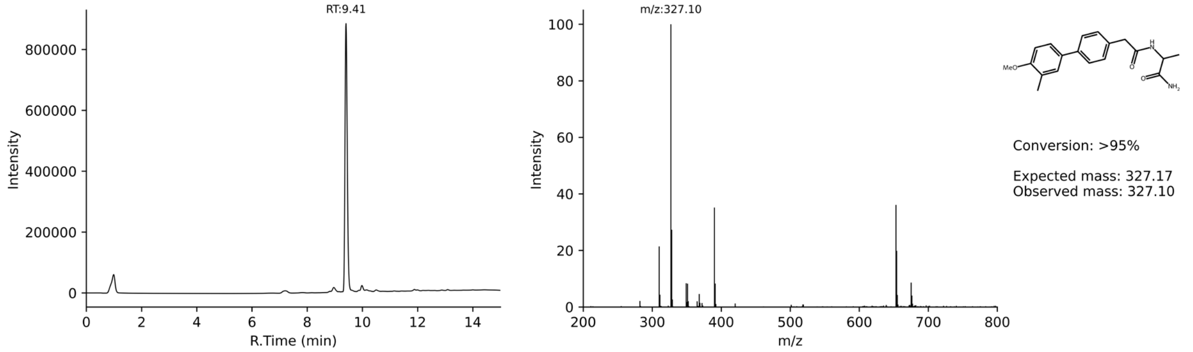

Supplementary Fig 328. LC-MS chromatogram obtained using (4-methoxy-3-methylphenyl)boronic acid.

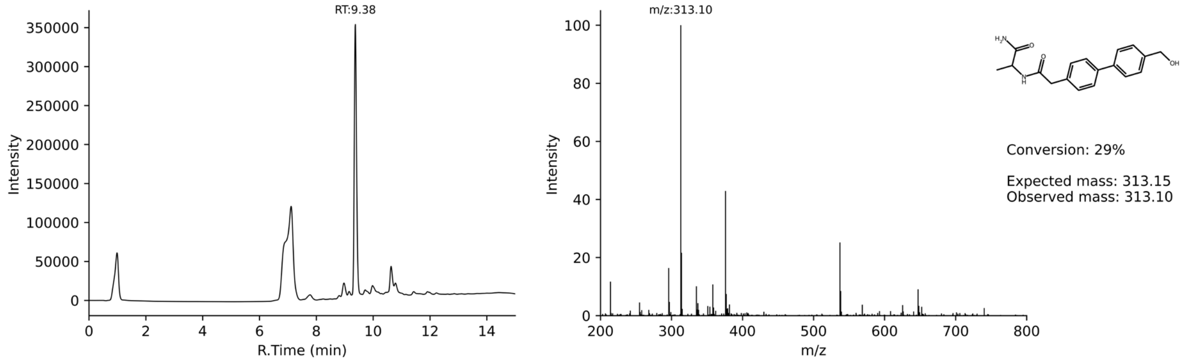

Supplementary Fig 329. LC-MS chromatogram obtained using [4-(hydroxymethyl)phenyl]boronic acid.

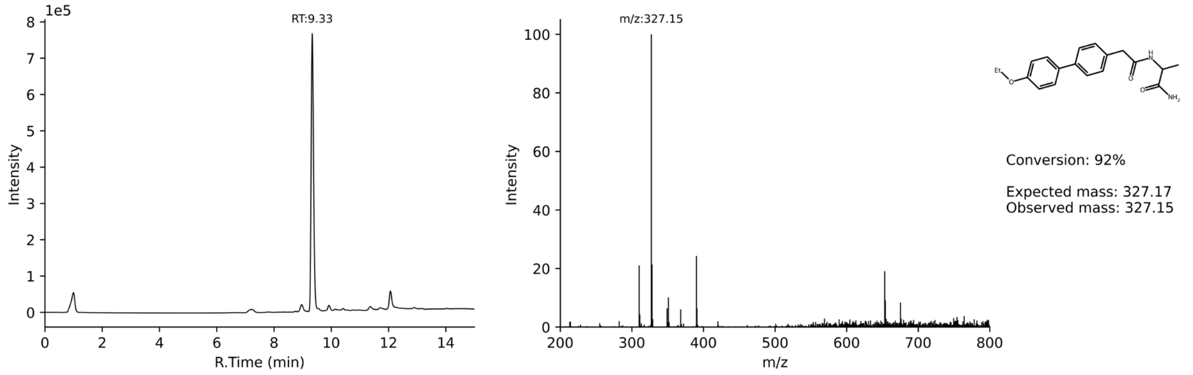

Supplementary Fig 330. LC-MS chromatogram obtained using (4-ethoxyphenyl)boronic acid

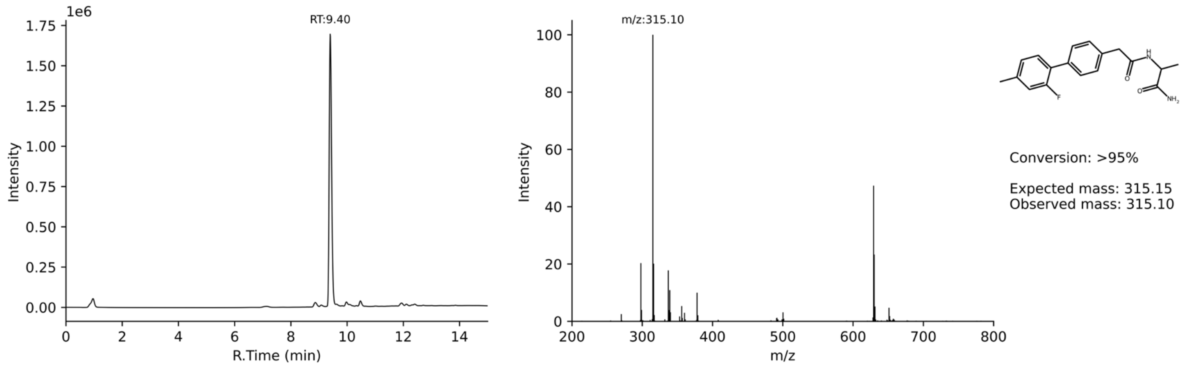

Supplementary Fig 331. LC-MS chromatogram obtained using (2-fluoro-4-methylphenyl)boronic acid.

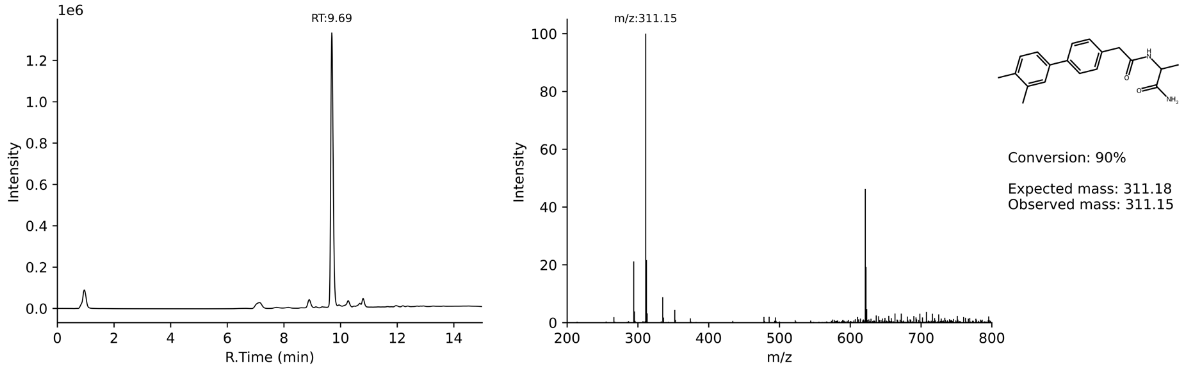

Supplementary Fig 332. LC-MS chromatogram obtained using (3,4-dimethylphenyl)boronic acid.

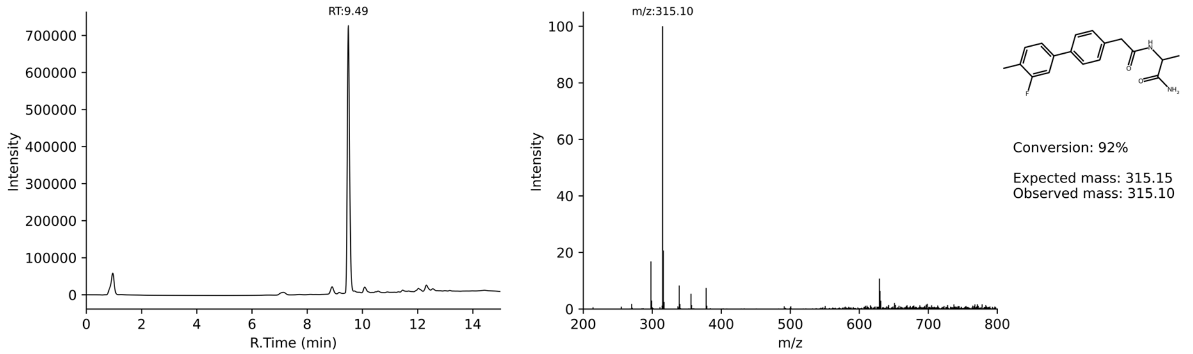

Supplementary Fig 333. LC-MS chromatogram obtained using (3-fluoro-4-methylphenyl)boronic acid.

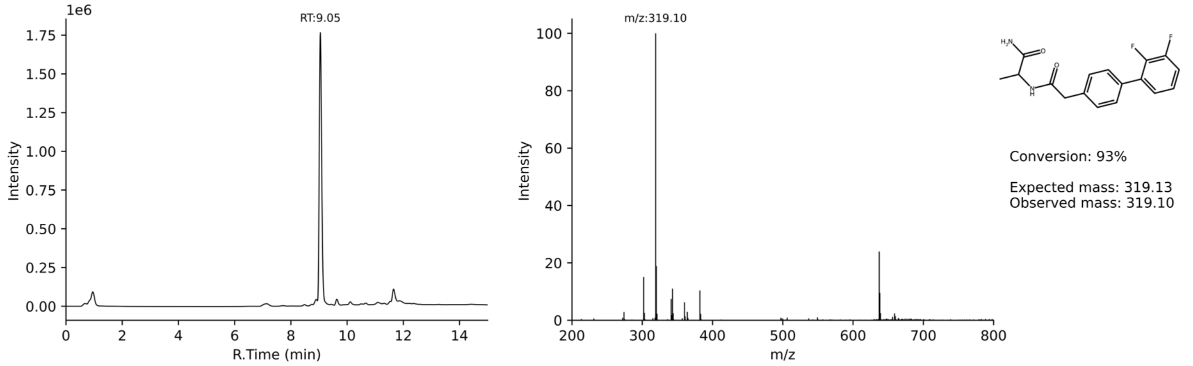

Supplementary Fig 334. LC-MS chromatogram obtained using (2,3-difluorophenyl)boronic acid.

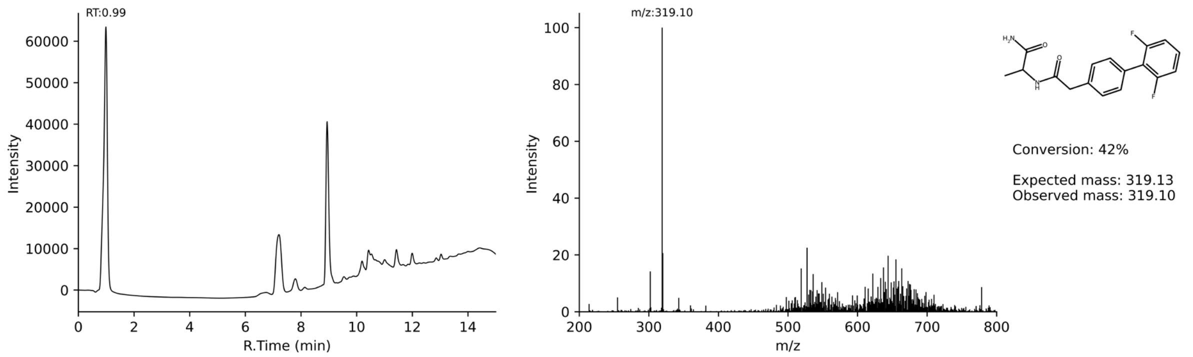

Supplementary Fig 335. LC-MS chromatogram obtained using (2,6-difluorophenyl)boronic acid.

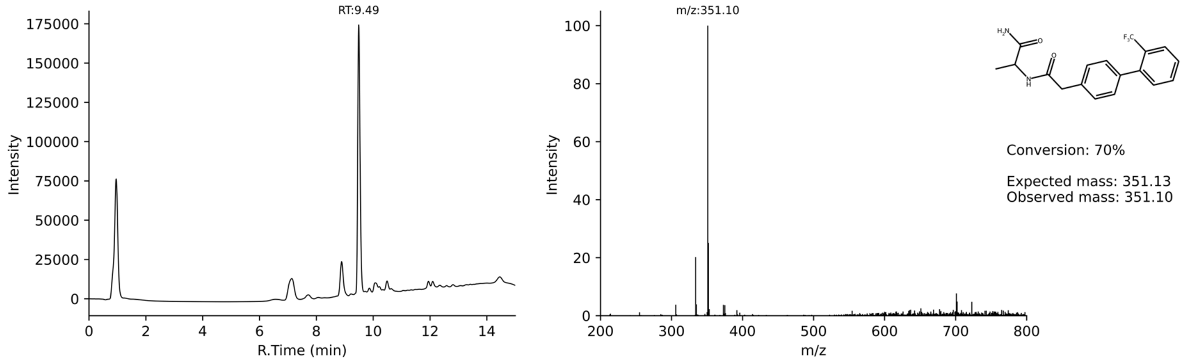

Supplementary Fig 336. LC-MS chromatogram obtained using [2-(trifluoromethyl)phenyl]boronic acid.

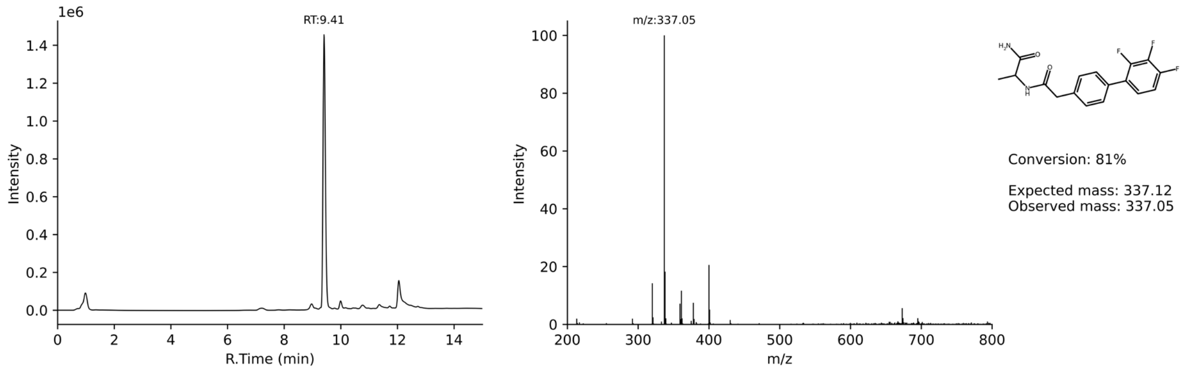

Supplementary Fig 337. LC-MS chromatogram obtained using (2,3,4-trifluorophenyl)boronic acid.

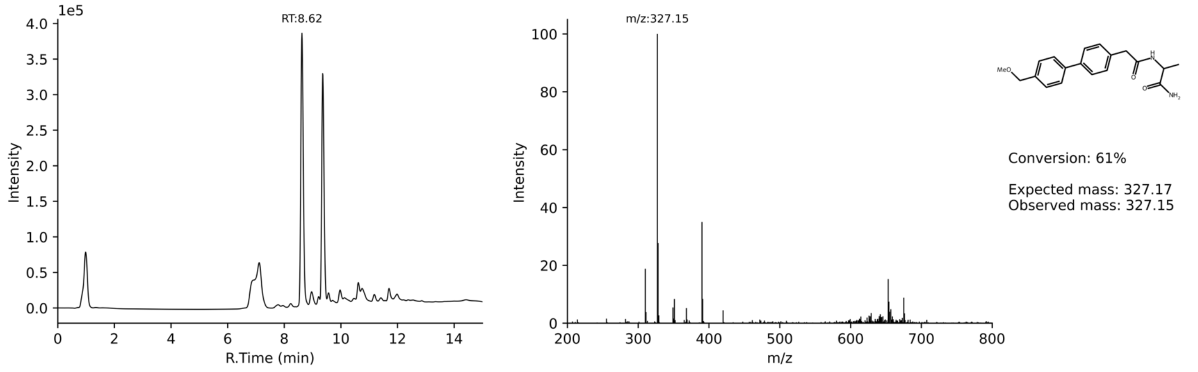

Supplementary Fig 338. LC-MS chromatogram obtained using [4-(methoxymethyl)phenyl]boronic acid.

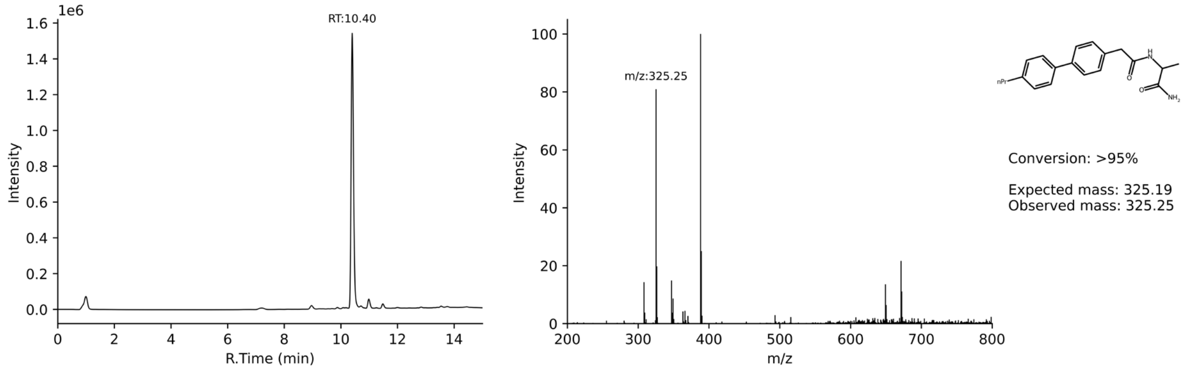

Supplementary Fig 339. LC-MS chromatogram obtained using (4-propylphenyl)boronic acid.

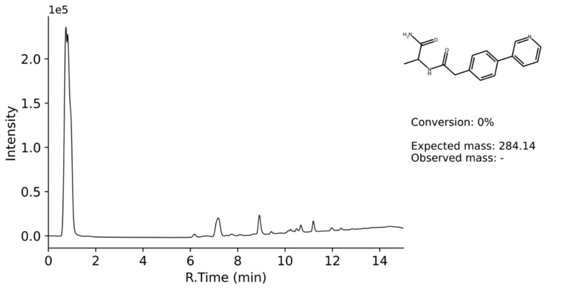

Supplementary Fig 340. LC-MS chromatogram obtained using pyridin-3-ylboronic acid.

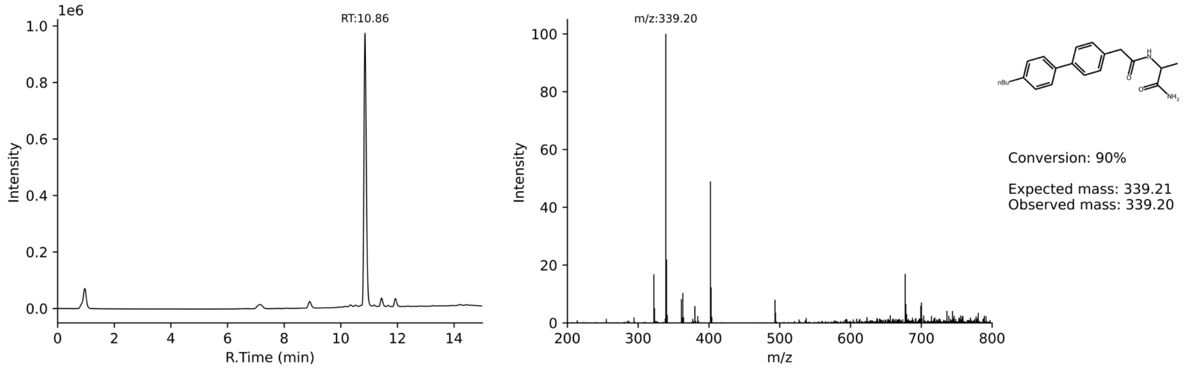

Supplementary Fig 341. LC-MS chromatogram obtained using (4-butylphenyl)boronic acid.

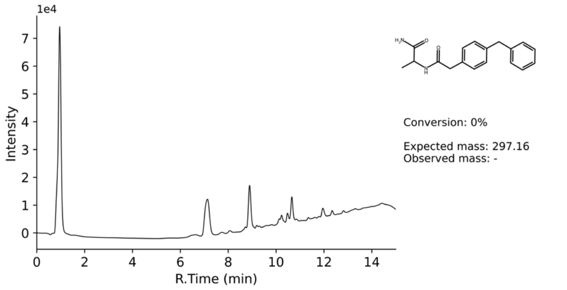

Supplementary Fig 342. LC-MS chromatogram obtained using benzylboronic acid pinacol ester.

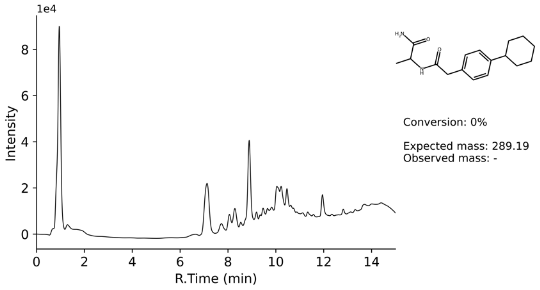

Supplementary Fig 343. LC-MS chromatogram obtained using cyclohexyl boronic acid.

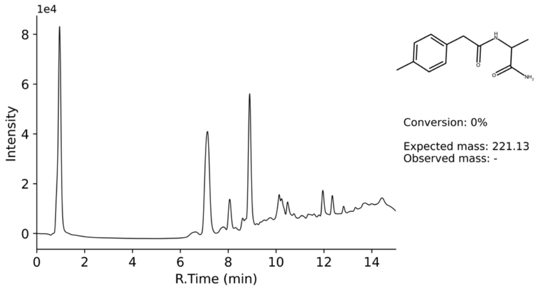

Supplementary Fig 344. LC-MS chromatogram obtained using methylboronic acid.

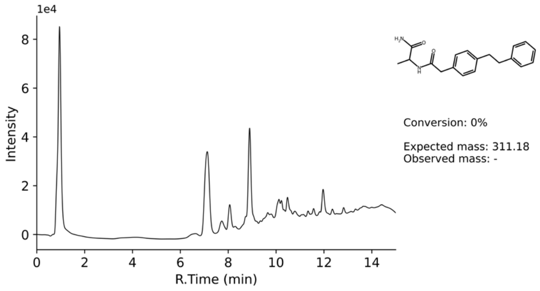

Supplementary Fig 345. LC-MS chromatogram obtained using phenethylboronic acid.

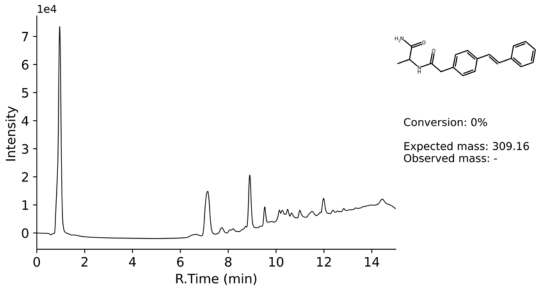

Supplementary Fig 346. LC-MS chromatogram obtained using trans-2-phenylvinylboronic acid.

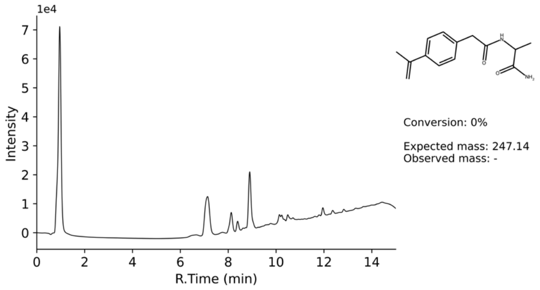

Supplementary Fig 347. LC-MS chromatogram obtained using isopropenylboronic acid pinacol ester.

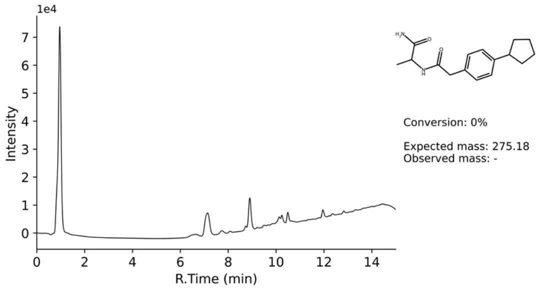

Supplementary Fig 348. LC-MS chromatogram obtained using cyclopentylboronic acid.

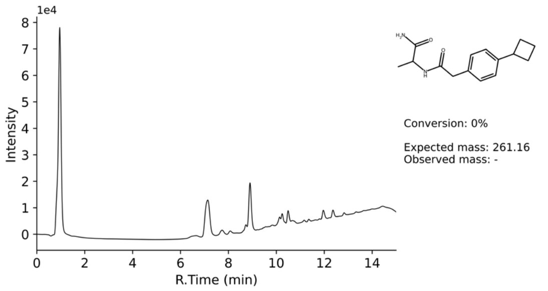

Supplementary Fig 349. LC-MS chromatogram obtained using cyclobutylboronic acid.

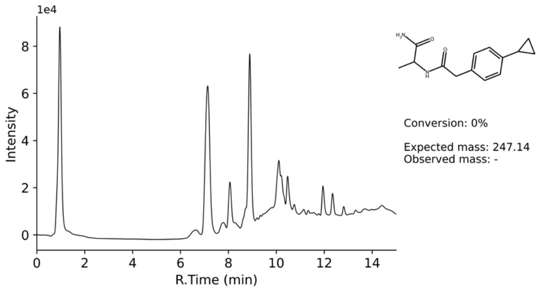

Supplementary Fig 350. LC-MS chromatogram obtained using Cyclopropyl boronic acid.

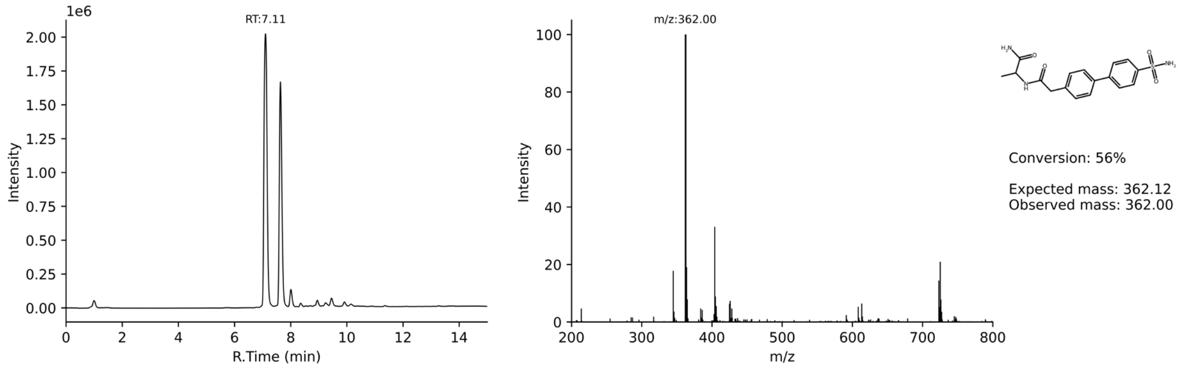

Supplementary Fig 351. LC-MS chromatogram obtained using 4-(N-(tert-Butyl)sulfamoyl)phenyl)boronic acid.

12.5 LC-MS data from the synthesis of CA131 and CA132

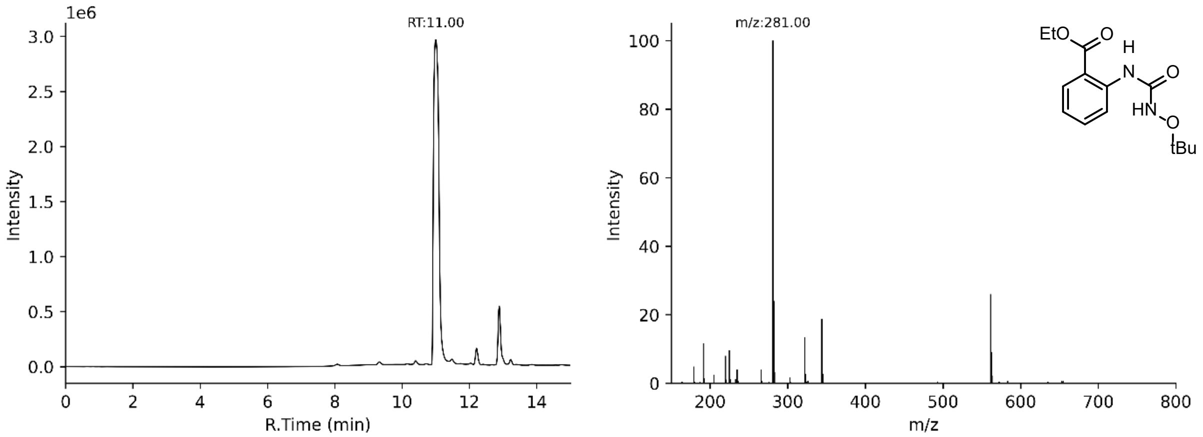

Supplementary Fig 352. LC-MS chromatogram of compound 21.

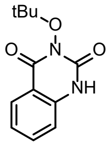

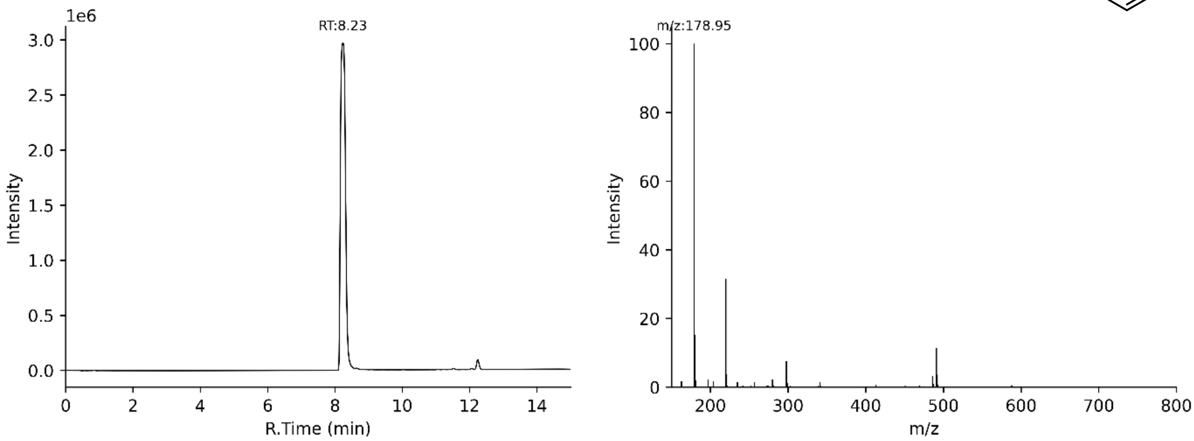

Supplementary Fig 353. LC-MS chromatogram of compound 22.

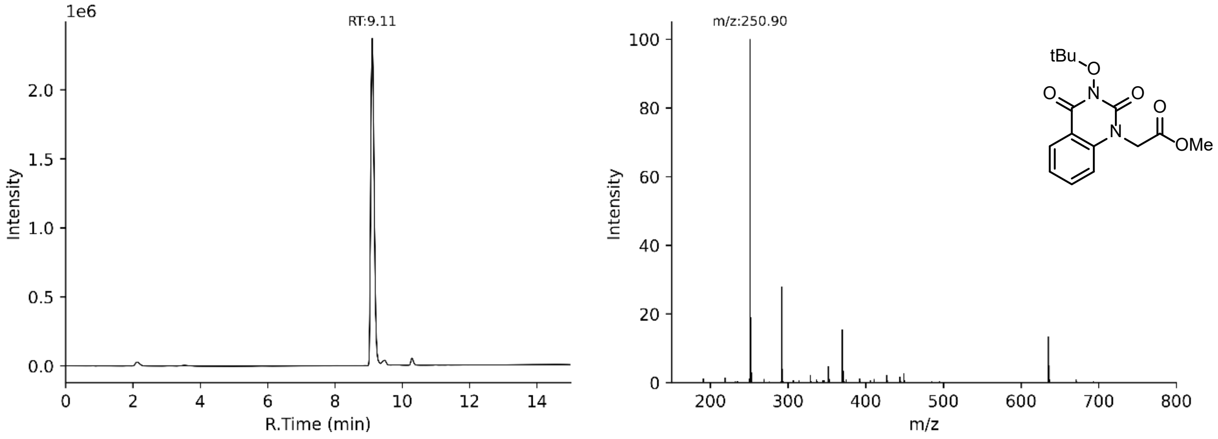

Supplementary Fig 354. LC-MS chromatogram of compound 23.

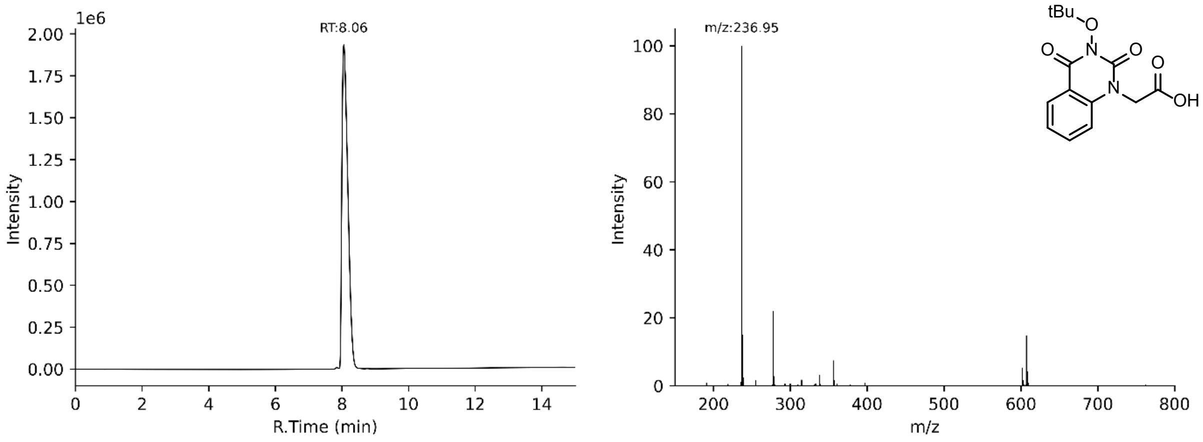

Supplementary Fig 355. LC-MS chromatogram of compound 24.
